# Supplementary material for: Site-selective C-H hydroxylation of pentacyclic triterpenoids directed by transient chiral pyridine-imino groups
Source: Nat Commun. 2020 Sep 1;11:4371. doi: 10.1038/s41467-020-18138-9 (PMC7462855; doi:10.1038/s41467-020-18138-9)
Supplement: Supplementary file 1 — Supplementary Information [file 41467_2020_18138_MOESM1_ESM.pdf]

SUPPLEMENTARY INFORMATION

Site-Selective C-H Hydroxylation of Pentacyclic Triterpenoids Directed by Transient Chiral Pyridine-Imino Groups

Mu et al.

Supplementary Figures

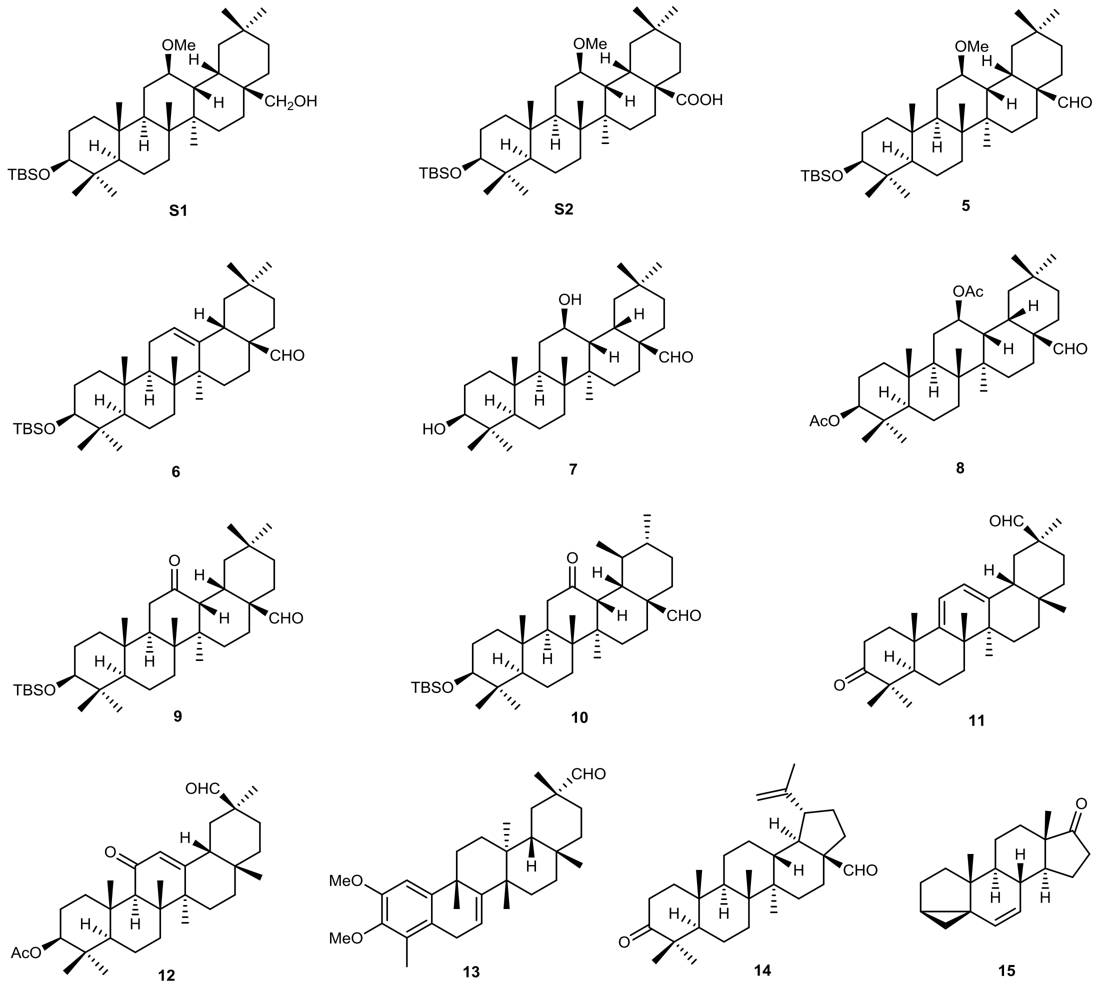

Supplementary Figure 1. PTs substrates used for C-H oxidation in this work.

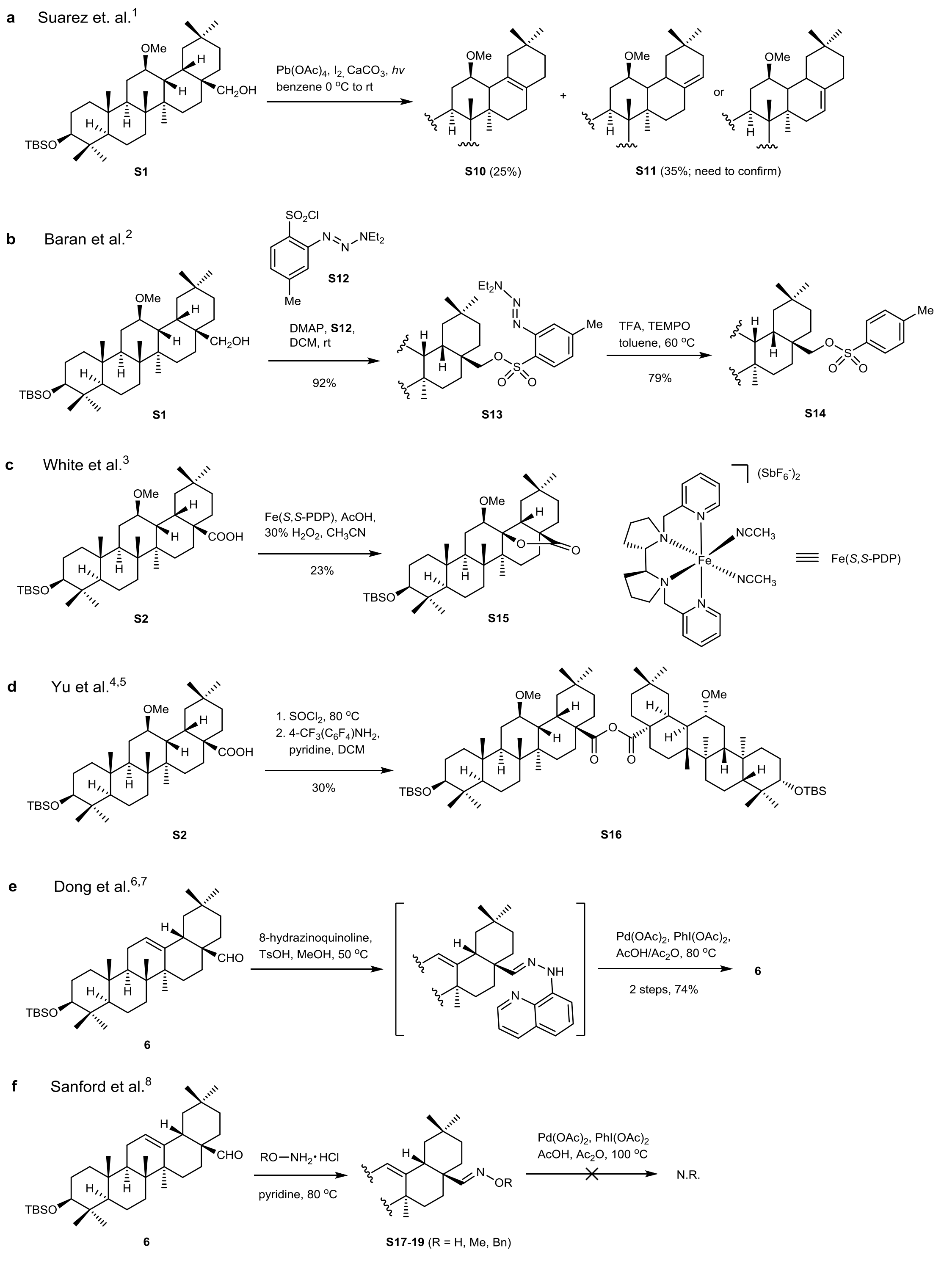

Supplementary Figure 2. Unsuccessful attempts at directed C-H oxidation on PTs.

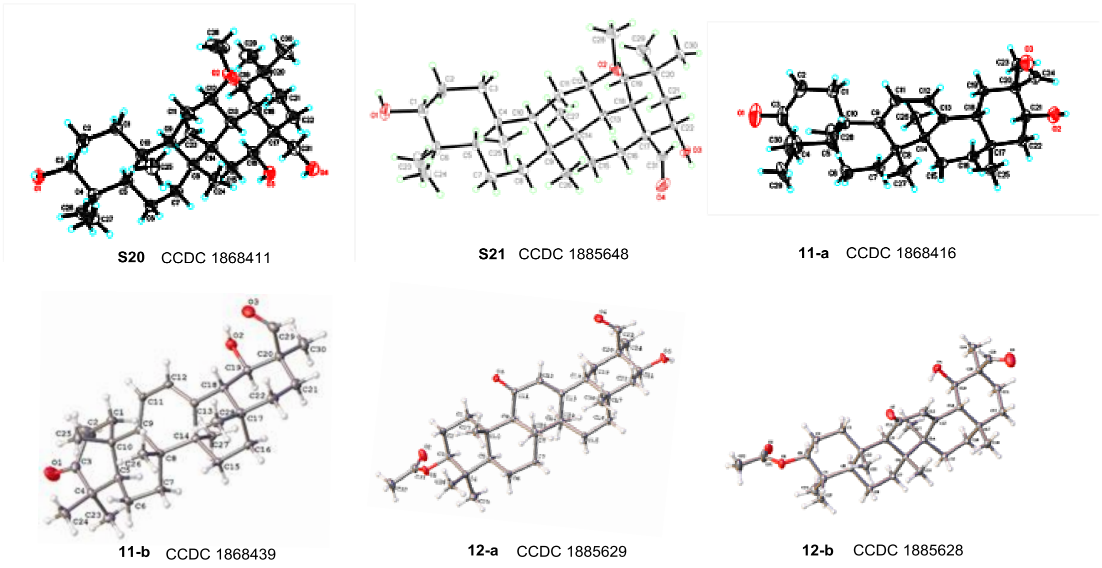

Supplementary Figure 3. X-Ray diffraction structures of S20, S21, 11-a, 11-b, 12-a, and 12-b.

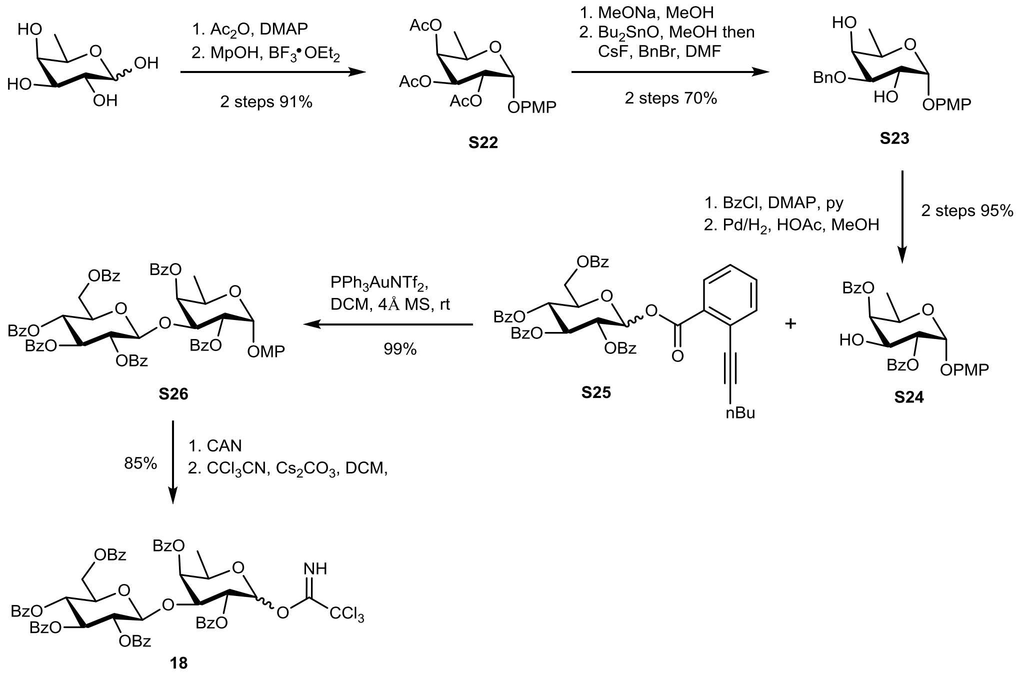

Supplementary Figure 4. Synthesis of disaccharide donor 18.

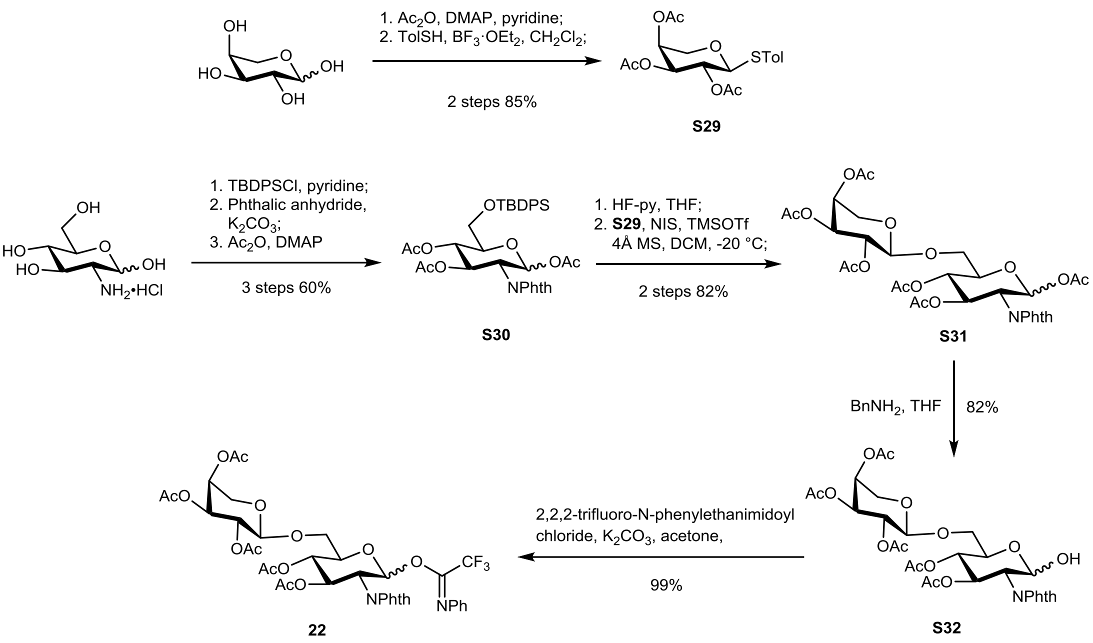

Supplementary Figure 5. Synthesis of disaccharide donor 22.

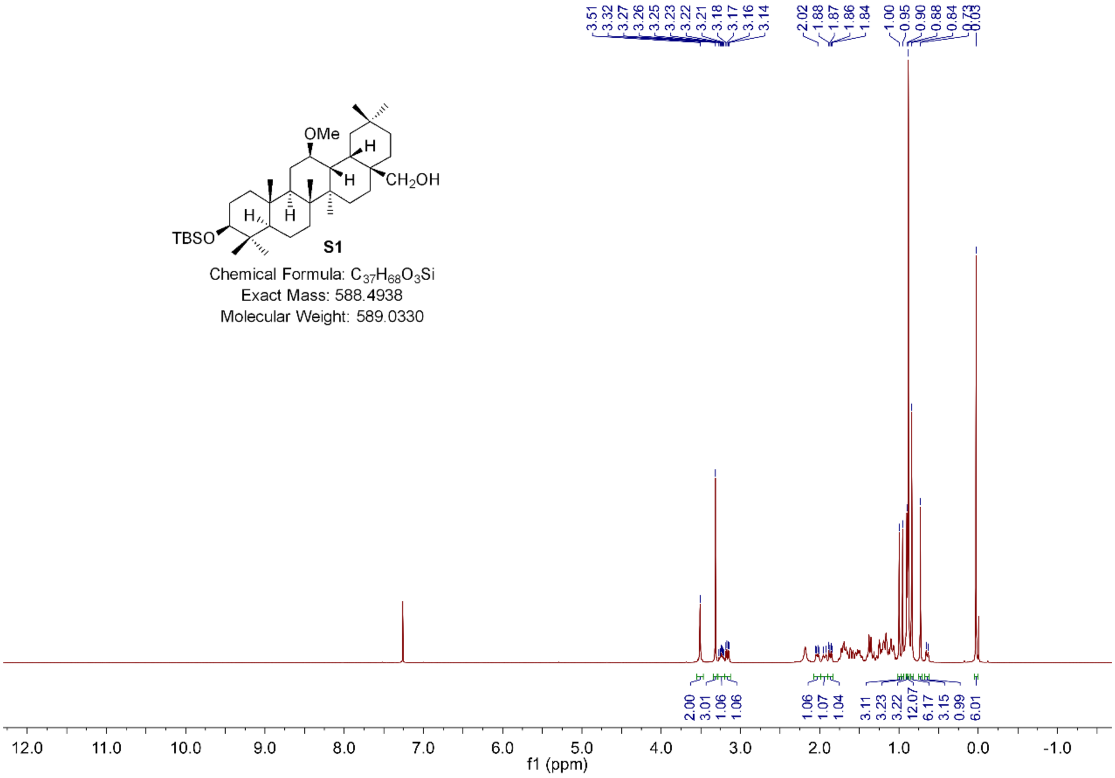

Supplementary Figure 6. ^{1}H-NMR spectrum of compound S1 (400 MHz, $CDCl_3$, 25 ^{o}C)

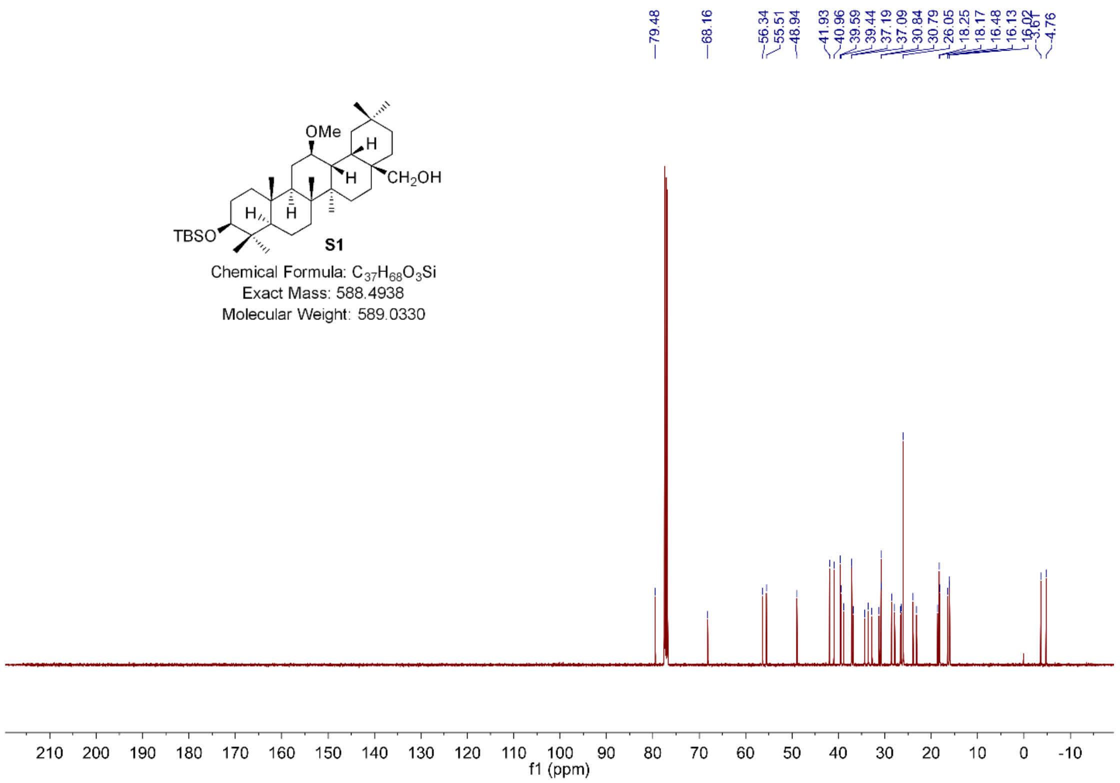

Supplementary Figure 7. ^{13}C-NMR spectrum of compound S1 (101 MHz, $CDCl_3$, 25 ^{o}C)

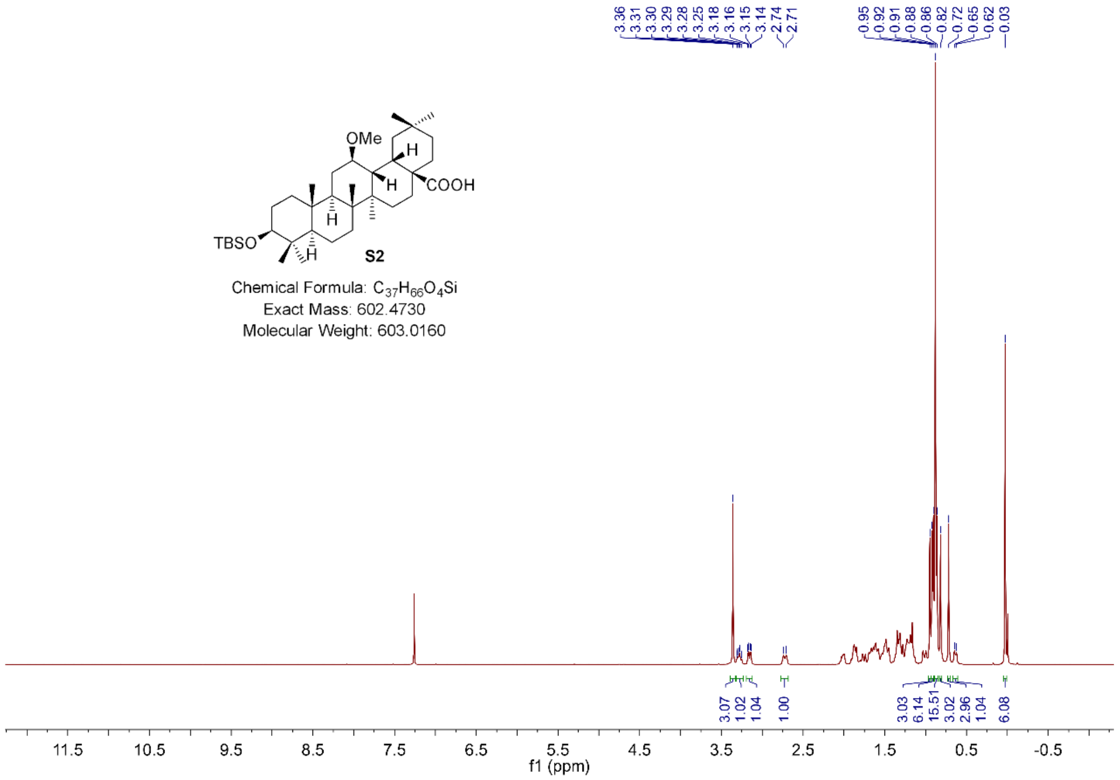

Supplementary Figure 8. ^{1}H-NMR spectrum of compound S2 (400 MHz, $CDCl_3$, 25 ^{o}C)

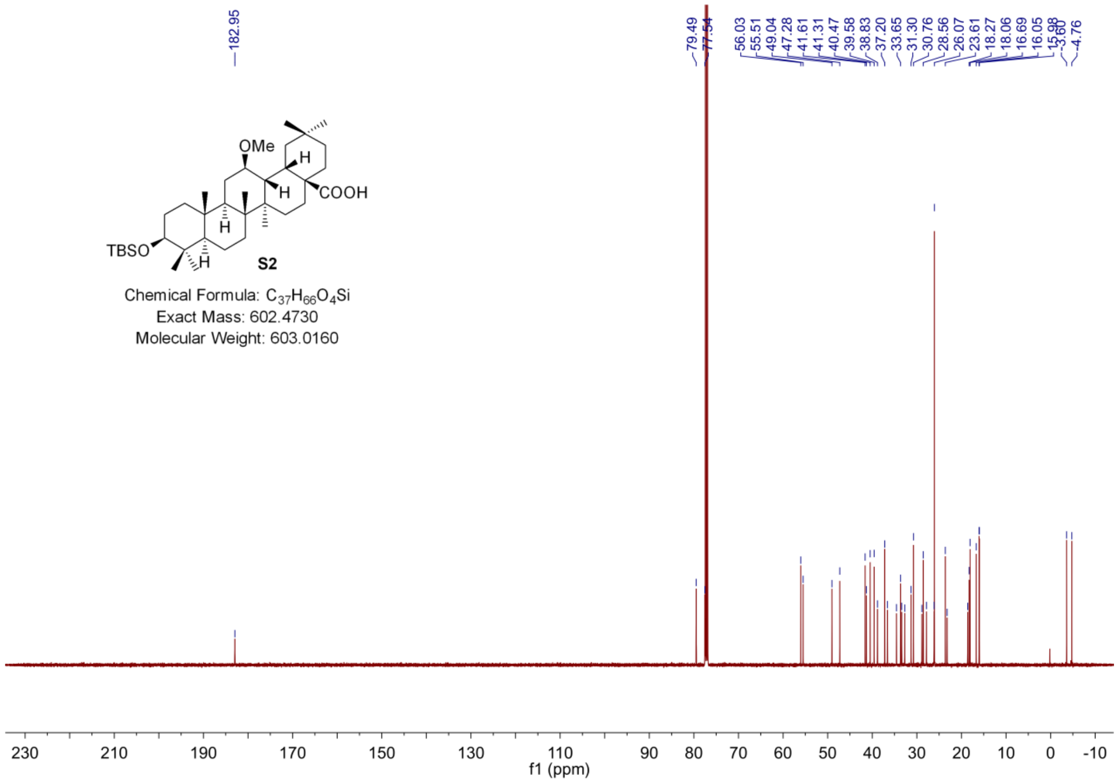

Supplementary Figure 9. ^{13}C-NMR spectrum of compound S2 (126 MHz, $CDCl_3$, 25 ^{o}C)

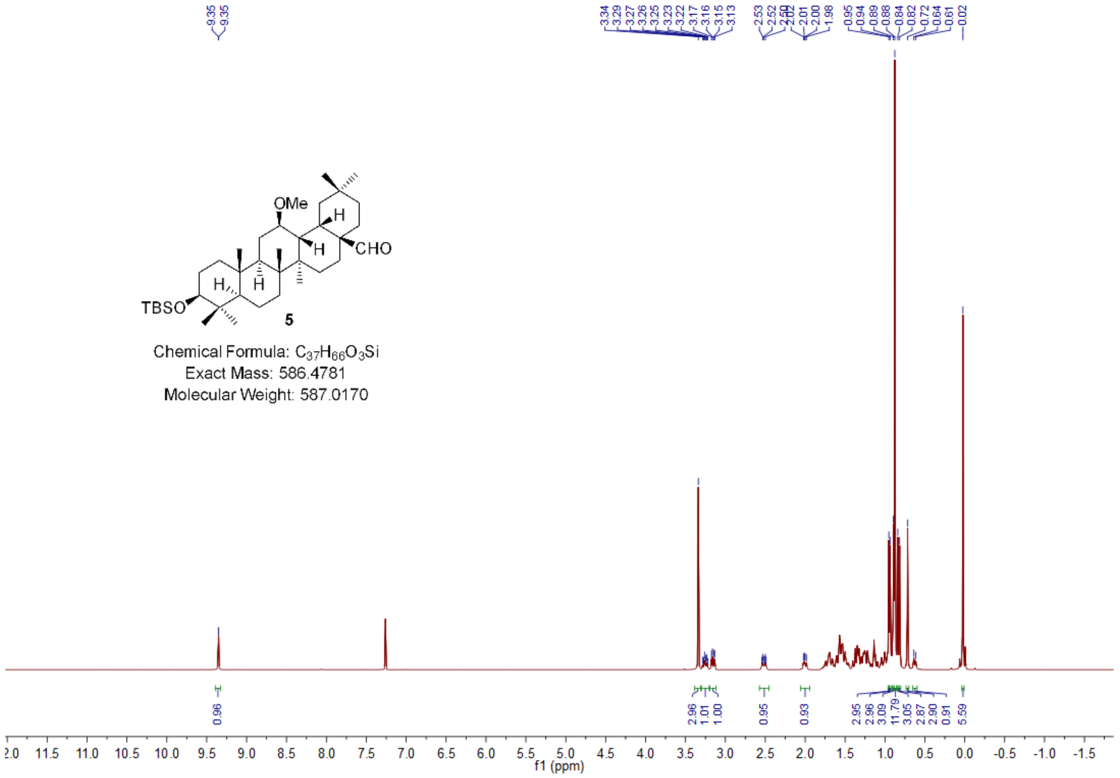

Supplementary Figure 10. ^{1}H-NMR spectrum of compound 5 (400 MHz, $CDCl_3$, 25 ^{o}C)

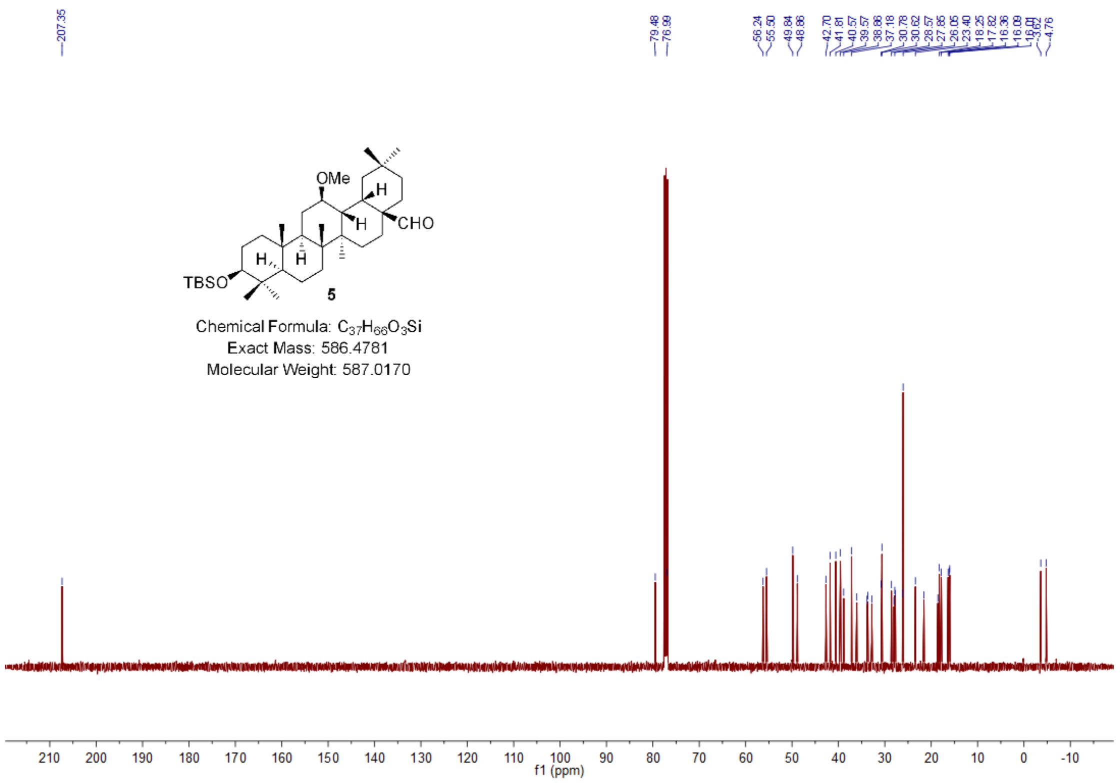

Supplementary Figure 11. ^{13}C-NMR spectrum of compound 5 (101 MHz, $CDCl_3$, 25 °C)

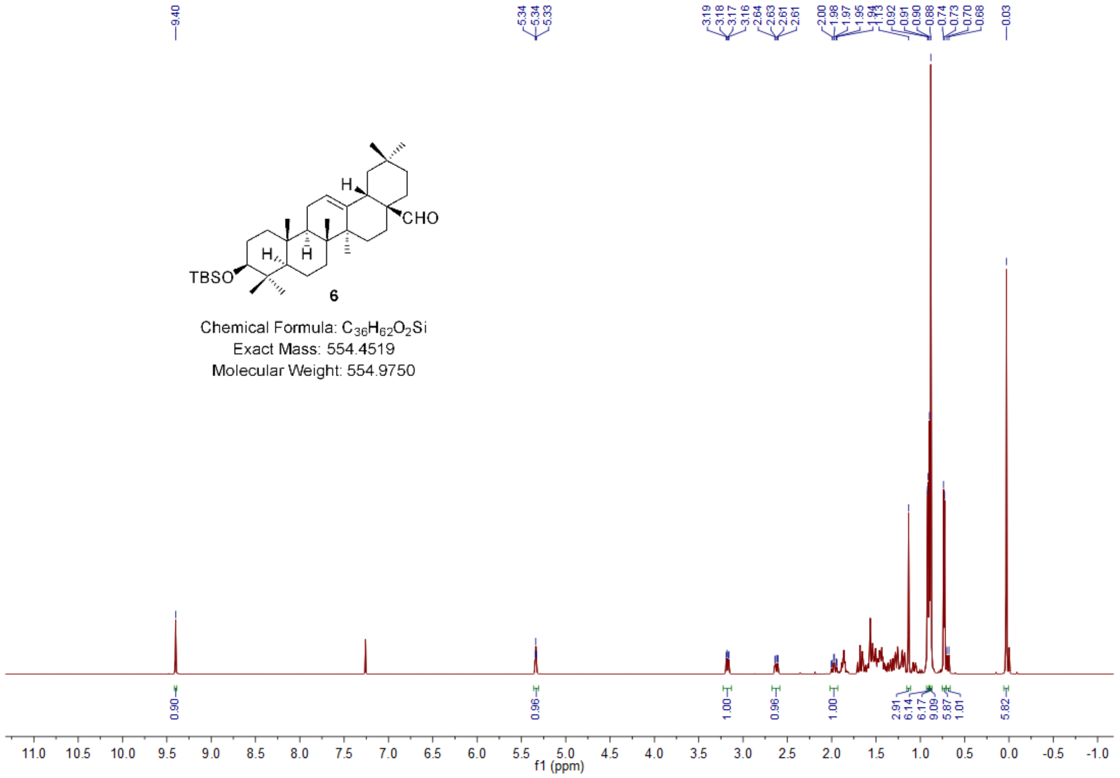

Supplementary Figure 12. ^{1}H-NMR spectrum of compound 6 (500 MHz, $CDCl_3$, 25 °C)

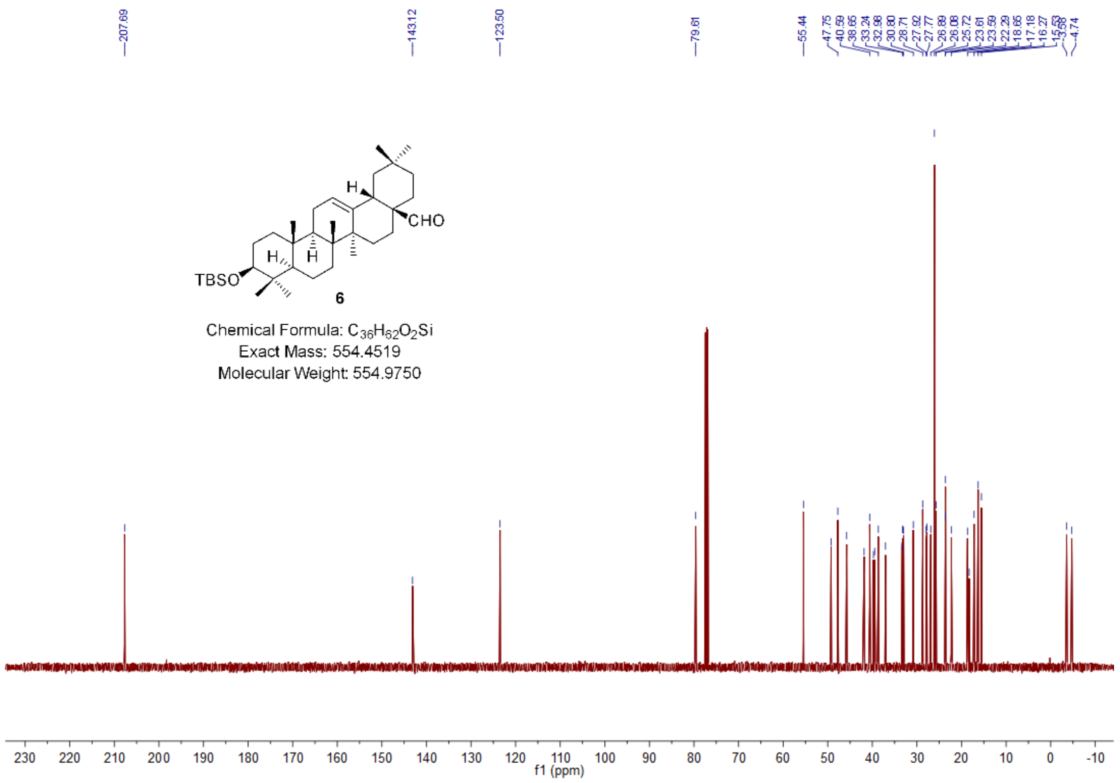

Supplementary Figure 13. ^{13}C-NMR spectrum of compound 6 (126 MHz, $CDCl_3$, 25 ^{o}C)

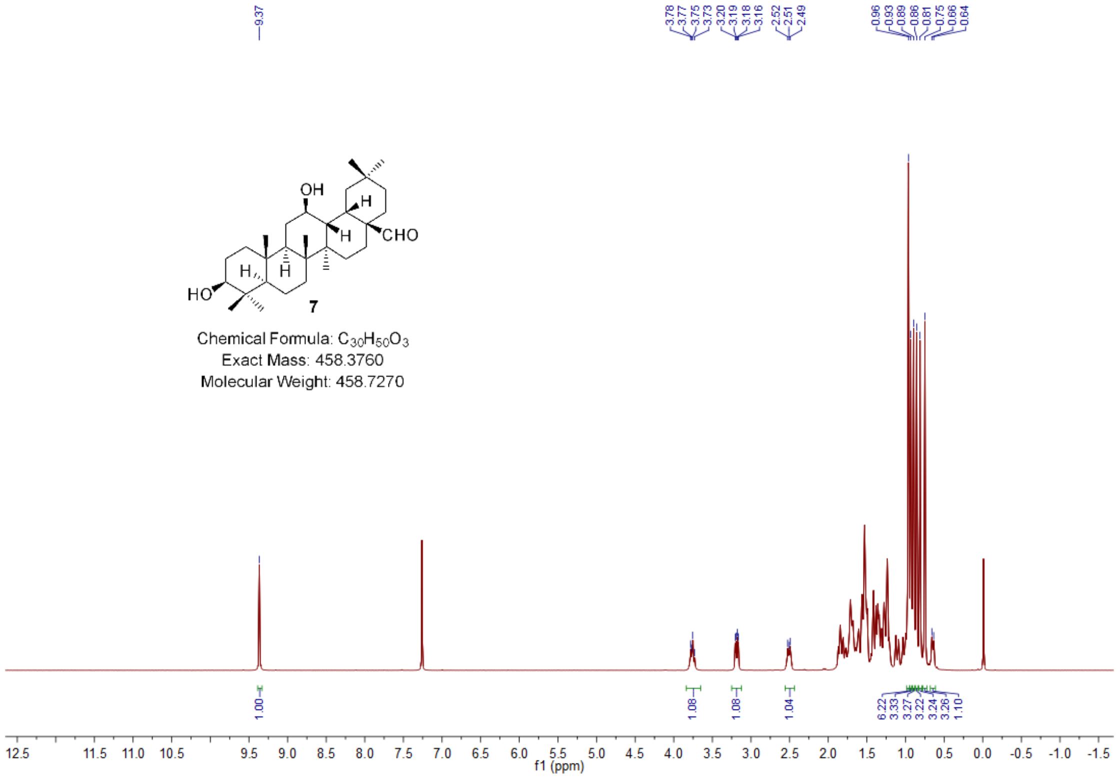

Supplementary Figure 14. ^{1}H-NMR spectrum of compound 7 (400 MHz, $CDCl_3$, 25 ^{o}C)

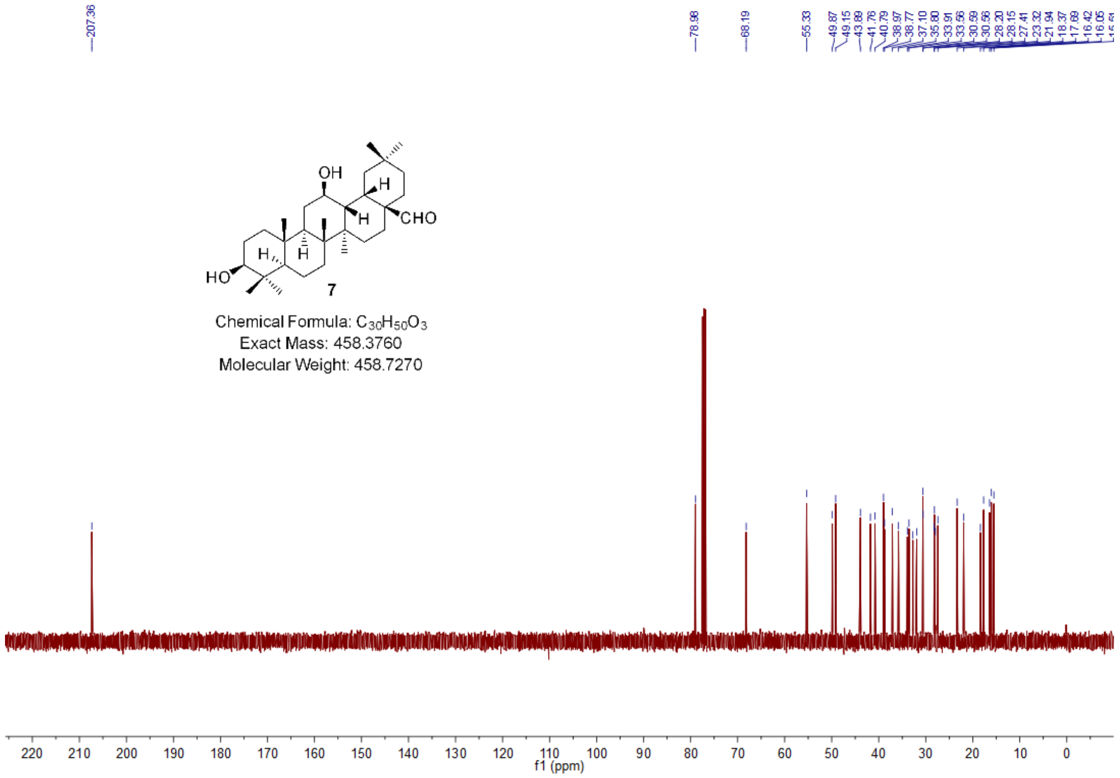

Supplementary Figure 15. ^{13}C-NMR spectrum of compound 7 (126 MHz, $CDCl_3$, 25 °C)

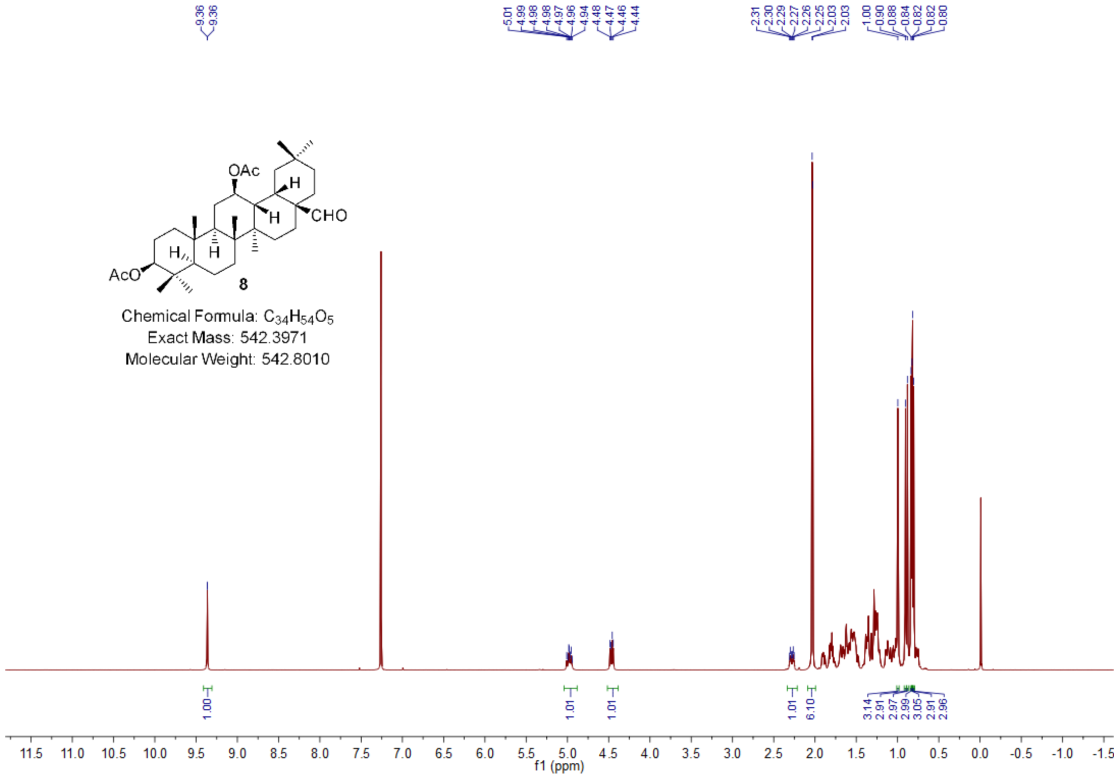

Supplementary Figure 16. ^{1}H-NMR spectrum of compound 8 (400 MHz, $CDCl_3$, 25 °C)

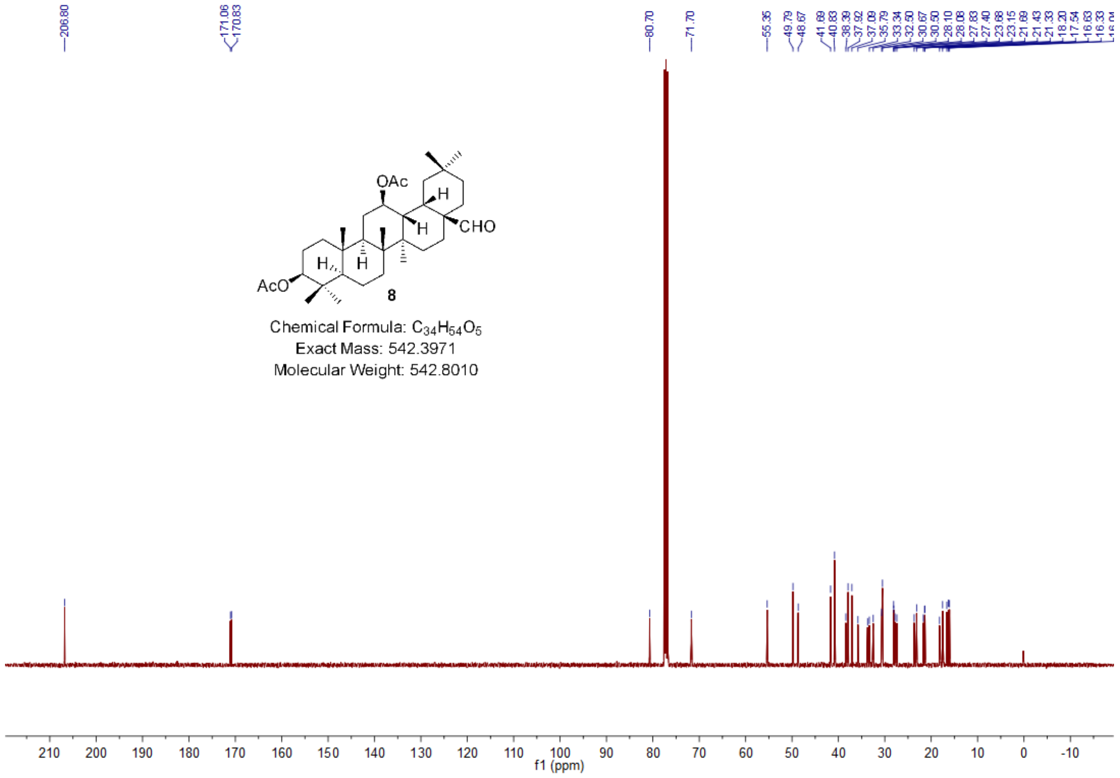

Supplementary Figure 17. ^{13}**C-NMR spectrum of compound 8 (101 MHz, $CDCl_3$, 25 °C)**

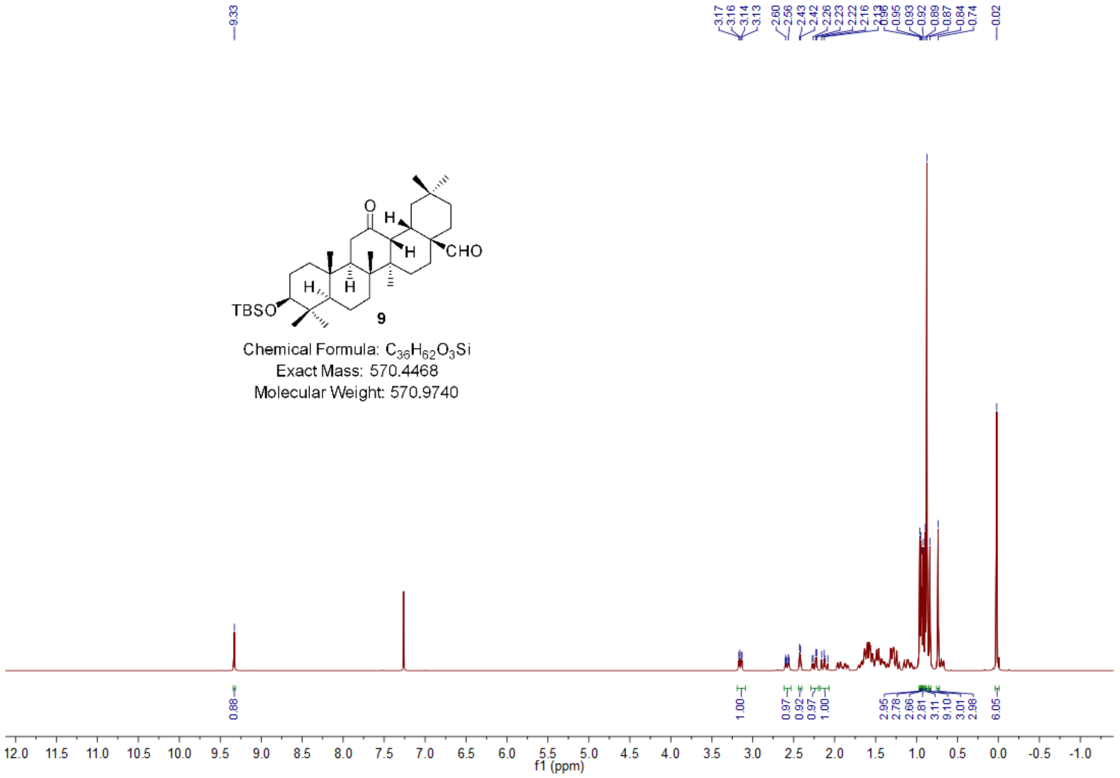

Supplementary Figure 18. ^{1}**H-NMR spectrum of compound 9 (400 MHz, $CDCl_3$, 25 °C)**

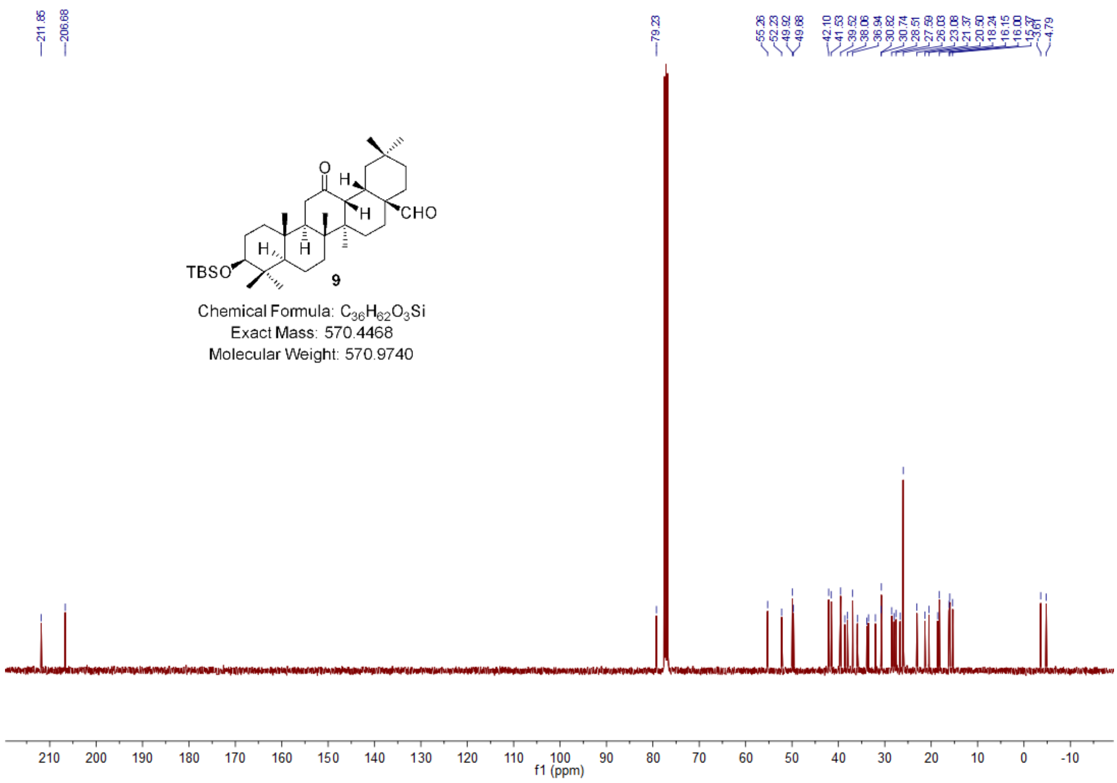

Supplementary Figure 19. ^{13}C-NMR spectrum of compound 9 (101 MHz, $CDCl_3$, 25 °C)

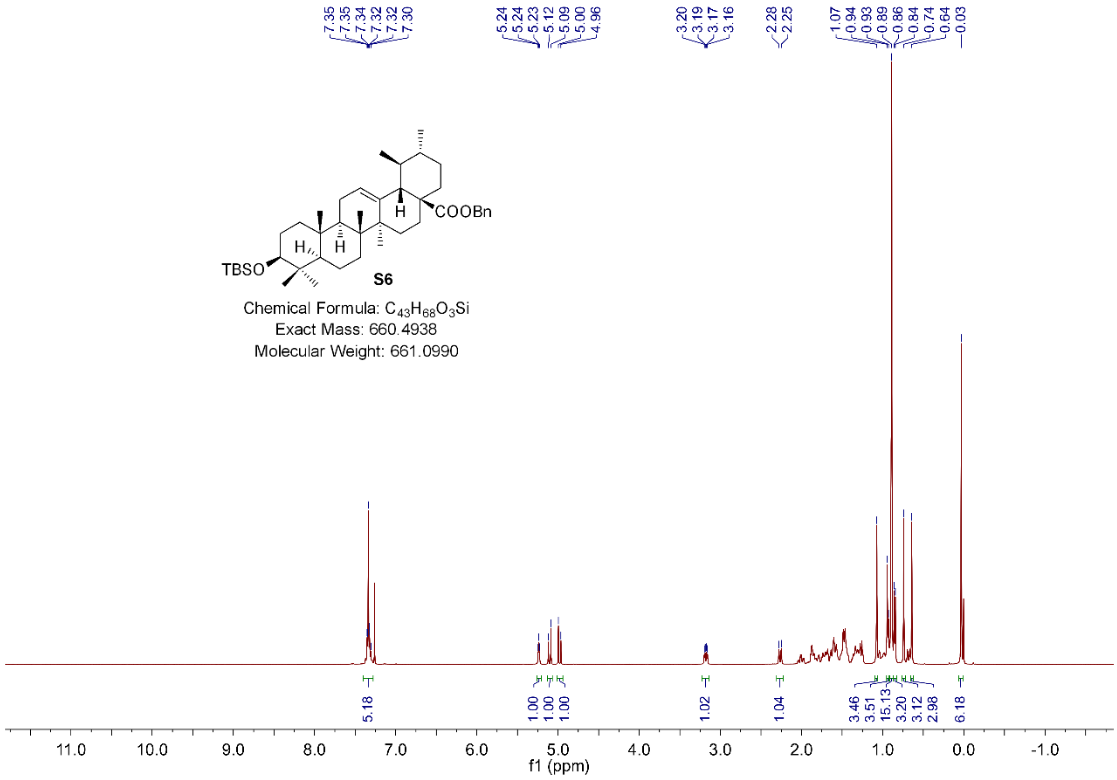

Supplementary Figure 20. ^{1}H-NMR spectrum of compound S6 (400 MHz, $CDCl_3$, 25 °C)

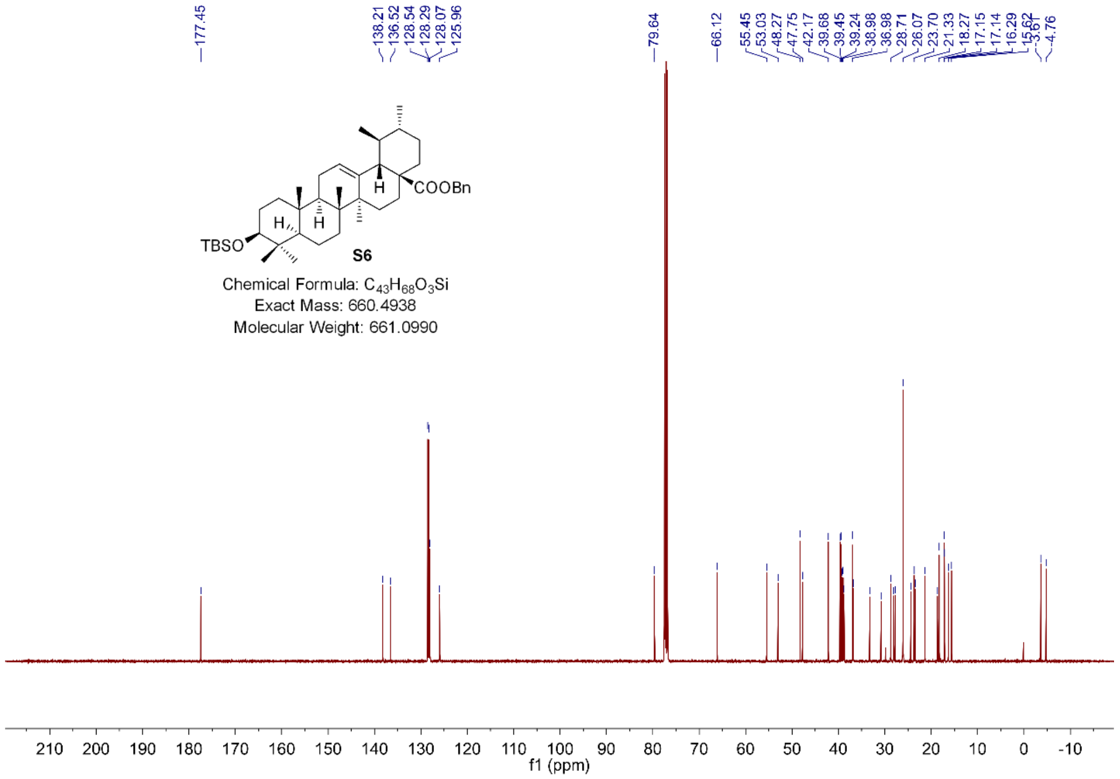

Supplementary Figure 21. ^{13}C-NMR spectrum of compound S6 (101 MHz, $CDCl_3$, 25 °C)

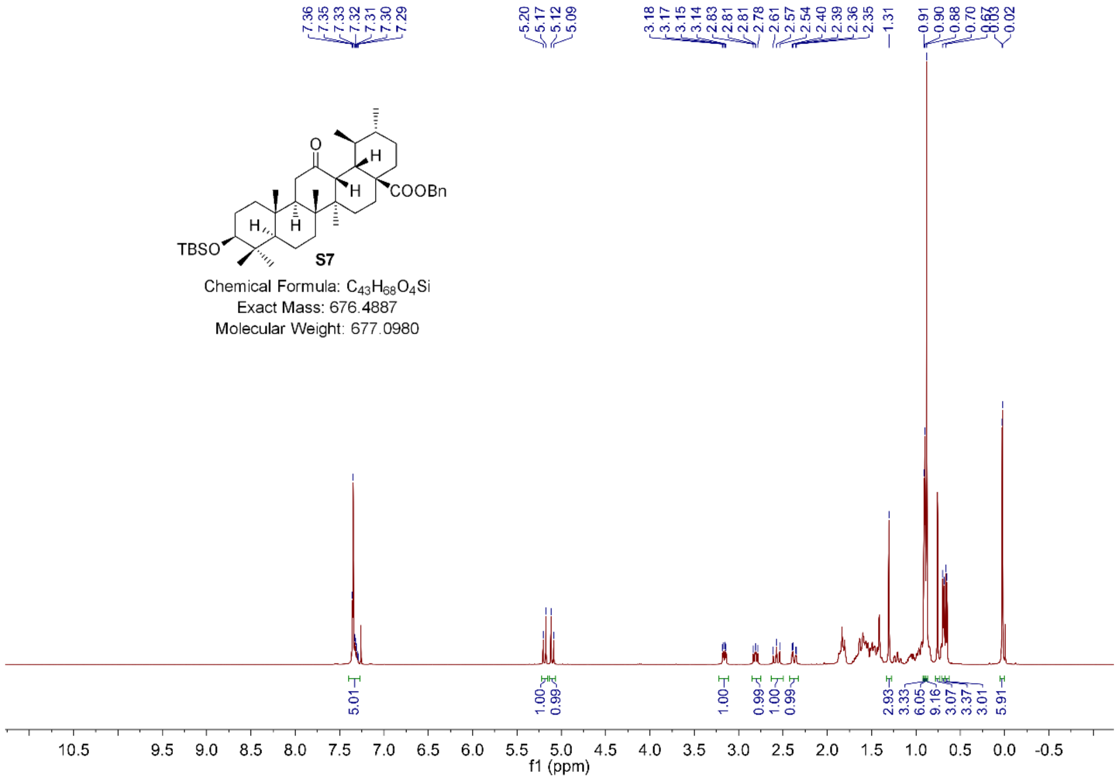

Supplementary Figure 22. ^{1}H-NMR spectrum of compound S7 (400 MHz, $CDCl_3$, 25 °C)

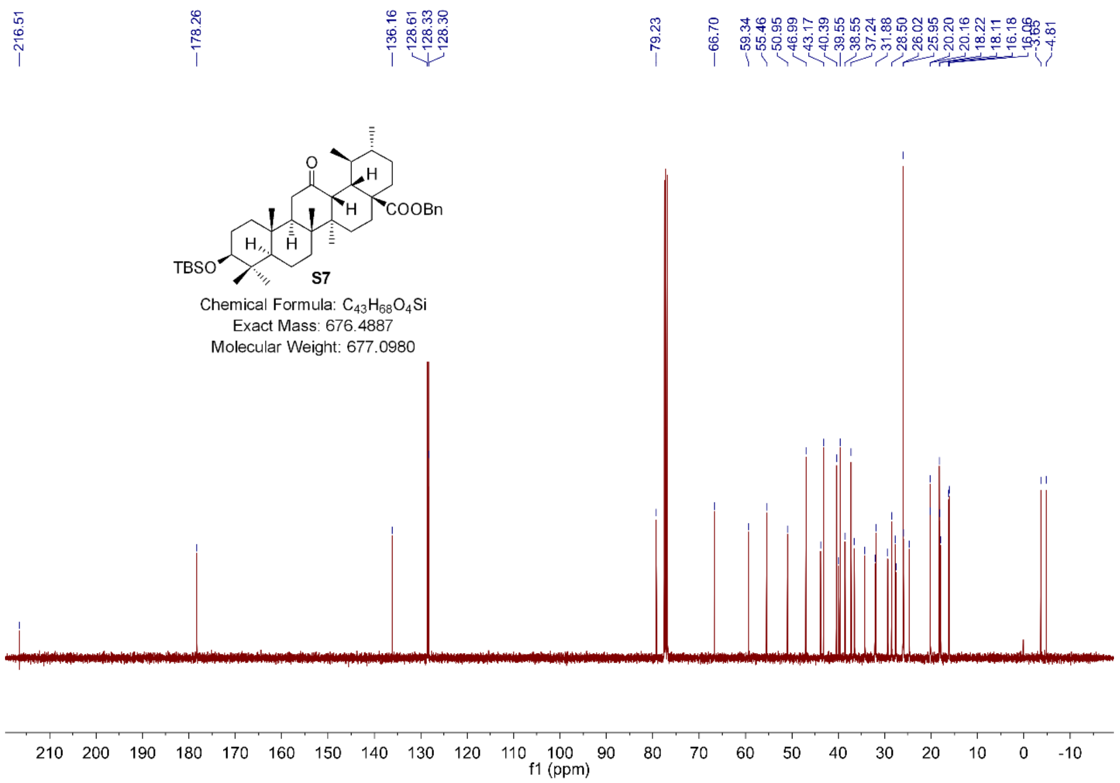

Supplementary Figure 23. ^{13}C-NMR spectrum of compound S7 (101 MHz, $CDCl_3$, 25 ºC)

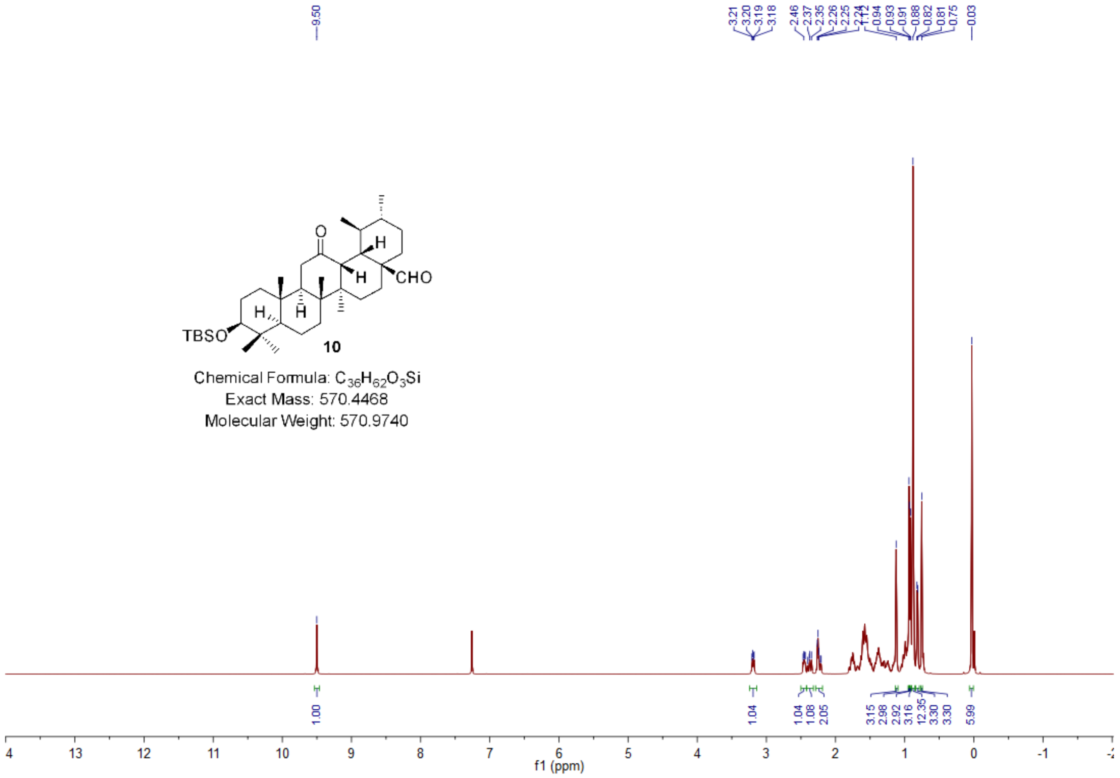

Supplementary Figure 24. ^{1}H-NMR spectrum of compound 10 (500 MHz, $CDCl_3$, 25 ºC)

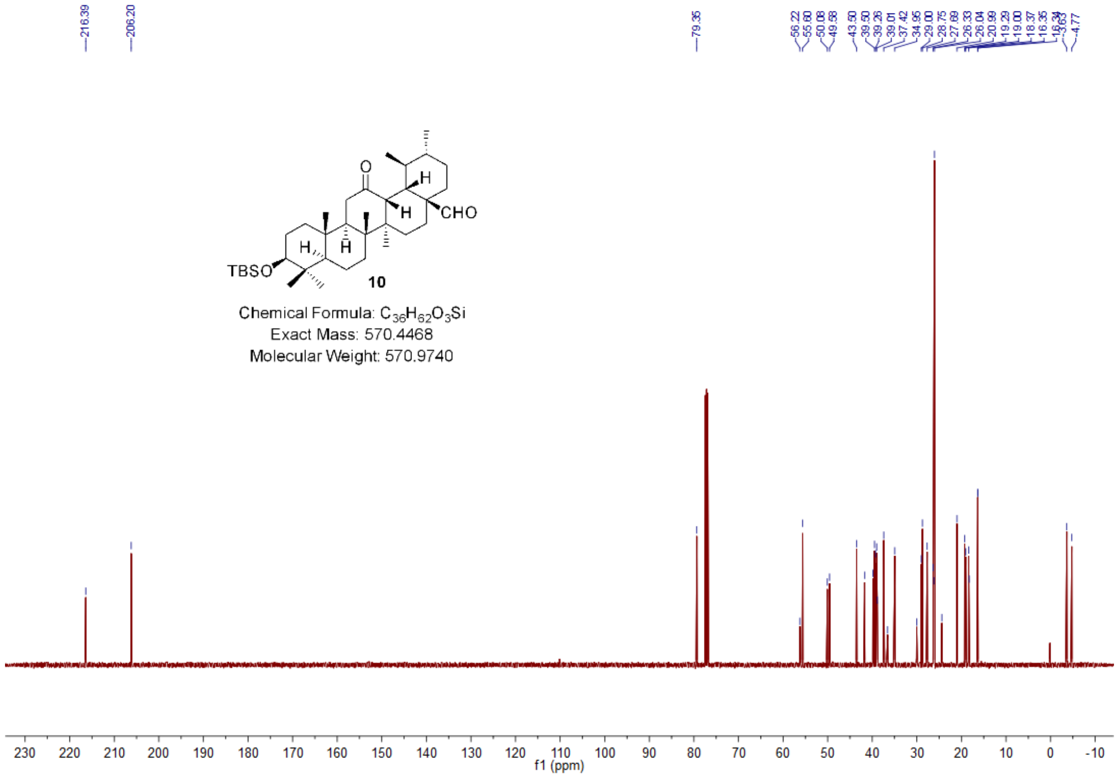

Supplementary Figure 25. ^{13}C-NMR spectrum of compound 10 (126 MHz, $CDCl_3$, 25 ºC)

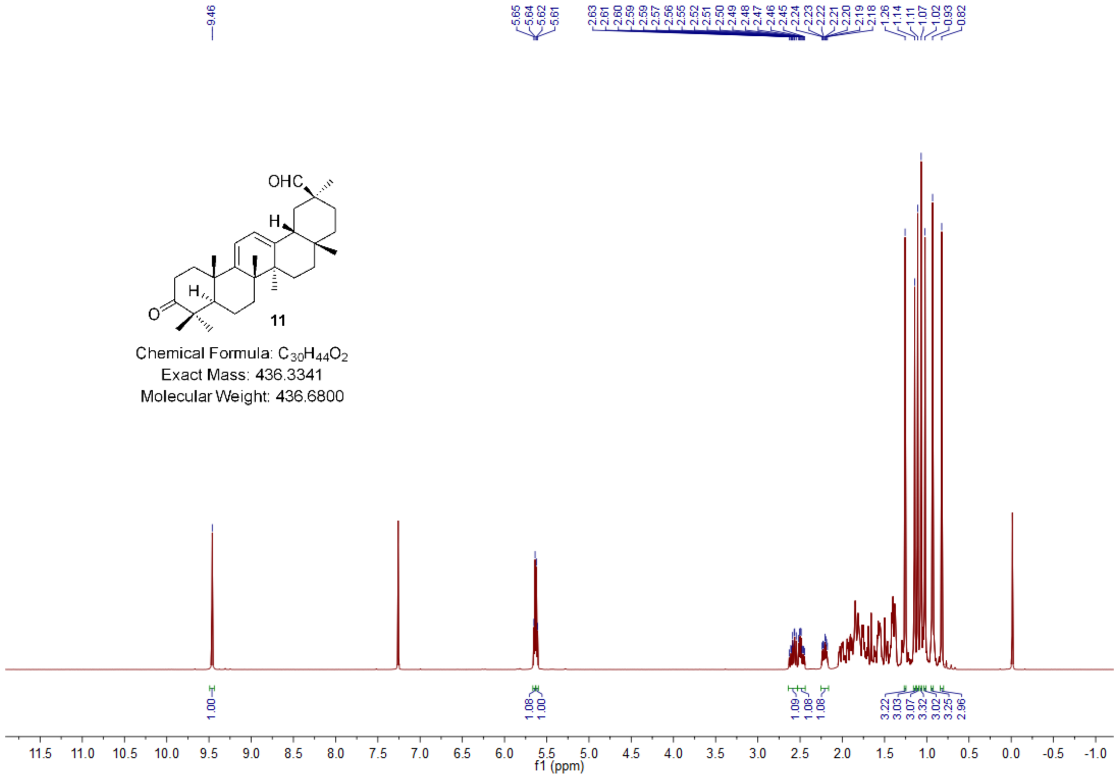

Supplementary Figure 26. ^{1}H-NMR spectrum of compound 11 (400 MHz, $CDCl_3$, 25 ºC)

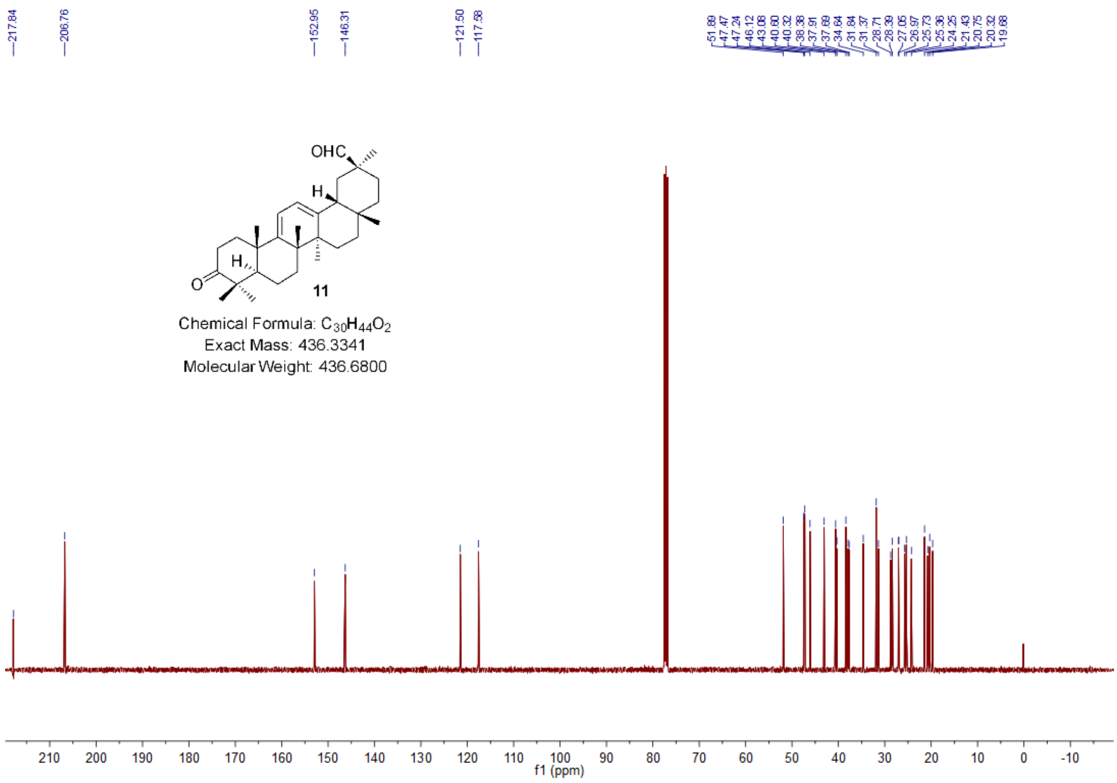

Supplementary Figure 27. ^{13}C-NMR spectrum of compound 11 (101 MHz, $CDCl_3$, 25 °C)

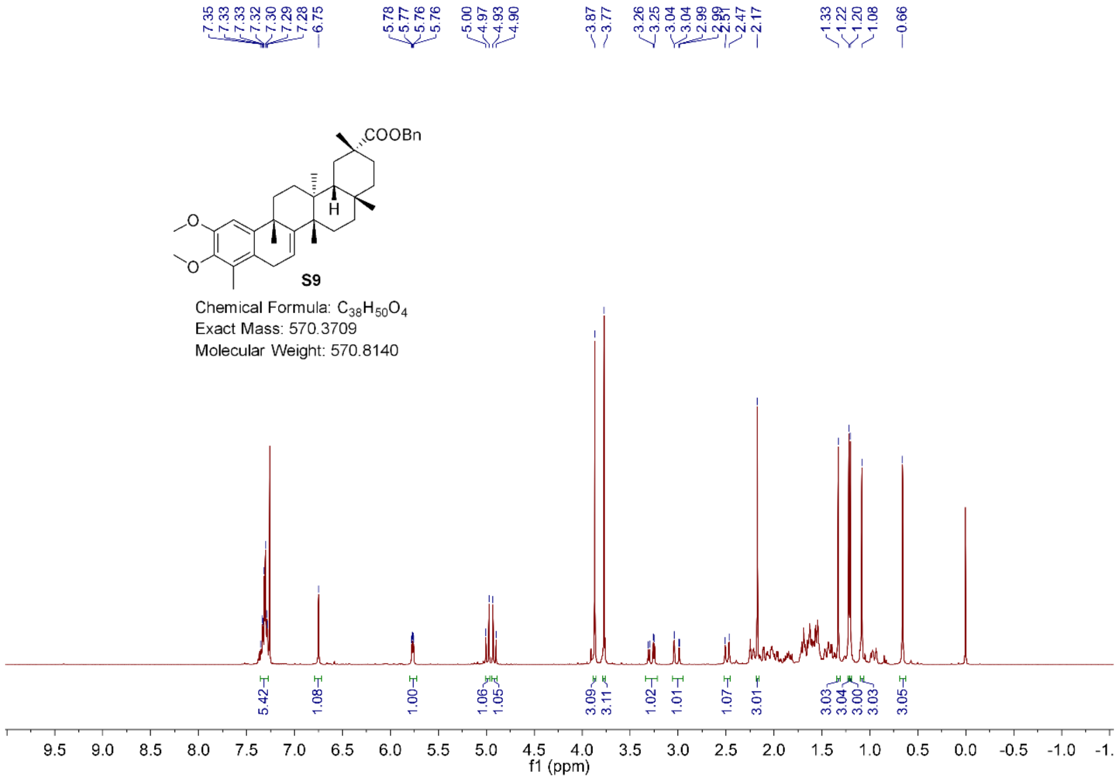

Supplementary Figure 28. ^{1}H-NMR spectrum of compound S9 (400 MHz, $CDCl_3$, 25 °C)

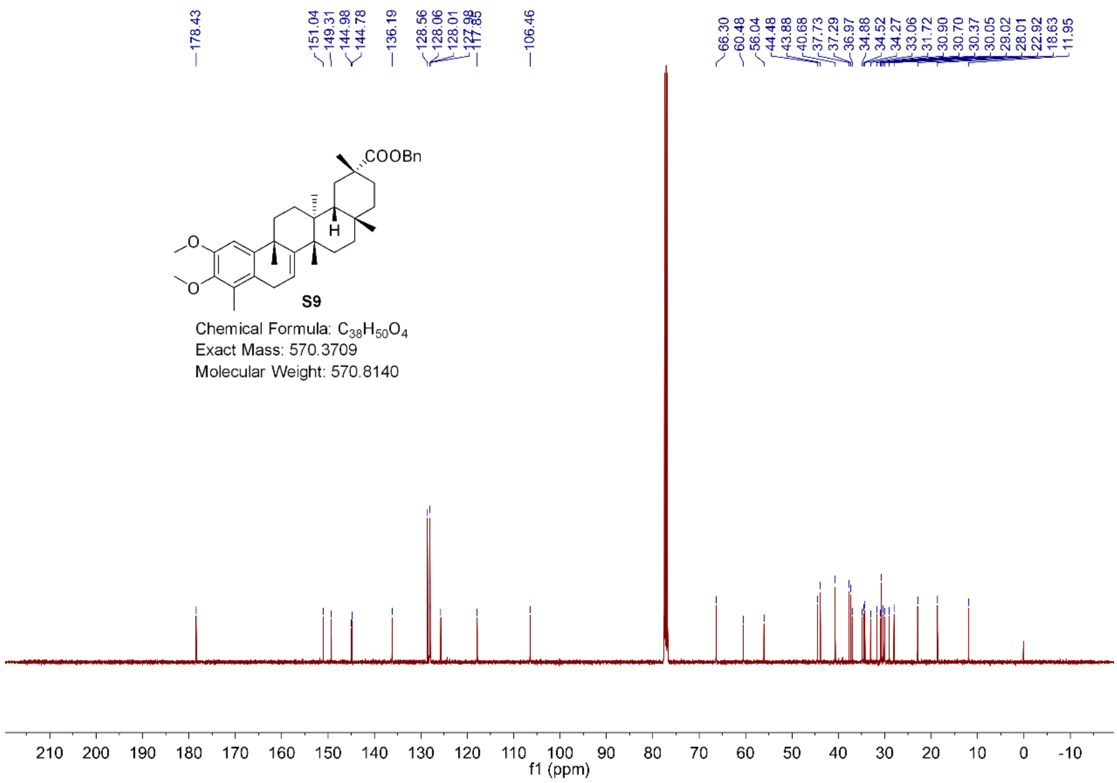

Supplementary Figure 29. ^{13}**C-NMR spectrum of compound S9 (101 MHz, $CDCl_3$, 25 °C)**

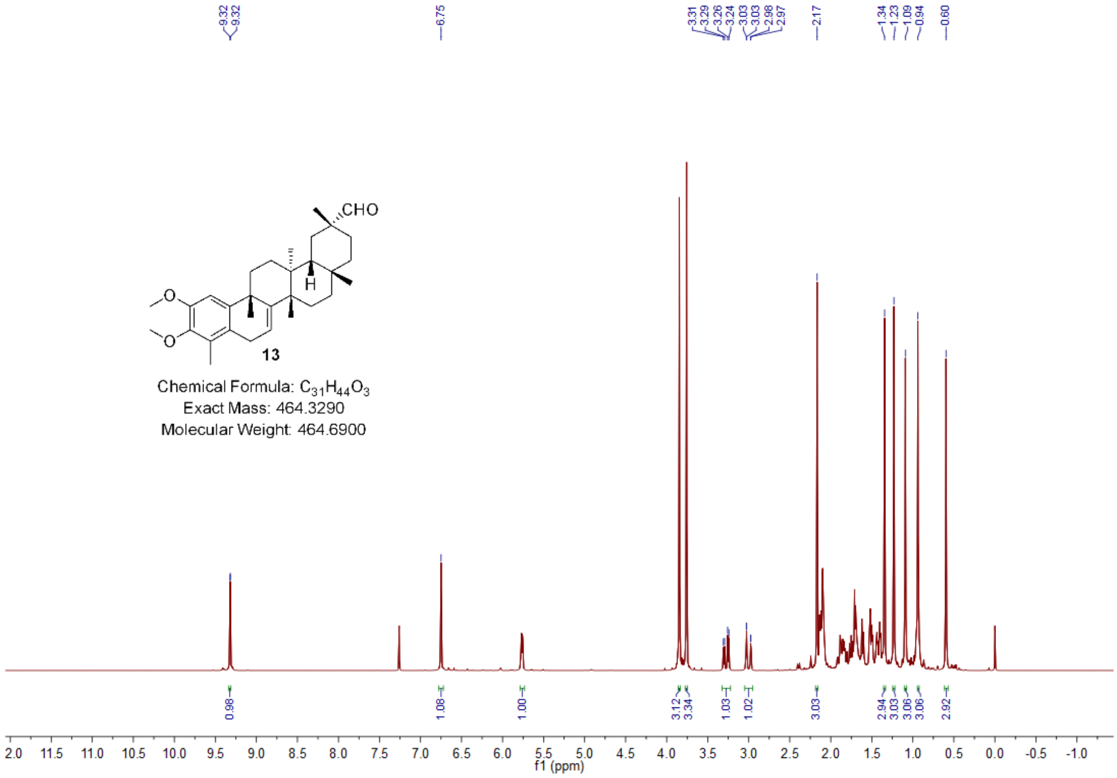

Supplementary Figure 30. ^{1}**H-NMR spectrum of compound 13 (400 MHz, $CDCl_3$, 25 °C)**

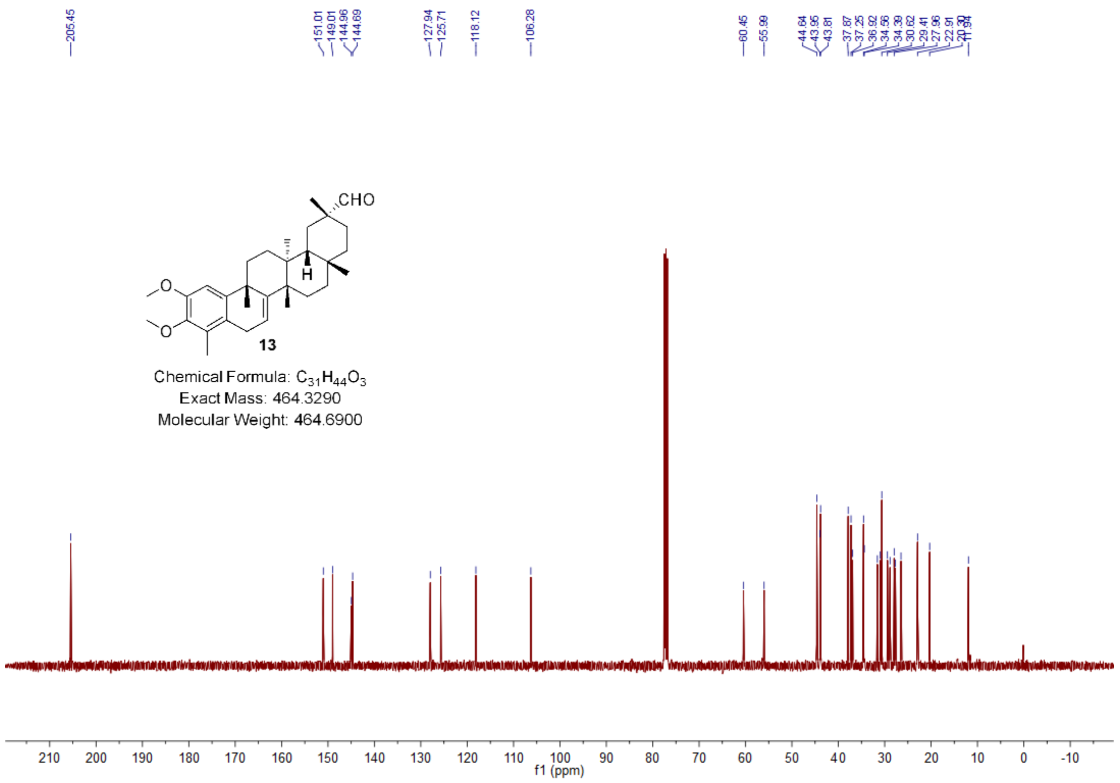

Supplementary Figure 31. **^{13}C-NMR spectrum of compound 13 (101 MHz, $CDCl_3$, 25 ºC)**

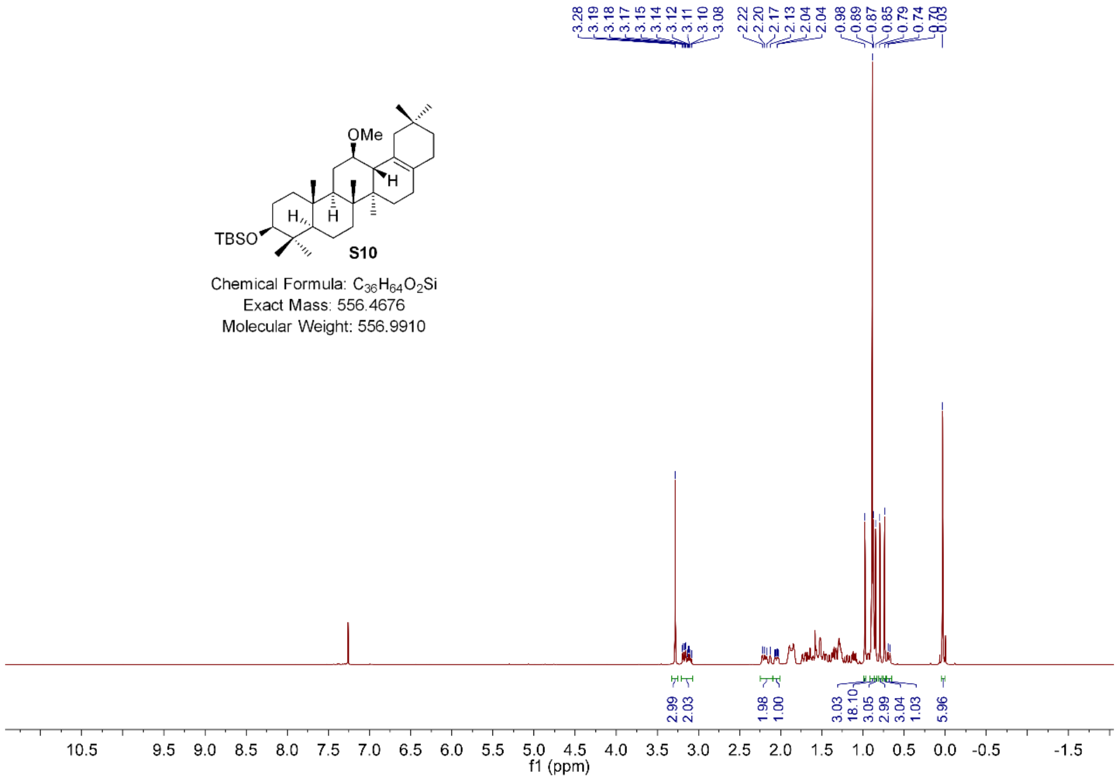

Supplementary Figure 32. **^{1}H-NMR spectrum of compound S10 (400 MHz, $CDCl_3$, 25 ºC)**

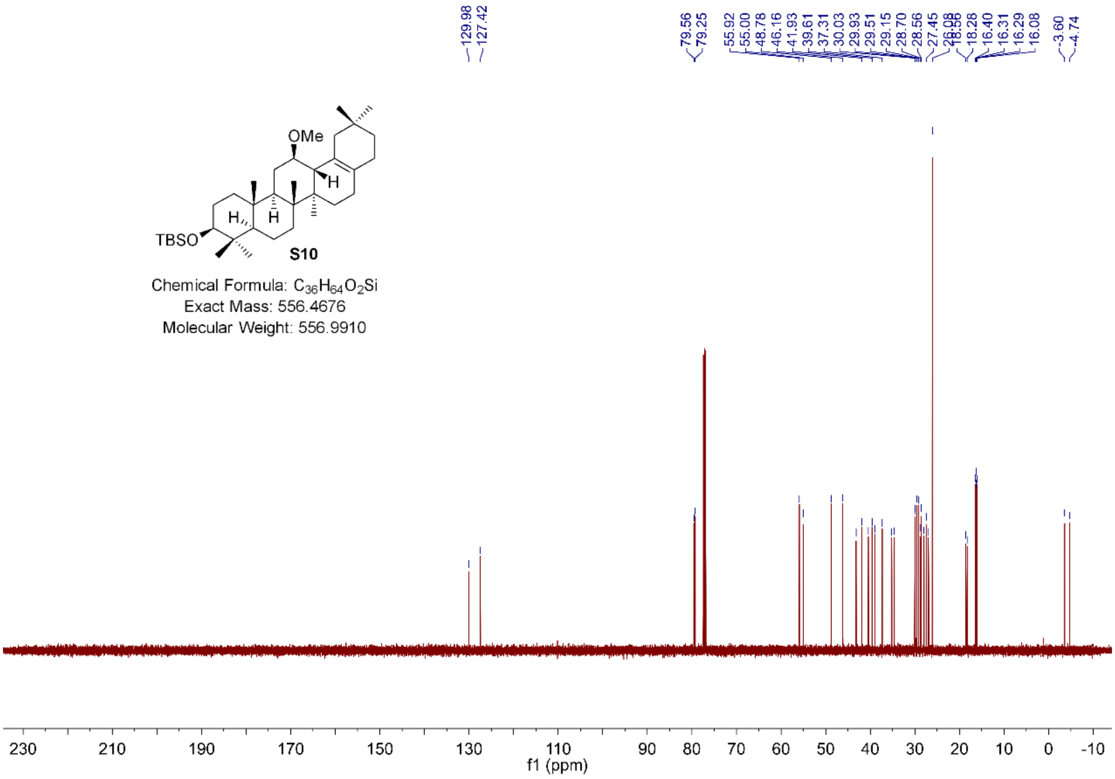

Supplementary Figure 33. ^{13}C-NMR spectrum of compound S10 (126 MHz, $CDCl_3$, 25 ºC)

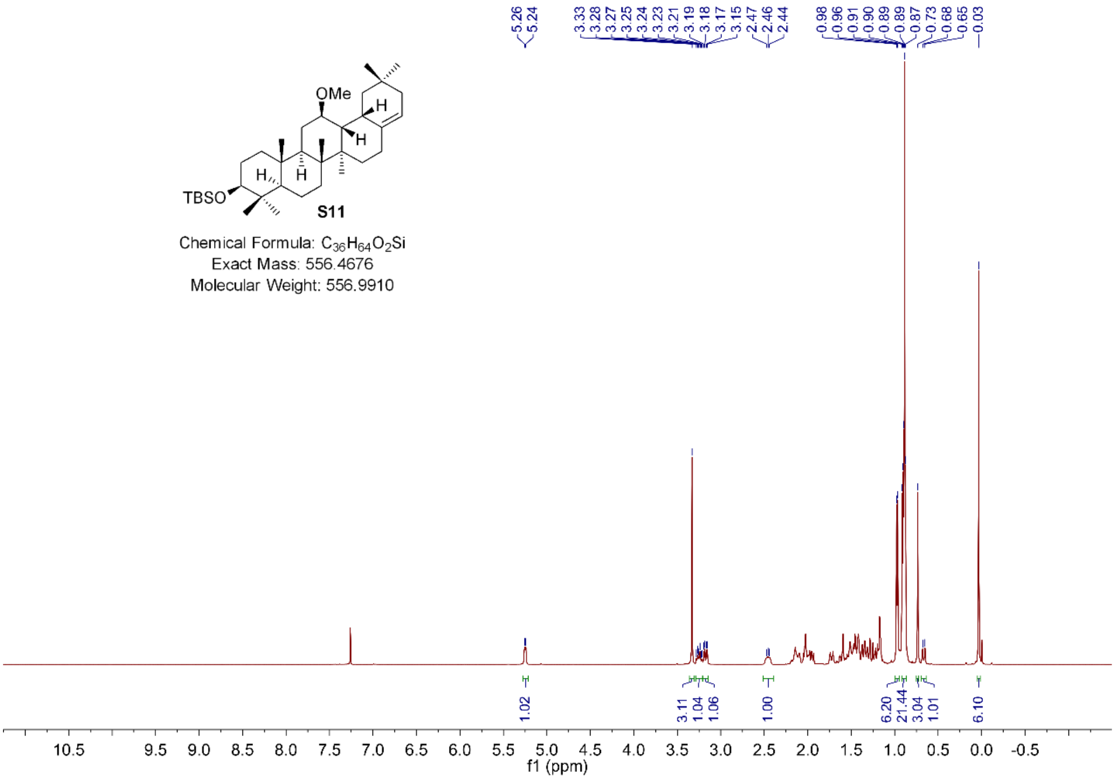

Supplementary Figure 34. ^{1}H-NMR spectrum of compound S11 (400 MHz, $CDCl_3$, 25 ºC)

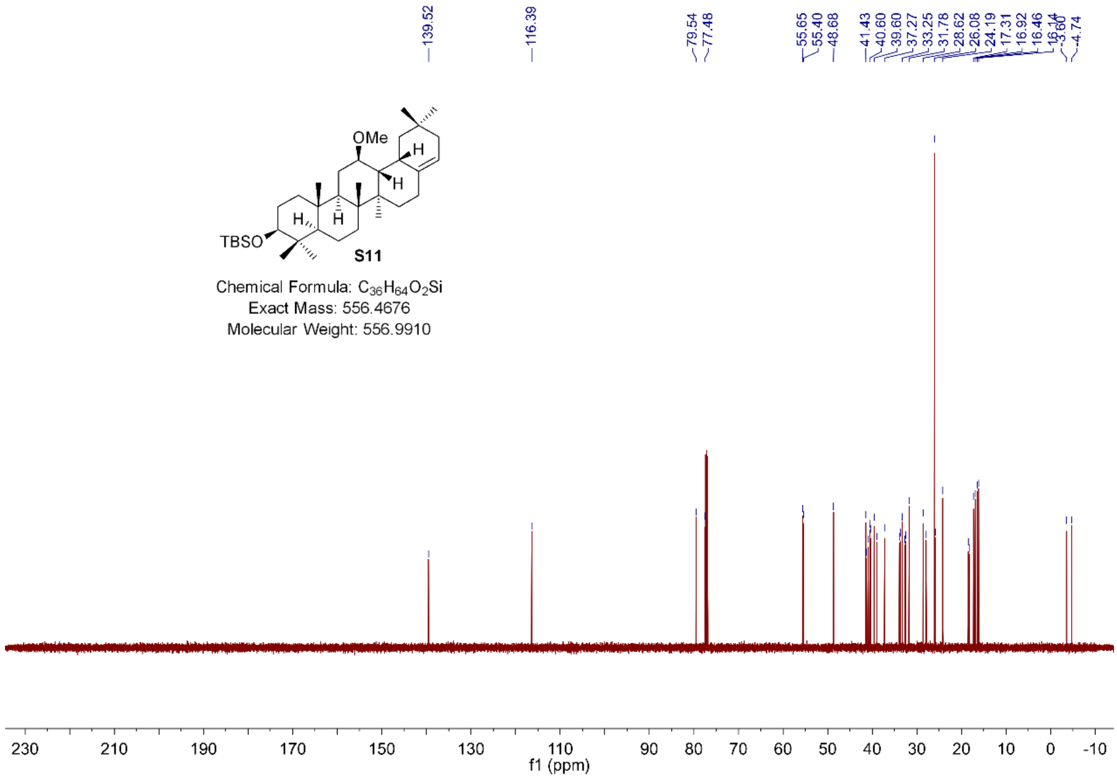

Supplementary Figure 35. ^{13}C-NMR spectrum of compound S11 (126 MHz, $CDCl_3$, 25 °C)

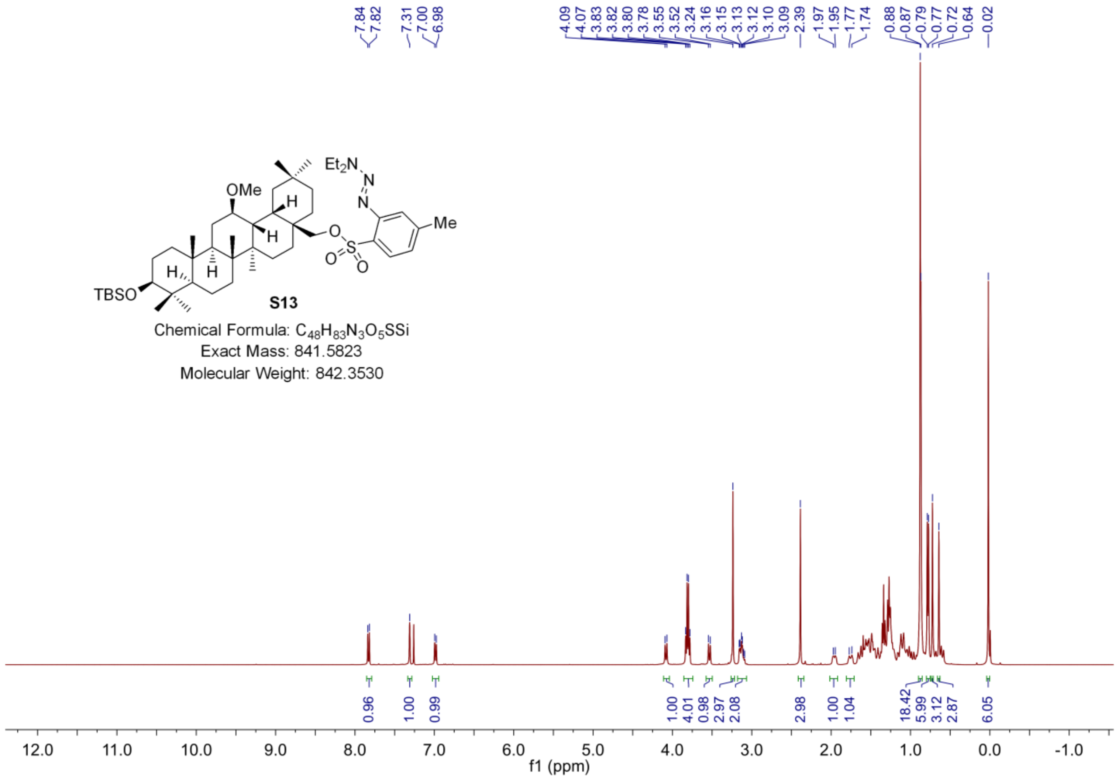

Supplementary Figure 36. ^{1}H-NMR spectrum of compound S13 (400 MHz, $CDCl_3$, 25 °C)

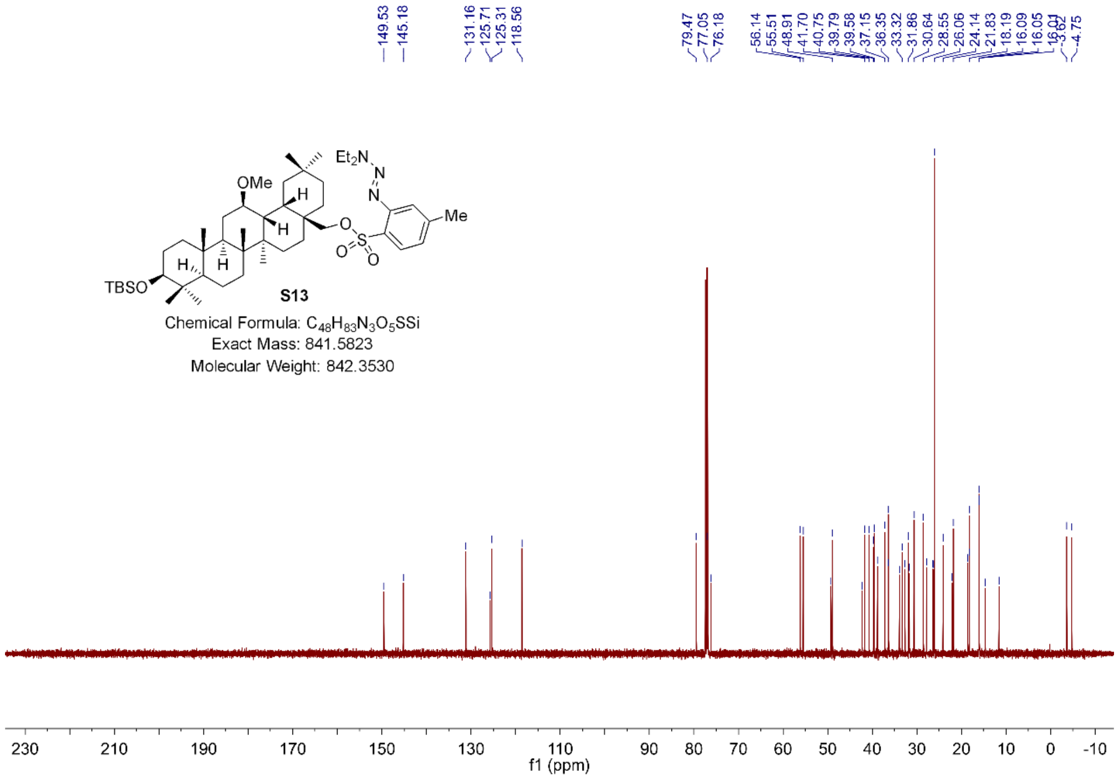

Supplementary Figure 37. ^{13}**C-NMR spectrum of compound S13 (126 MHz, CDCl$_3$, 25 °C)**

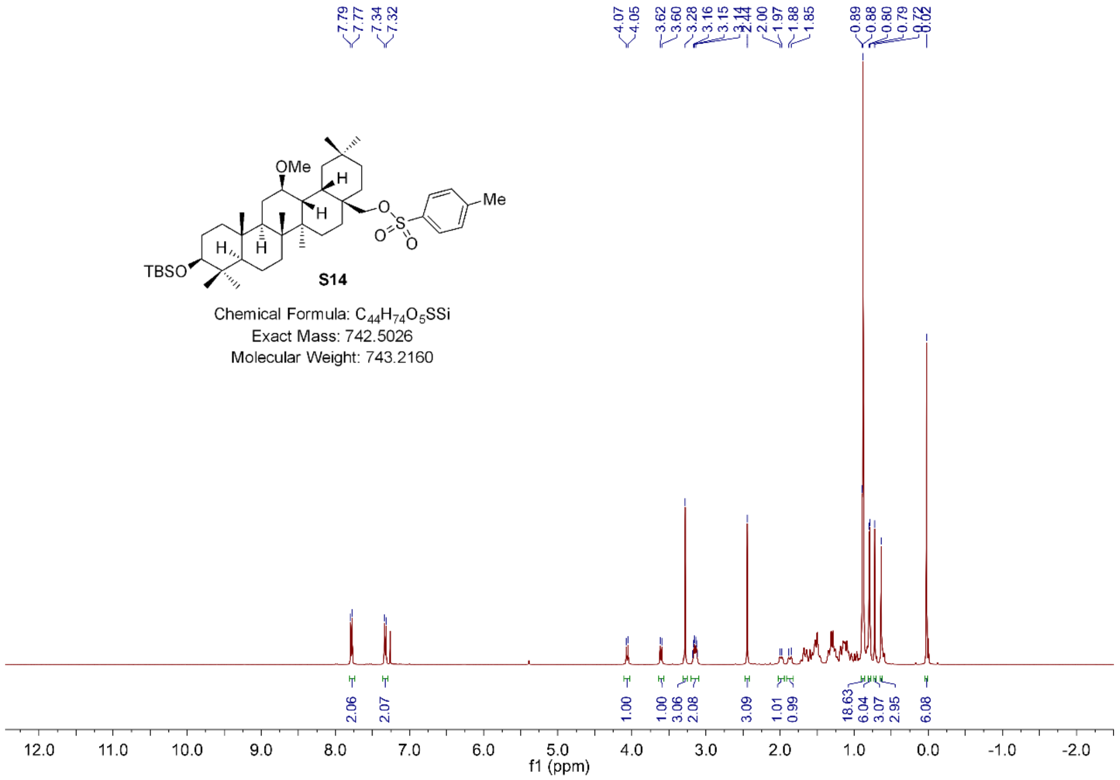

Supplementary Figure 38. ^{1}**H-NMR spectrum of compound S14 (400 MHz, CDCl$_3$, 25 °C)**

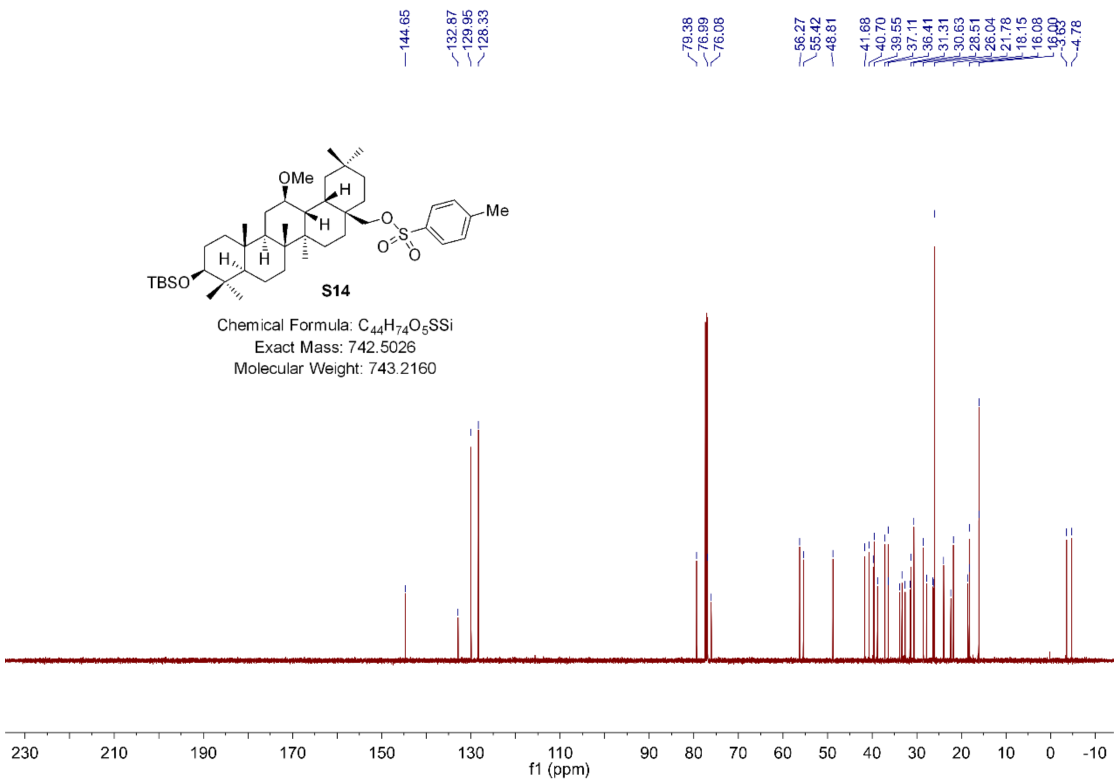

Supplementary Figure 39. ^{13}**C-NMR spectrum of compound S14 (126 MHz,** $CDCl_3$**, 25 °C)**

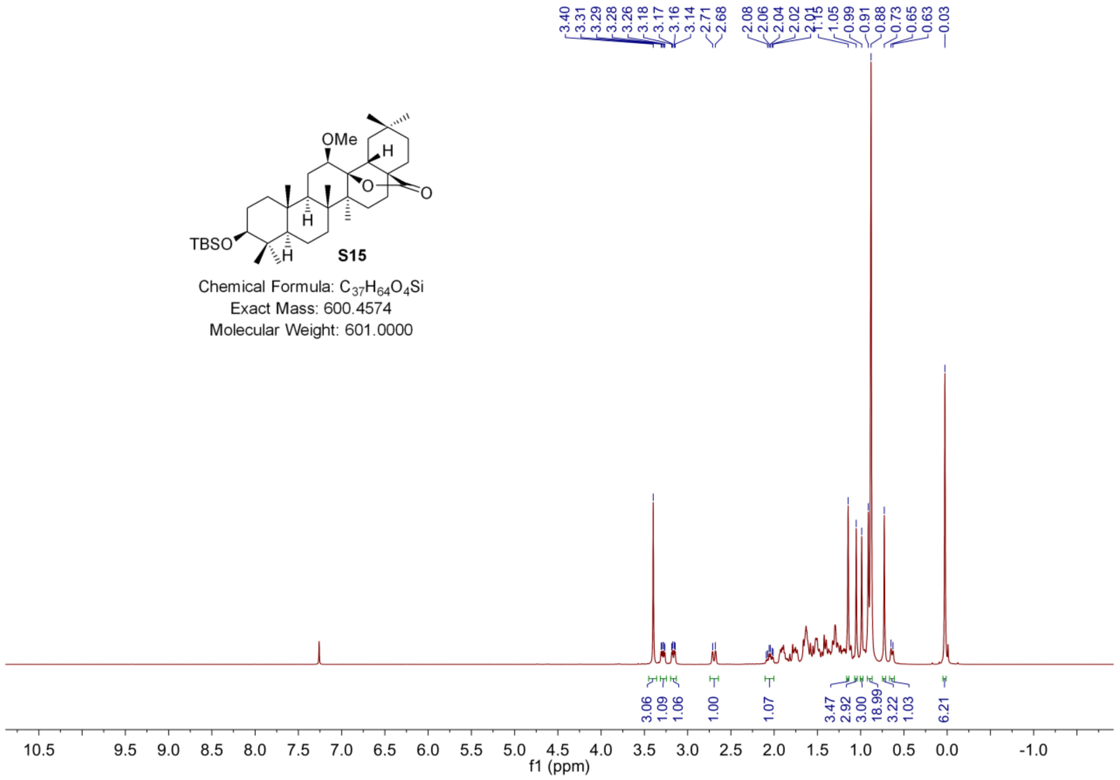

Supplementary Figure 40. ^{1}**H-NMR spectrum of compound S15 (400 MHz,** $CDCl_3$**, 25 °C)**

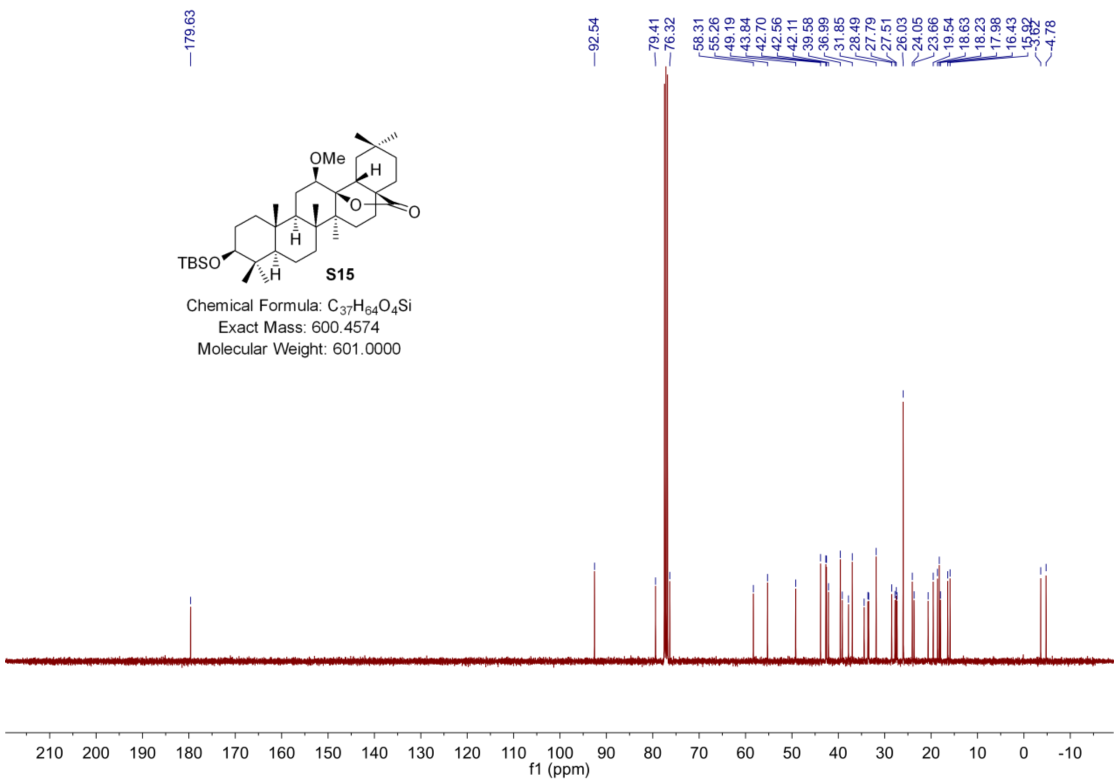

Supplementary Figure 41. ^{13}C-NMR spectrum of compound S15 (101 MHz, $CDCl_3$, 25 °C)

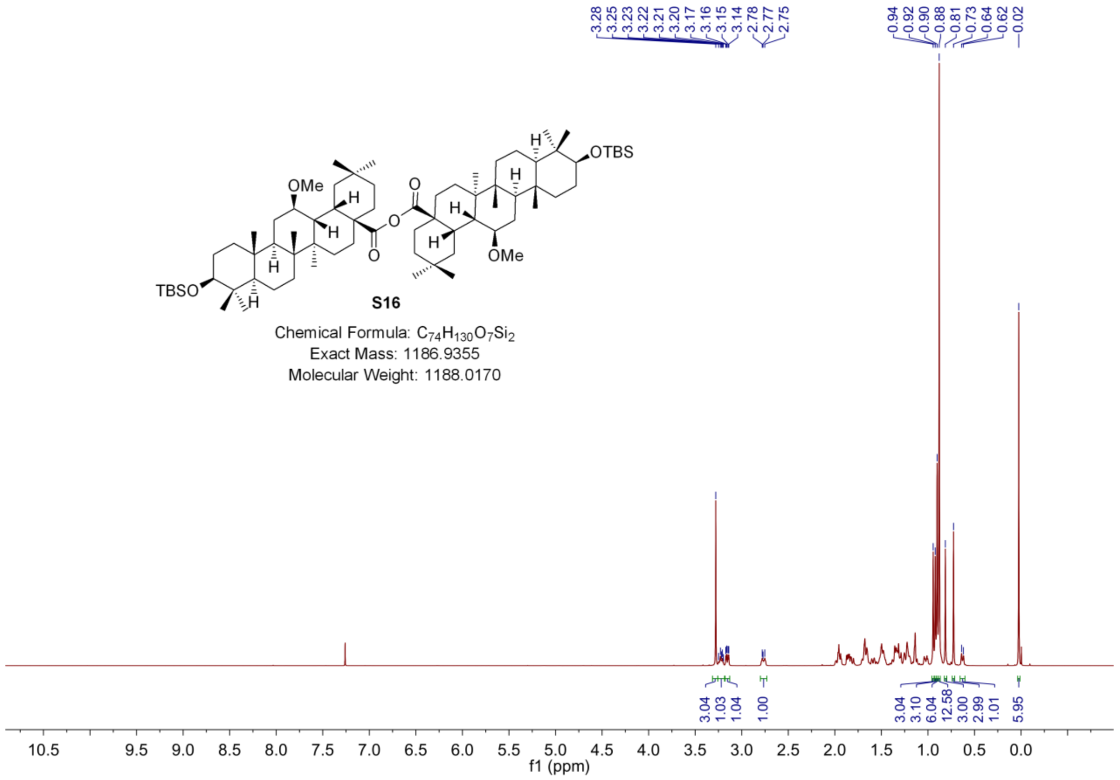

Supplementary Figure 42. ^{1}H-NMR spectrum of compound S16 (500 MHz, $CDCl_3$, 25 °C)

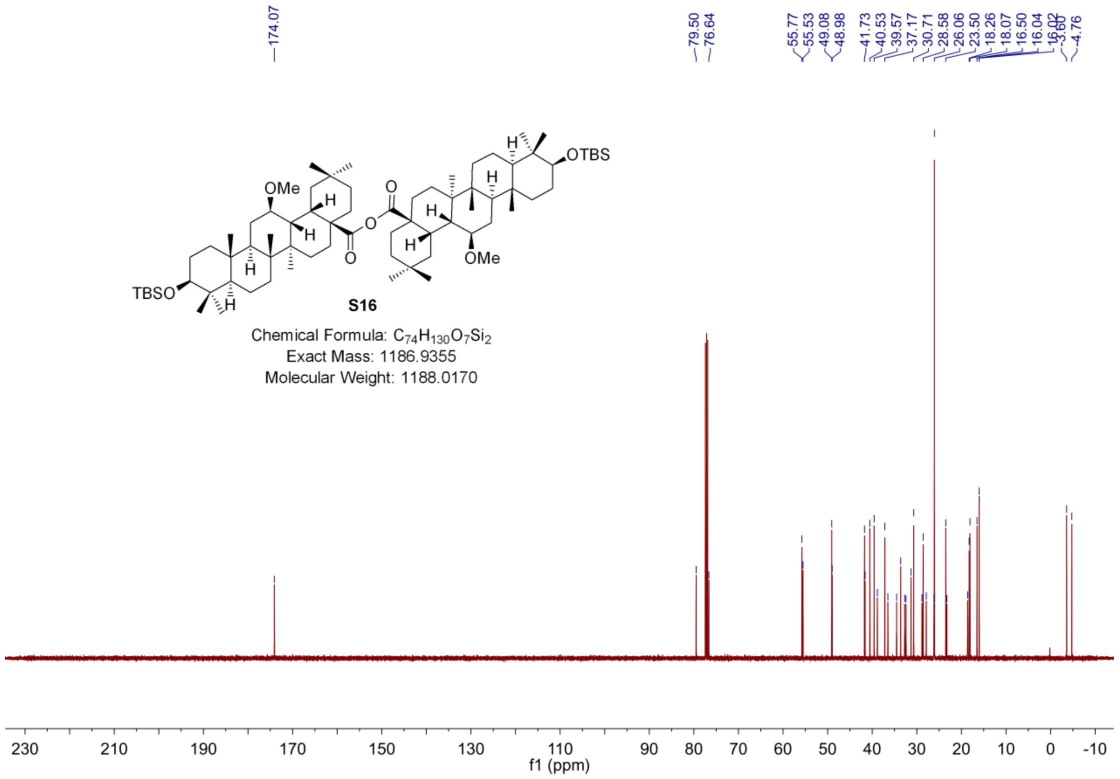

Supplementary Figure 43. **^{13}C-NMR spectrum of compound S16 (126 MHz, $CDCl_3$, 25 ^{o}C)**

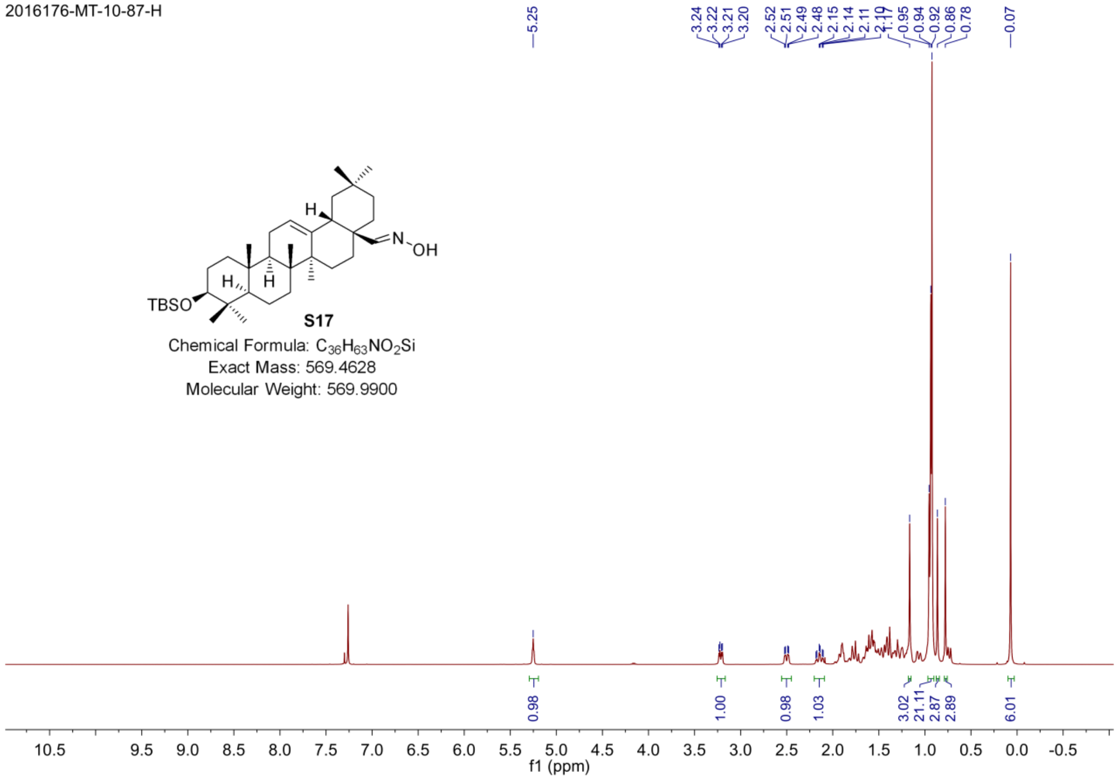

Supplementary Figure 44. **^{1}H-NMR spectrum of compound S17 (400 MHz, $CDCl_3$, 25 ^{o}C)**

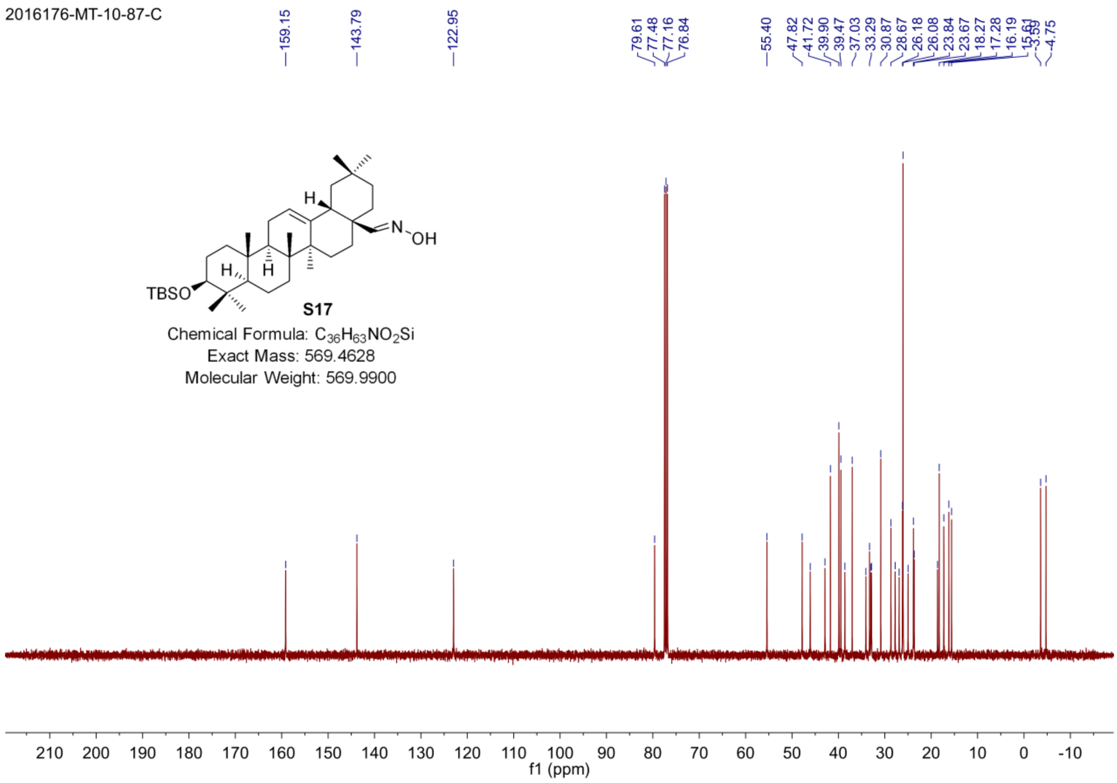

Supplementary Figure 45. ^{13}C-NMR spectrum of compound S17 (101 MHz, $CDCl_3$, 25 ^{o}C)

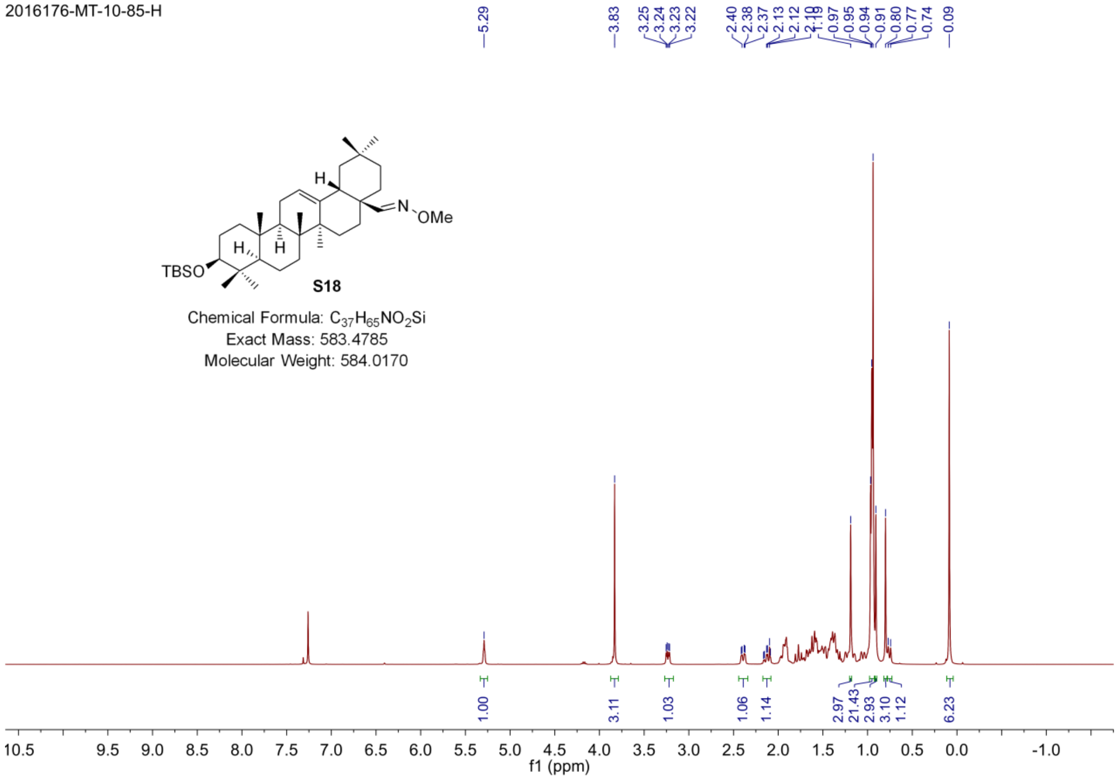

Supplementary Figure 46. ^{1}H-NMR spectrum of compound S18 (400 MHz, $CDCl_3$, 25 ^{o}C)

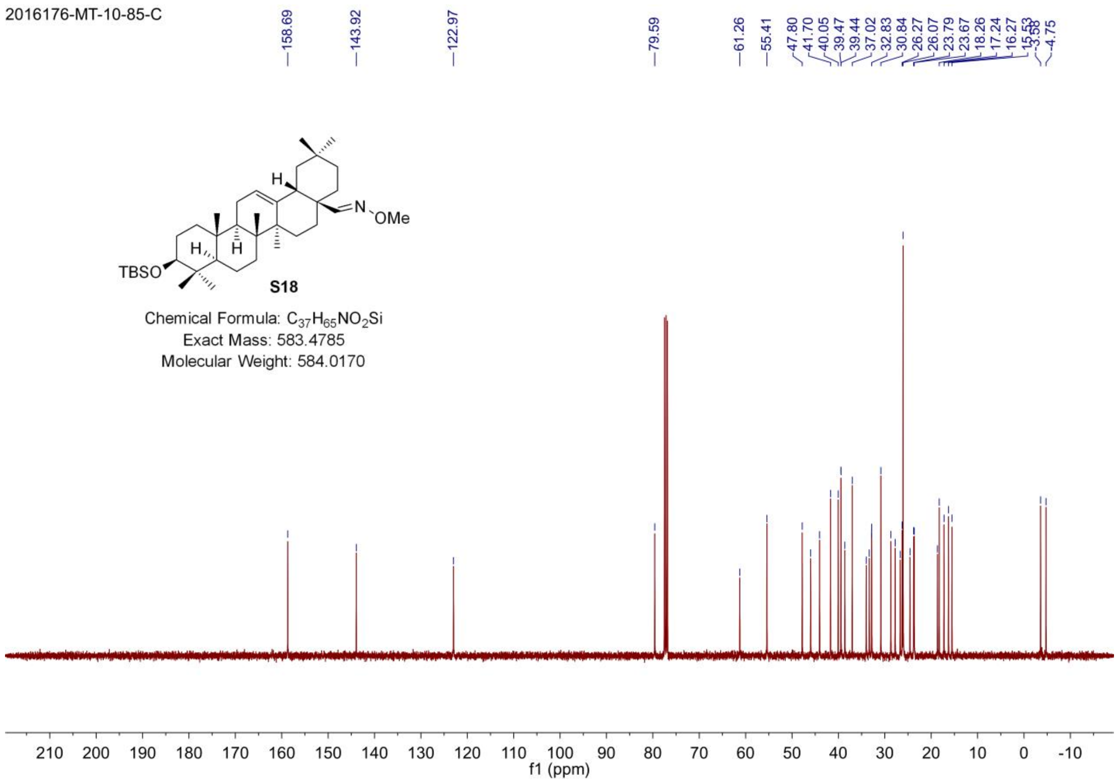

Supplementary Figure 47. ^{13}C-NMR spectrum of compound S18 (101 MHz, $CDCl_3$, 25 ^{o}C)

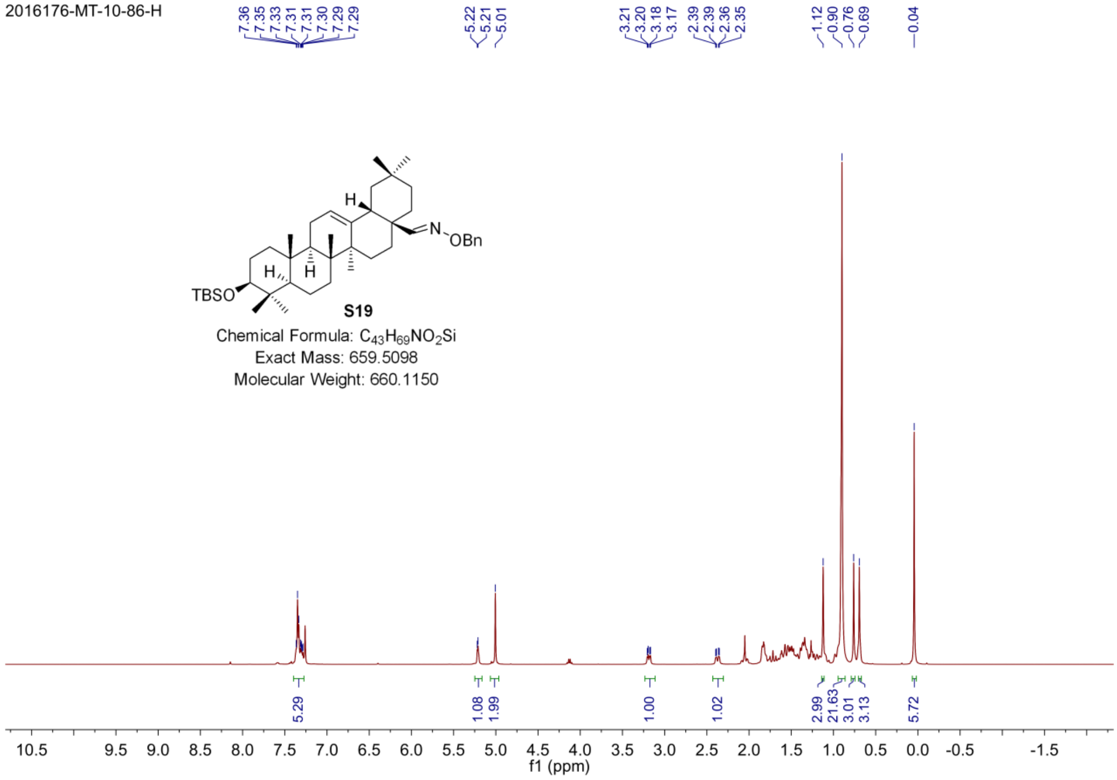

Supplementary Figure 48. ^{1}H-NMR spectrum of compound S19 (400 MHz, $CDCl_3$, 25 ^{o}C)

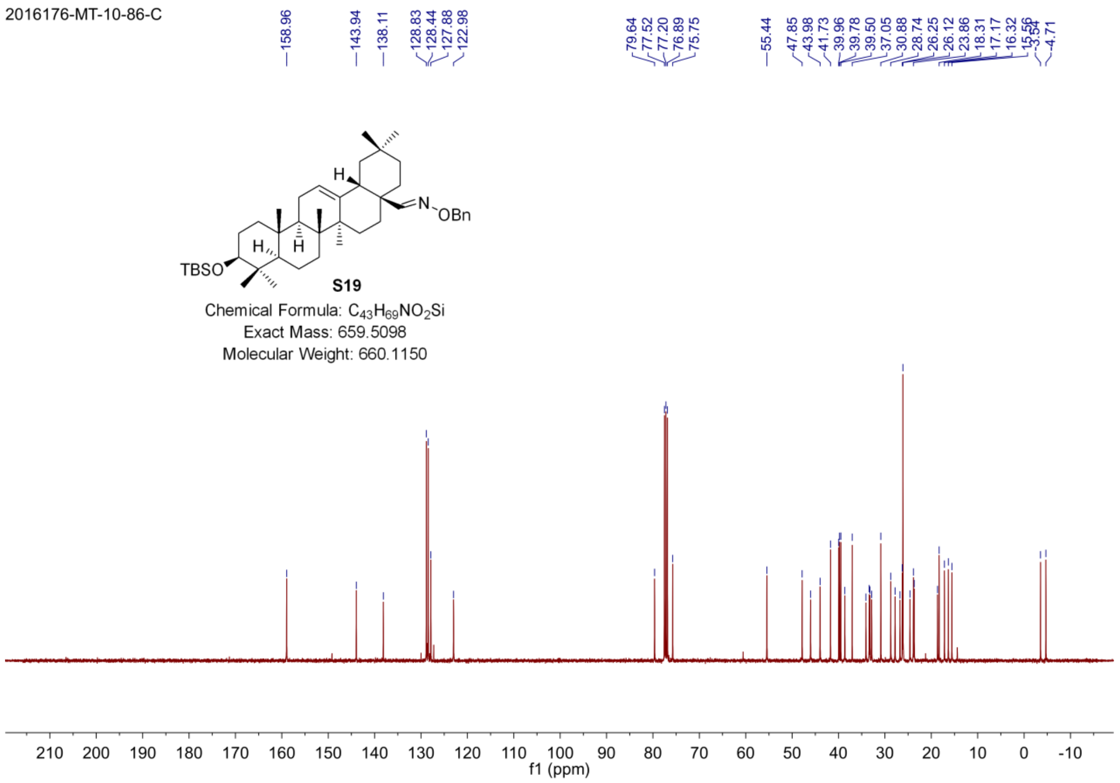

Supplementary Figure 49. ^{13}**C-NMR spectrum of compound S19 (101 MHz, CDCl$_3$, 25 ^{o}C)**

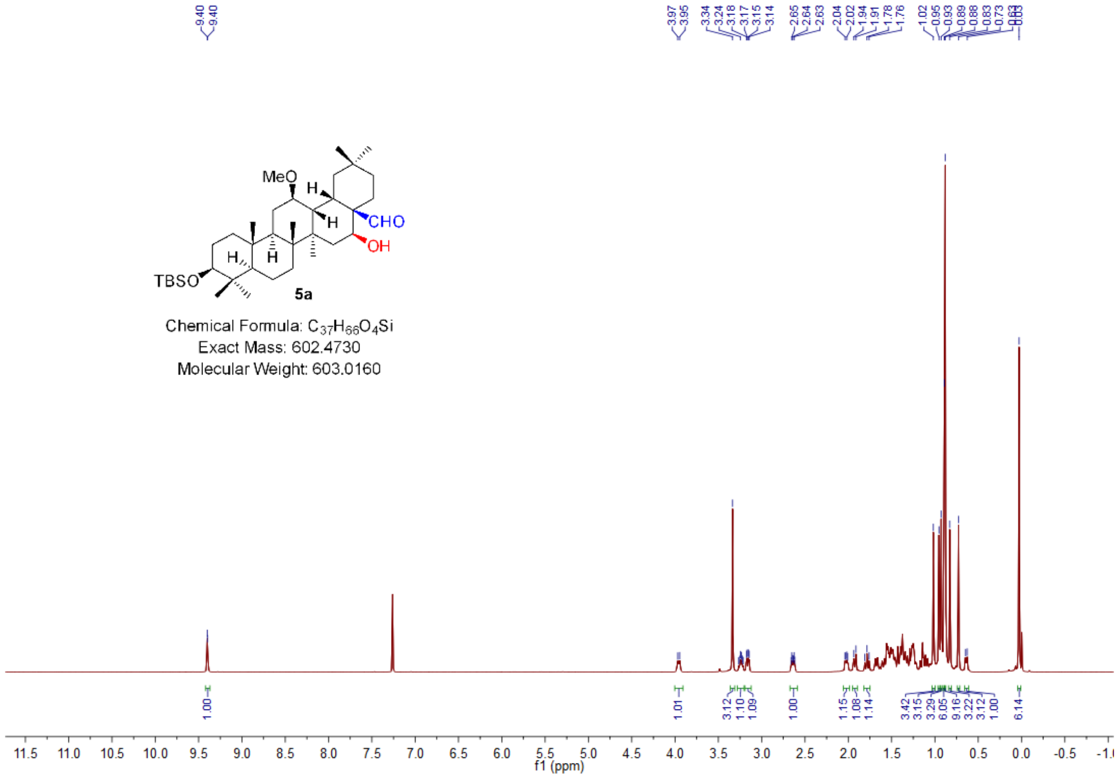

Supplementary Figure 50. ^{1}**H-NMR spectrum of compound 5-a (500 MHz, CDCl$_3$, 25 ^{o}C)**

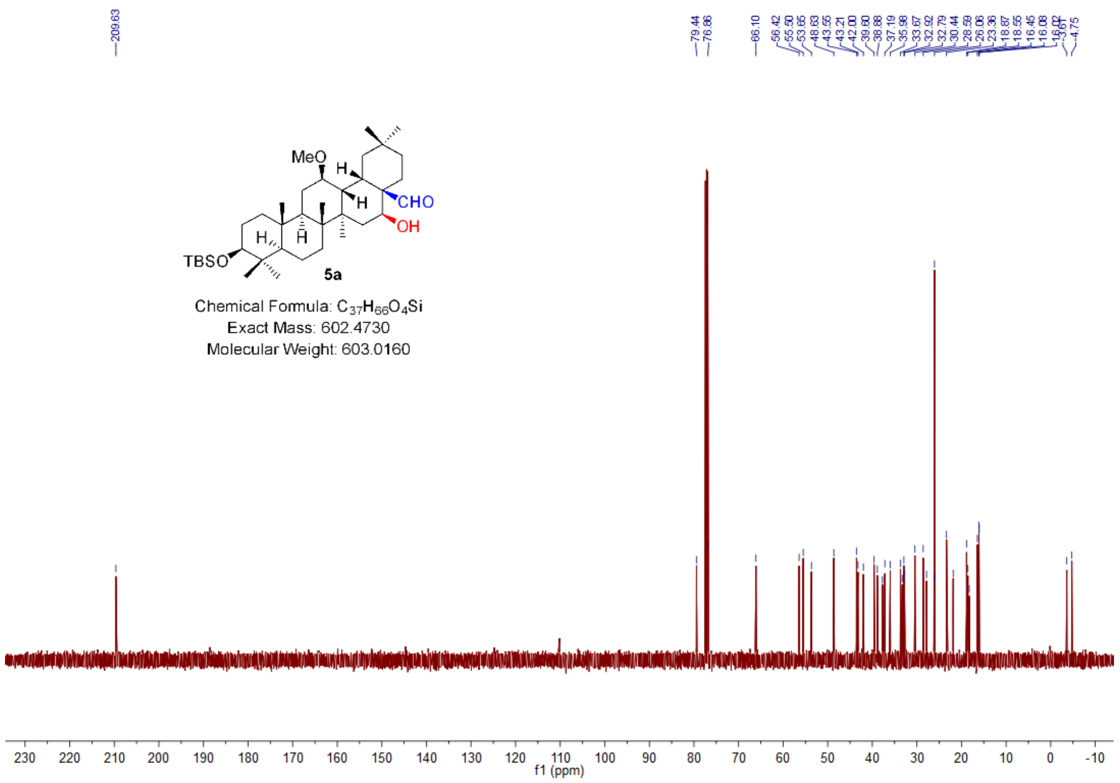

Supplementary Figure 51. ^{13}**C-NMR spectrum of compound 5-a (126 MHz, $CDCl_3$, 25 °C)**

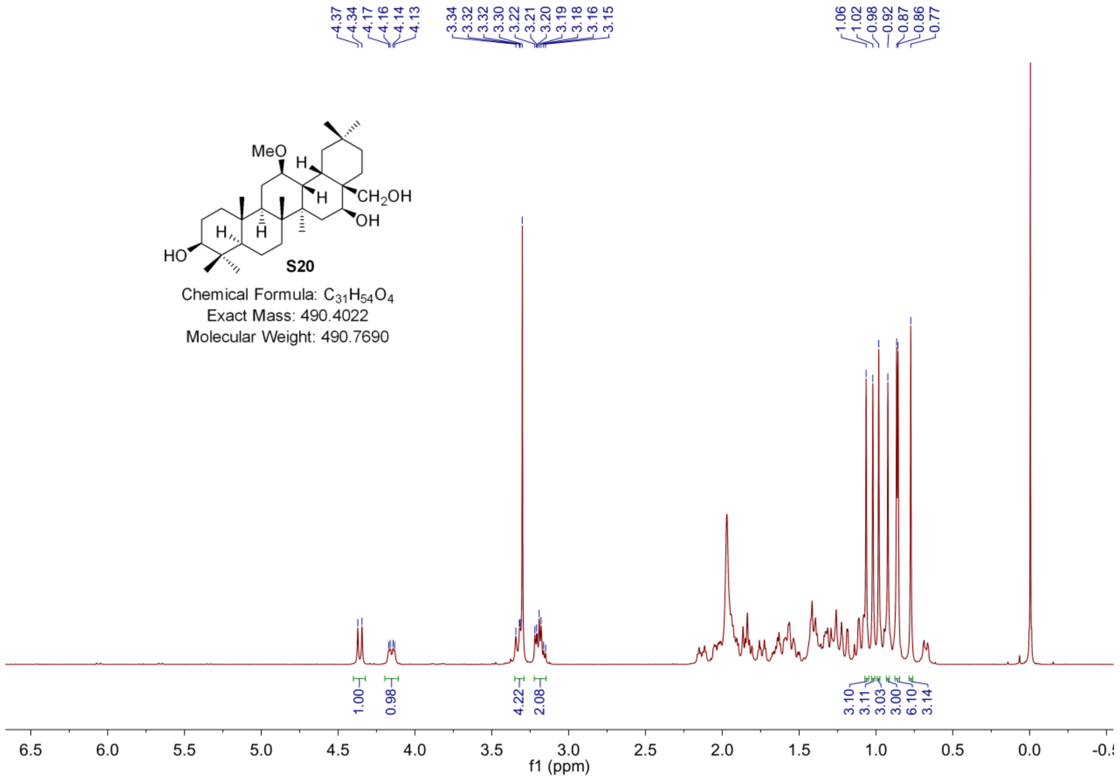

Supplementary Figure 52. ^{1}**H-NMR spectrum of compound S20 (400 MHz, $CDCl_3$, 25 °C)**

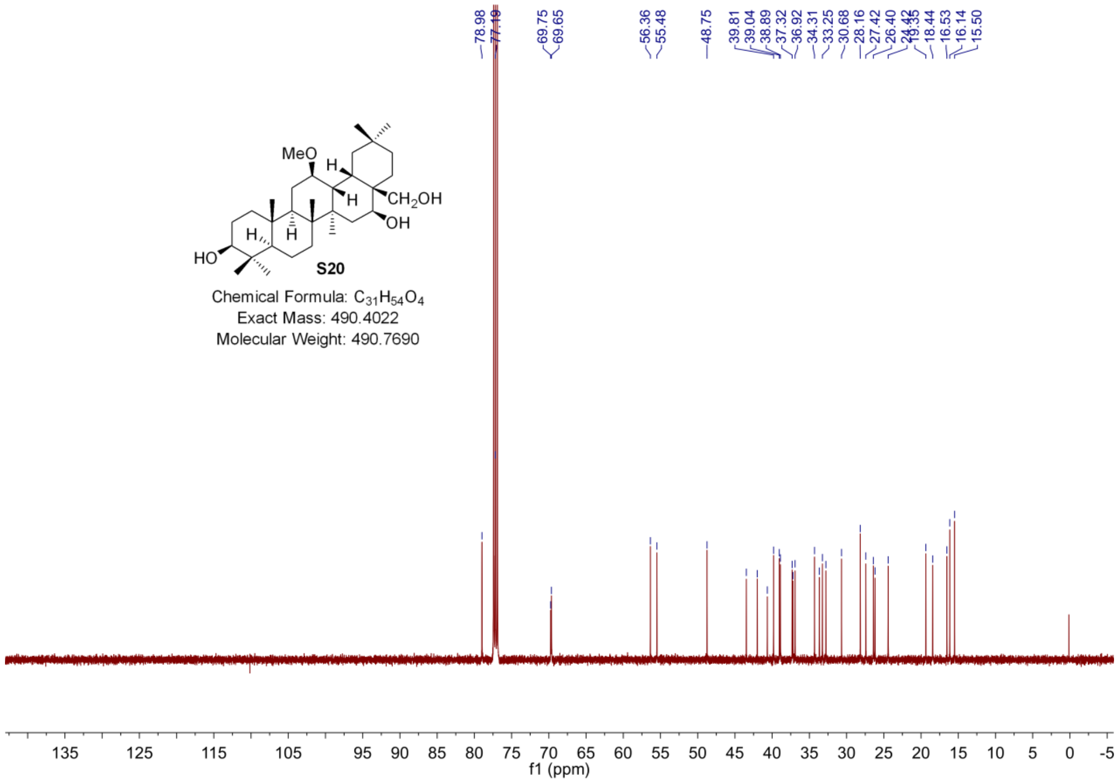

Supplementary Figure 53. ^{13}C-NMR spectrum of compound S20 (126 MHz, $CDCl_3$, 25 ºC)

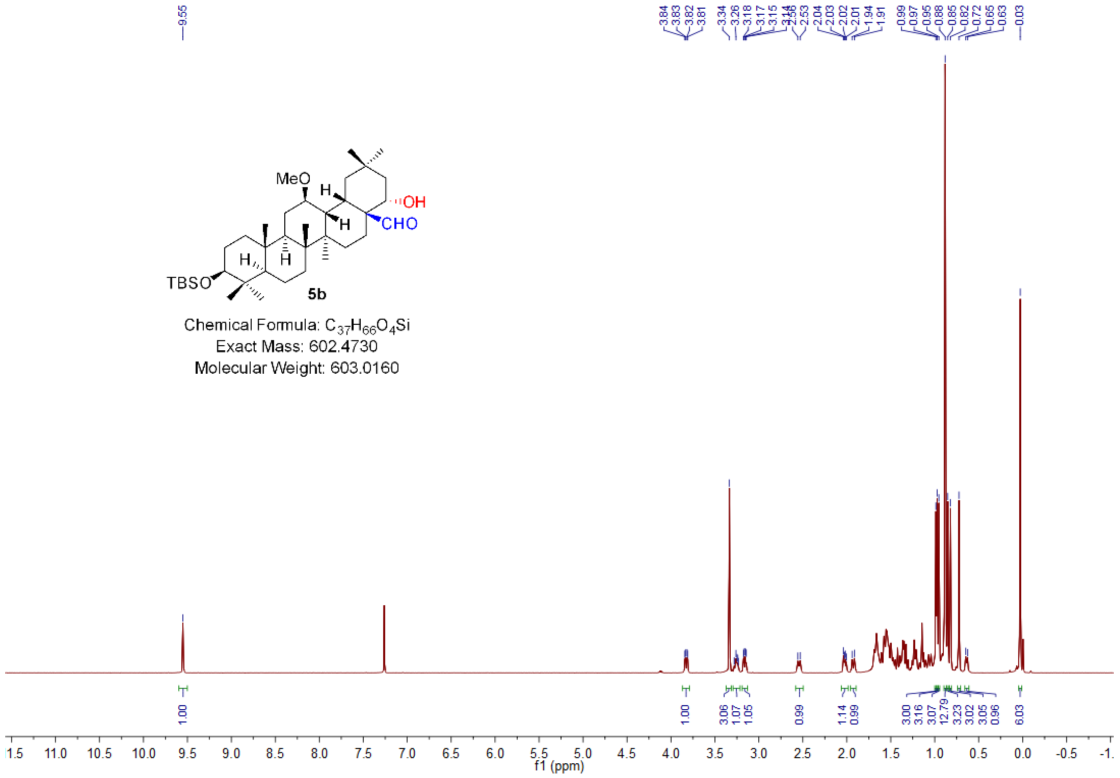

Supplementary Figure 54. ^{1}H-NMR spectrum of compound 5-b (500 MHz, $CDCl_3$, 25 ºC)

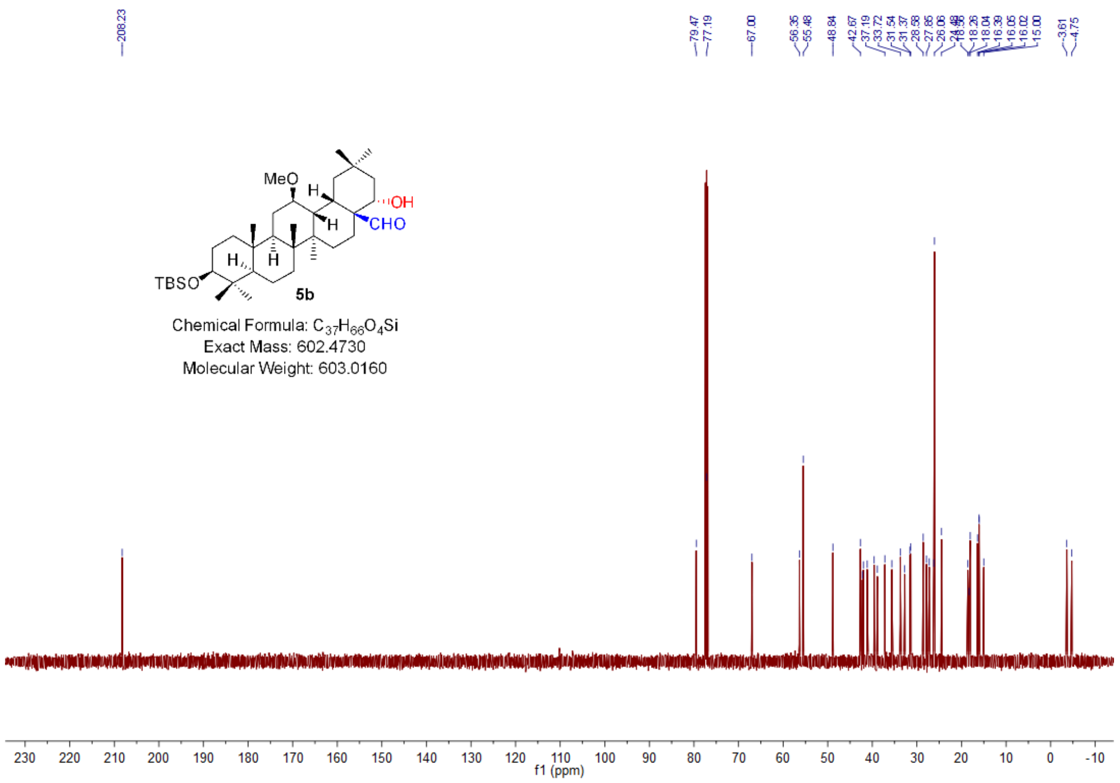

Supplementary Figure 55. ^{13}C-NMR spectrum of compound 5-b (126 MHz, $CDCl_3$, 25 °C)

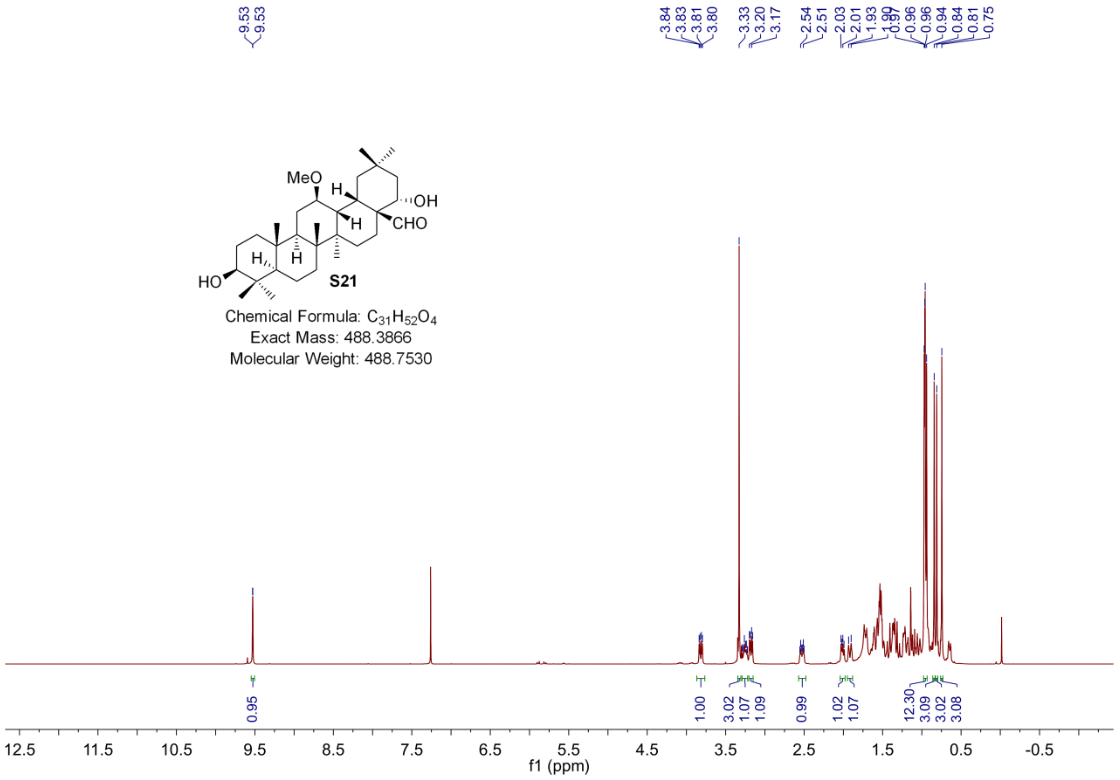

Supplementary Figure 56. ^{1}H-NMR spectrum of compound S21 (400 MHz, $CDCl_3$, 25 °C)

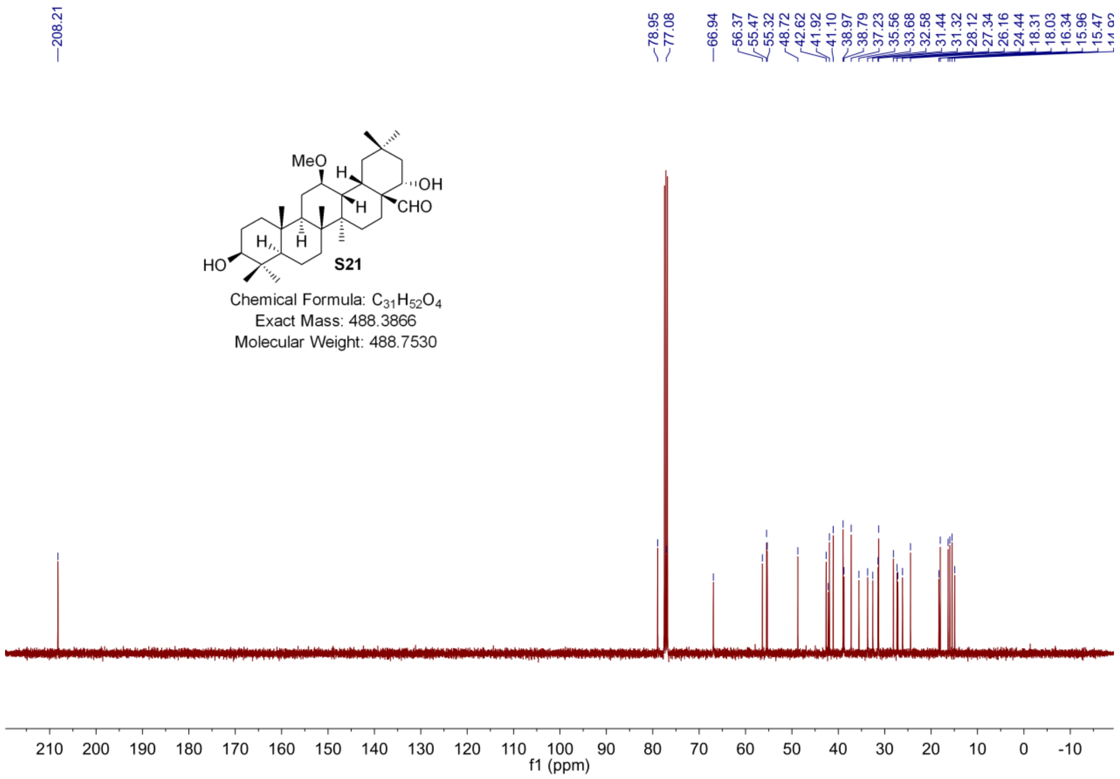

Supplementary Figure 57. ^{13}C-NMR spectrum of compound S21 (101 MHz, $CDCl_3$, 25 ^{o}C)

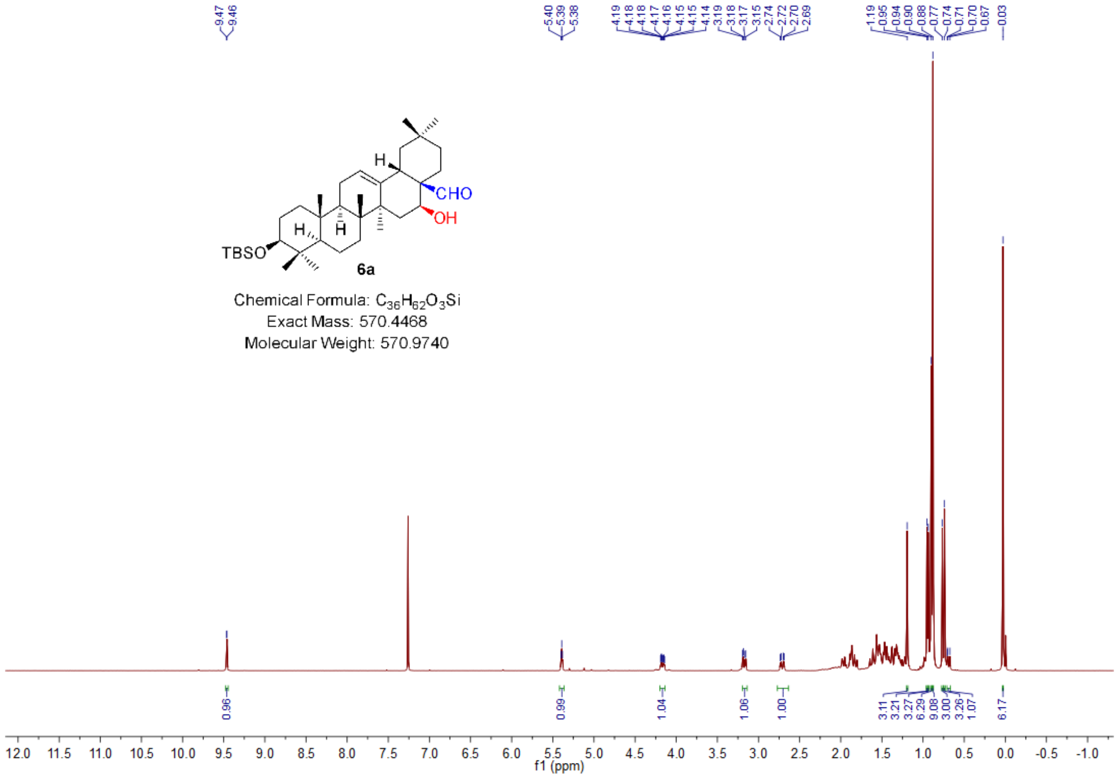

Supplementary Figure 58. ^{1}H-NMR spectrum of compound 6-a (400 MHz, $CDCl_3$, 25 ^{o}C)

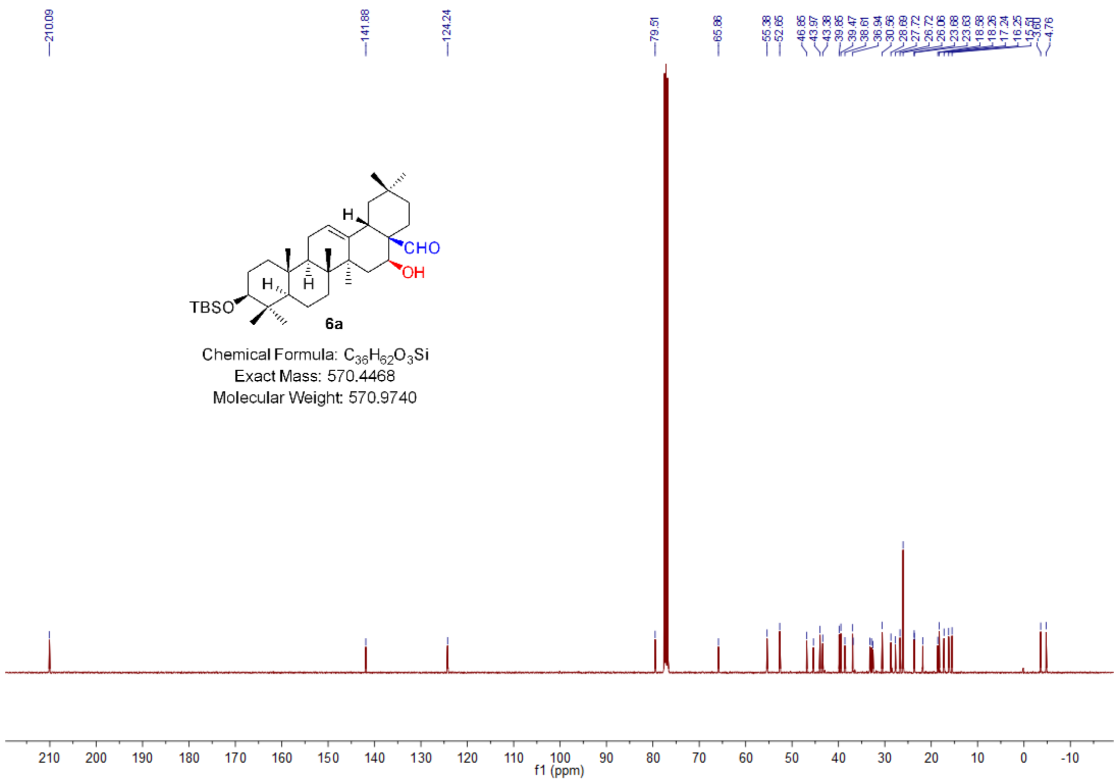

Supplementary Figure 59. ^{13}C-NMR spectrum of compound 6-a (101 MHz, $CDCl_3$, 25 ^{o}C)

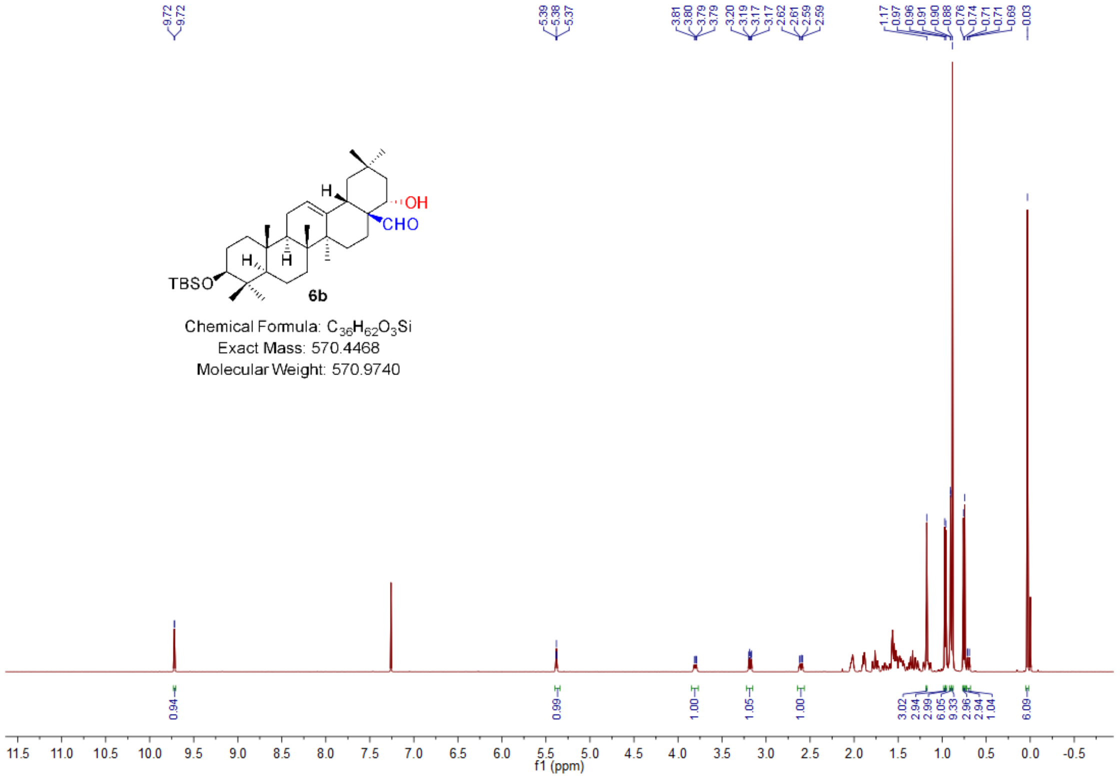

Supplementary Figure 60. ^{1}H-NMR spectrum of compound 6-b (500 MHz, $CDCl_3$, 25 ^{o}C)

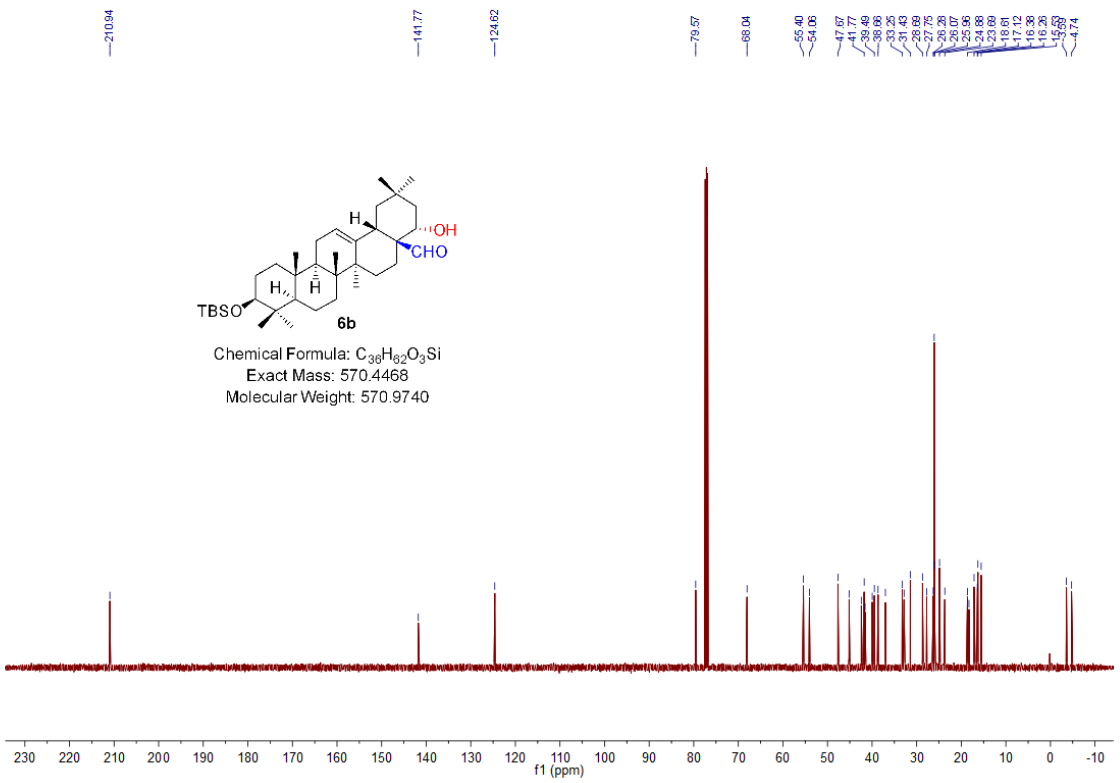

Supplementary Figure 61. ^{13}**C-NMR spectrum of compound 6-b (126 MHz, CDCl$_3$, 25 °C)**

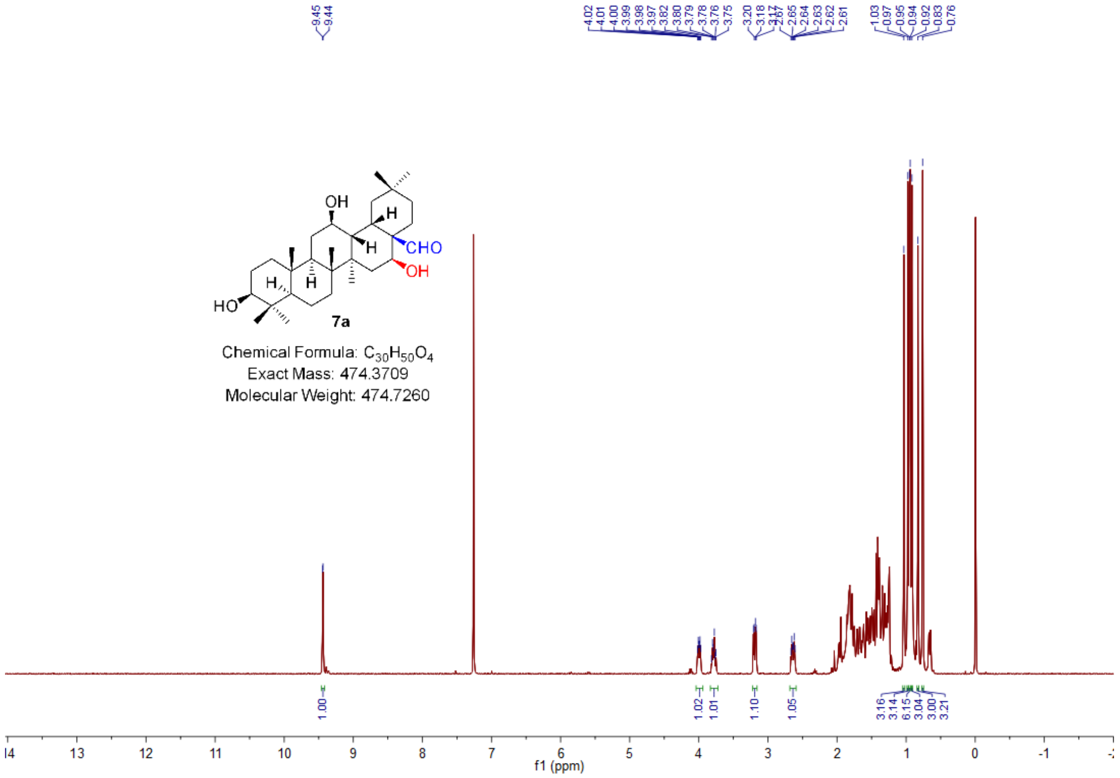

Supplementary Figure 62. ^{1}**H-NMR spectrum of compound 7-a (400 MHz, CDCl$_3$, 25 °C)**

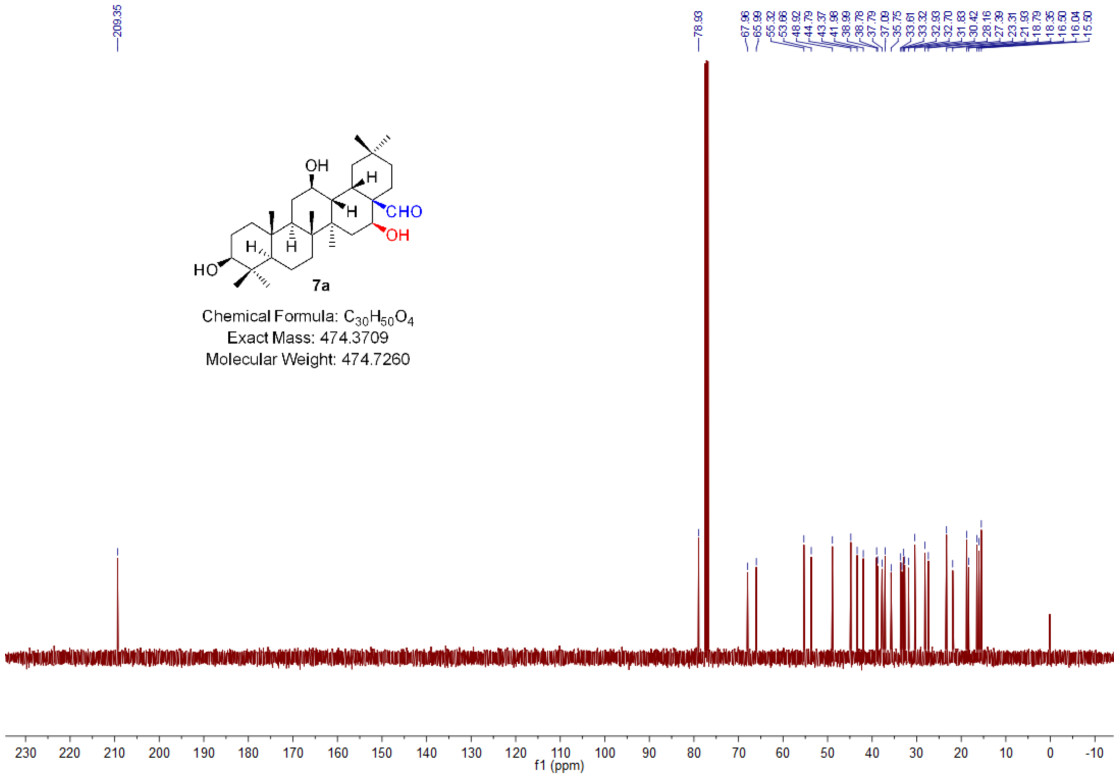

Supplementary Figure 63. ^{13}C-NMR spectrum of compound 7-a (101 MHz, $CDCl_3$, 25 °C)

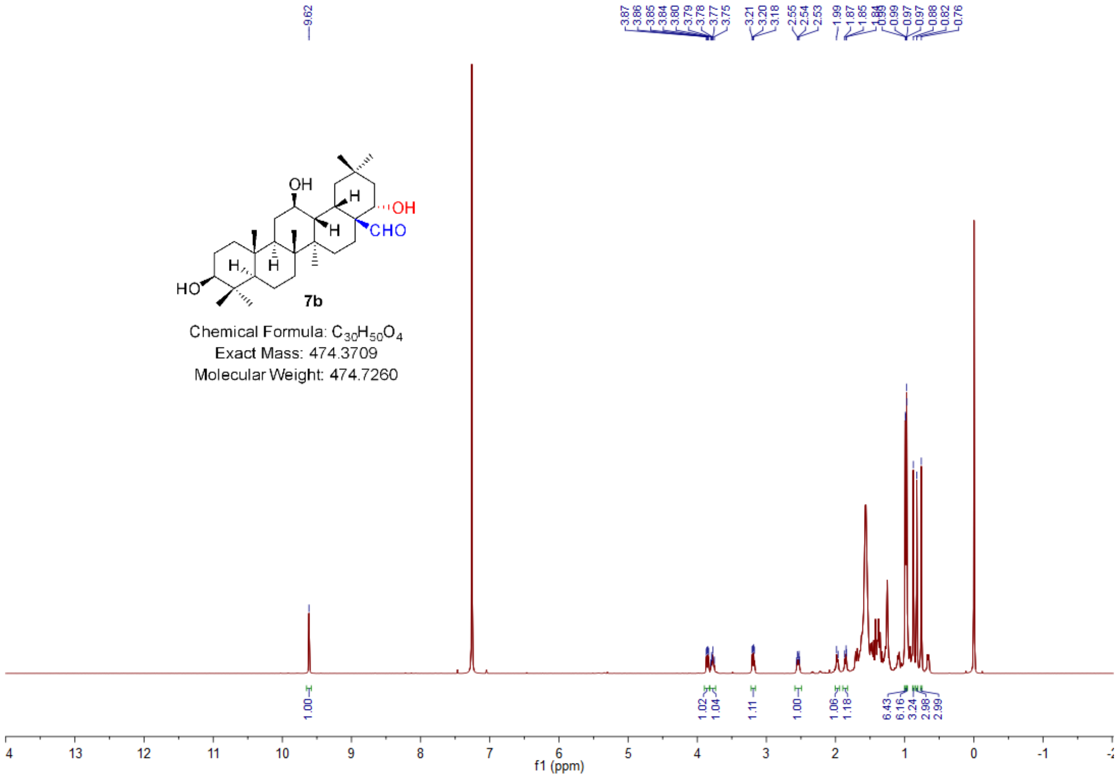

Supplementary Figure 64. ^{1}H-NMR spectrum of compound 7-b (500 MHz, $CDCl_3$, 25 °C)

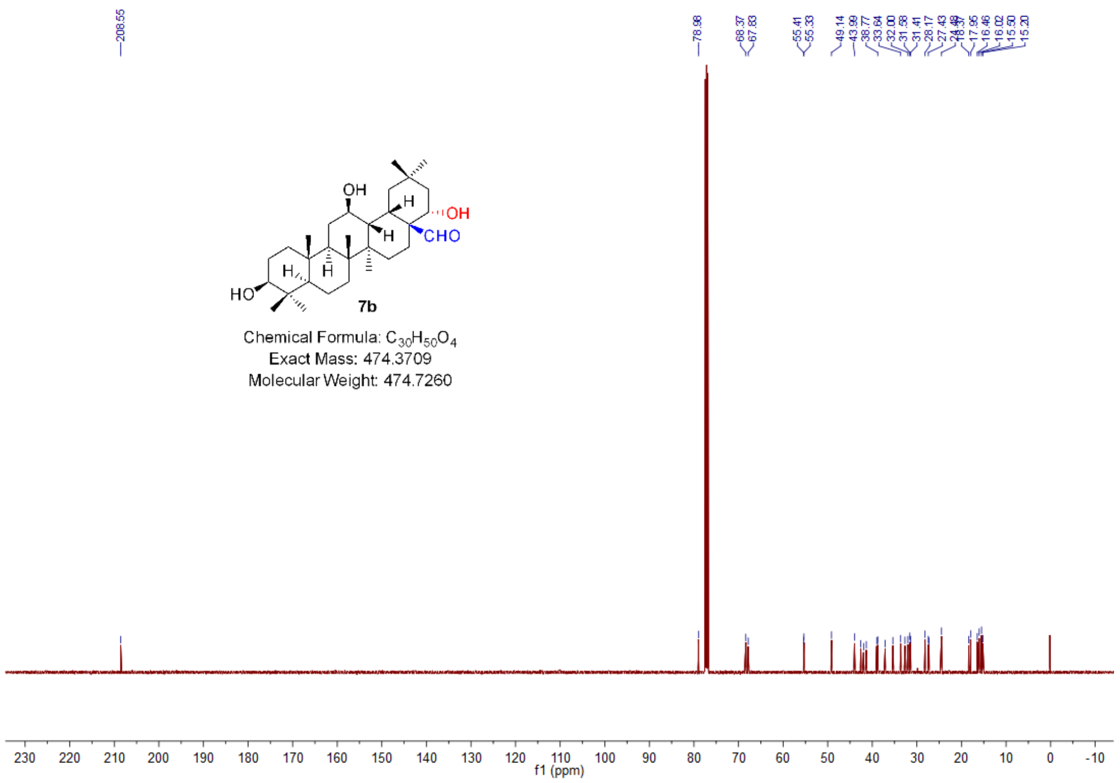

Supplementary Figure 65. **^{13}C-NMR spectrum of compound 7-b (126 MHz, $CDCl_3$, 25 °C)**

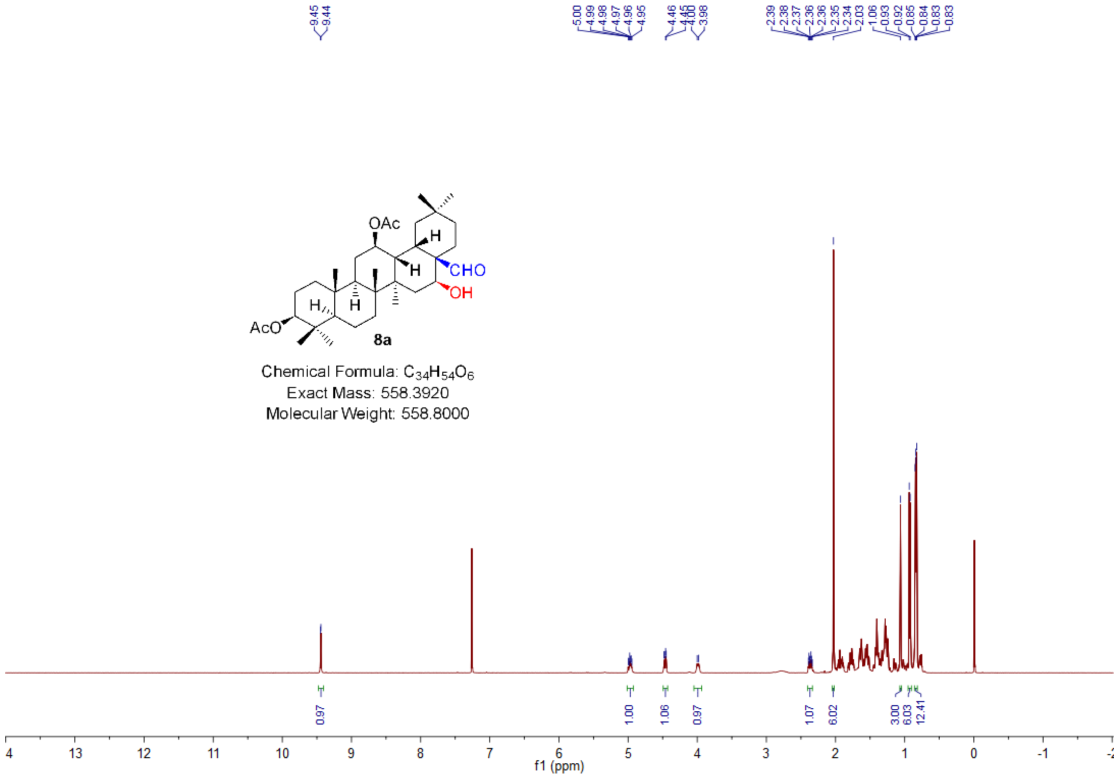

Supplementary Figure 66. **^{1}H-NMR spectrum of compound 8-a (500 MHz, $CDCl_3$, 25 °C)**

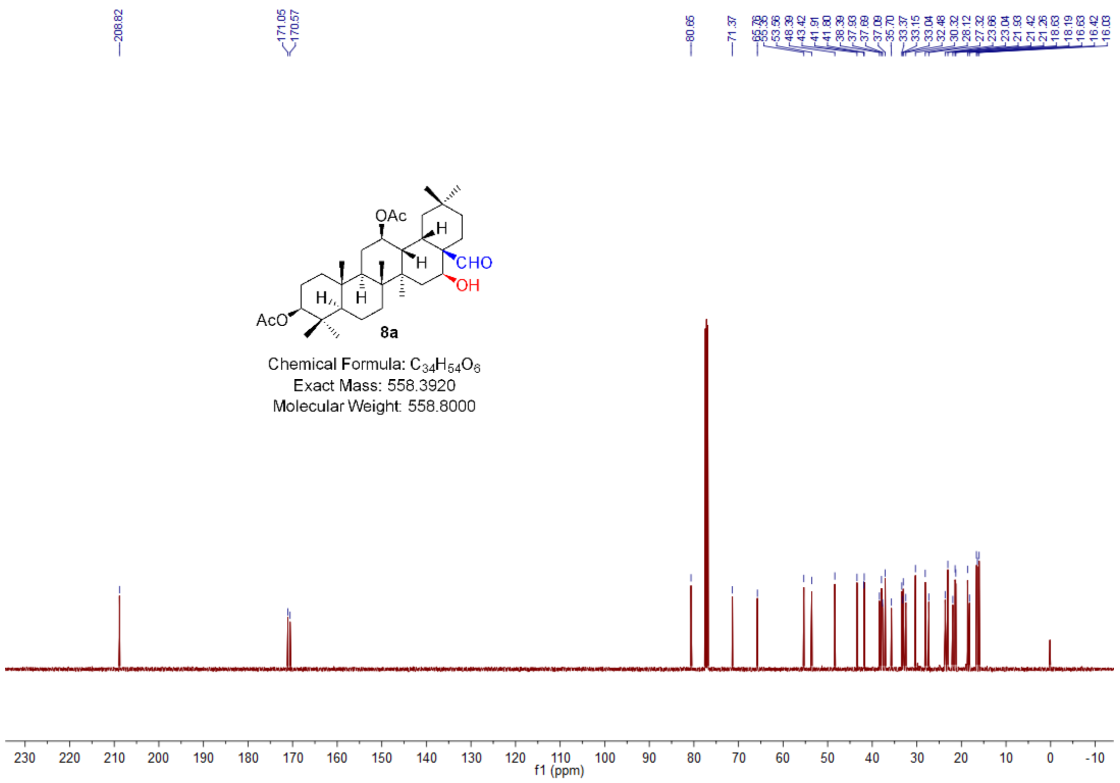

Supplementary Figure 67. ^{13}**C-NMR spectrum of compound 8-a (126 MHz, $CDCl_3$, 25 °C)**

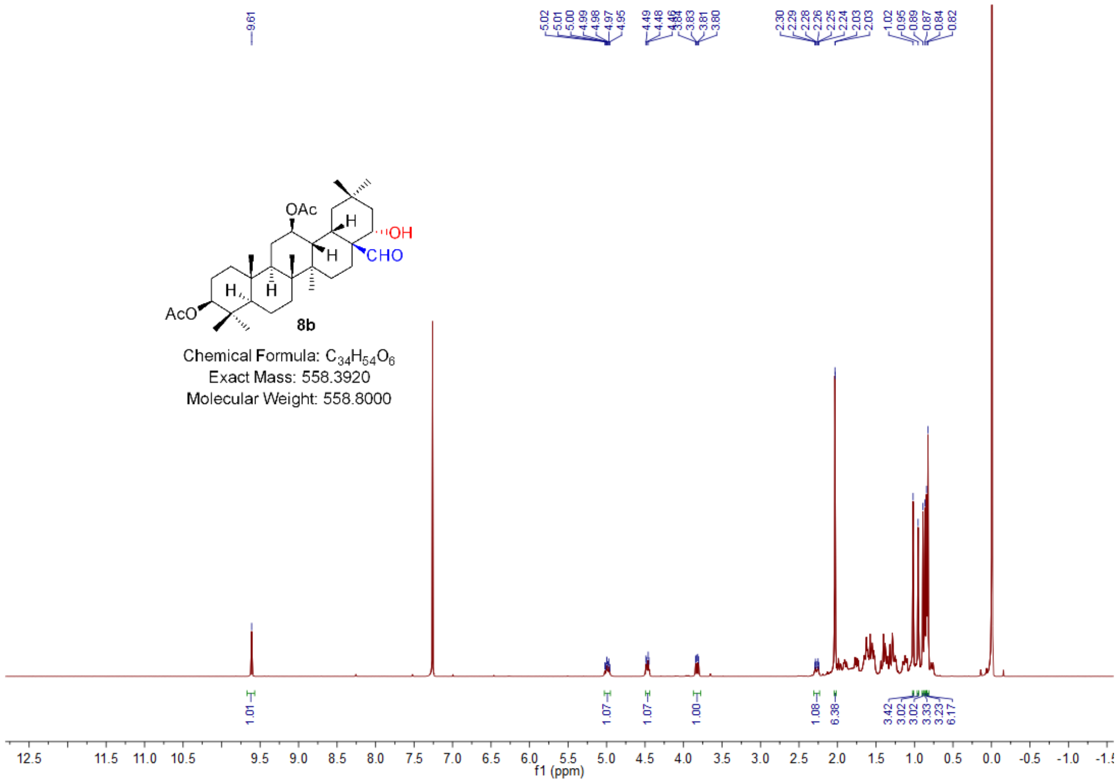

Supplementary Figure 68. ^{1}**H-NMR spectrum of compound 8-b (400 MHz, $CDCl_3$, 25 °C)**

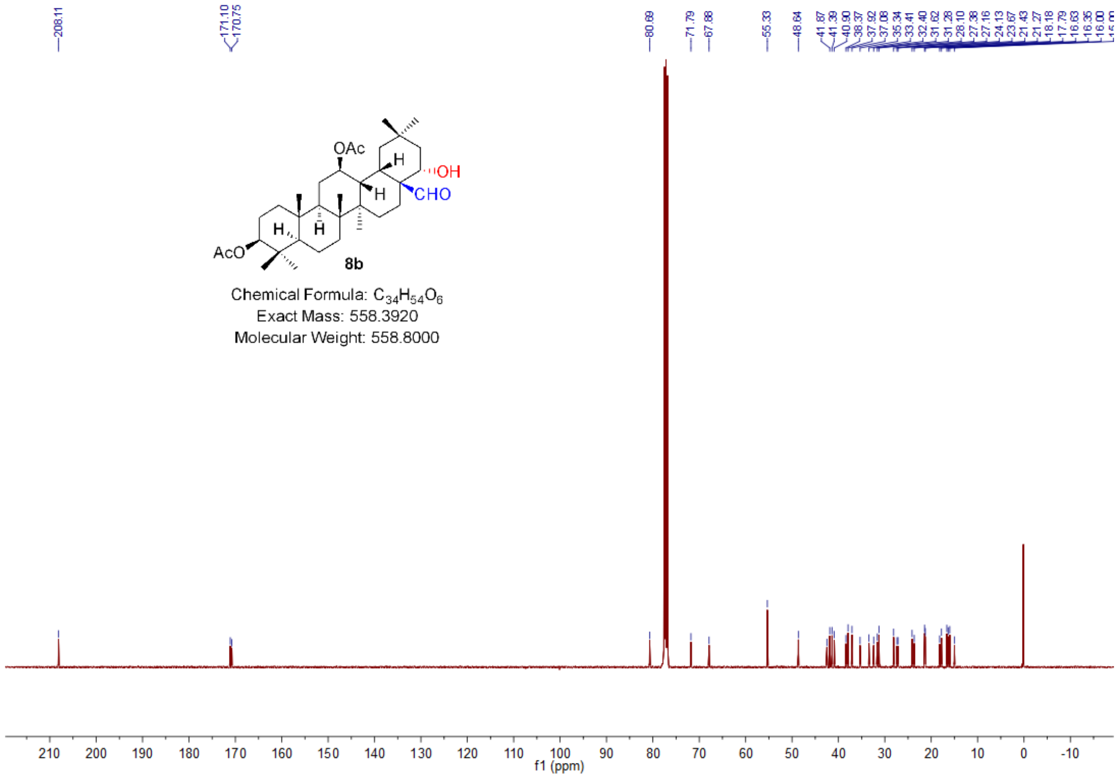

Supplementary Figure 69. [13]**C-NMR spectrum of compound 8-b (101 MHz, $CDCl_3$, 25 °C)**

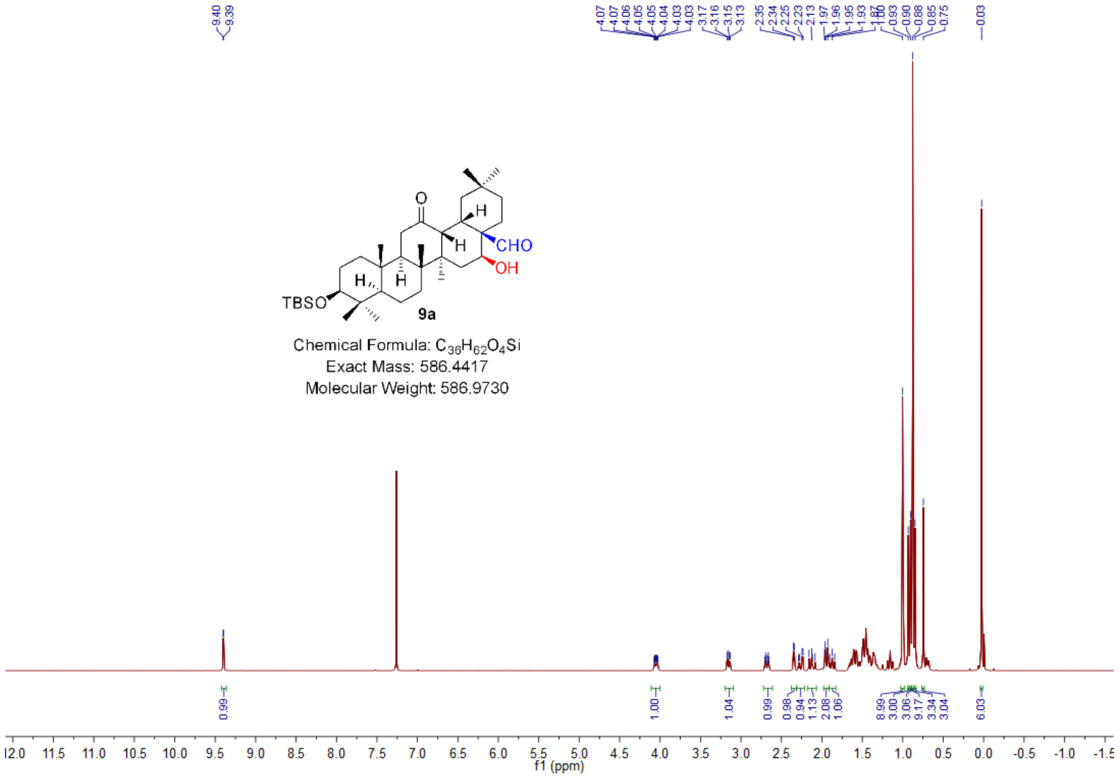

Supplementary Figure 70. [1]**H-NMR spectrum of compound 9-a (400 MHz, $CDCl_3$, 25 °C)**

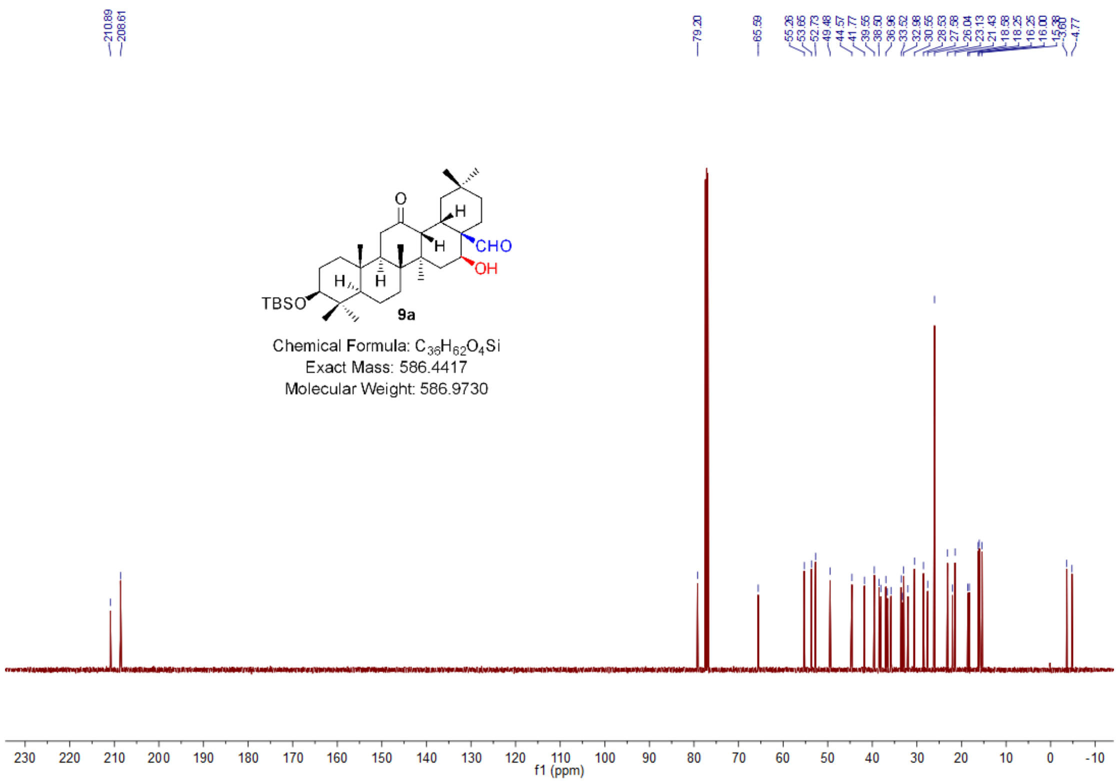

Supplementary Figure 71. **^{13}C-NMR spectrum of compound 9-a (126 MHz, $CDCl_3$, 25 °C)**

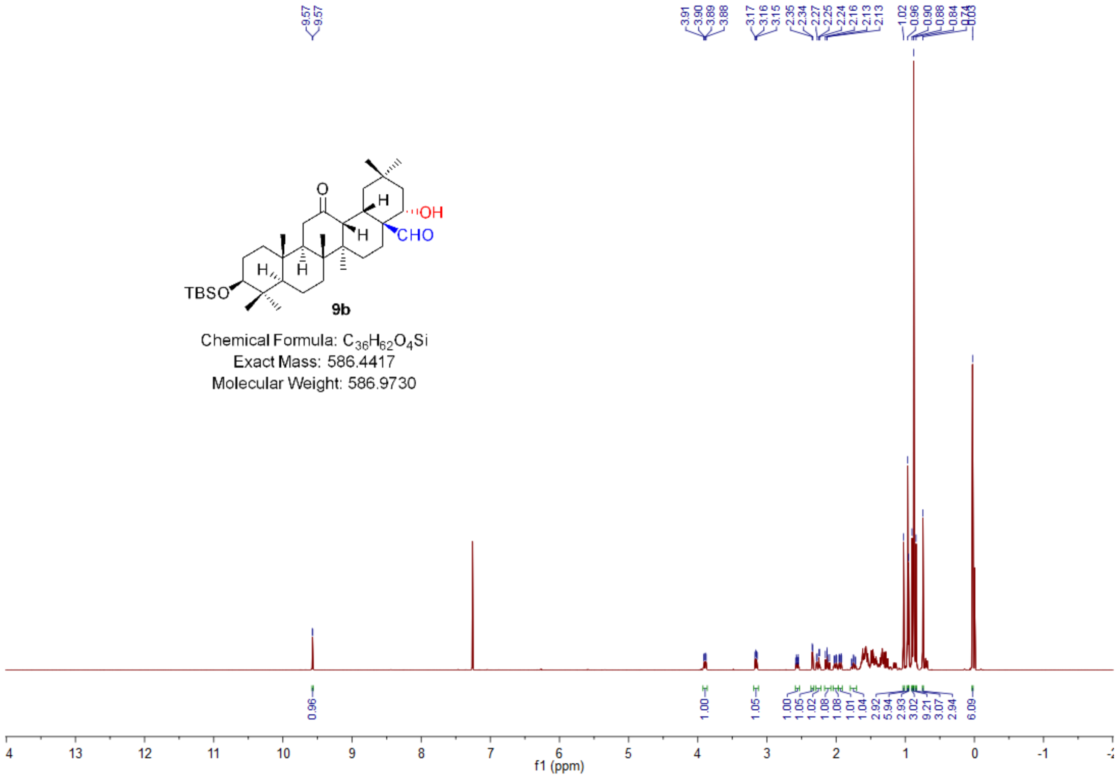

Supplementary Figure 72. **^{1}H-NMR spectrum of compound 9-b (500 MHz, $CDCl_3$, 25 °C)**

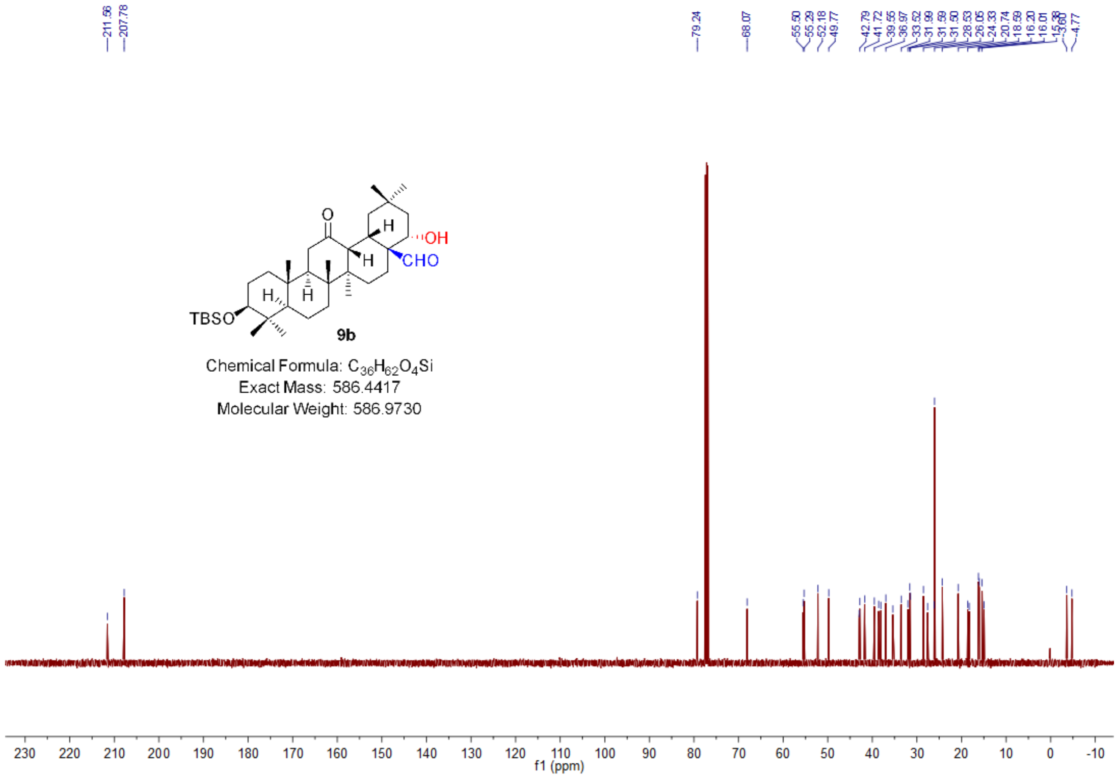

Supplementary Figure 73. ^{13}**C-NMR spectrum of compound 9-b (126 MHz,** $CDCl_3$**, 25 ºC)**

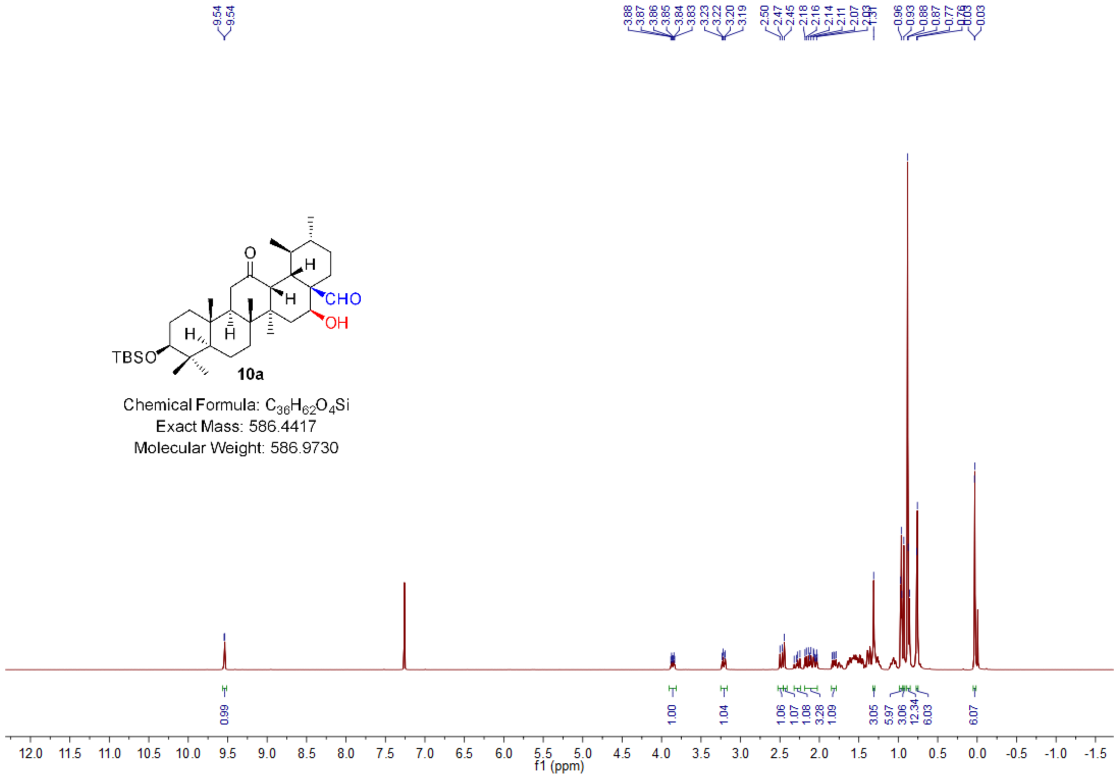

Supplementary Figure 74. ^{1}**H-NMR spectrum of compound 10-a (400 MHz,** $CDCl_3$**, 25 ºC)**

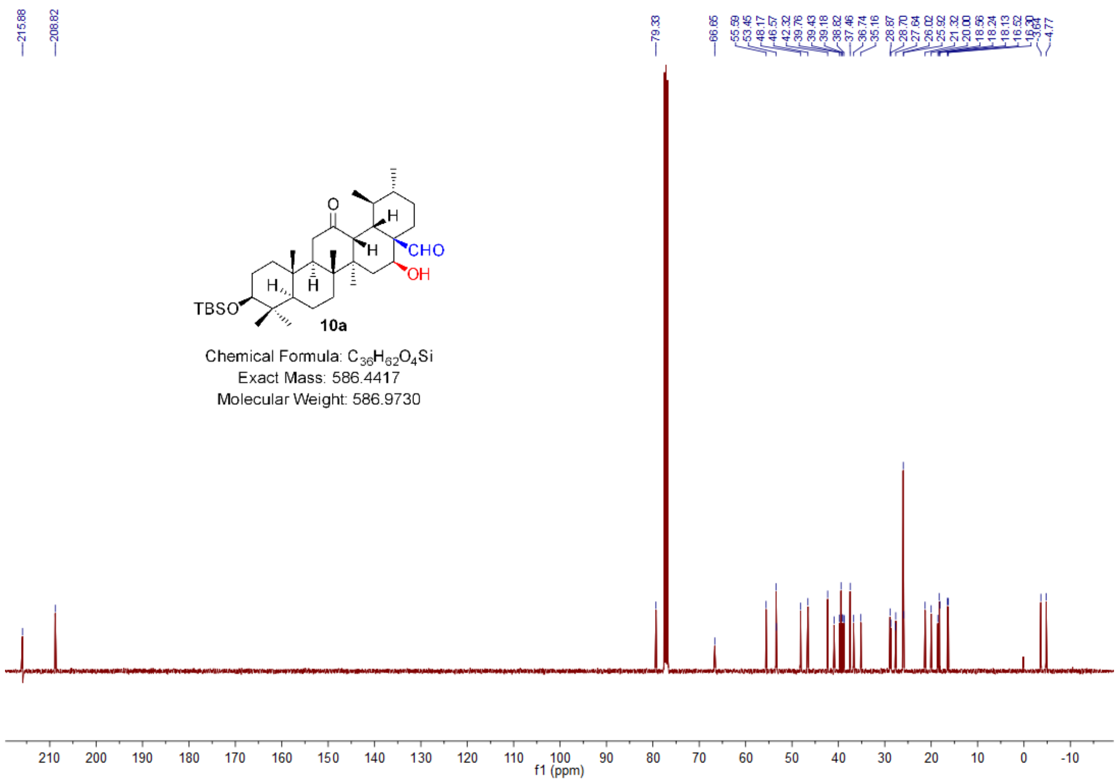

Supplementary Figure 75. ^{13}C-NMR spectrum of compound 10-a (101 MHz, $CDCl_3$, 25 ºC)

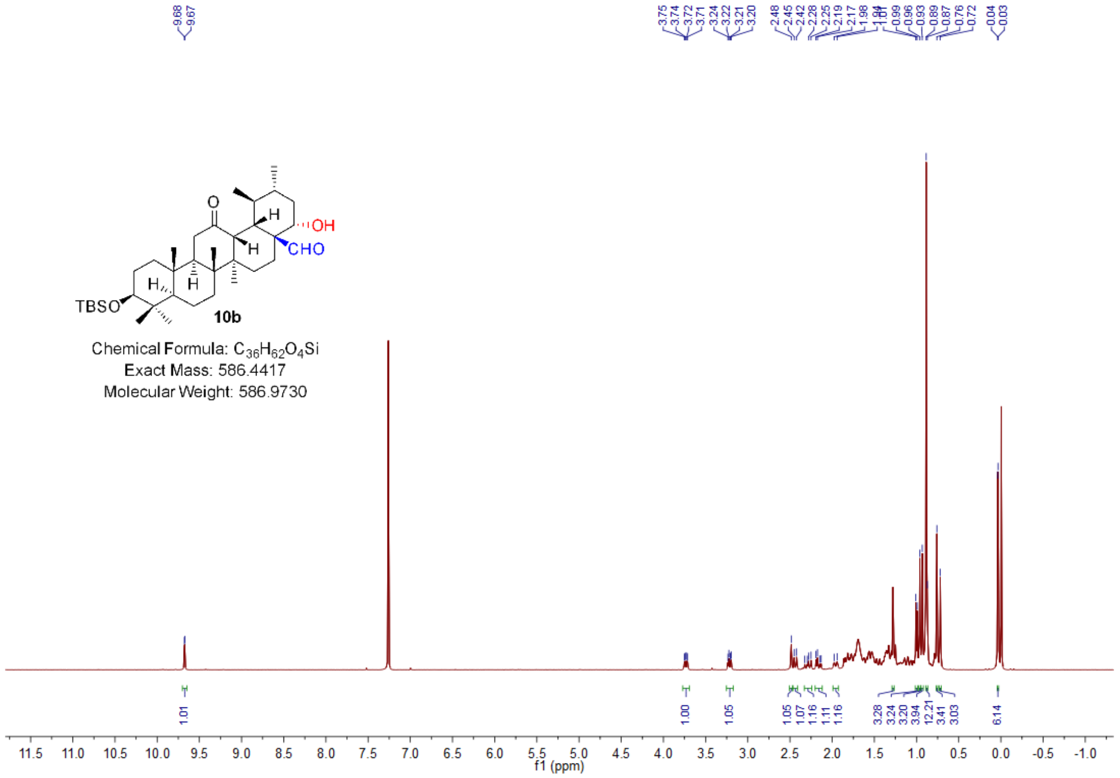

Supplementary Figure 76. ^{1}H-NMR spectrum of compound 10-b (400 MHz, $CDCl_3$, 25 ºC)

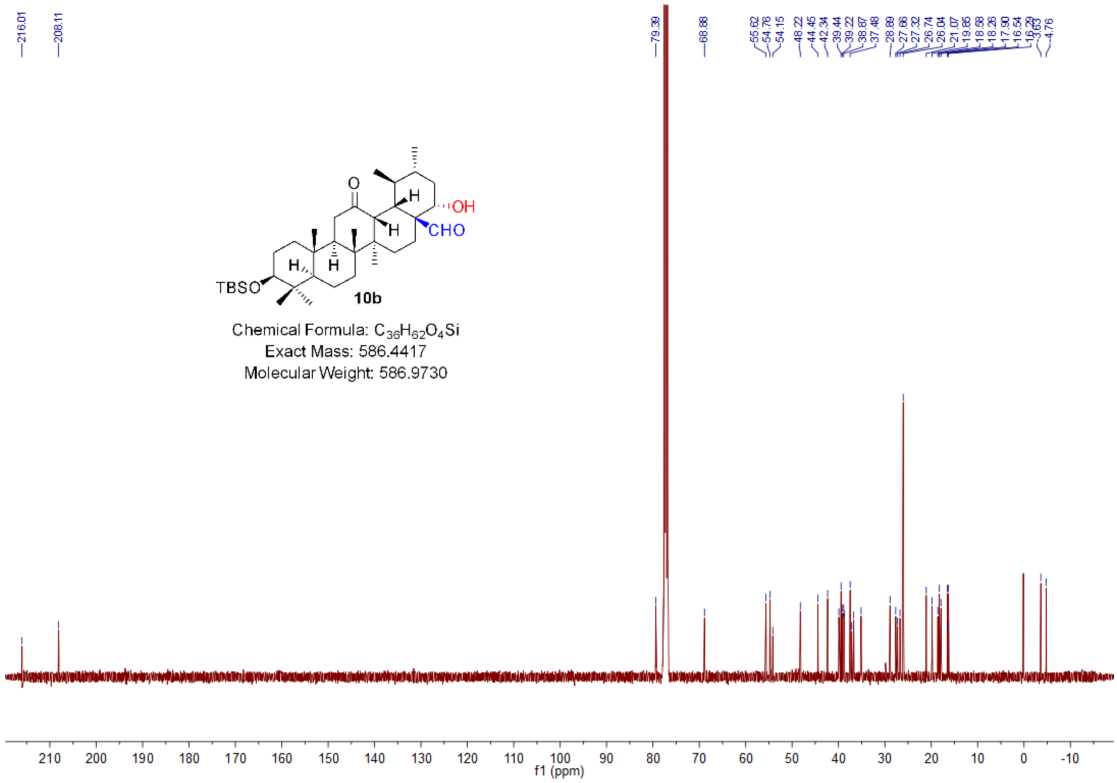

Supplementary Figure 77. ^{13}**C-NMR spectrum of compound 10-b (101 MHz, CDCl$_3$, 25 °C)**

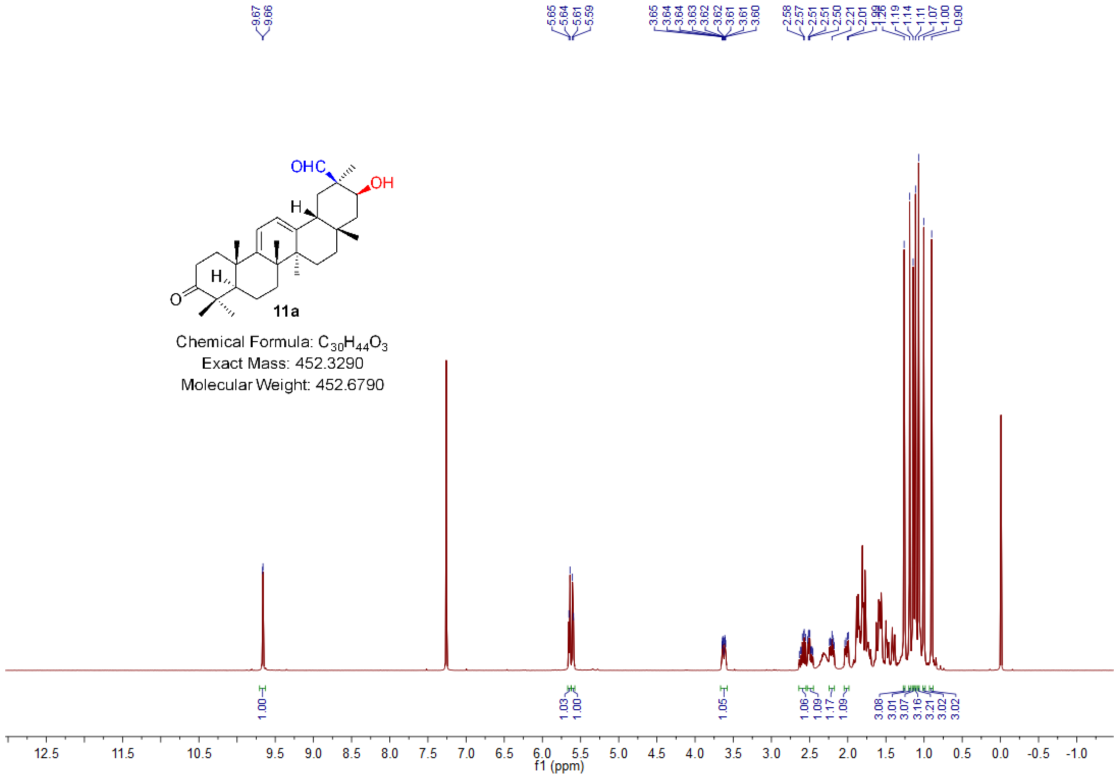

Supplementary Figure 78. ^{1}**H NMR-spectrum of compound 11-a (400 MHz, CDCl$_3$, 25 °C)**

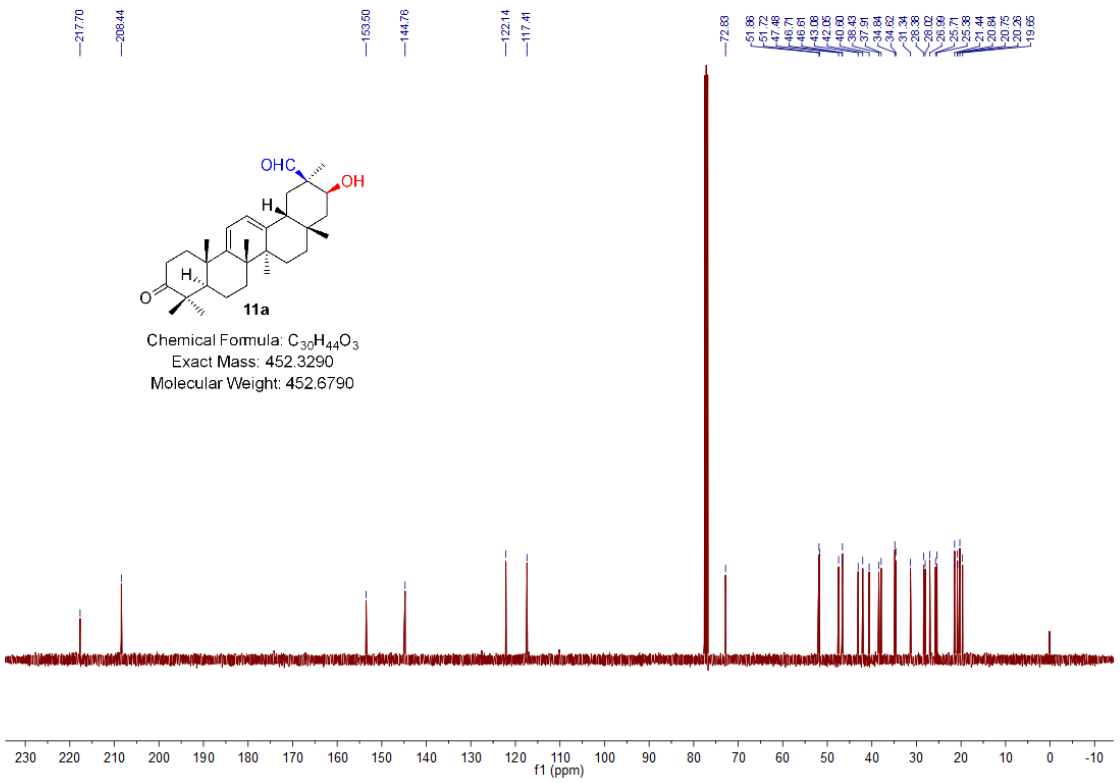

Supplementary Figure 79. ^{13}C-NMR spectrum of compound 11-a (101 MHz, $CDCl_3$, 25 ^{o}C)

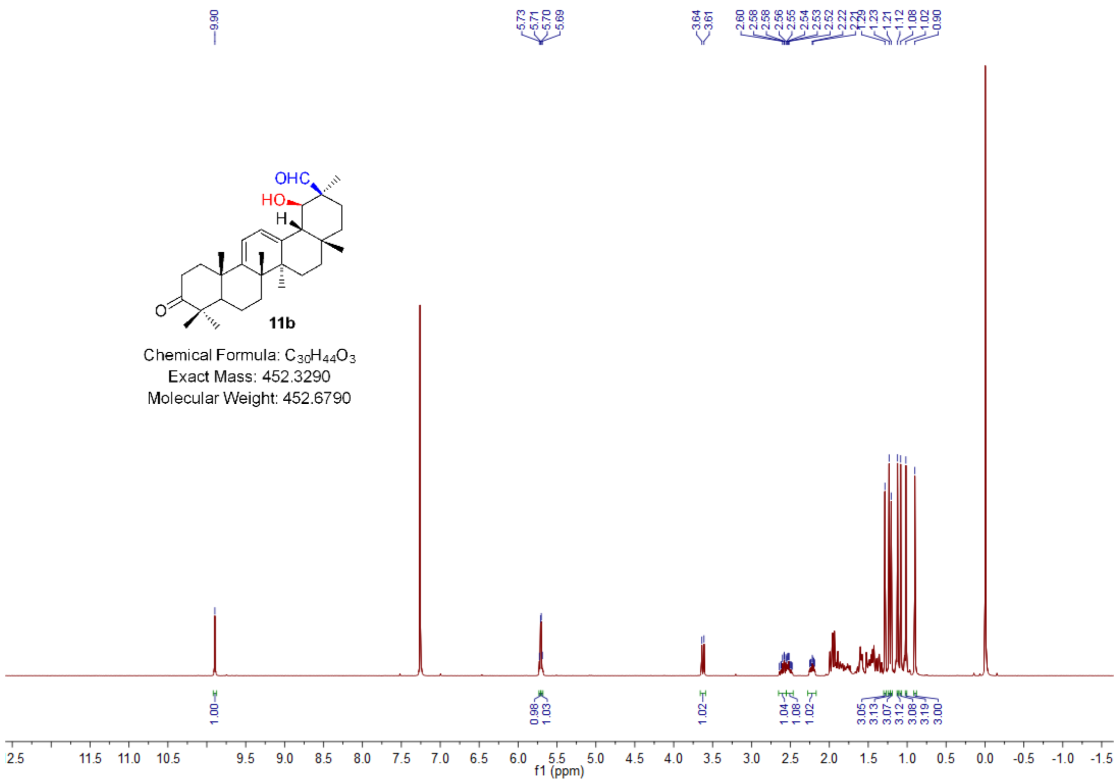

Supplementary Figure 80. ^{1}H-NMR spectrum of compound 11-b (400 MHz, $CDCl_3$, 25 ^{o}C)

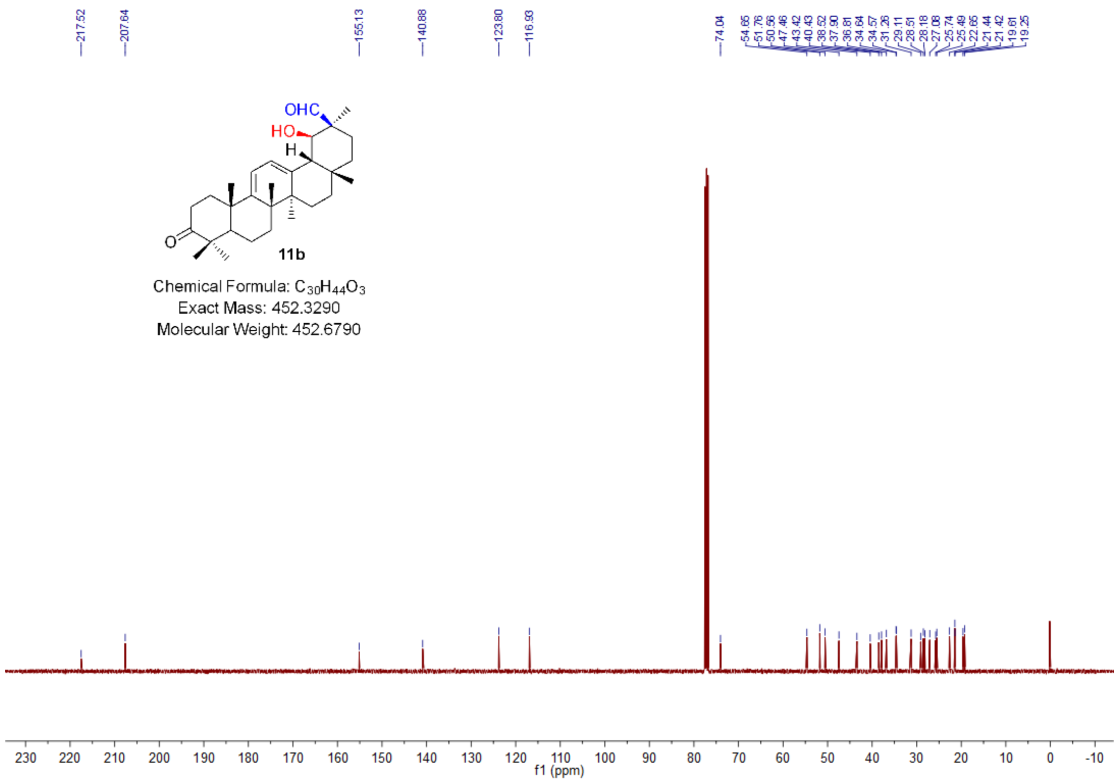

Supplementary Figure 81. **^{13}C-NMR spectrum of compound 11-b (101 MHz, $CDCl_3$, 25 ^{o}C)**

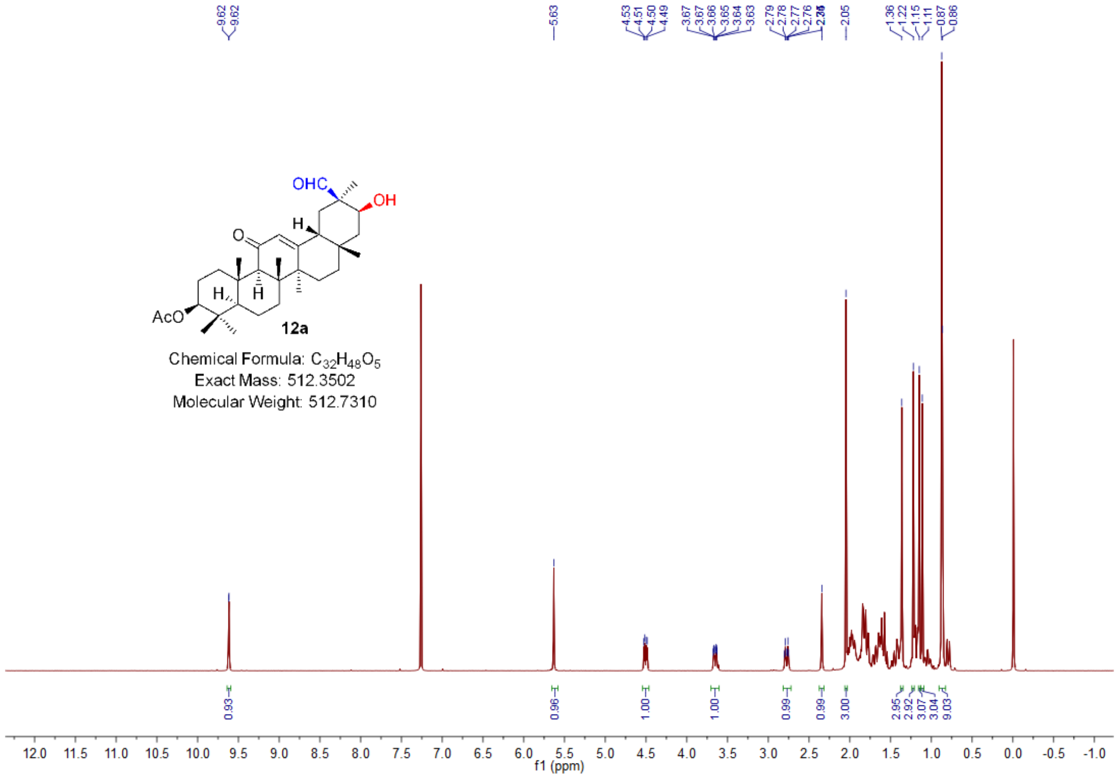

Supplementary Figure 82. **^{1}H-NMR spectrum of compound 12-a (400 MHz, $CDCl_3$, 25 ^{o}C)**

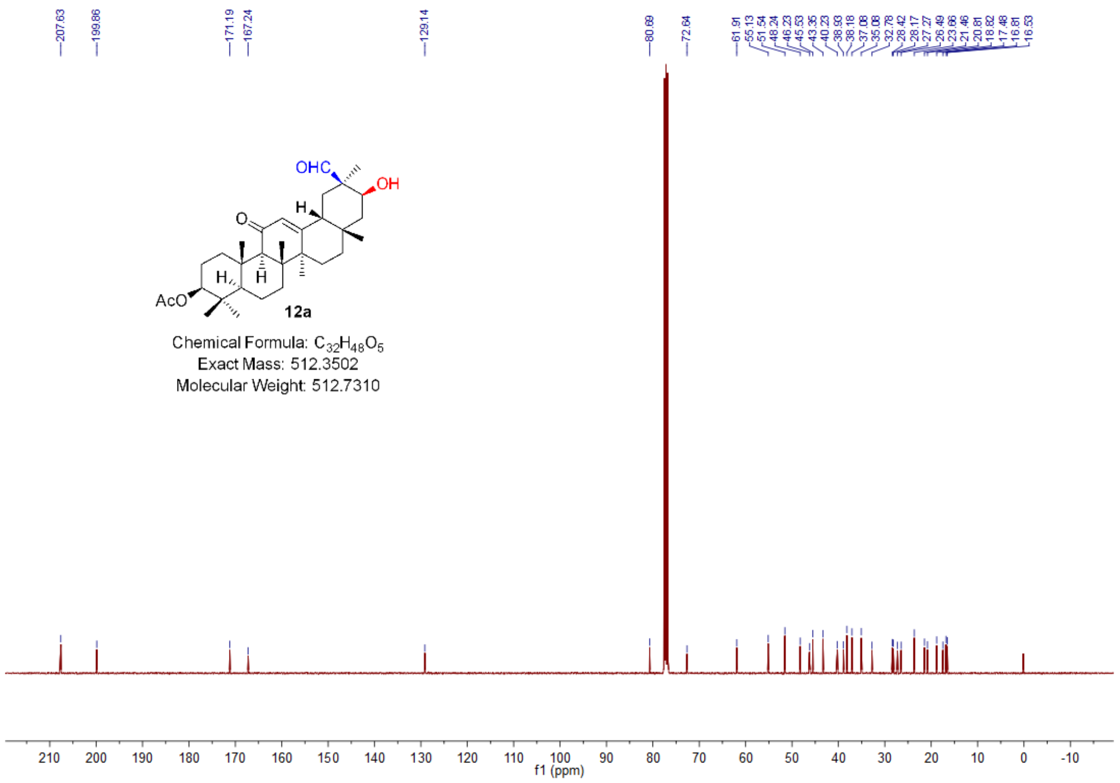

Supplementary Figure 83. ^{13}C-NMR spectrum of compound 12-a (101 MHz, $CDCl_3$, 25 ºC)

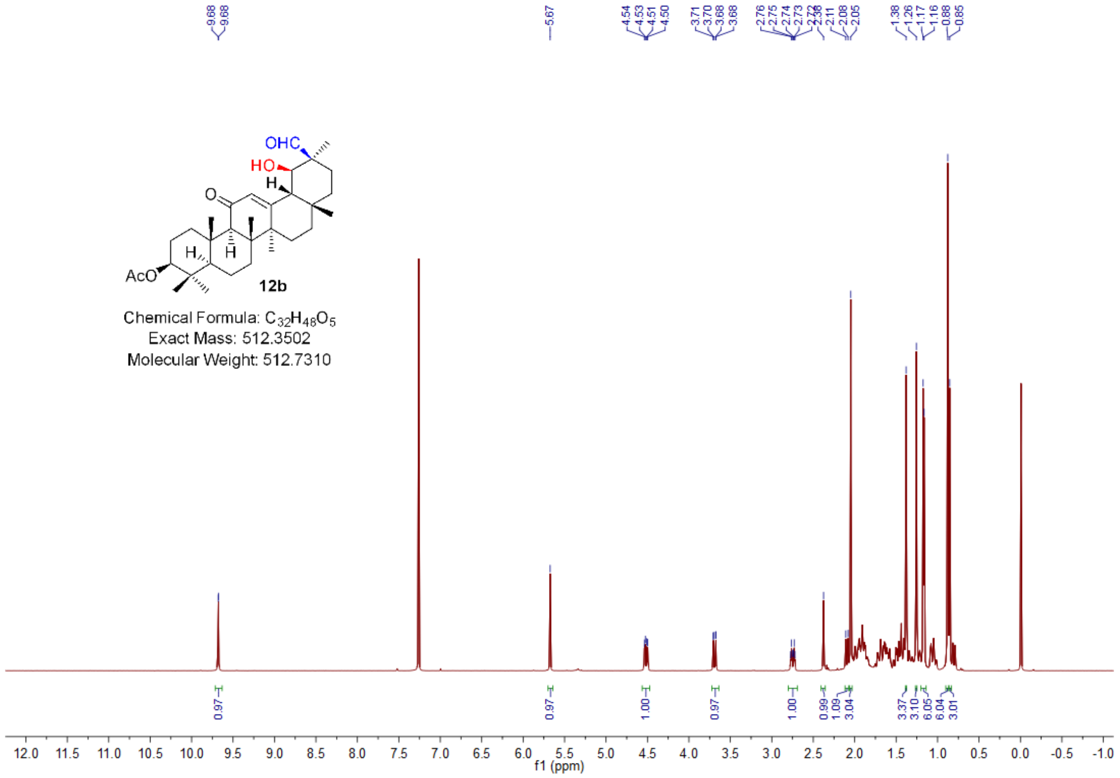

Supplementary Figure 84. ^{1}H-NMR spectrum of compound 12-b (400 MHz, $CDCl_3$, 25 ºC)

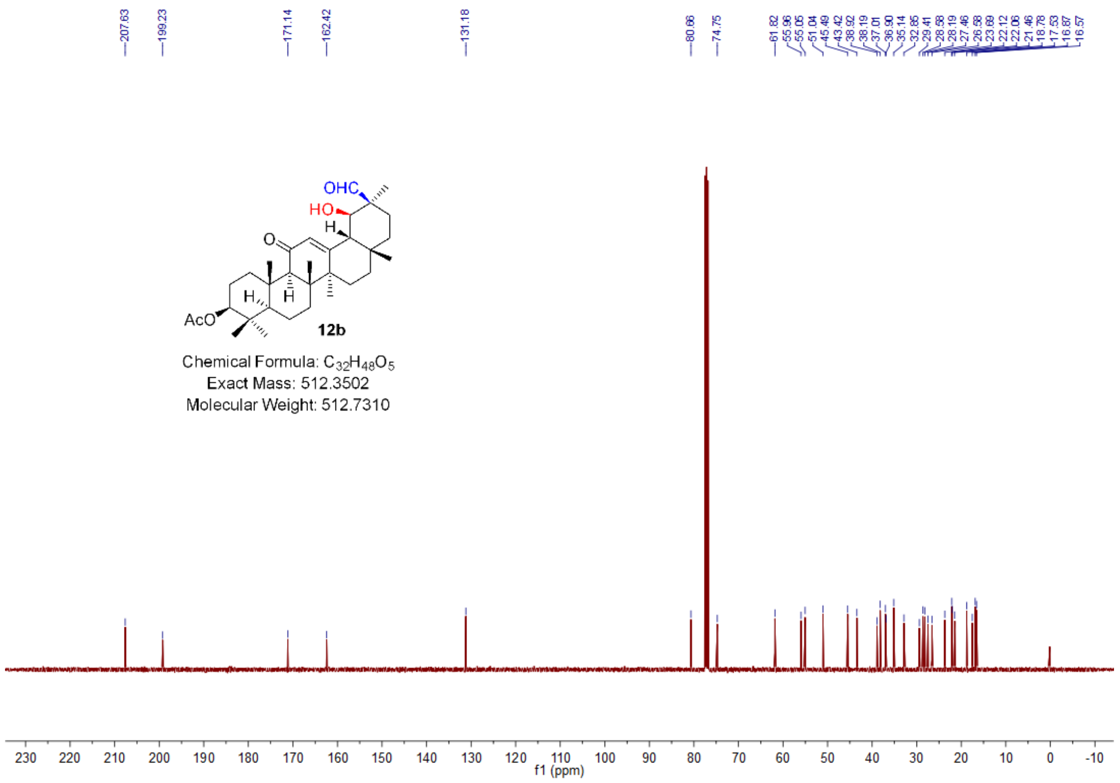

Supplementary Figure 85. ^{13}C-NMR spectrum of compound 12-b (101 MHz, $CDCl_3$, 25 ºC)

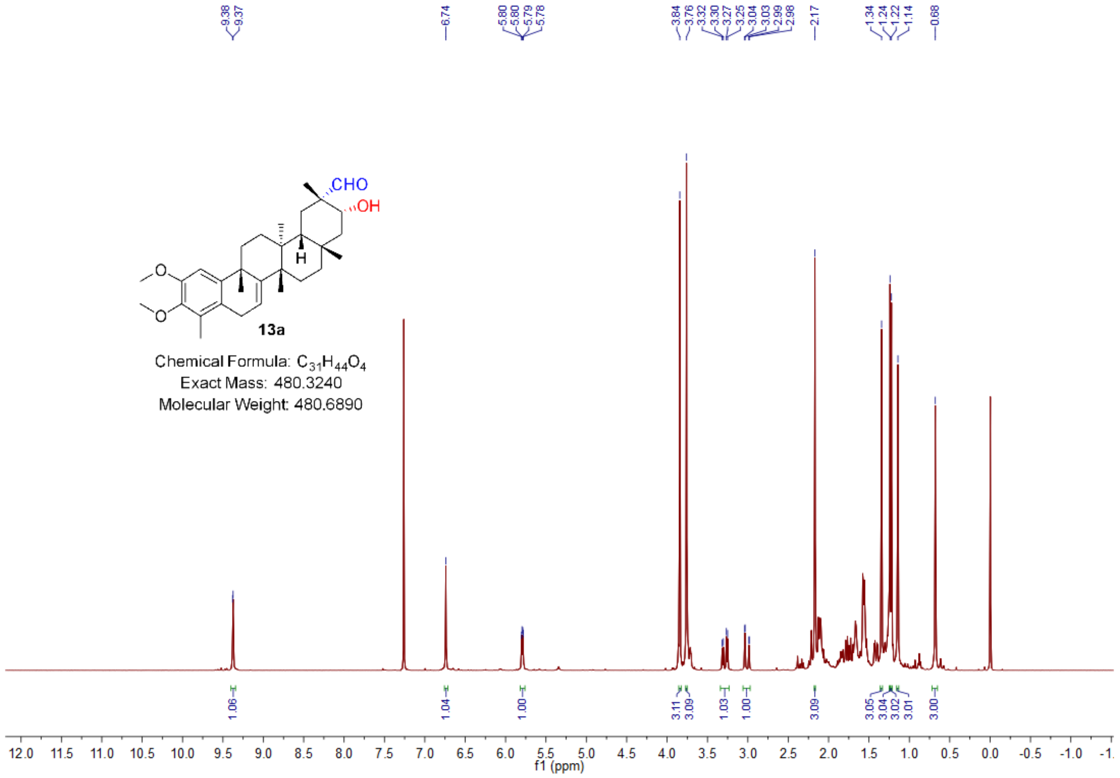

Supplementary Figure 86. ^{1}H-NMR spectrum of compound 13-a (400 MHz, $CDCl_3$, 25 ºC)

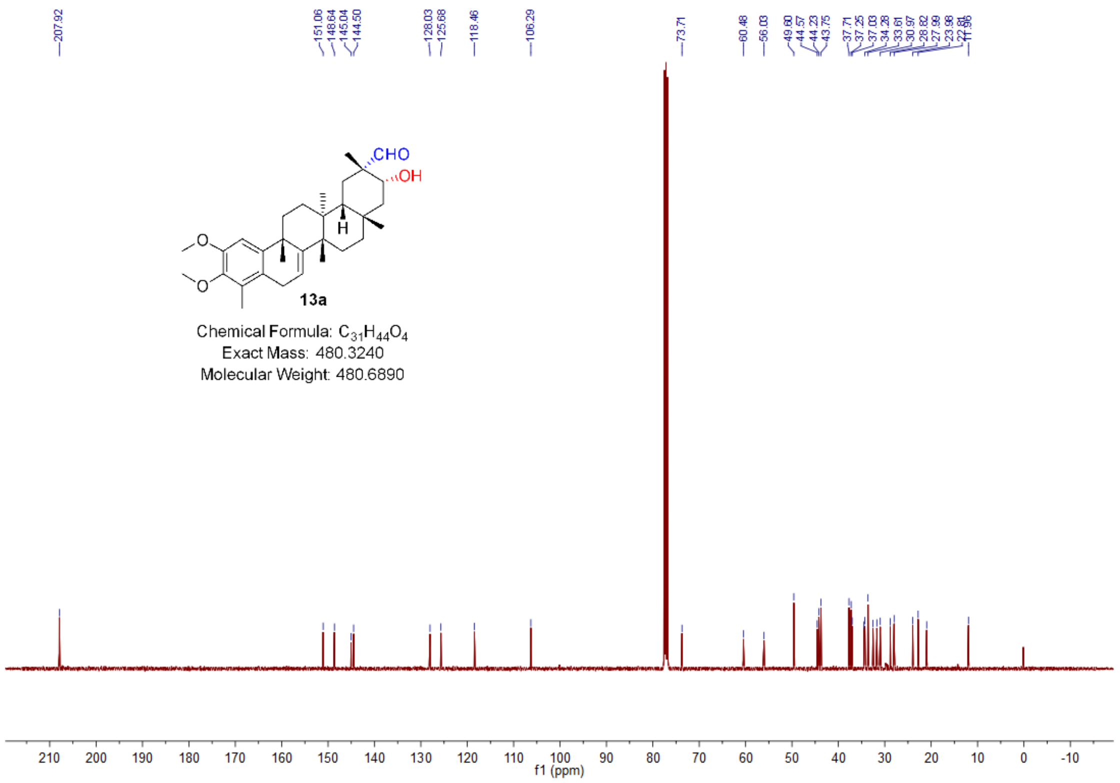

Supplementary Figure 87. [13]**C-NMR spectrum of compound 13-a (101 MHz, $CDCl_3$, 25 ºC)**

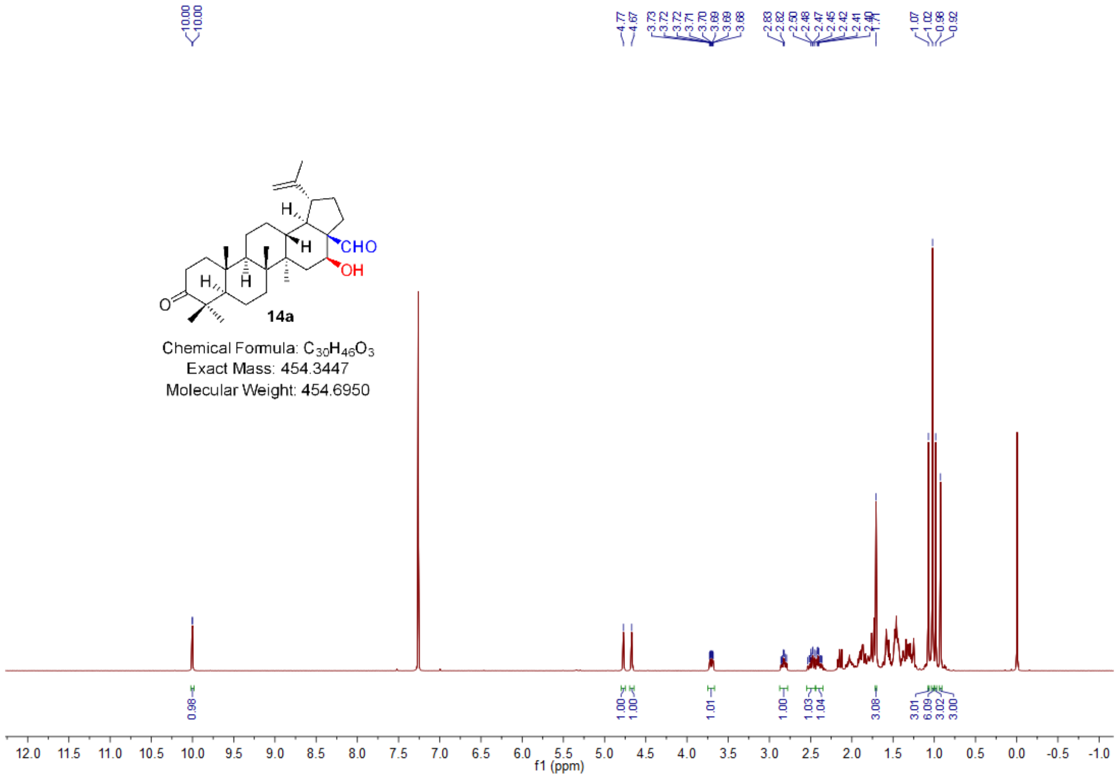

Supplementary Figure 88. [1]**H-NMR spectrum of compound 14-a (400 MHz, $CDCl_3$, 25 ºC)**

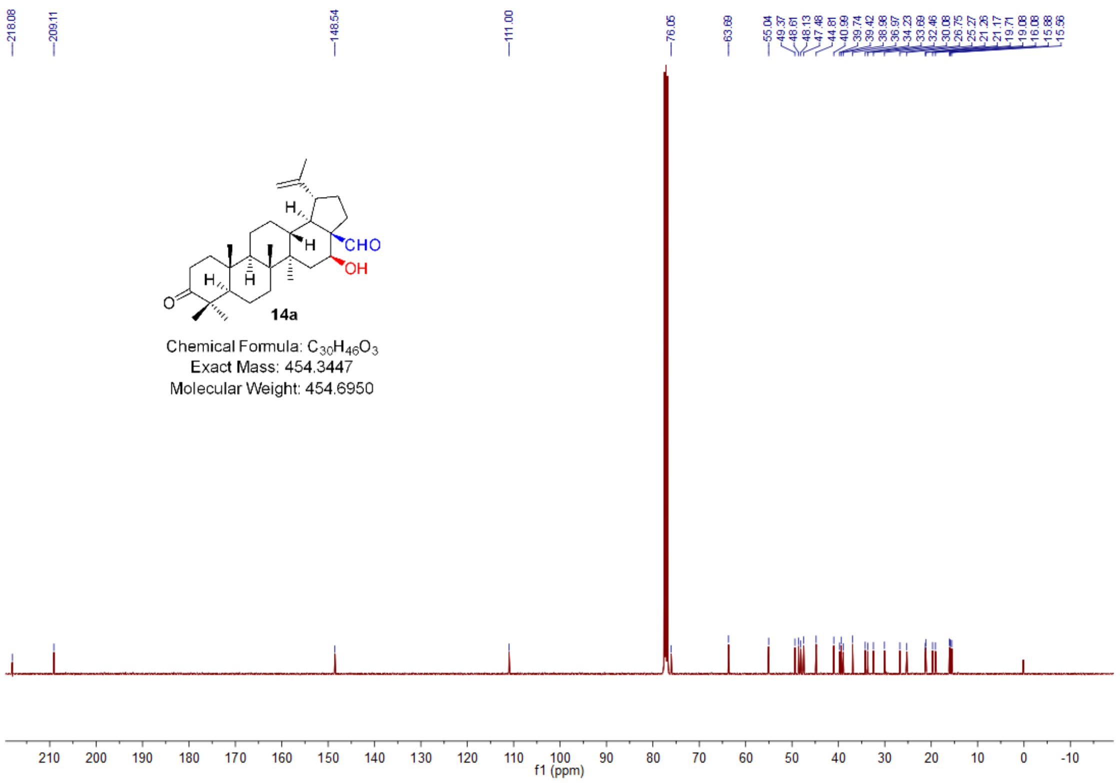

Supplementary Figure 89. **^{13}C-NMR spectrum of compound 14-a (101 MHz, $CDCl_3$, 25 °C)**

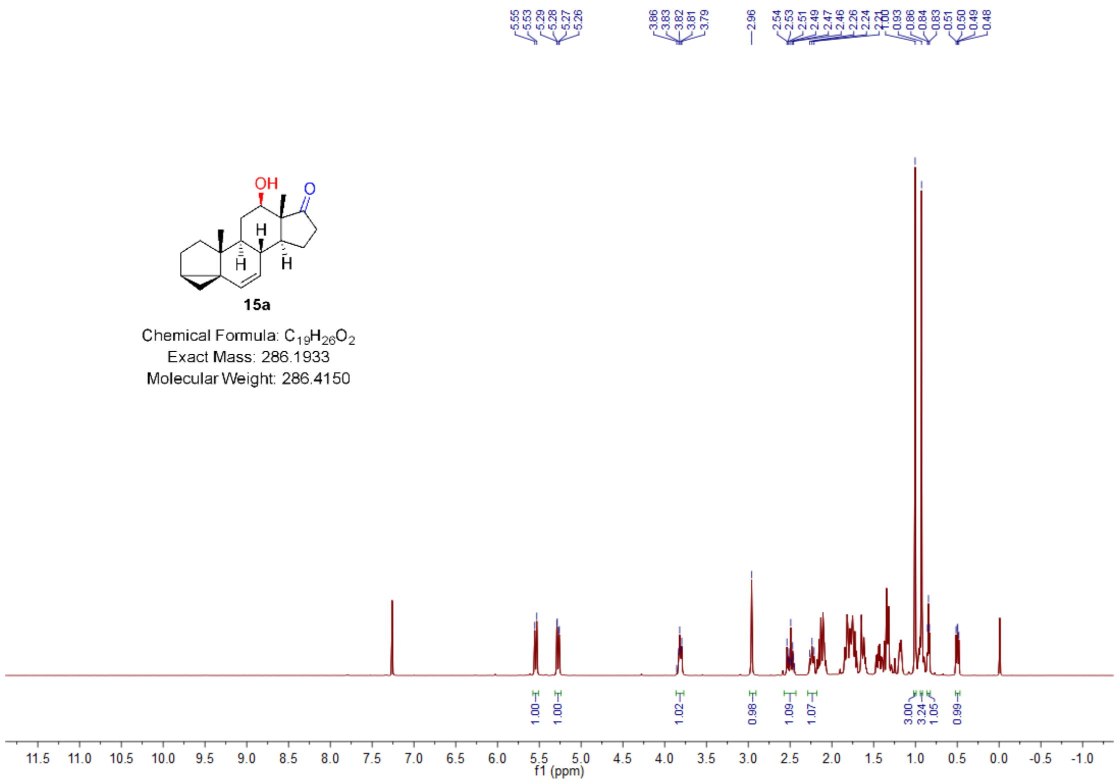

Supplementary Figure 90. **^{1}H-NMR spectrum of compound 15-a (400 MHz, $CDCl_3$, 25 °C)**

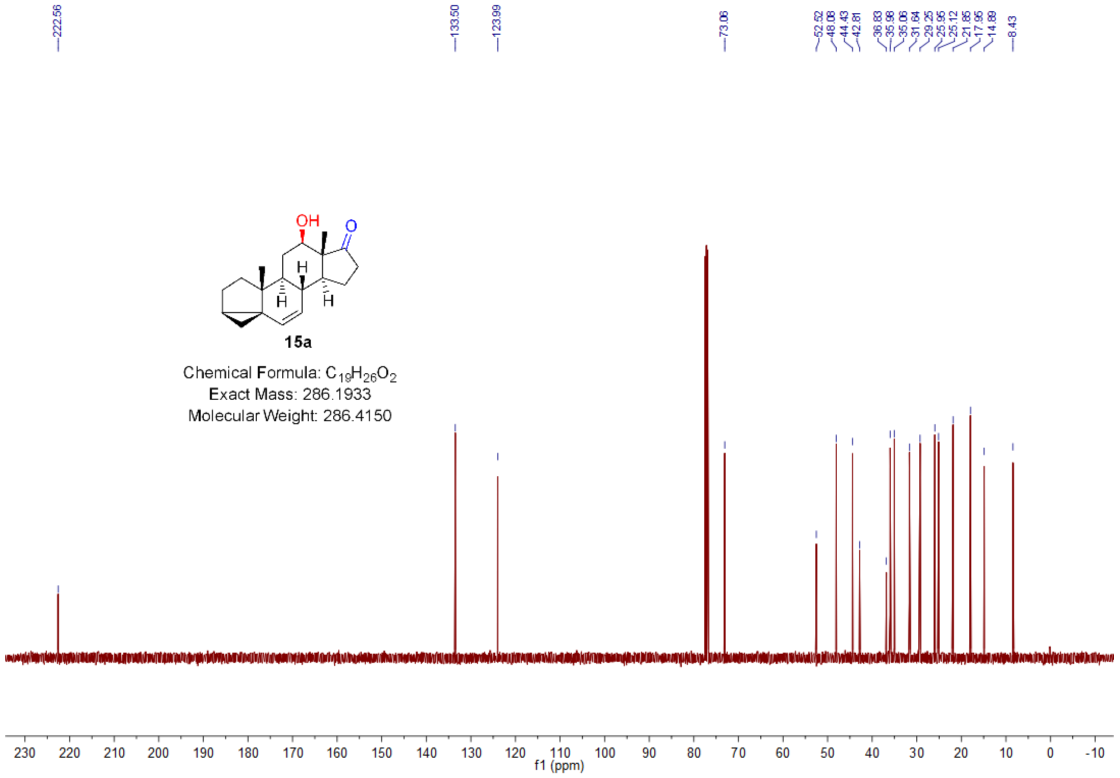

Supplementary Figure 91. ^{13}C-NMR spectrum of compound 15-a (126 MHz, $CDCl_3$, 25 °C)

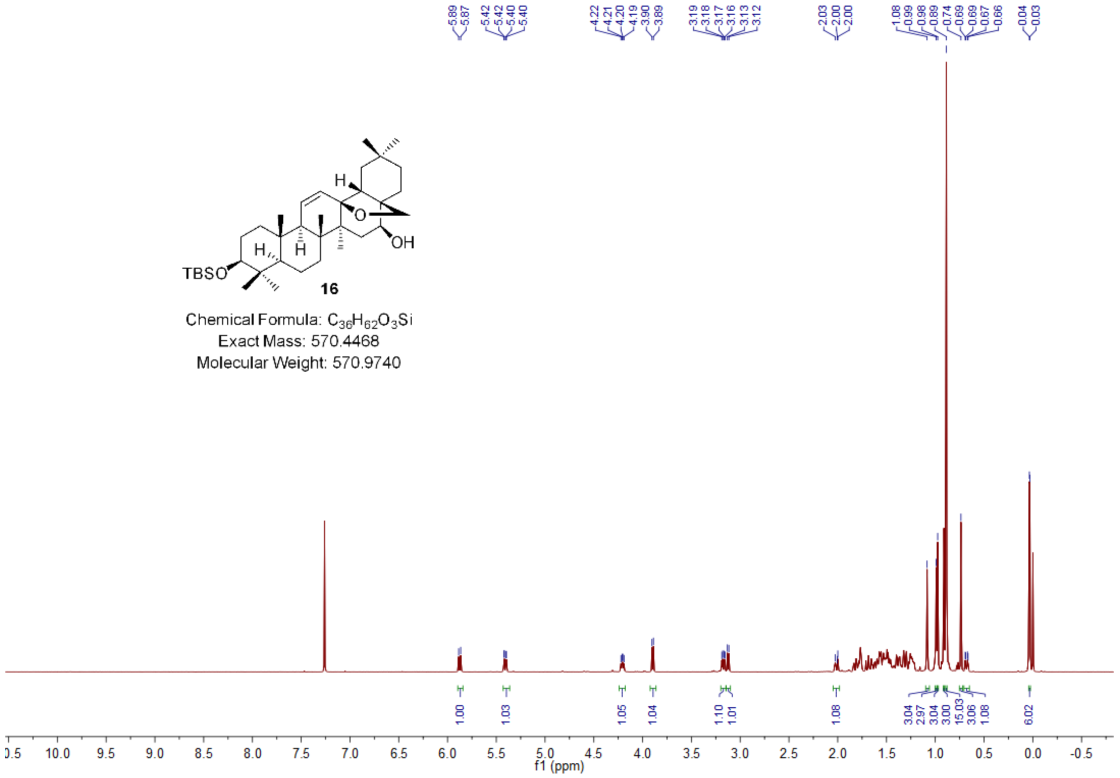

Supplementary Figure 92. ^{1}H-NMR spectrum of compound 16 (500 MHz, $CDCl_3$, 25 °C)

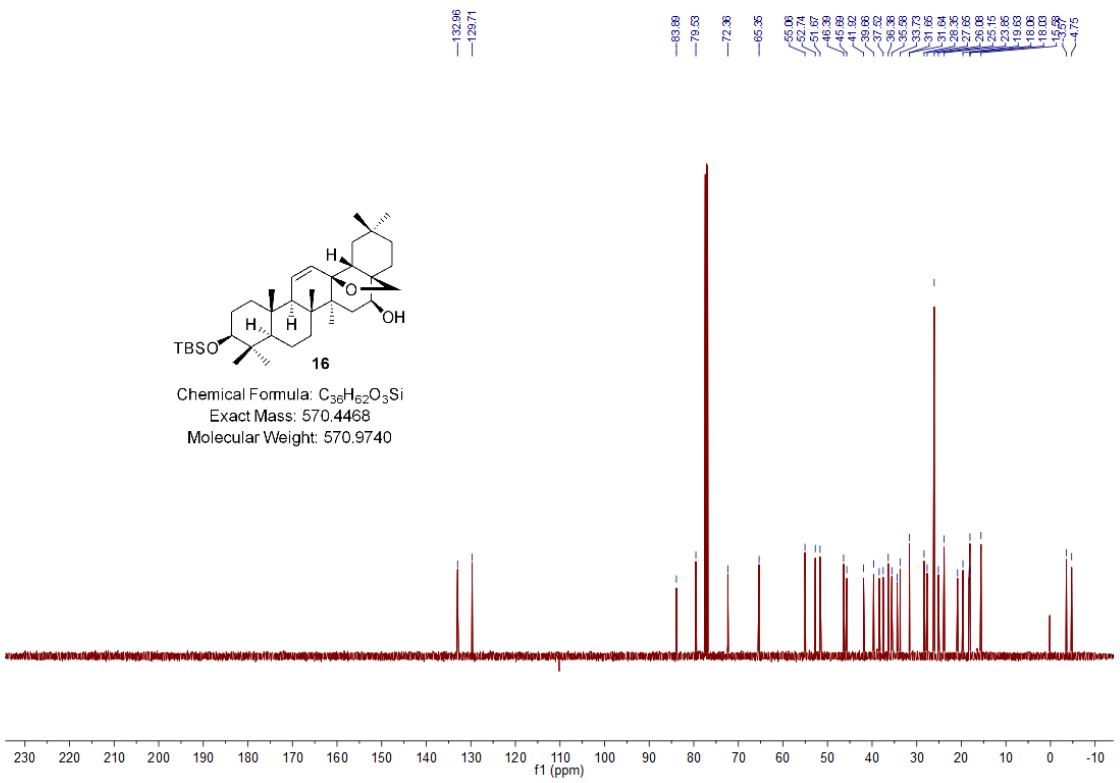

Supplementary Figure 93. ^{13}C-NMR spectrum of compound 16 (126 MHz, $CDCl_3$, 25 °C)

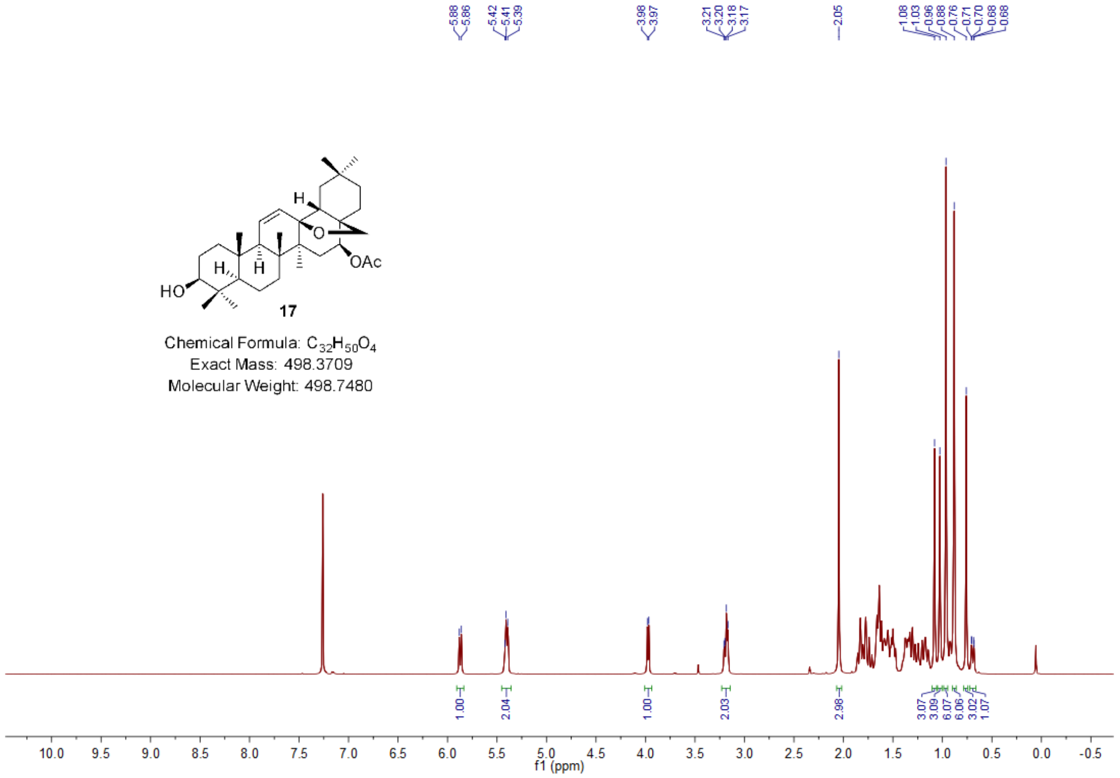

Supplementary Figure 94. ^{1}H-NMR spectrum of compound 17 (500 MHz, $CDCl_3$, 25 °C)

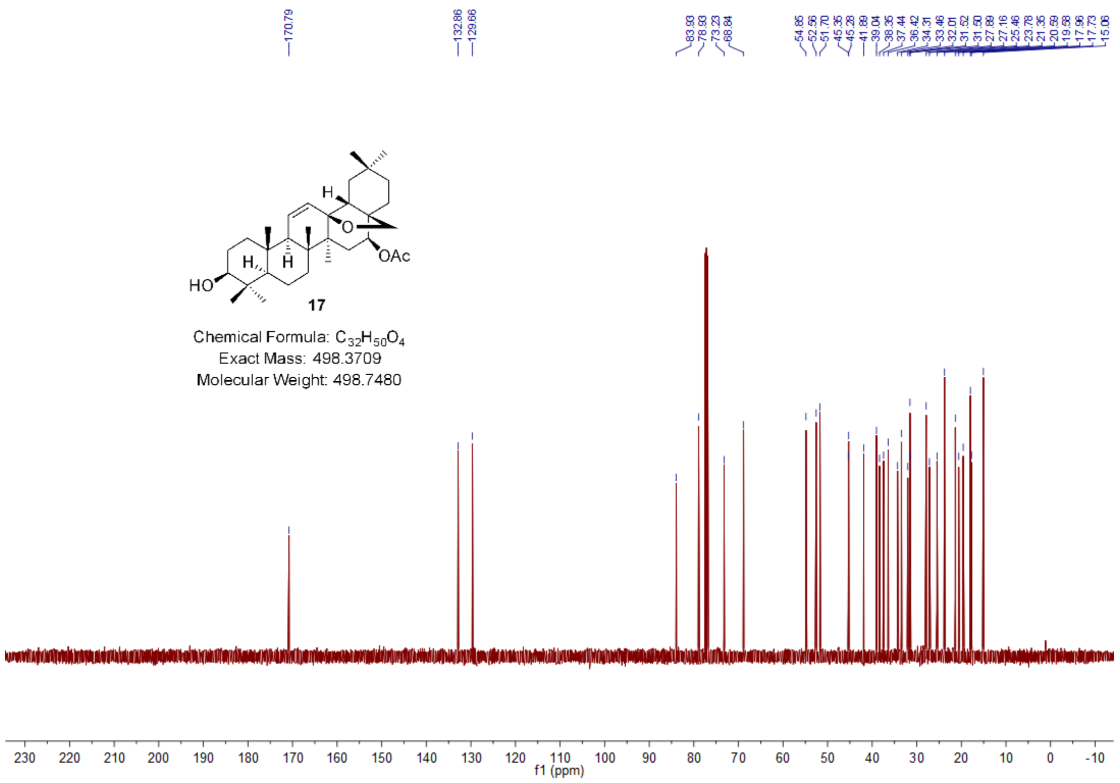

Supplementary Figure 95. ^{13}C-NMR spectrum of compound 17 (126 MHz, $CDCl_3$, 25 °C)

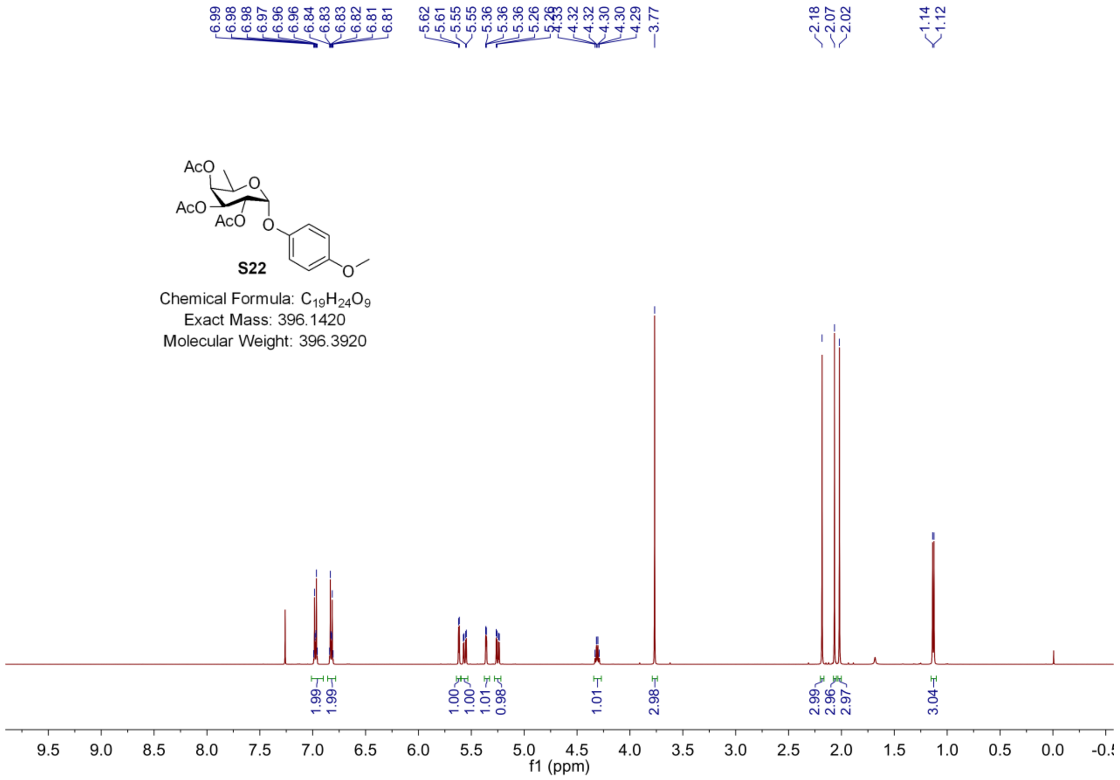

Supplementary Figure 96. ^{1}H-NMR spectrum of compound S22 (500 MHz, $CDCl_3$, 25 °C)

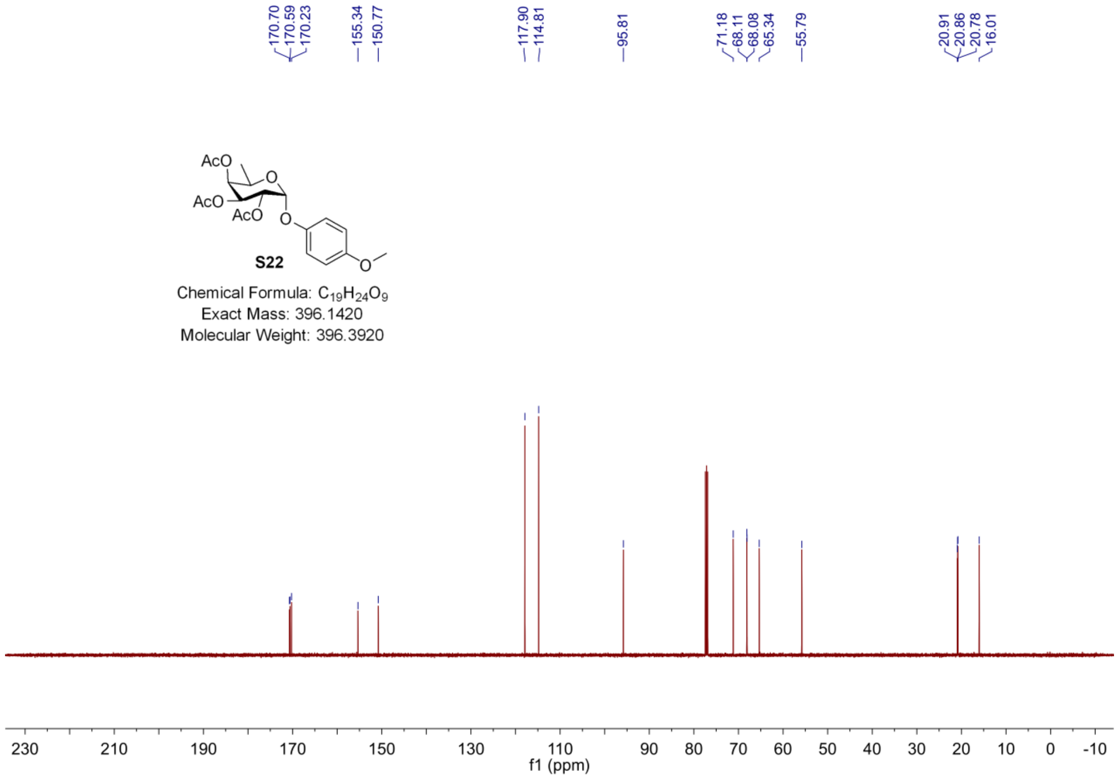

Supplementary Figure 97. ^{13}**C-NMR spectrum of compound S22 (126 MHz, CDCl$_3$, 25 °C)**

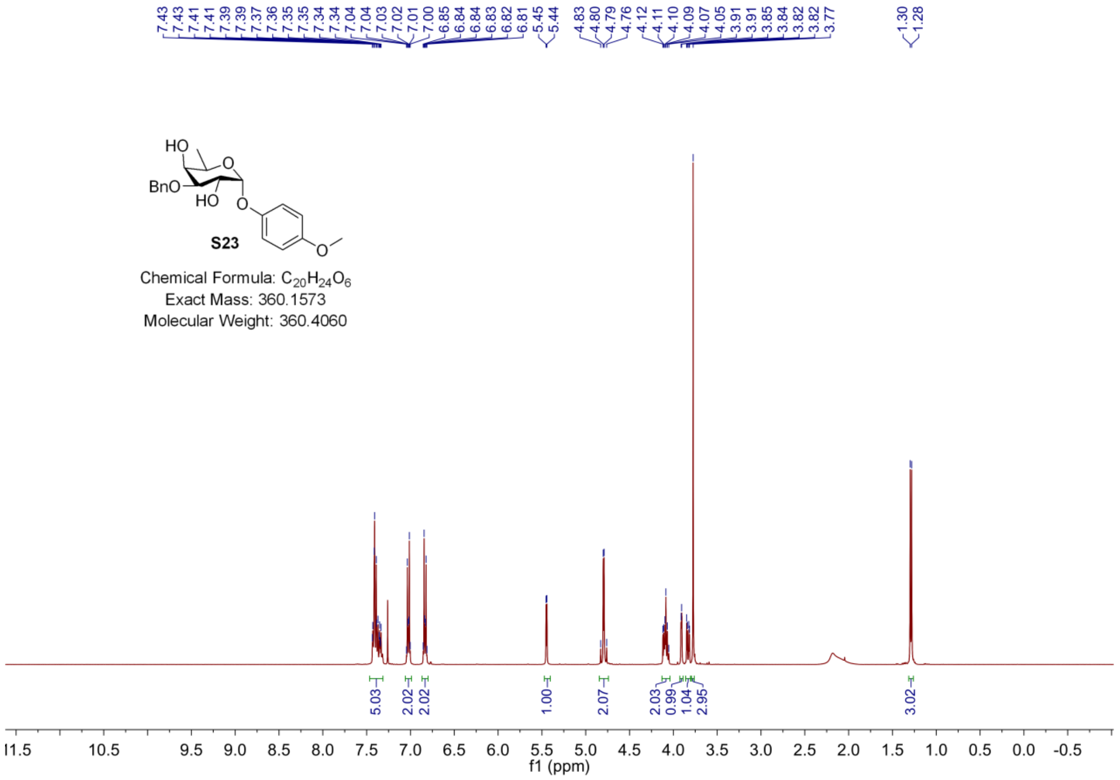

Supplementary Figure 98. ^{1}**H-NMR spectrum of compound S23 (400 MHz, CDCl$_3$, 25 °C)**

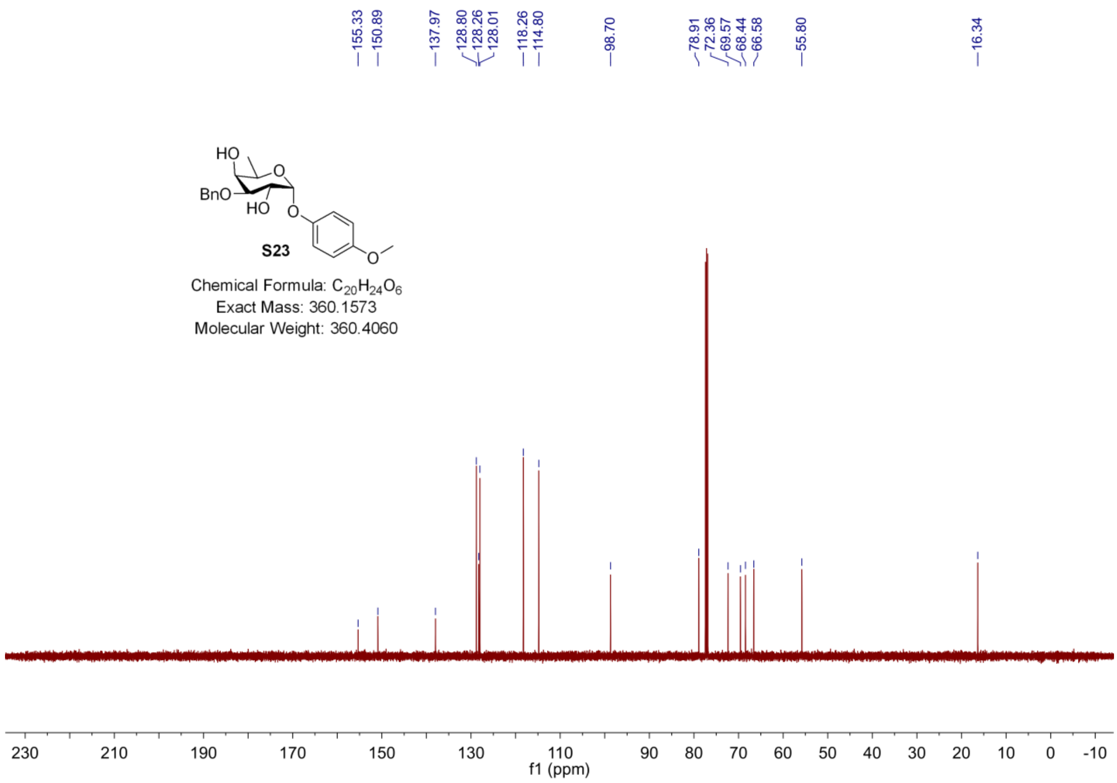

Supplementary Figure 99. **^{13}C-NMR spectrum of compound S23 (126 MHz, $CDCl_3$, 25 ºC)**

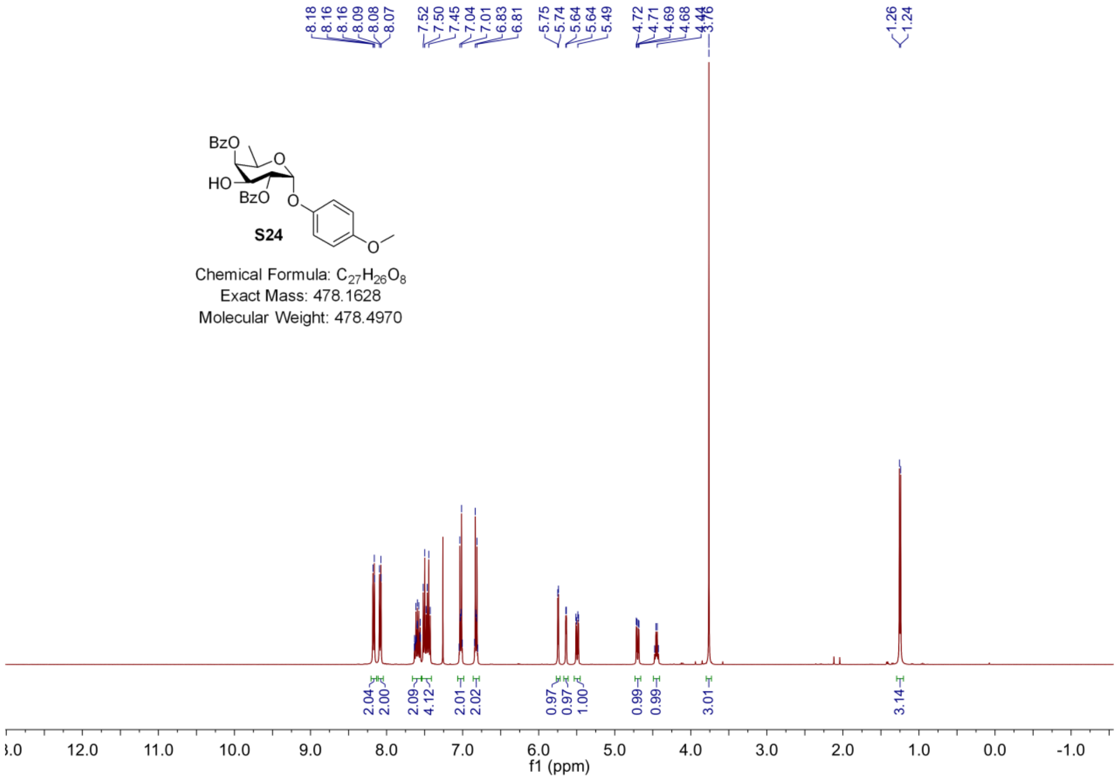

Supplementary Figure 100. **^{1}H-NMR spectrum of compound S24 (400 MHz, $CDCl_3$, 25 ºC)**

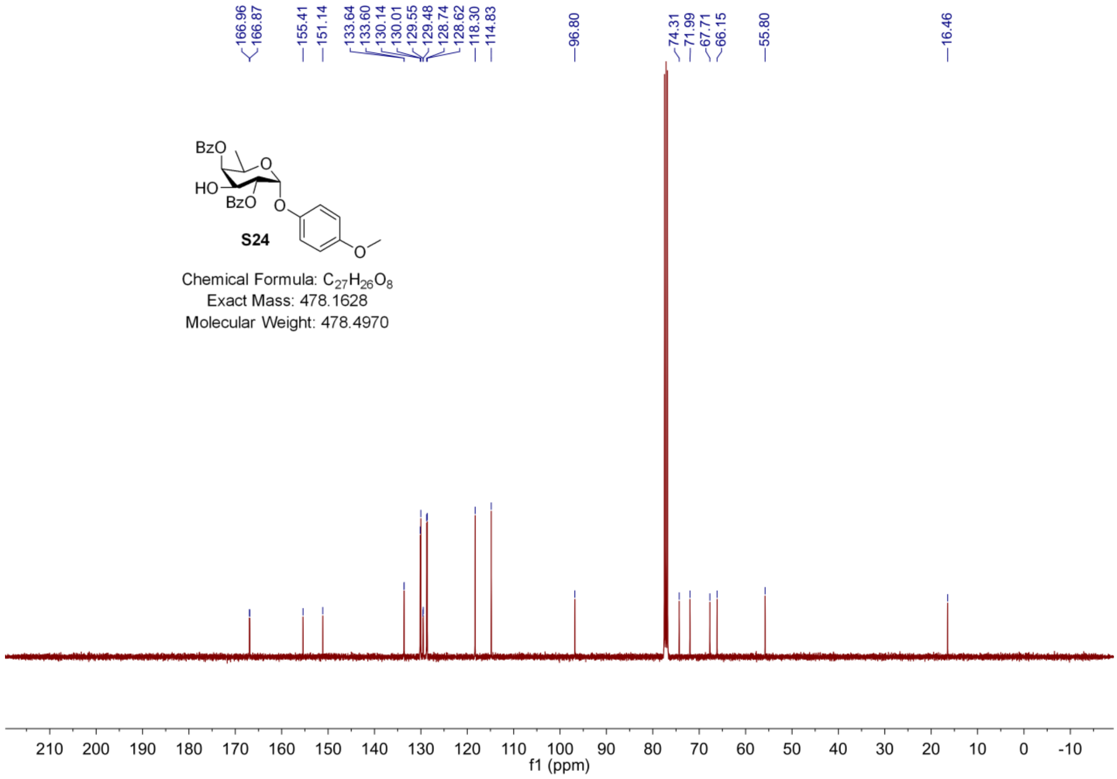

Supplementary Figure 101. ^{13}C-NMR spectrum of compound S24 (101 MHz, $CDCl_3$, 25 ºC)

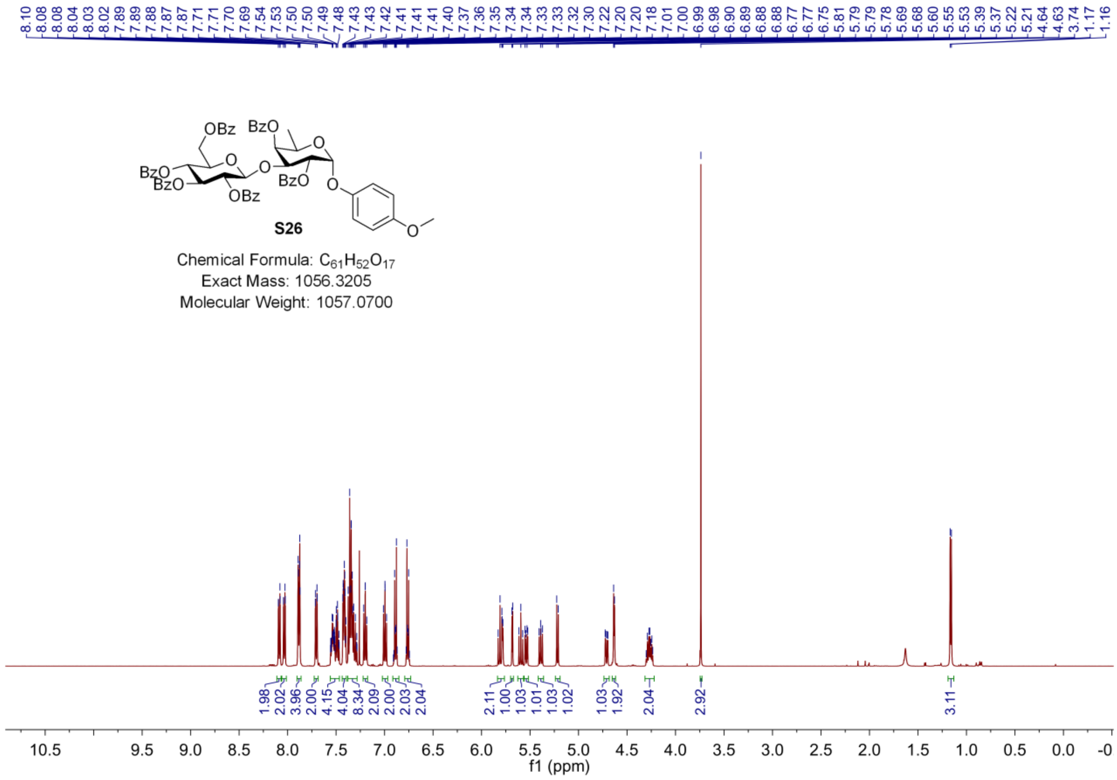

Supplementary Figure 102. ^{1}H-NMR spectrum of compound S26 (500 MHz, $CDCl_3$, 25 ºC)

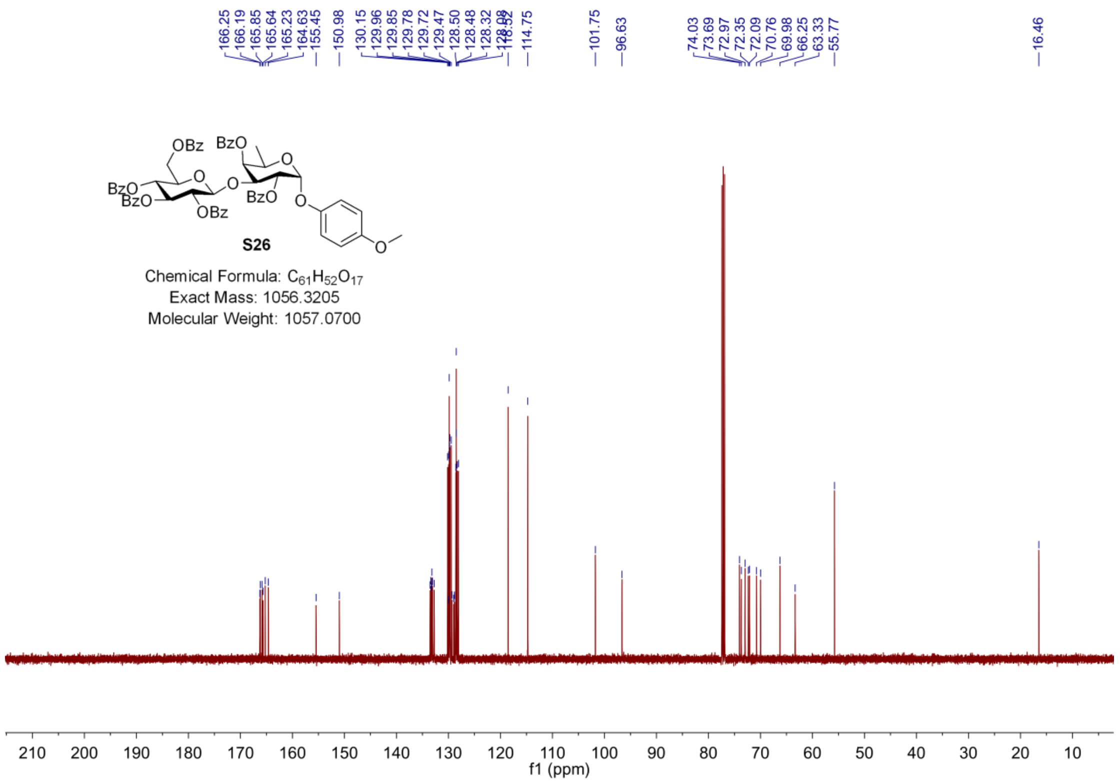

Supplementary Figure 103. ^{13}C-NMR spectrum of compound S26 (126 MHz, $CDCl_3$, 25 °C)

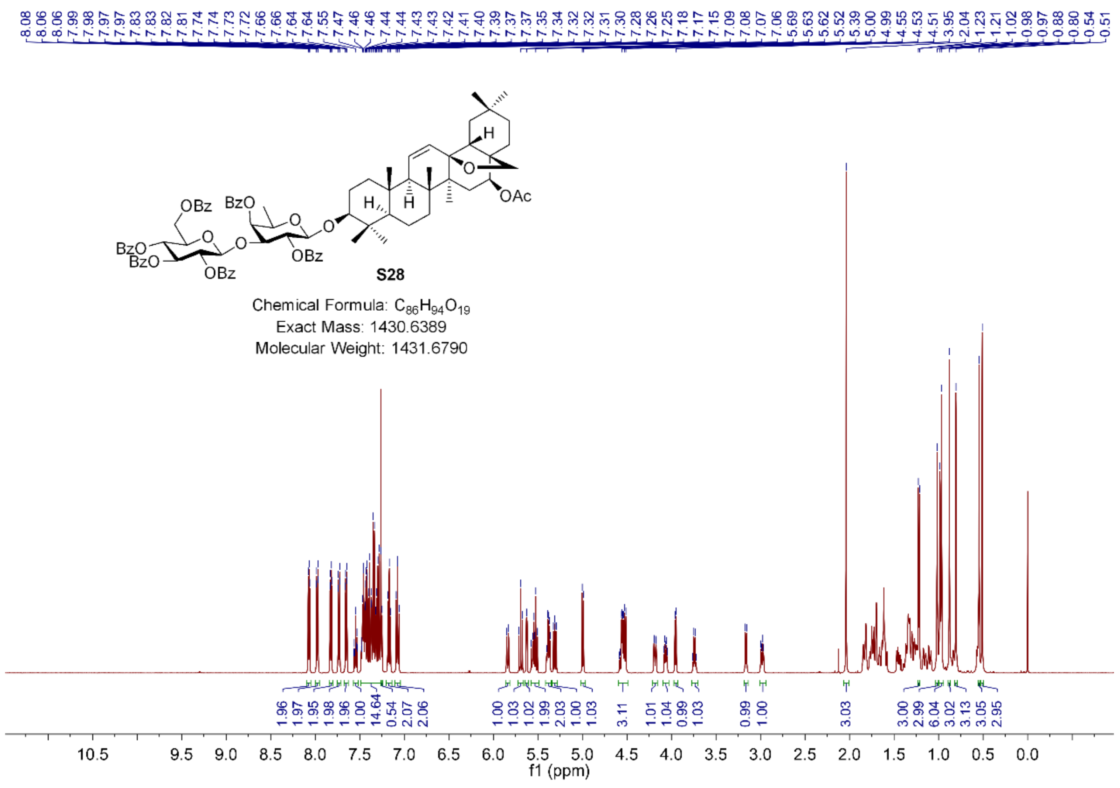

Supplementary Figure 104. ^{1}H-NMR spectrum of compound S28 (500 MHz, $CDCl_3$, 25 °C)

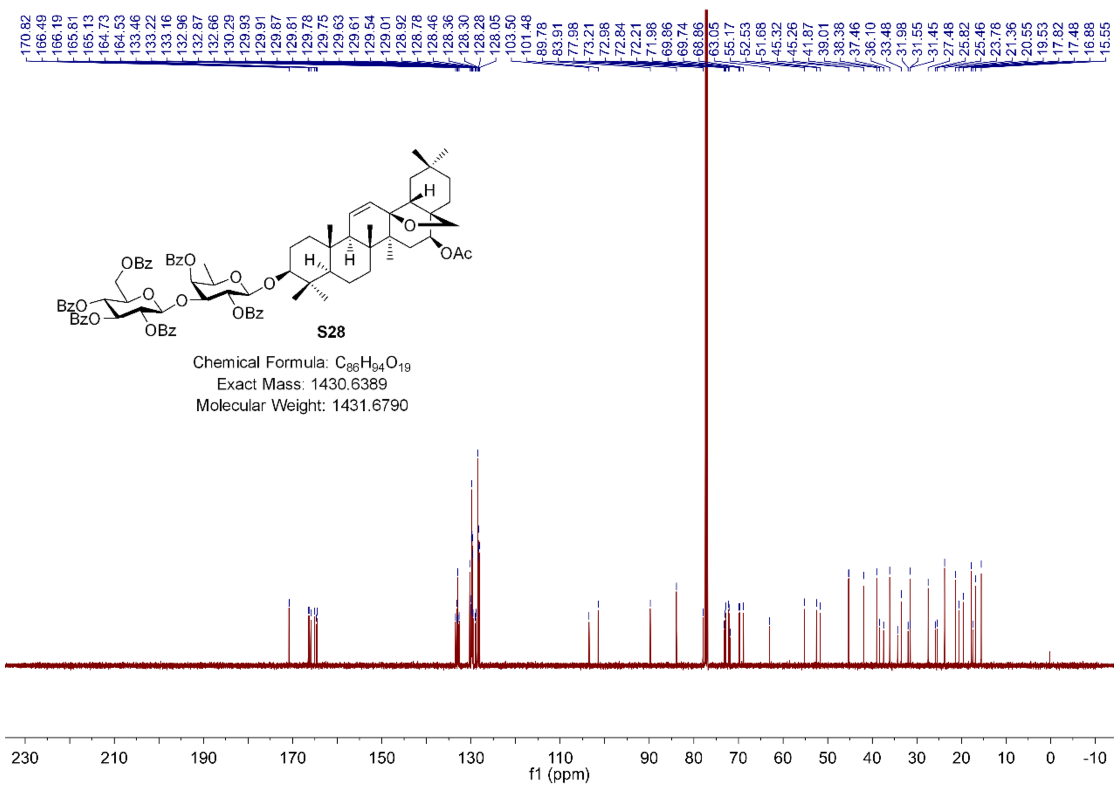

Supplementary Figure 105. ^{13}C-NMR spectrum of compound S28 (126 MHz, $CDCl_3$, 25 ^{o}C)

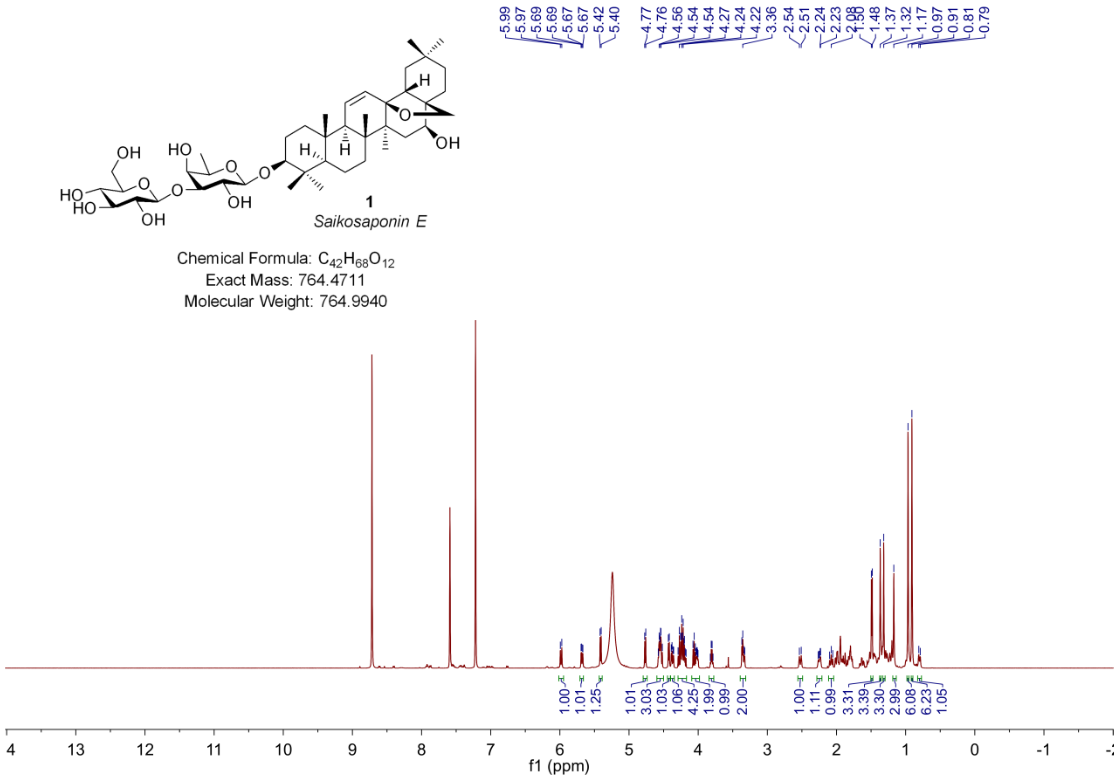

Supplementary Figure 106. ^{1}H-NMR spectrum of compound 1 (500 MHz, pyridine-d5, 25 ^{o}C)

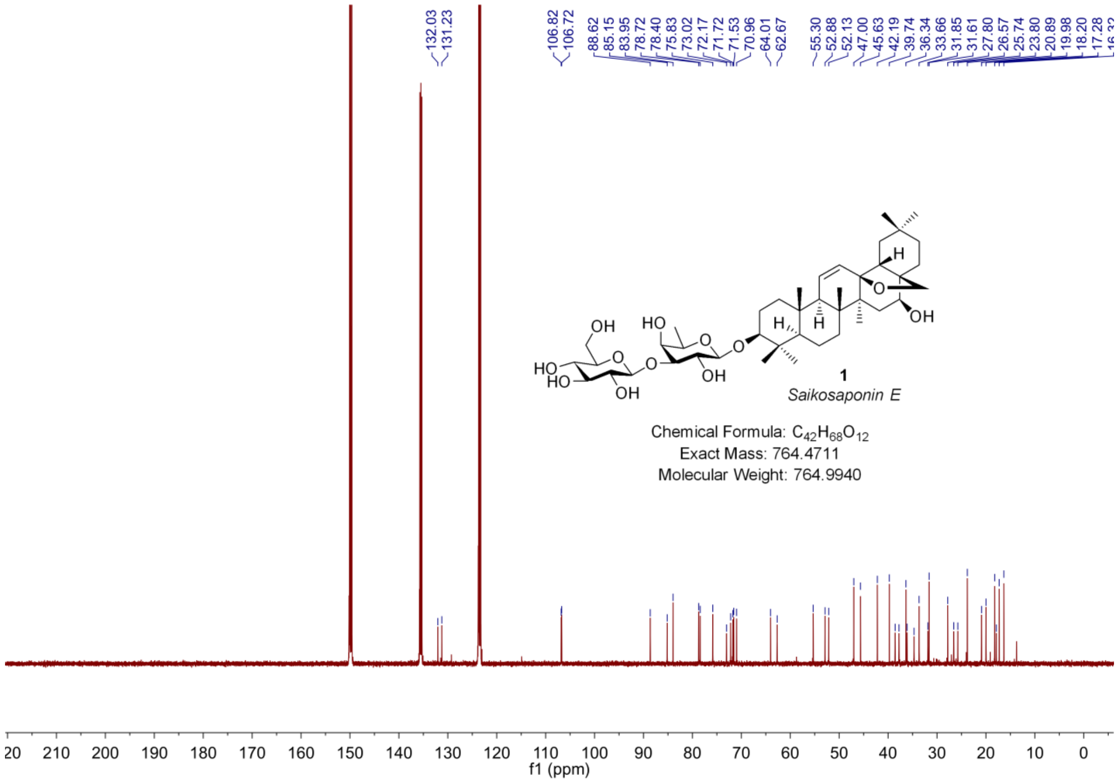

Supplementary Figure 107. ^{13}C-NMR spectrum of compound 1 (126 MHz, pyridine-d5, 25 ºC)

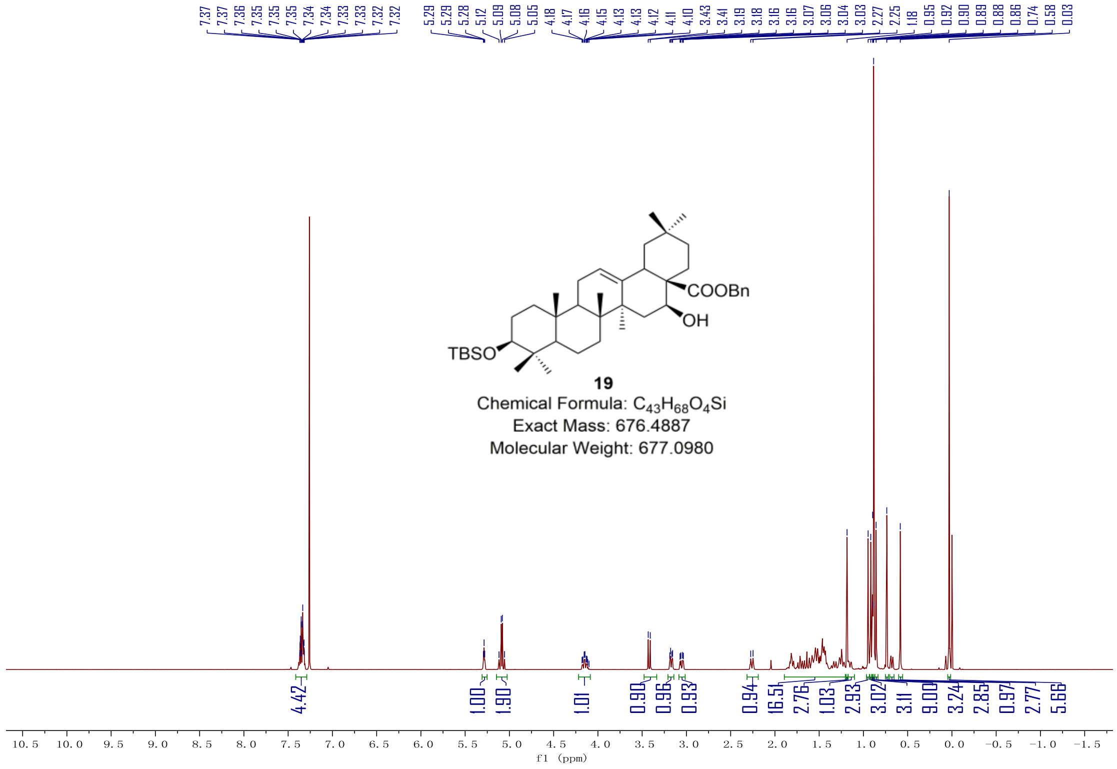

Supplementary Figure 108. ^{1}H-NMR spectrum of compound 19 (500 MHz, $CDCl_3$, 25 ºC)

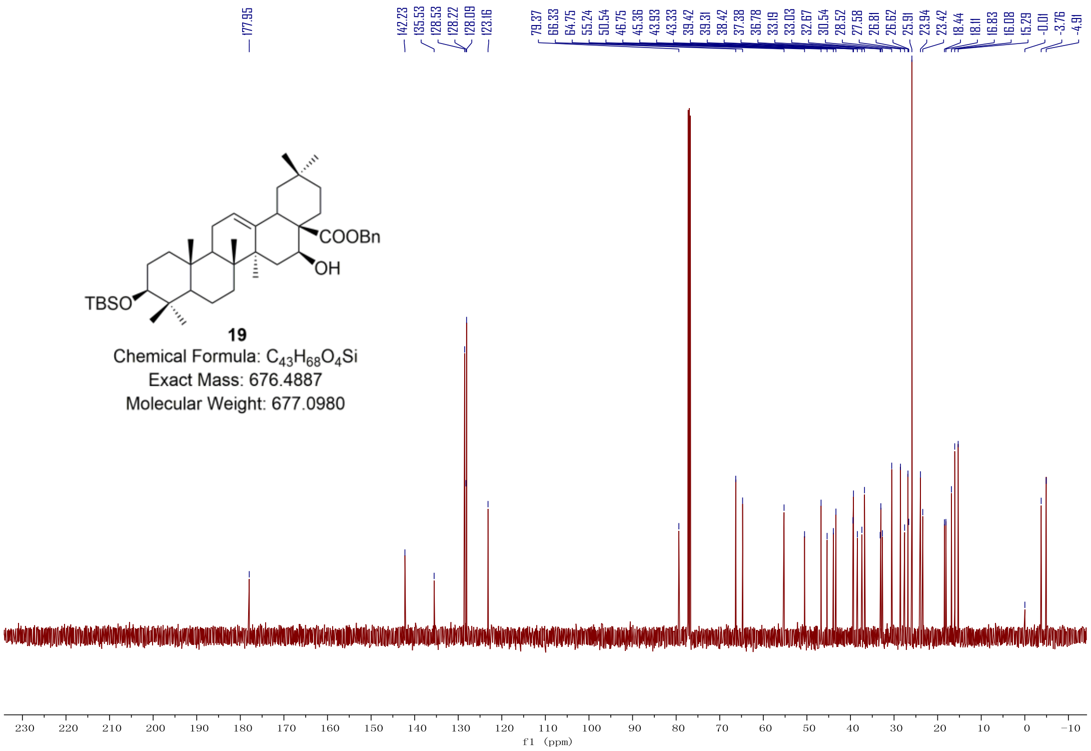

Supplementary Figure 109. ^{13}C-NMR spectrum of compound 19 (126 MHz, $CDCl_3$, 25 ^{o}C)

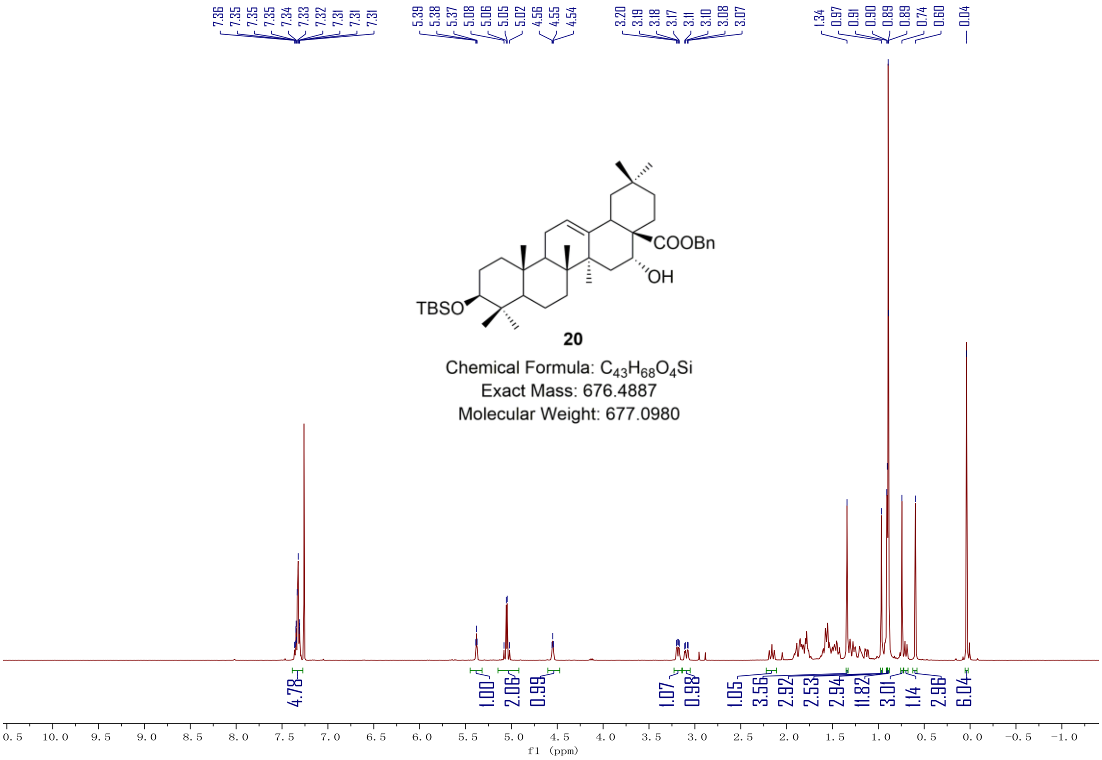

Supplementary Figure 110. ^{1}H-NMR spectrum of compound 20 (500 MHz, $CDCl_3$, 25 ^{o}C)

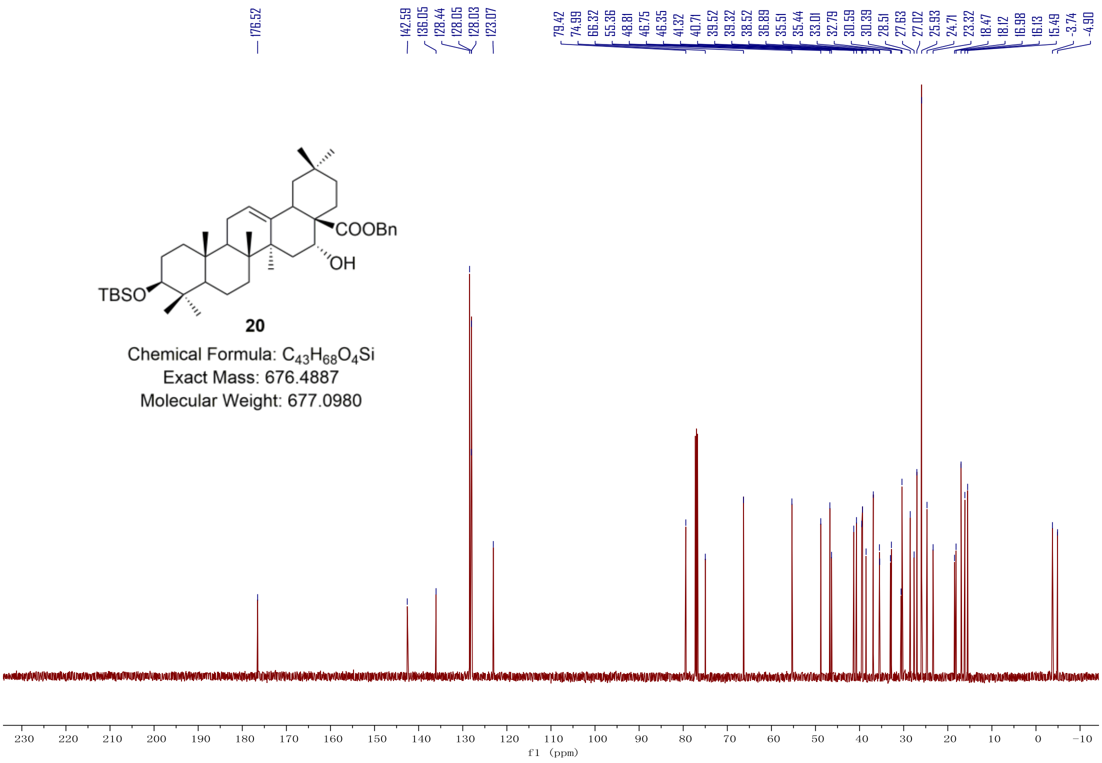

Supplementary Figure 111. ^{13}C-NMR spectrum of compound 20 (126 MHz, $CDCl_3$, 25 °C)

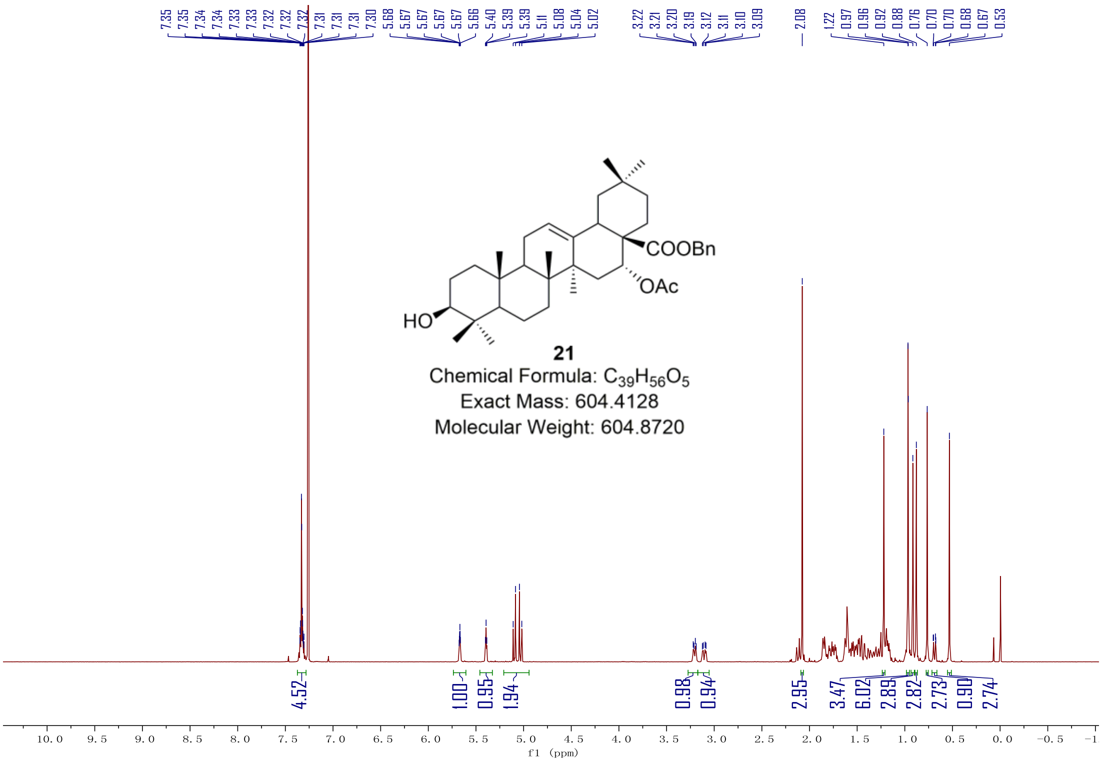

Supplementary Figure 112. ^{1}H-NMR spectrum of compound 21 (500 MHz, $CDCl_3$, 25 °C)

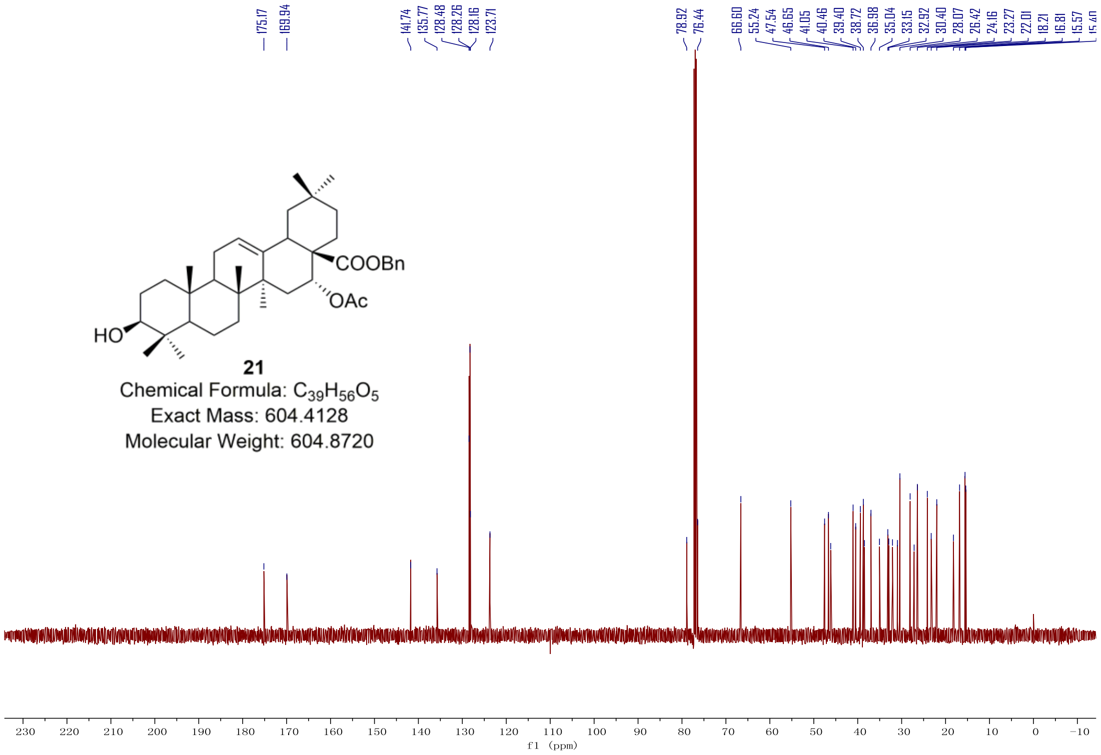

Supplementary Figure 113. ^{13}C-NMR spectrum of compound 21 (126 MHz, $CDCl_3$, 25 °C)

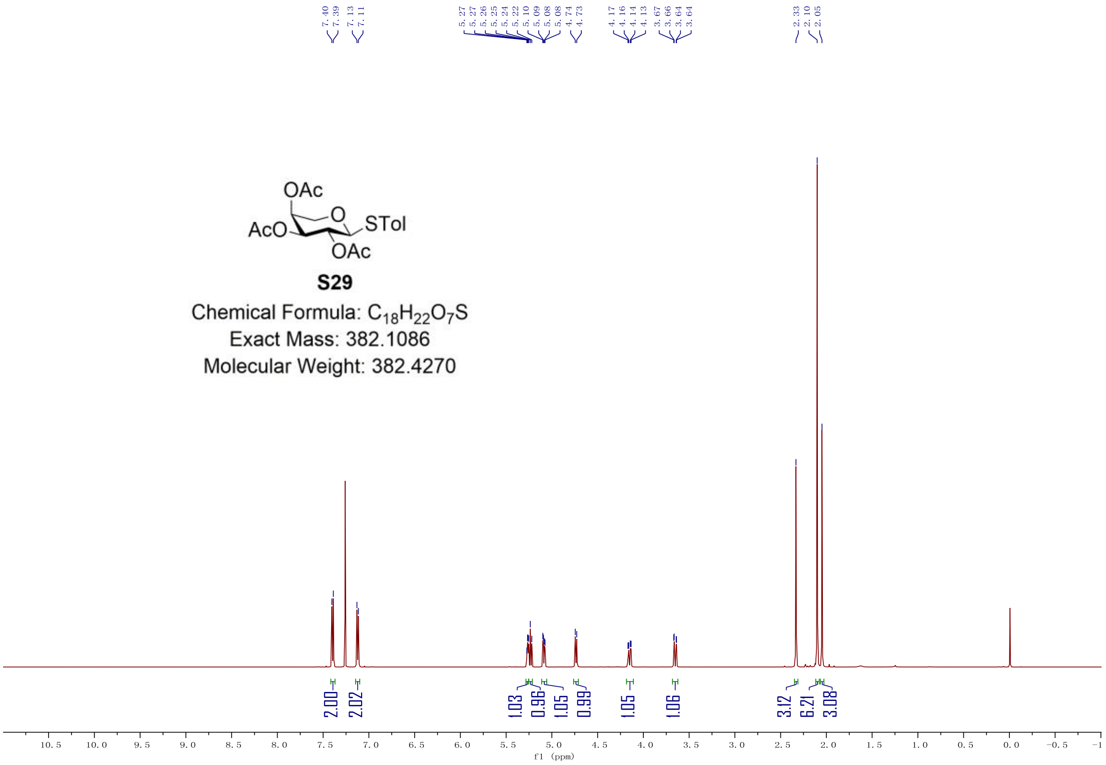

Supplementary Figure 114. ^{1}H-NMR spectrum of compound S29 (500 MHz, $CDCl_3$, 25 °C)

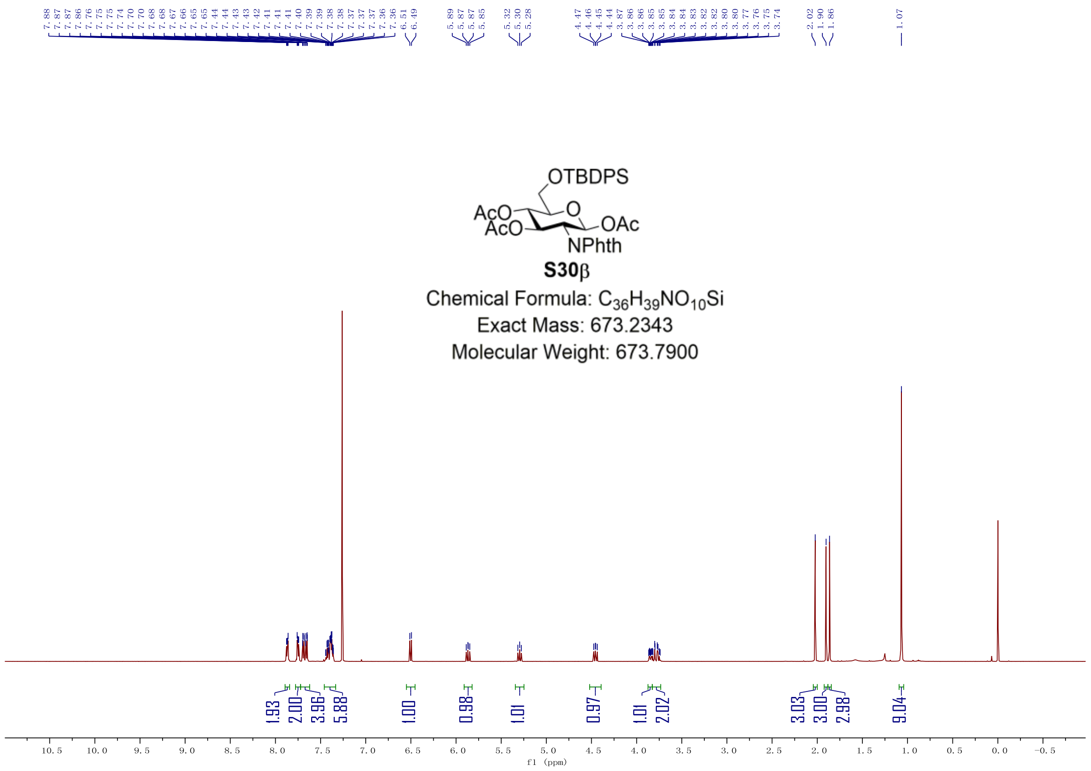

Supplementary Figure 115. ^{1}**H-NMR spectrum of compound S30β (500 MHz,** $CDCl_3$**, 25 °C)**

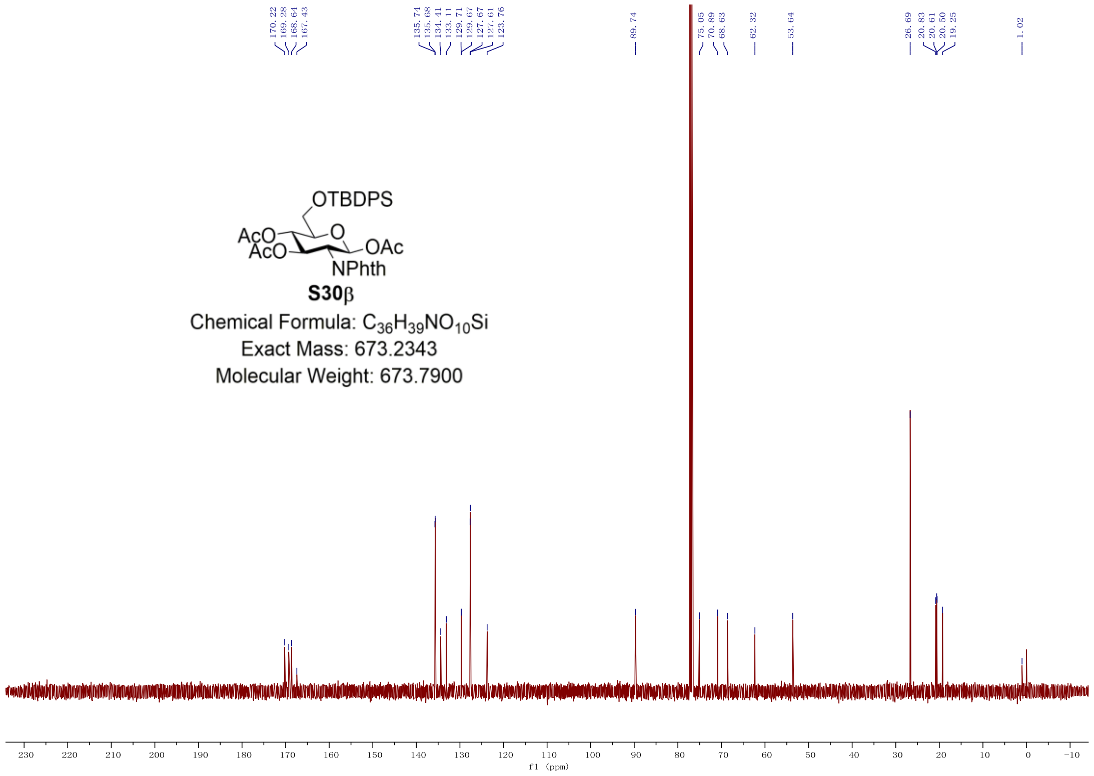

Supplementary Figure 116. ^{13}**C-NMR spectrum of compound S30β (126 MHz,** $CDCl_3$**, 25 °C)**

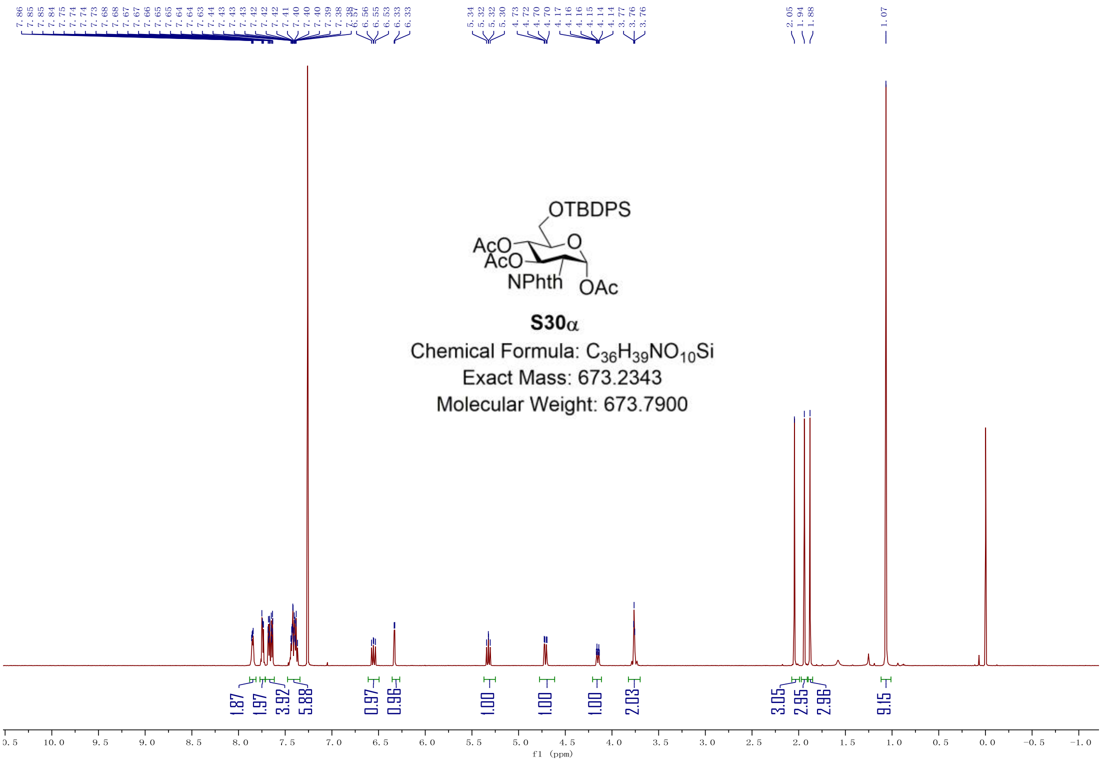

Supplementary Figure 117. ^{1}**H-NMR spectrum of compound S30α (500 MHz, CDCl**$_3$**, 25 °C)**

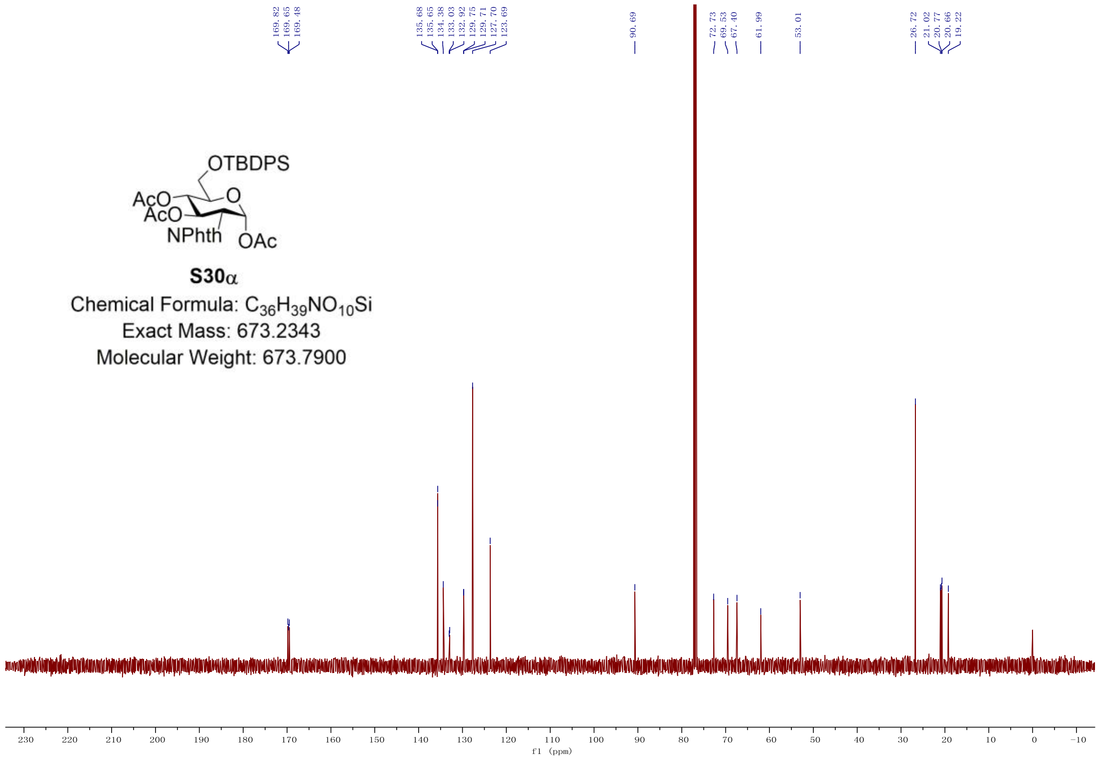

Supplementary Figure 118. ^{13}**C-NMR spectrum of compound S30α (126 MHz, CDCl**$_3$**, 25 °C)**

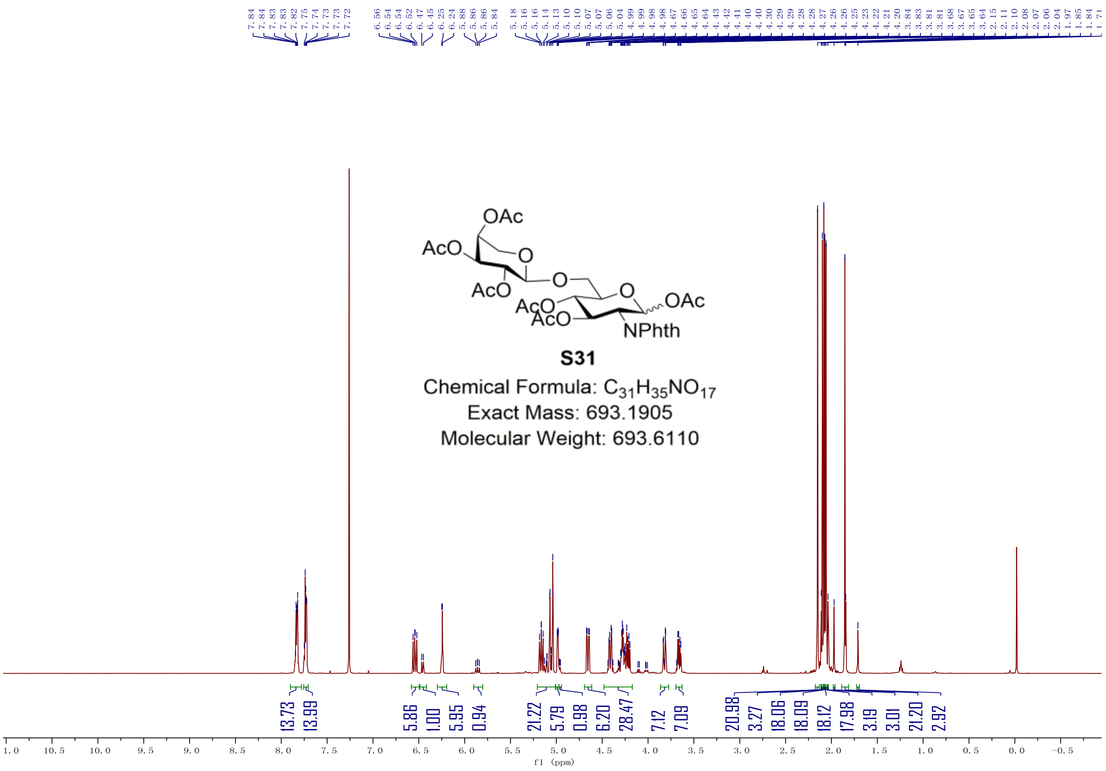

Supplementary Figure 119. ^{1}H-NMR spectrum of compound S31 (500 MHz, CDCl$_3$, 25 ^{o}C)

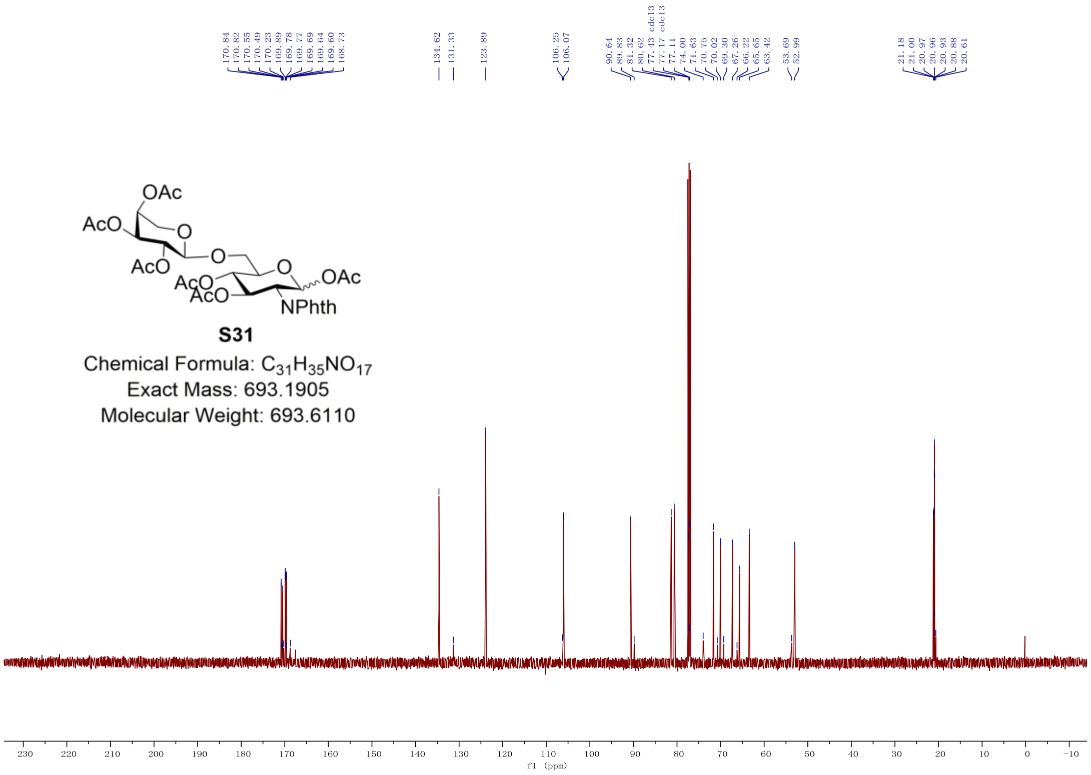

Supplementary Figure 120. ^{13}C-NMR spectrum of compound S31 (126 MHz, CDCl$_3$, 25 ^{o}C)

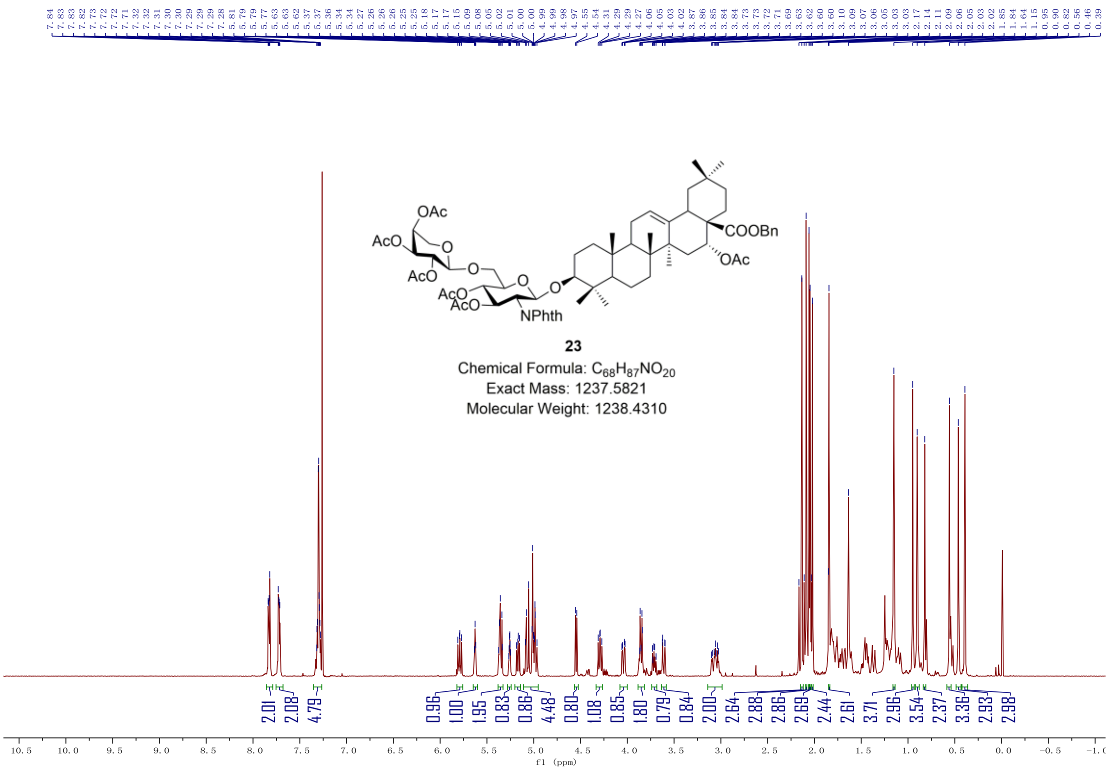

Supplementary Figure 121. ^{1}H-NMR spectrum of compound 23 (500 MHz, $CDCl_3$, 25 ºC)

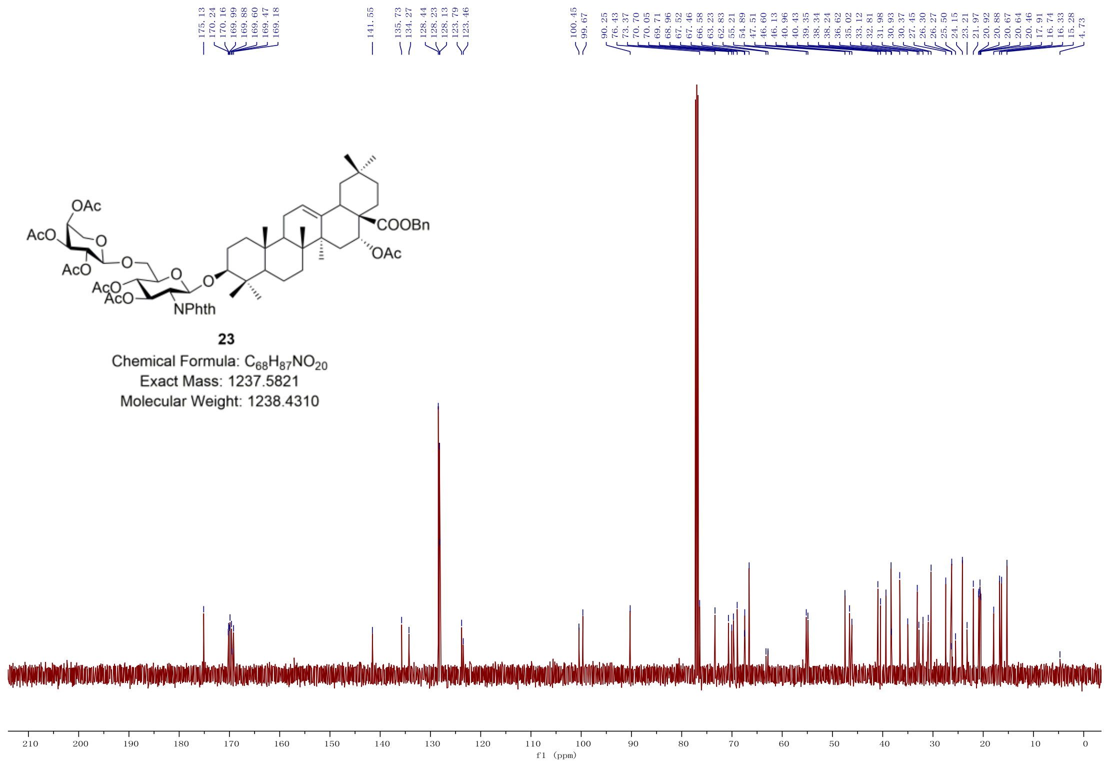

Supplementary Figure 122. ^{13}C-NMR spectrum of compound 23 (126 MHz, $CDCl_3$, 25 ºC)

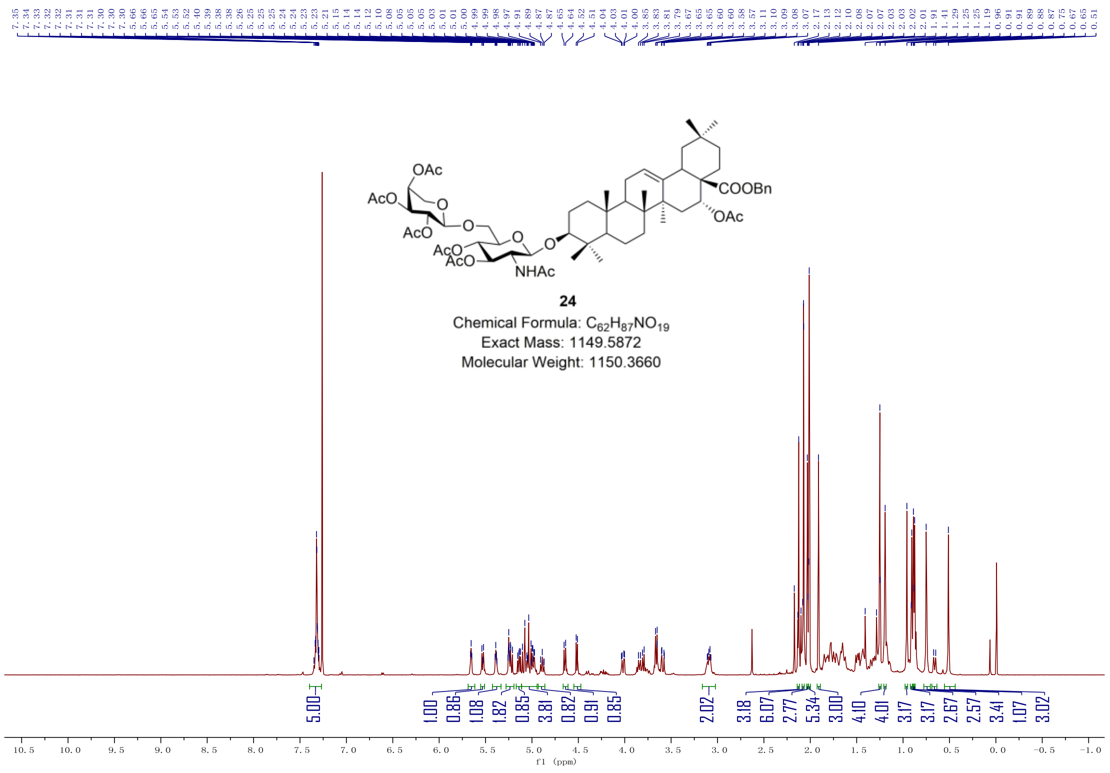

Supplementary Figure 123. ^{1}H-NMR spectrum of compound 24 (500 MHz, $CDCl_3$, 25 °C)

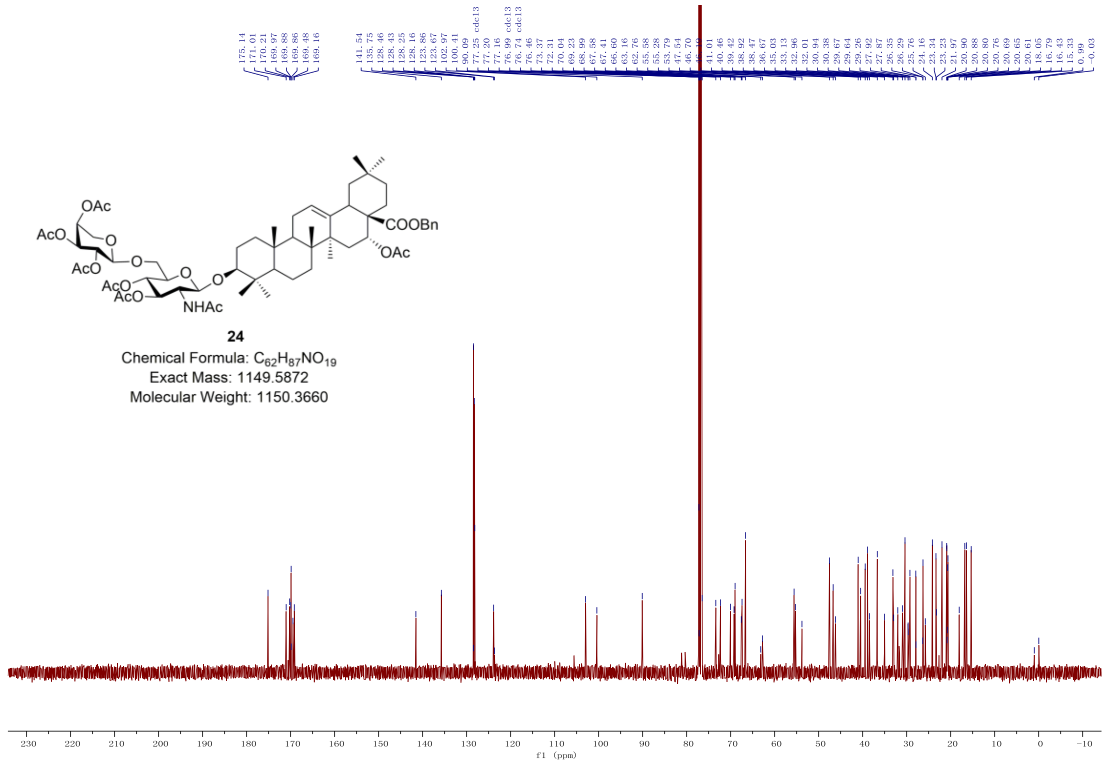

Supplementary Figure 124. ^{13}C-NMR spectrum of compound 24 (126 MHz, $CDCl_3$, 25 °C)

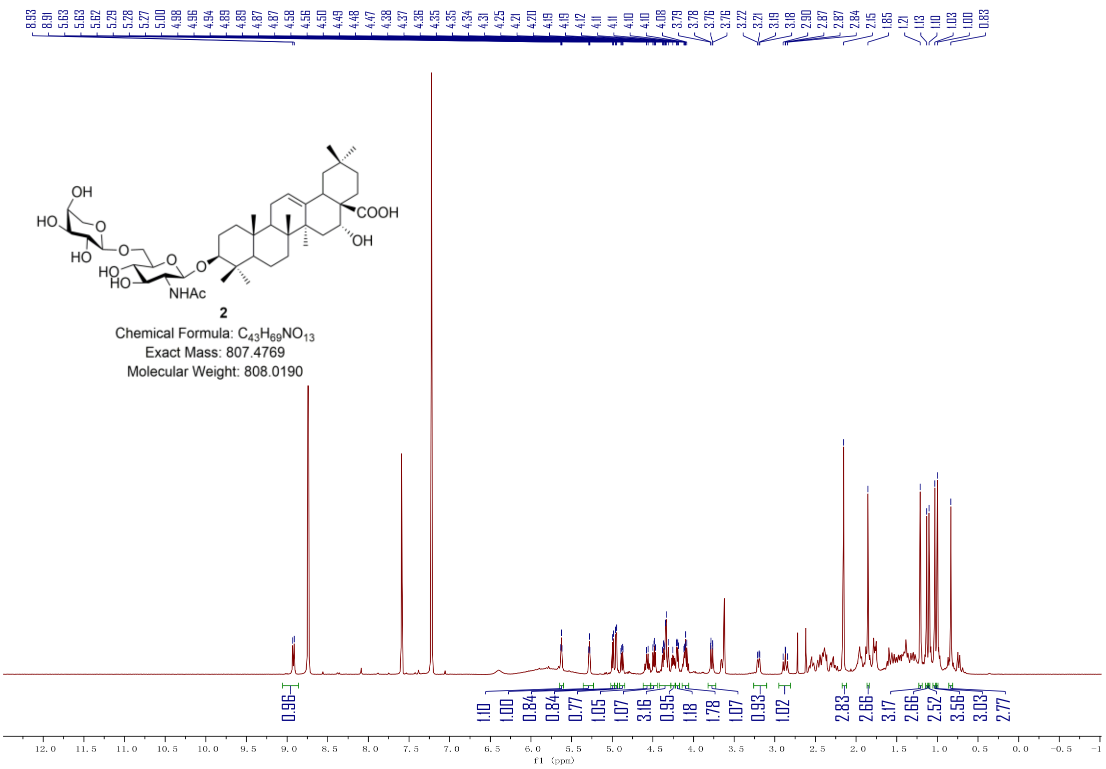

Supplementary Figure 125. ^{1}H-NMR spectrum of compound 2 (500 MHz, pyridine-d5, 25 ºC)

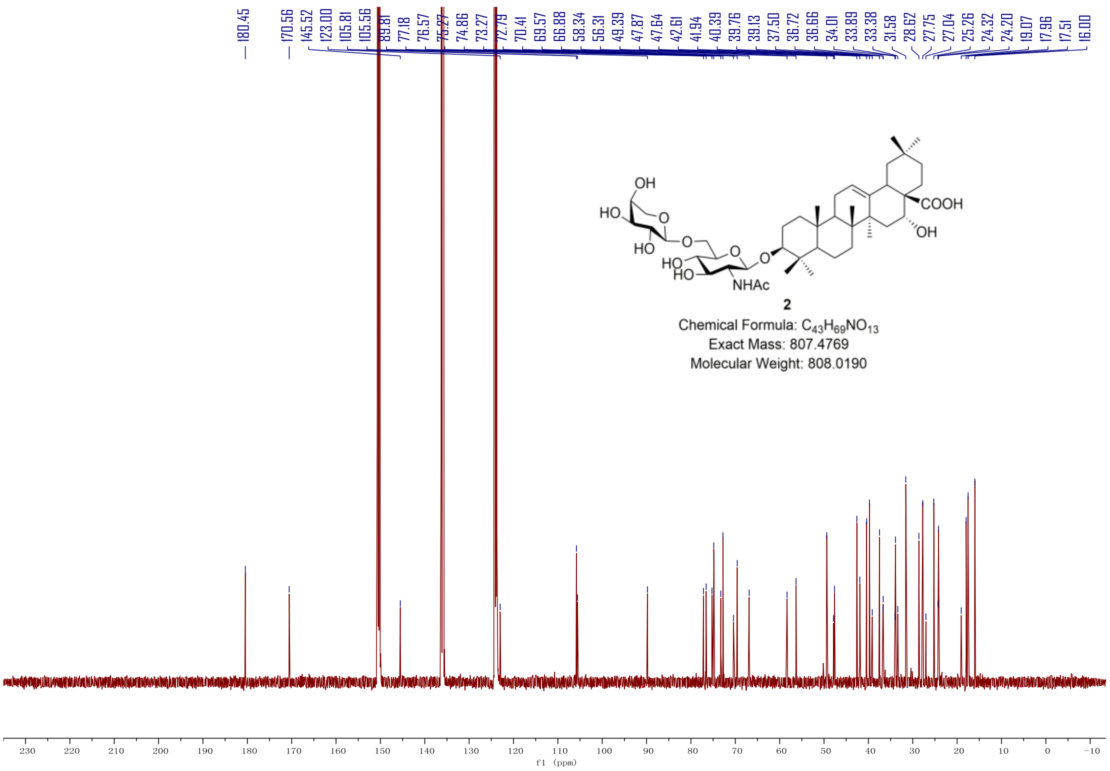

Supplementary Figure 126. ^{13}C-NMR spectrum of compound 2 (126 MHz, pyridine-d5, 25 ºC)

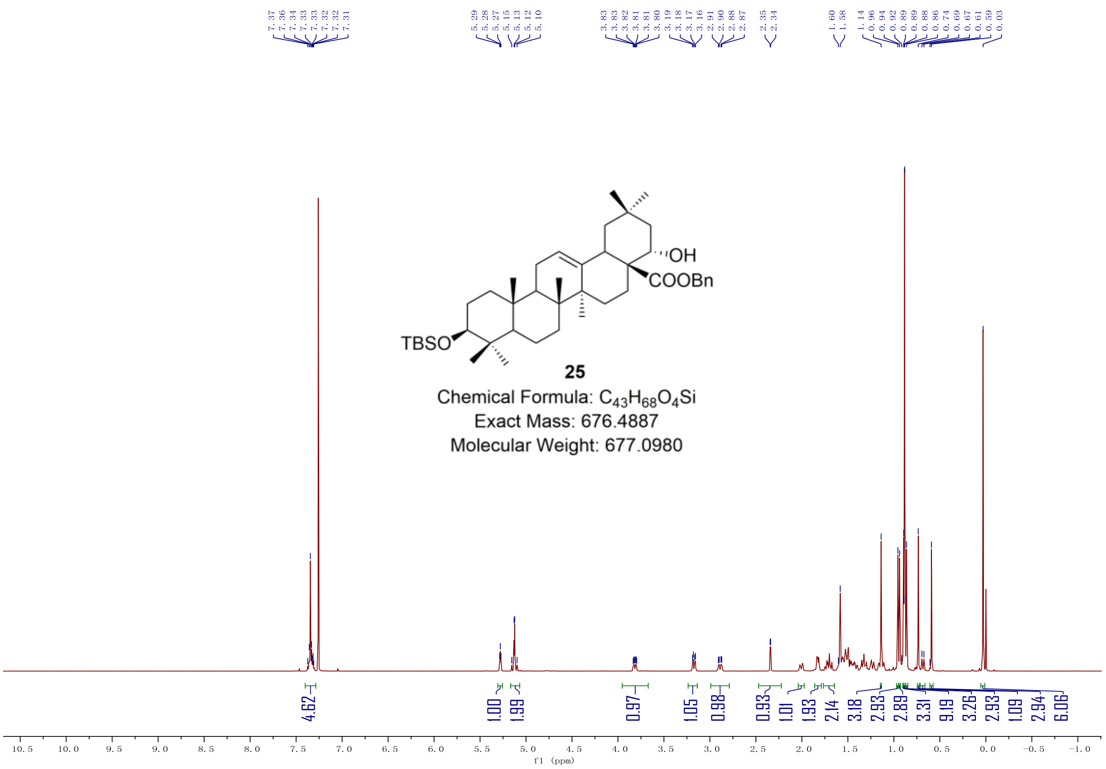

Supplementary Figure 127. ^{1}H-NMR spectrum of compound 25 (500 MHz, $CDCl_3$, 25 °C)

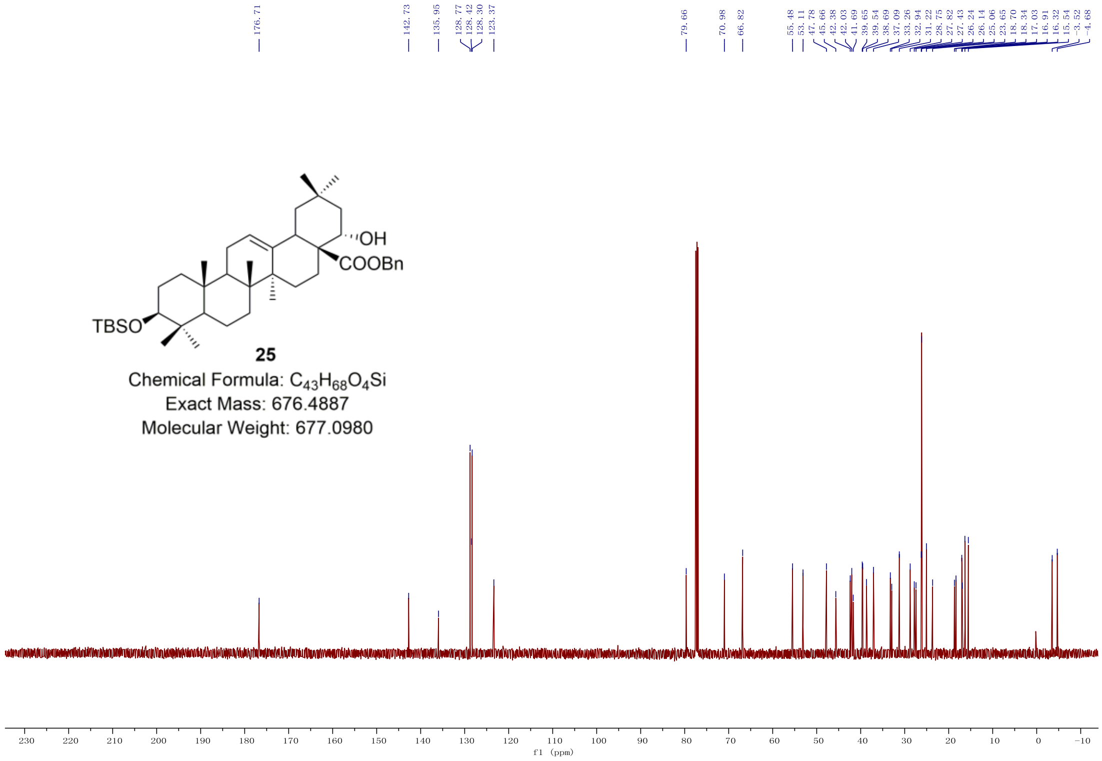

Supplementary Figure 128. ^{13}C-NMR spectrum of compound 25 (126 MHz, $CDCl_3$, 25 °C)

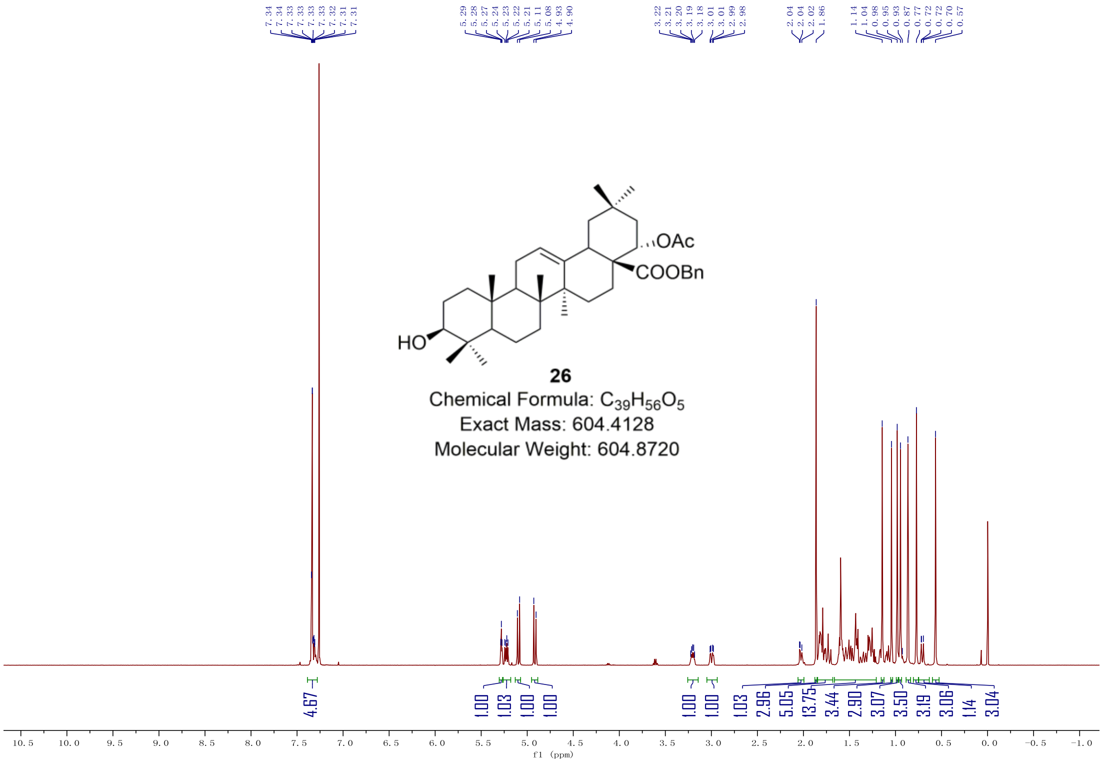

Supplementary Figure 129. ^{1}H-NMR spectrum of compound 26 (500 MHz, $CDCl_3$, 25 °C)

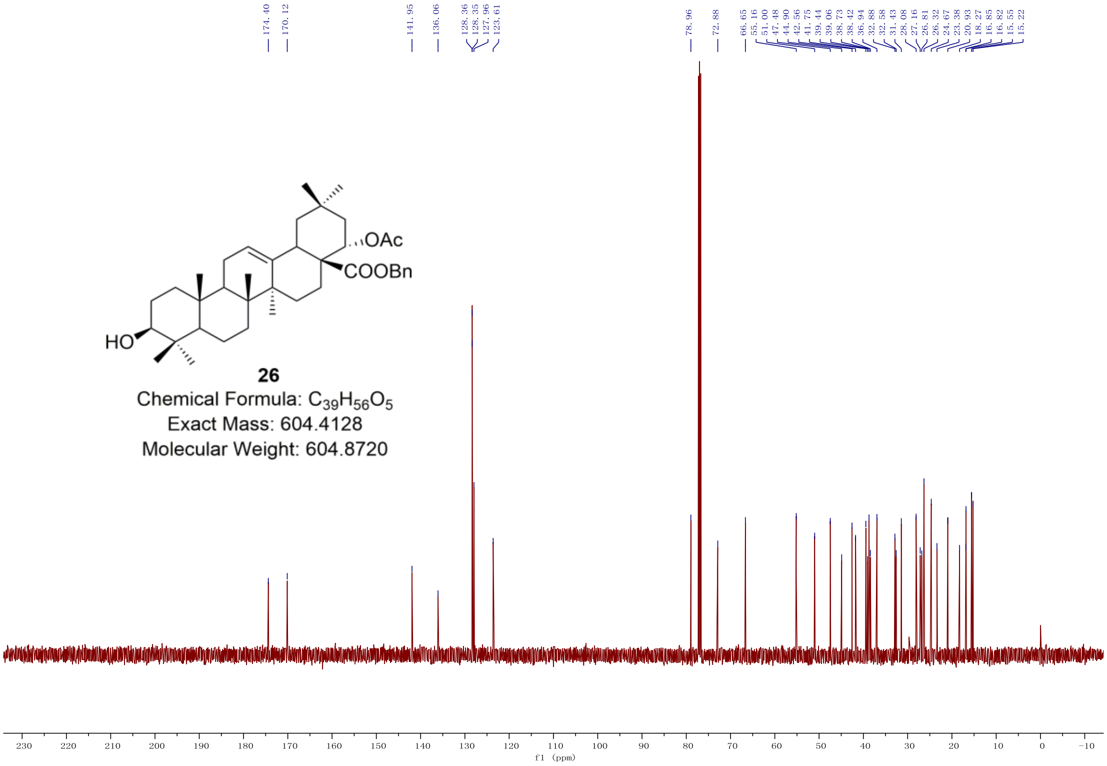

Supplementary Figure 130. ^{13}C-NMR spectrum of compound 26 (126 MHz, $CDCl_3$, 25 °C)

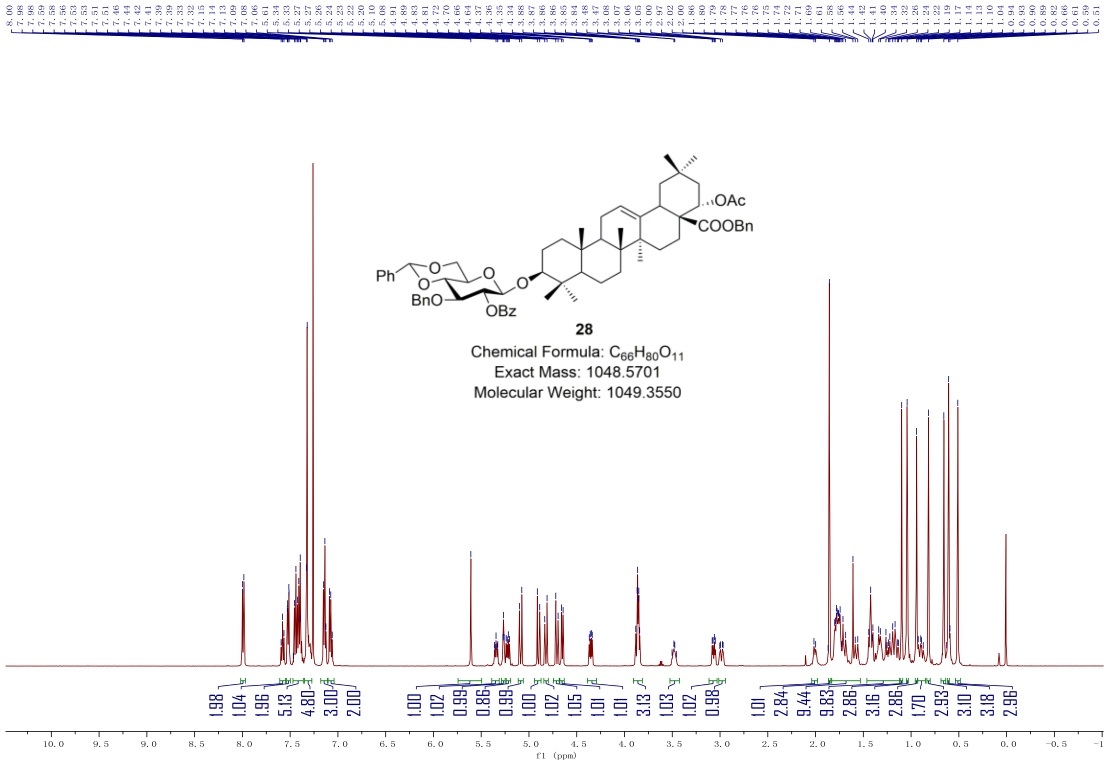

Supplementary Figure 131. ^{1}H-NMR spectrum of compound 28 (500 MHz, $CDCl_3$, 25 ºC)

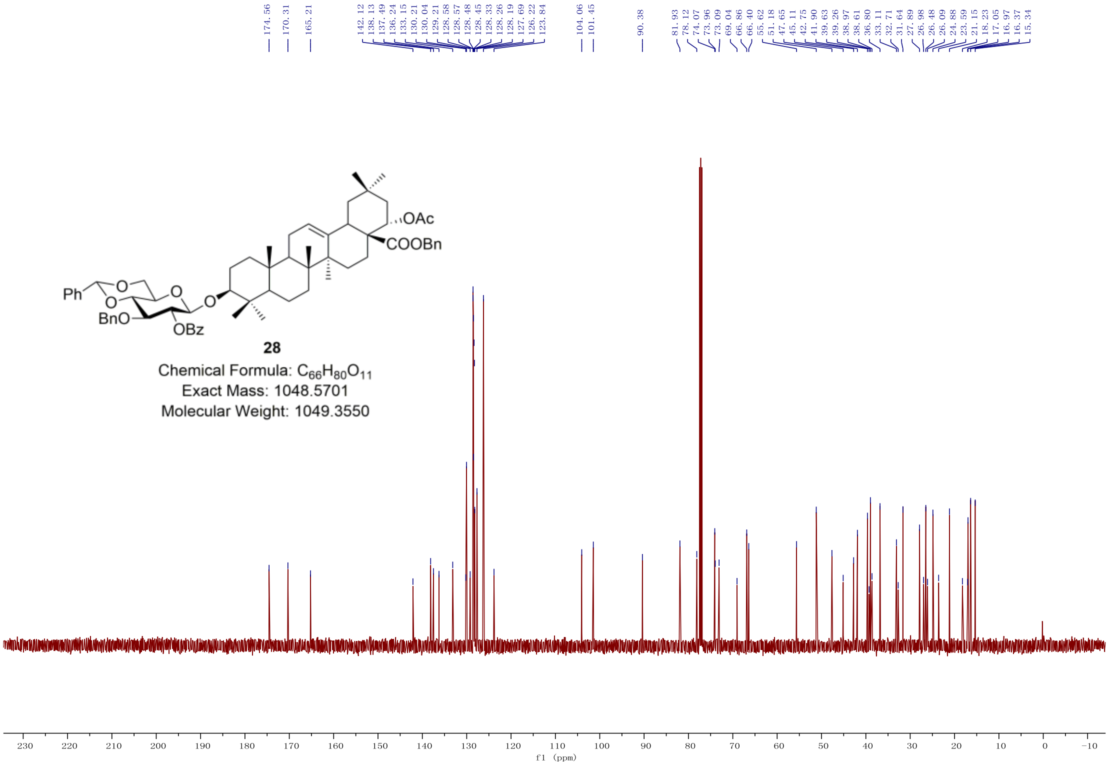

Supplementary Figure 132. ^{13}C-NMR spectrum of compound 28 (126 MHz, $CDCl_3$, 25 ºC)

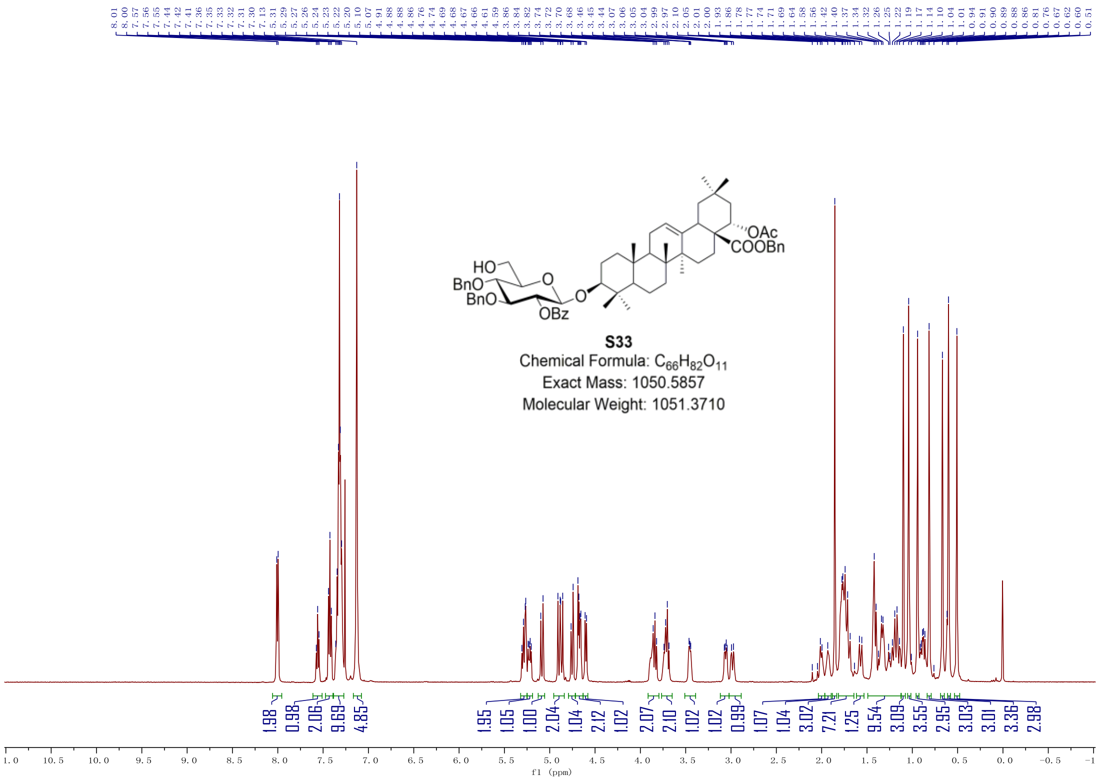

Supplementary Figure 133. ^{1}H-NMR spectrum of compound S33 (500 MHz, $CDCl_3$, 25 ^{o}C)

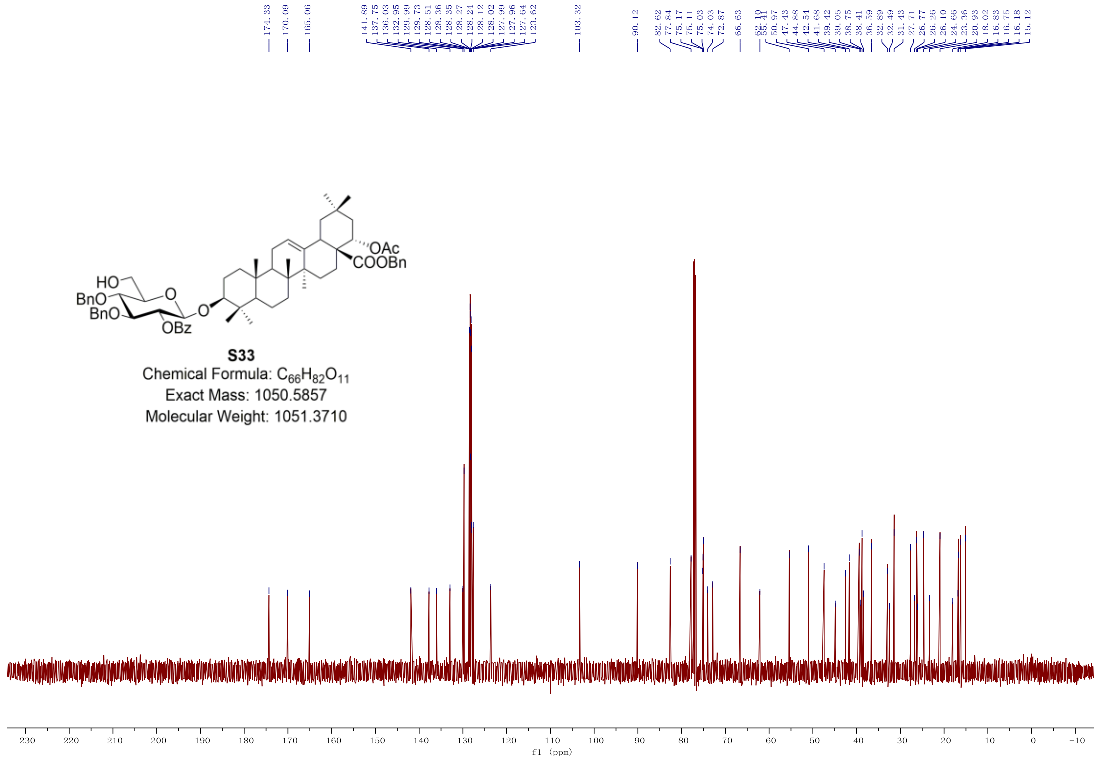

Supplementary Figure 134. ^{13}C-NMR spectrum of compound S33 (126 MHz, $CDCl_3$, 25 ^{o}C)

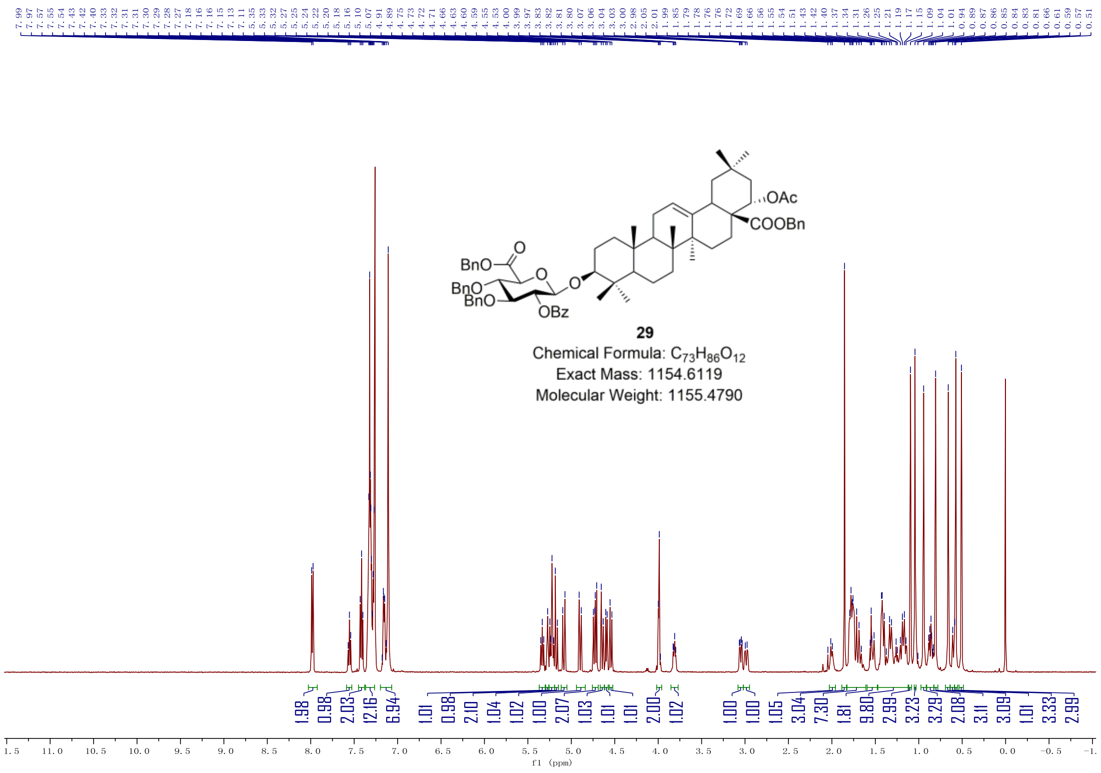

Supplementary Figure 135. ^{1}H-NMR spectrum of compound 29 (500 MHz, $CDCl_3$, 25 ºC)

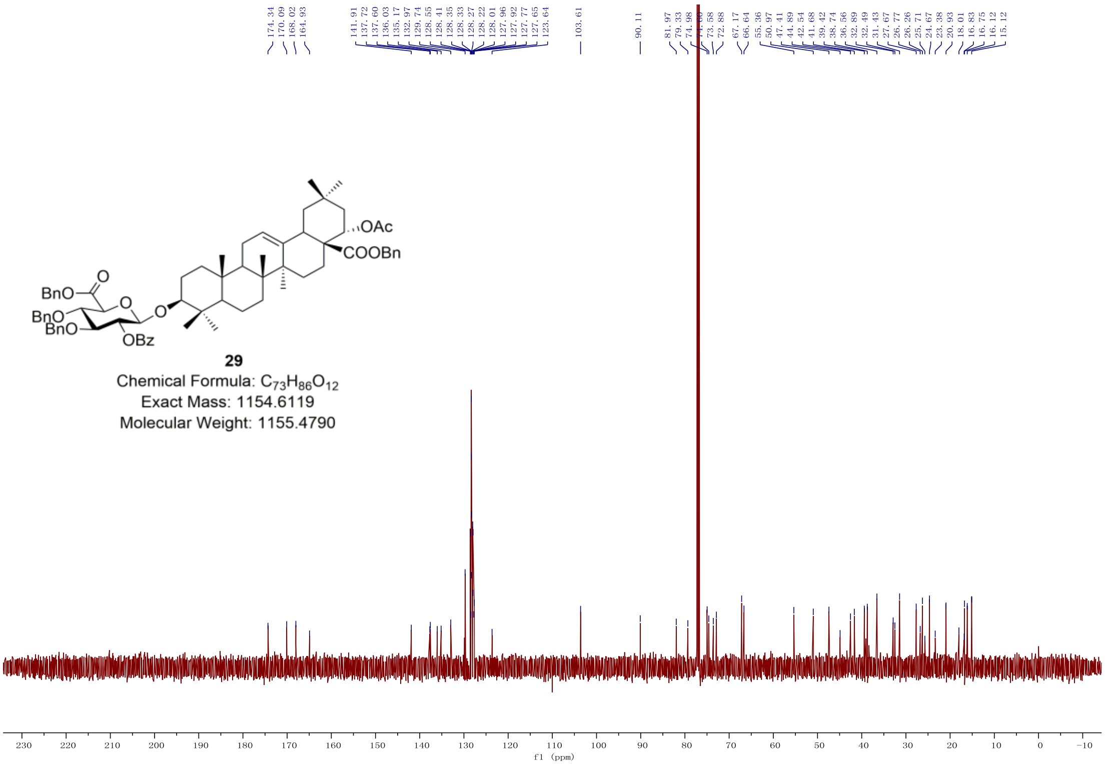

Supplementary Figure 136. ^{13}C-NMR spectrum of compound 29 (126 MHz, $CDCl_3$, 25 ºC)

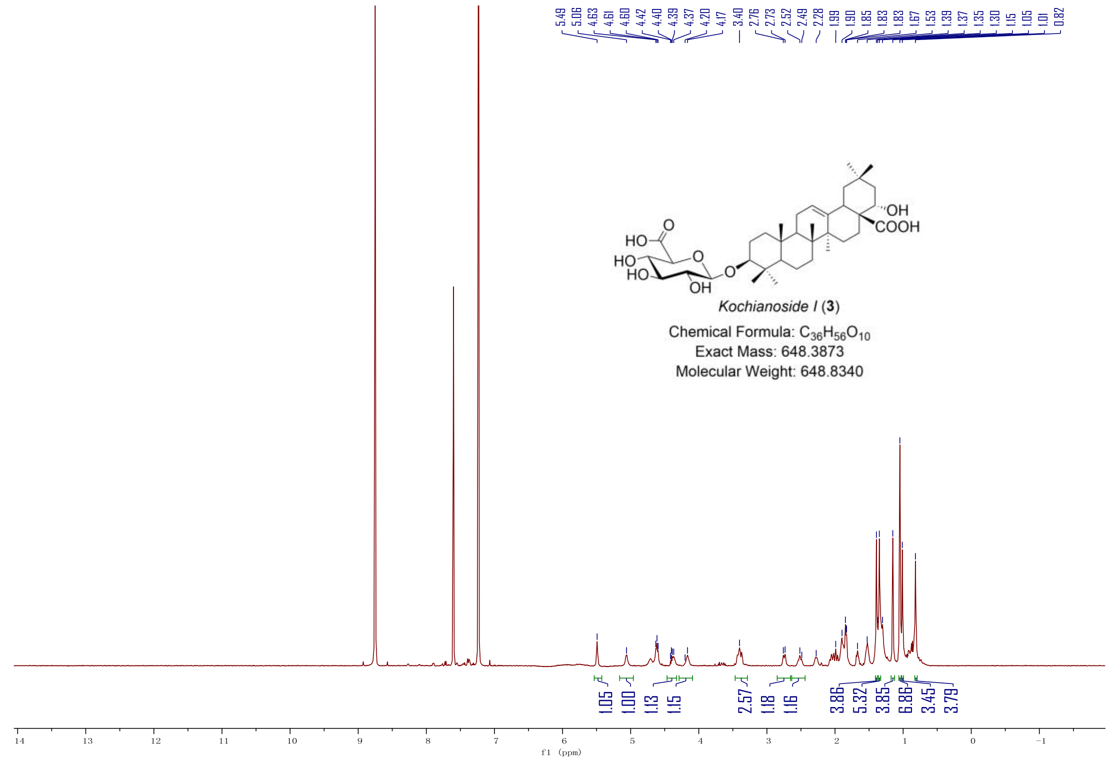

Supplementary Figure 137. ^{1}H-NMR spectrum of compound 3 (500 MHz, pyridine-d5, 25 ºC)

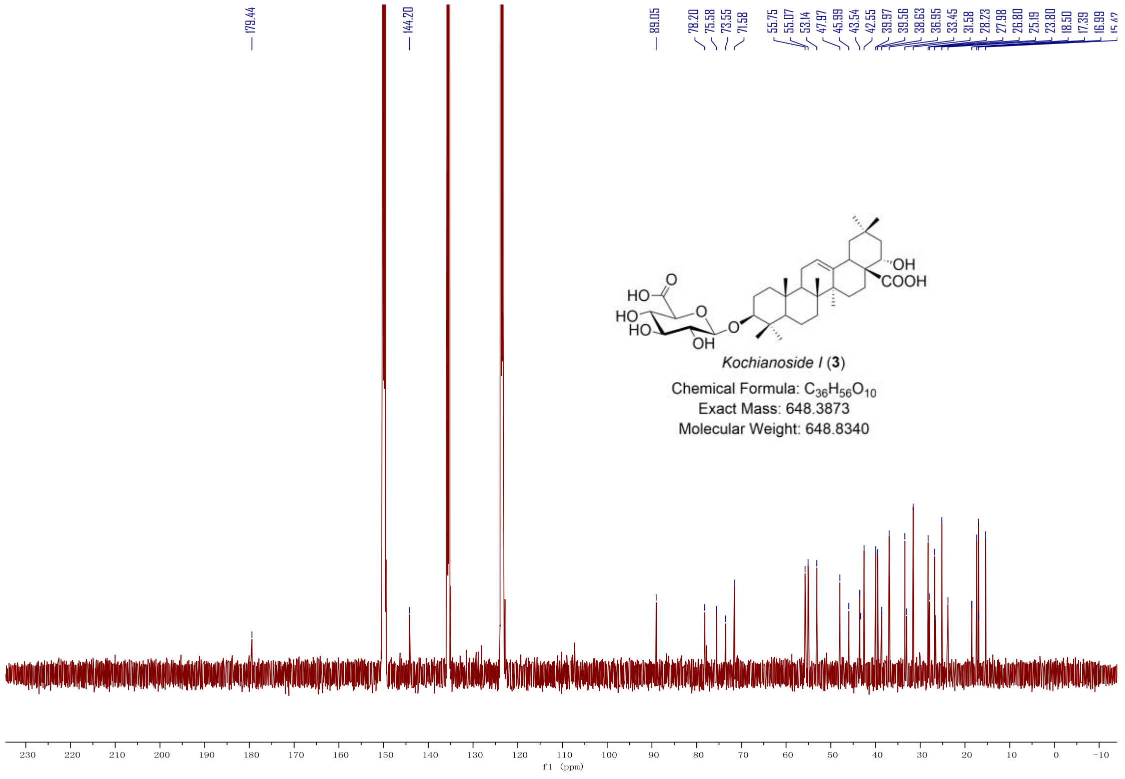

Supplementary Figure 138. ^{13}C-NMR spectrum of compound 3 (126 MHz, pyridine-d5, 25 ºC)

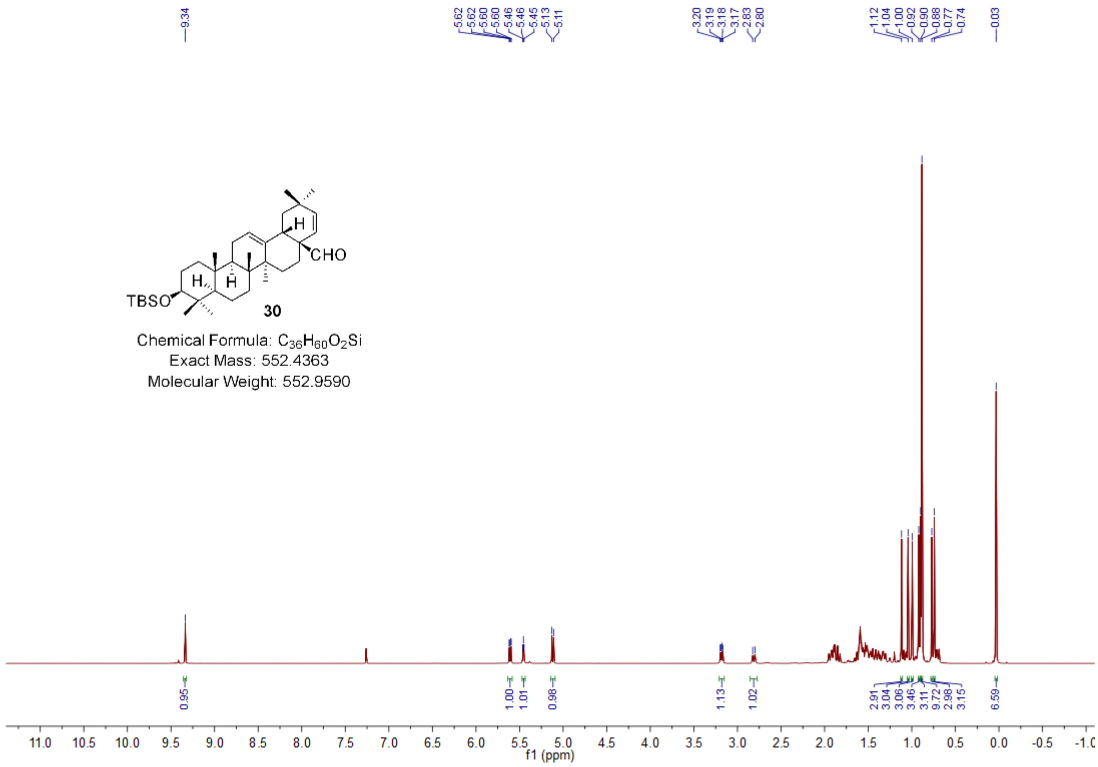

Supplementary Figure 139. ^{1}H-NMR spectrum of compound 30 (500 MHz, $CDCl_3$, 25 ºC)

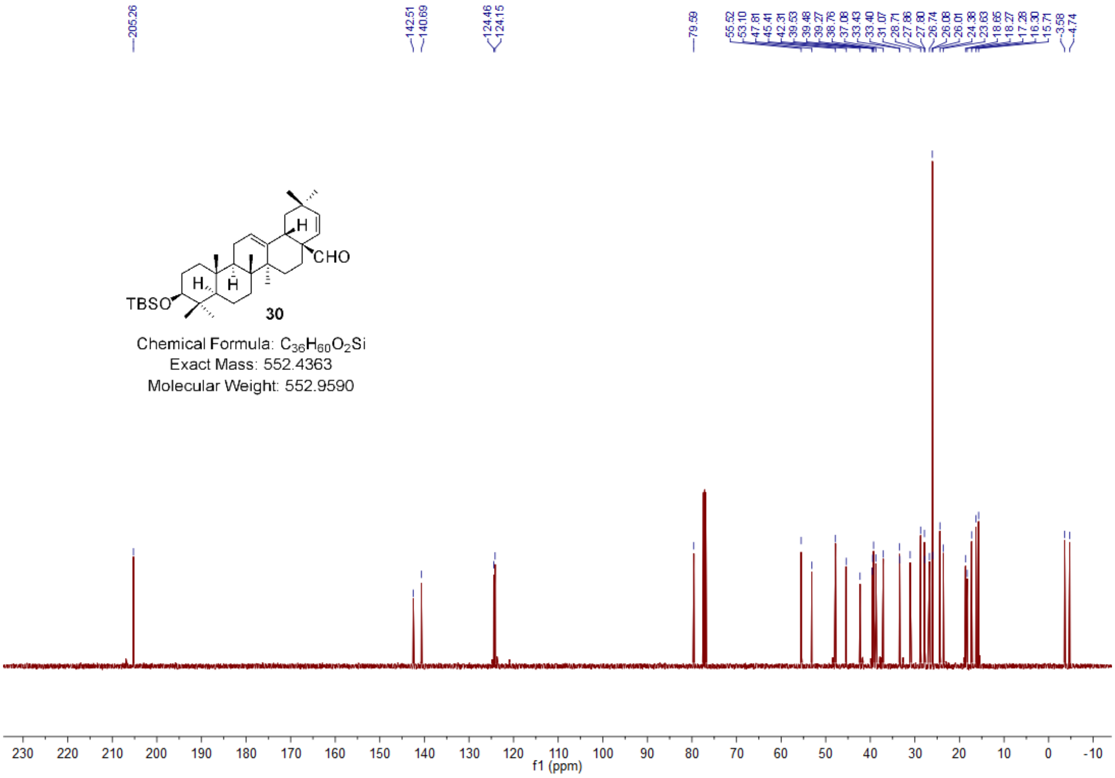

Supplementary Figure 140. ^{13}C-NMR spectrum of compound 30 (126 MHz, $CDCl_3$, 25 ºC)

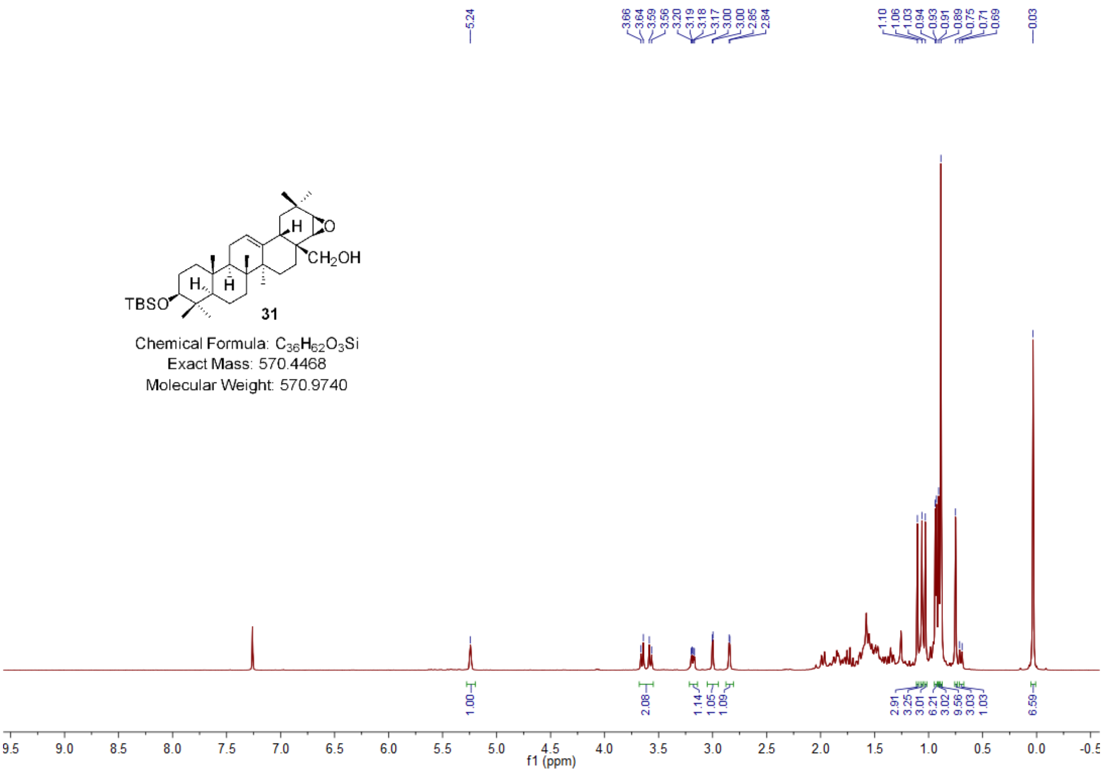

Supplementary Figure 141. **^{1}H-NMR spectrum of compound 31 (500 MHz, $CDCl_3$, 25 °C)**

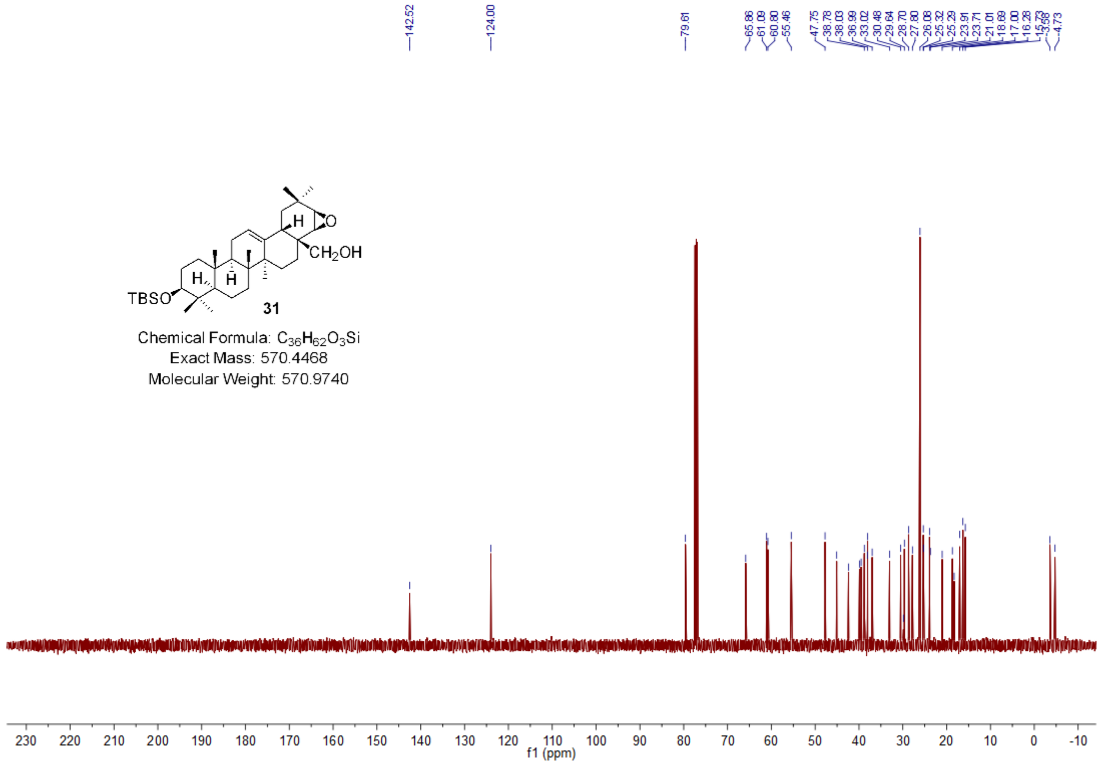

Supplementary Figure 142. **^{13}C-NMR spectrum of compound 31 (126 MHz, $CDCl_3$, 25 °C)**

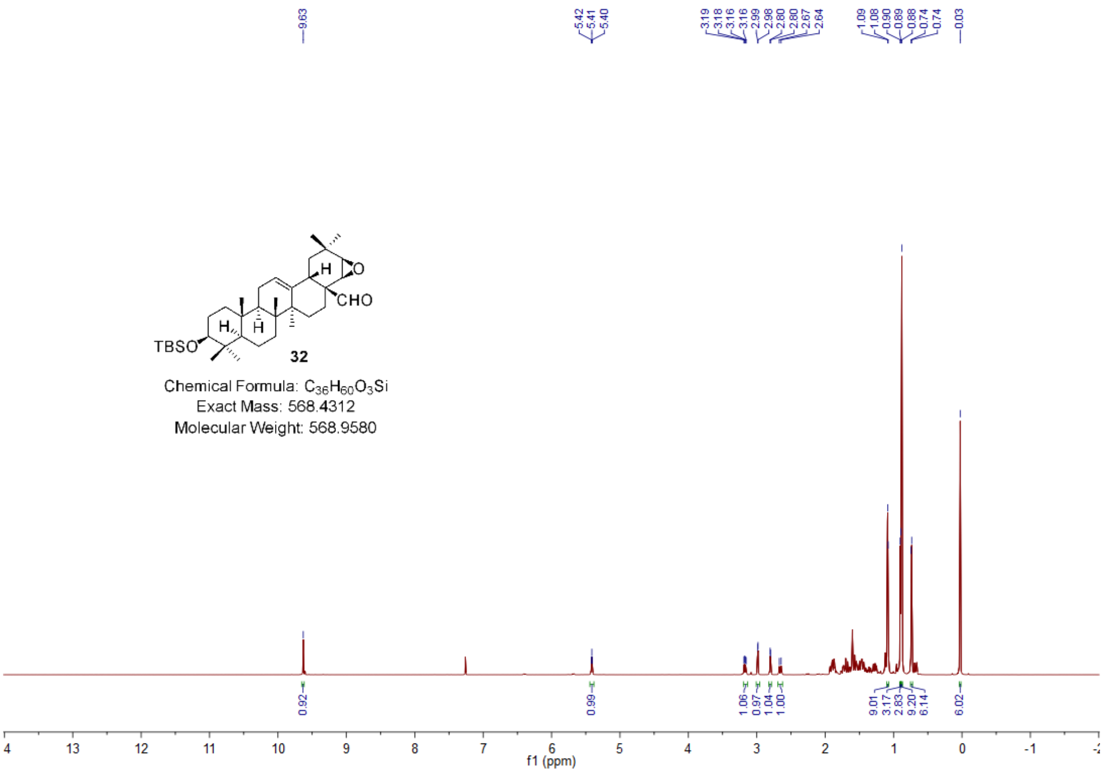

Supplementary Figure 143. **^{1}H-NMR spectrum of compound 32 (500 MHz, $CDCl_3$, 25 °C)**

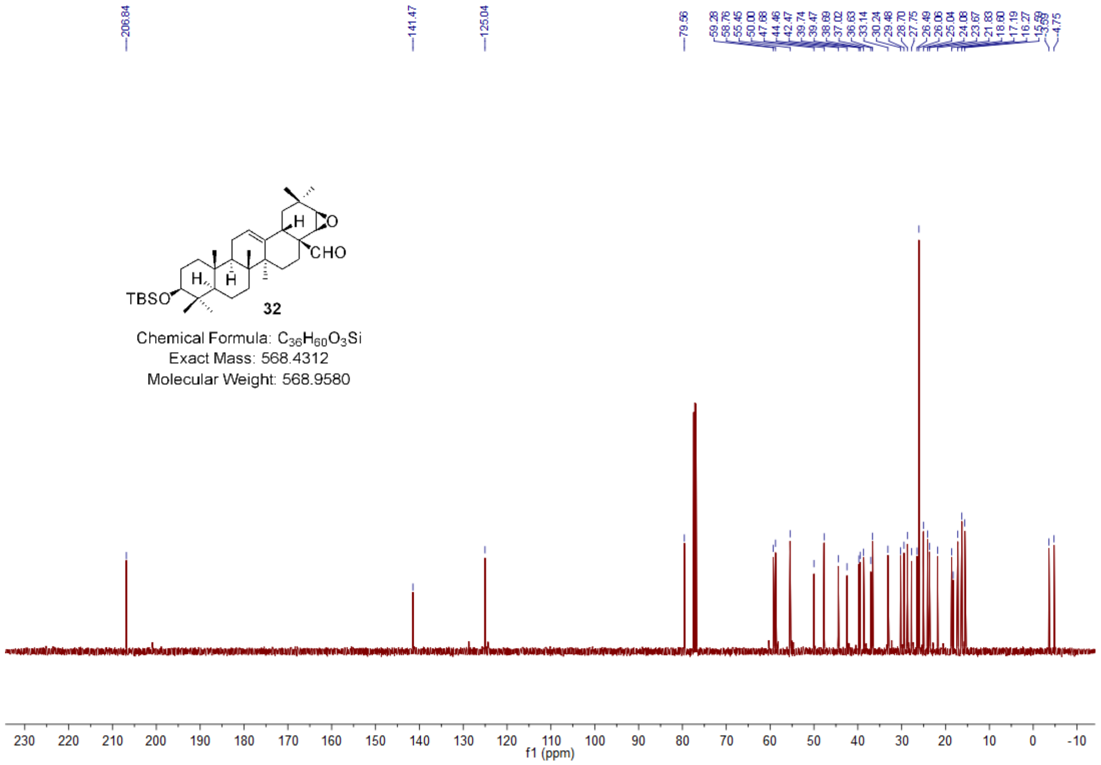

Supplementary Figure 144. **^{13}C-NMR spectrum of compound 32 (126 MHz, $CDCl_3$, 25 °C)**

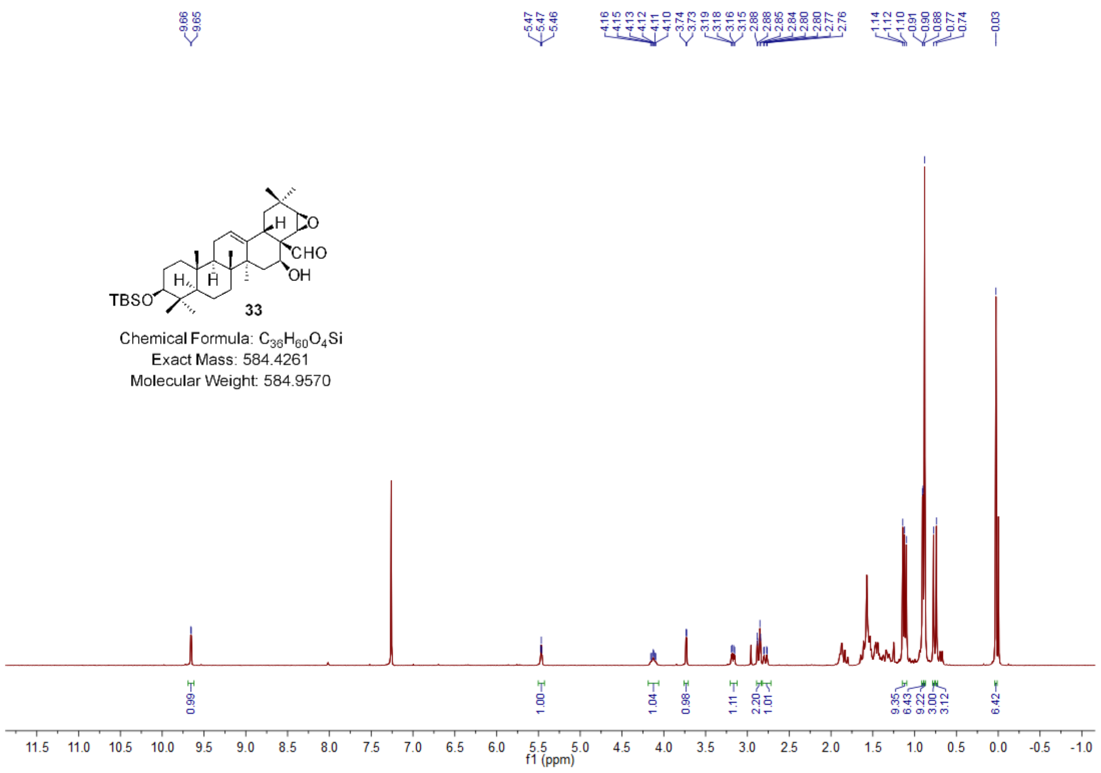

Supplementary Figure 145. ^{1}H-NMR spectrum of compound 33 (400 MHz, $CDCl_3$, 25 °C)

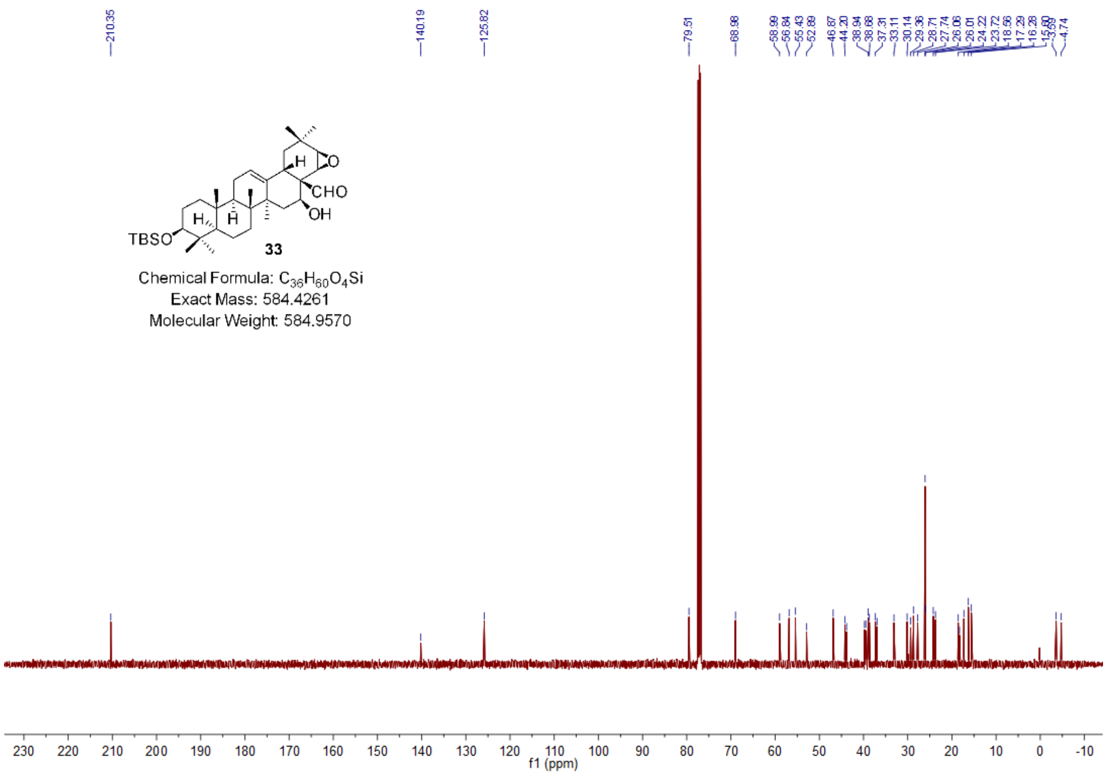

Supplementary Figure 146. ^{13}C-NMR spectrum of compound 33 (126 MHz, $CDCl_3$, 25 °C)

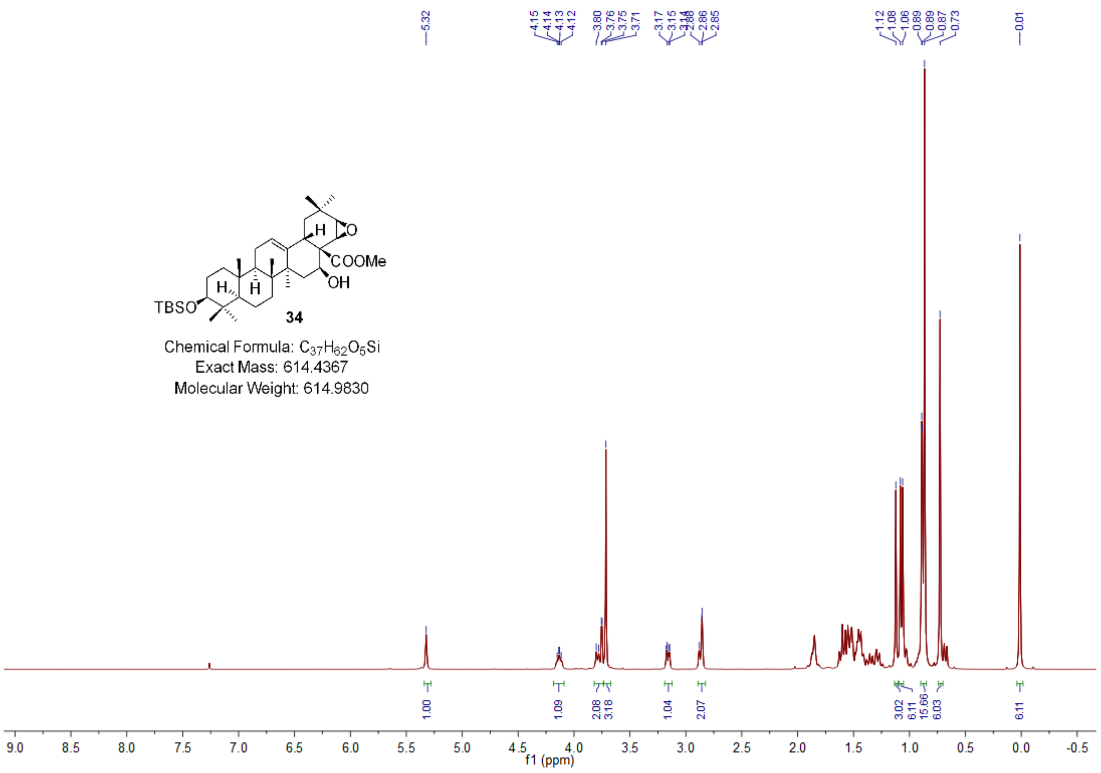

Supplementary Figure 147. ^{1}**H-NMR spectrum of compound 34 (500 MHz, $CDCl_3$, 25 °C)**

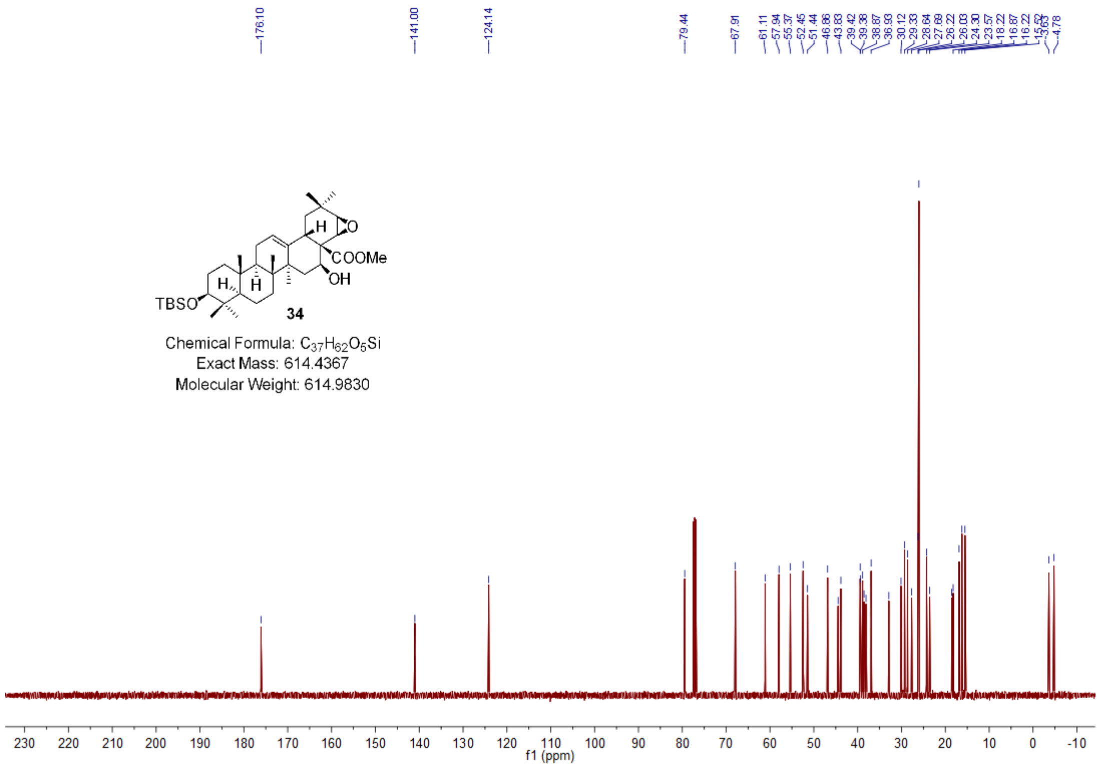

Supplementary Figure 148. ^{13}**C-NMR spectrum of compound 34 (126 MHz, $CDCl_3$, 25 °C)**

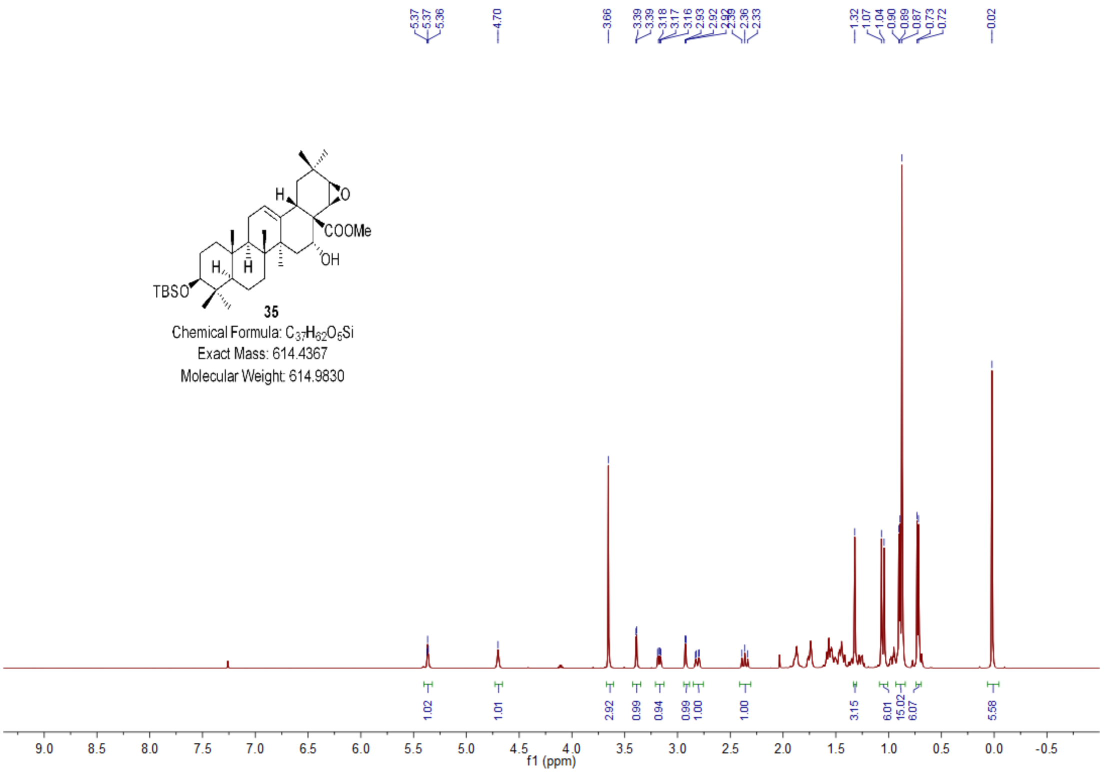

Supplementary Figure 149. **^{1}H-NMR spectrum of compound 35 (500 MHz, $CDCl_3$, 25 °C)**

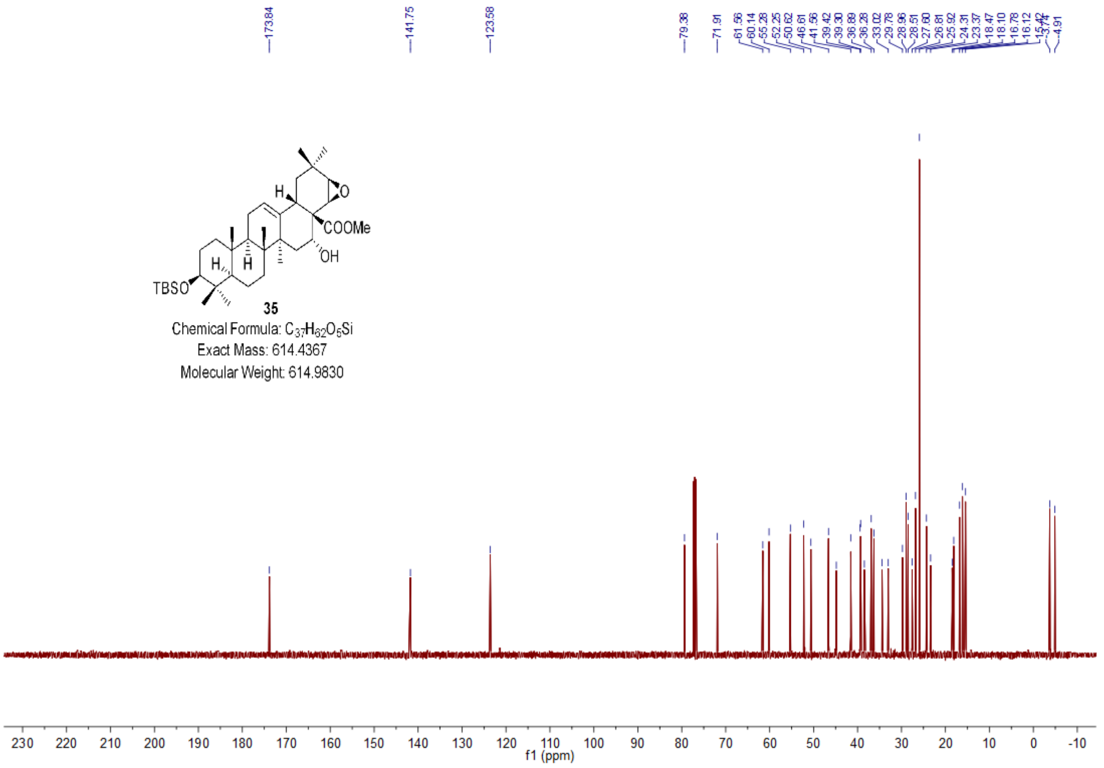

Supplementary Figure 150. **^{13}C-NMR spectrum of compound 35 (126 MHz, $CDCl_3$, 25 °C)**

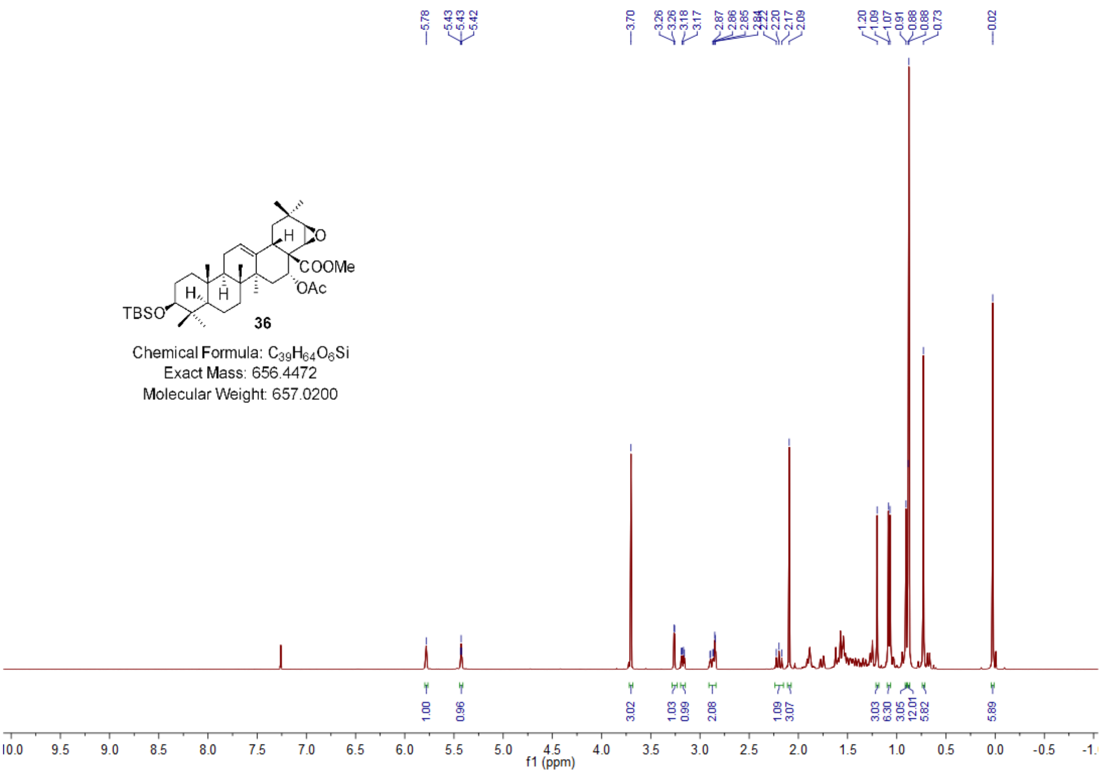

Supplementary Figure 151. ^{1}H-NMR spectrum of compound 36 (500 MHz, $CDCl_3$, 25 °C)

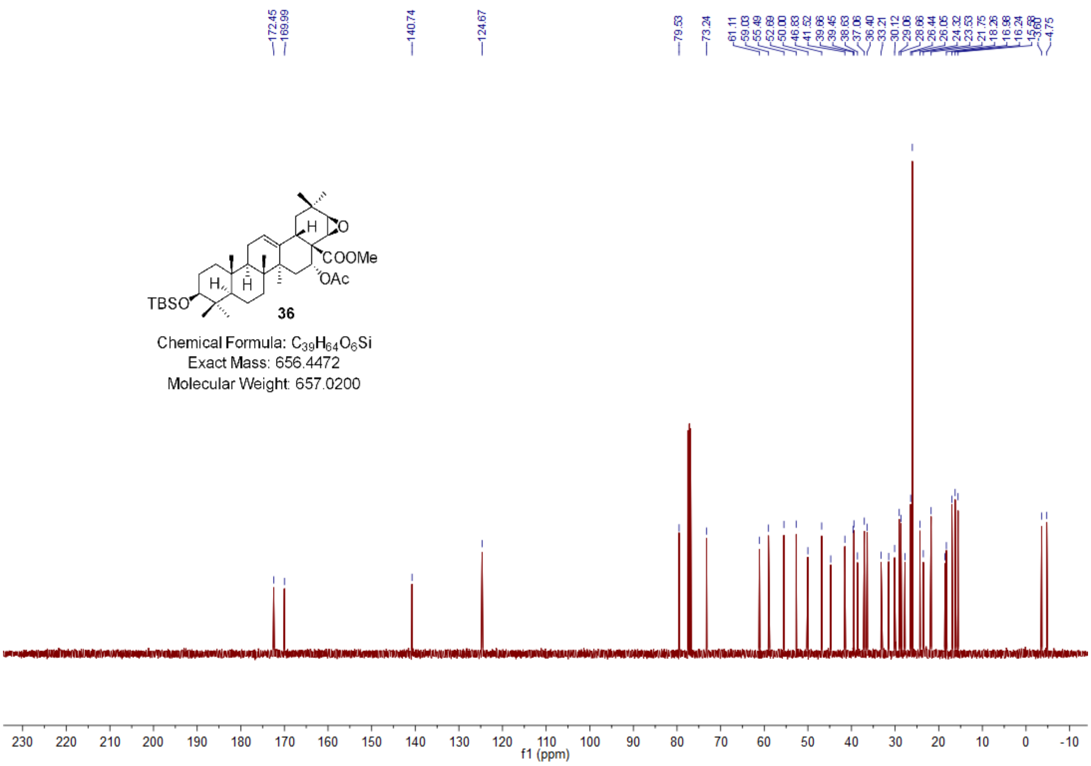

Supplementary Figure 152. ^{13}C-NMR spectrum of compound 36 (126 MHz, $CDCl_3$, 25 °C)

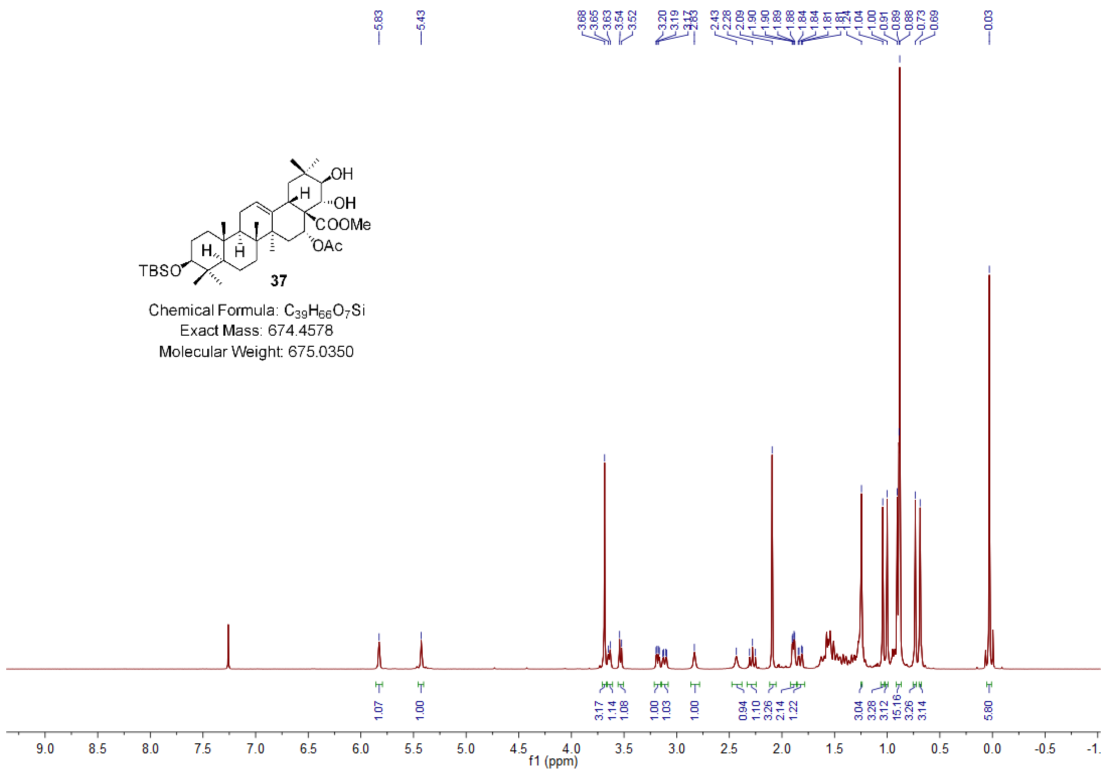

Supplementary Figure 153. ^{1}**H-NMR spectrum of compound 37 (500 MHz, $CDCl_3$, 25 °C)**

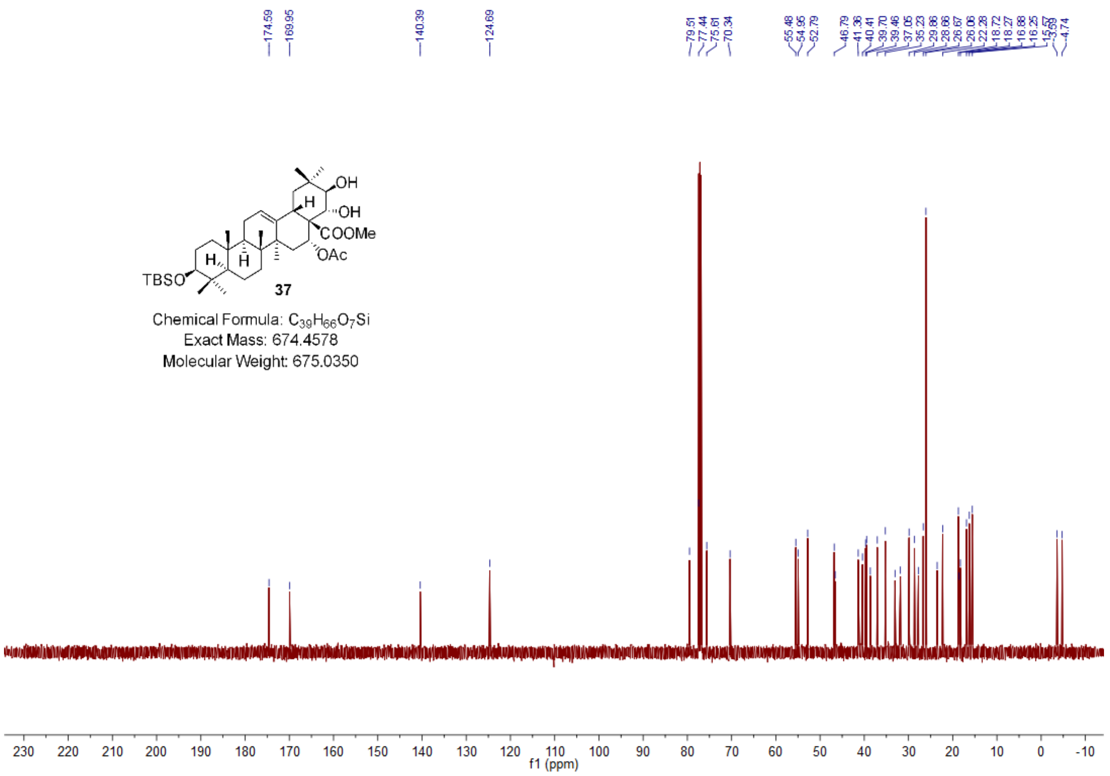

Supplementary Figure 154. ^{13}**C-NMR spectrum of compound 37 (126 MHz, $CDCl_3$, 25 °C)**

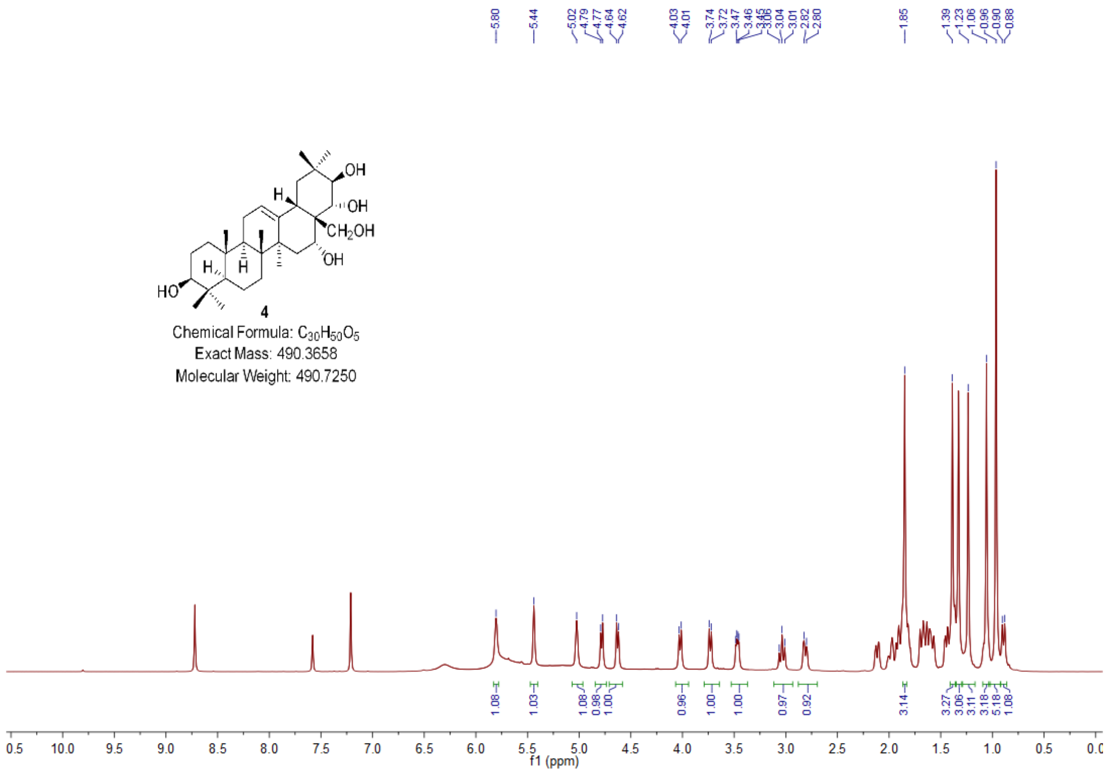

Supplementary Figure 155. ^{1}H-NMR spectrum of compound 4 (400 MHz, pyridine-d5, 25 ºC)

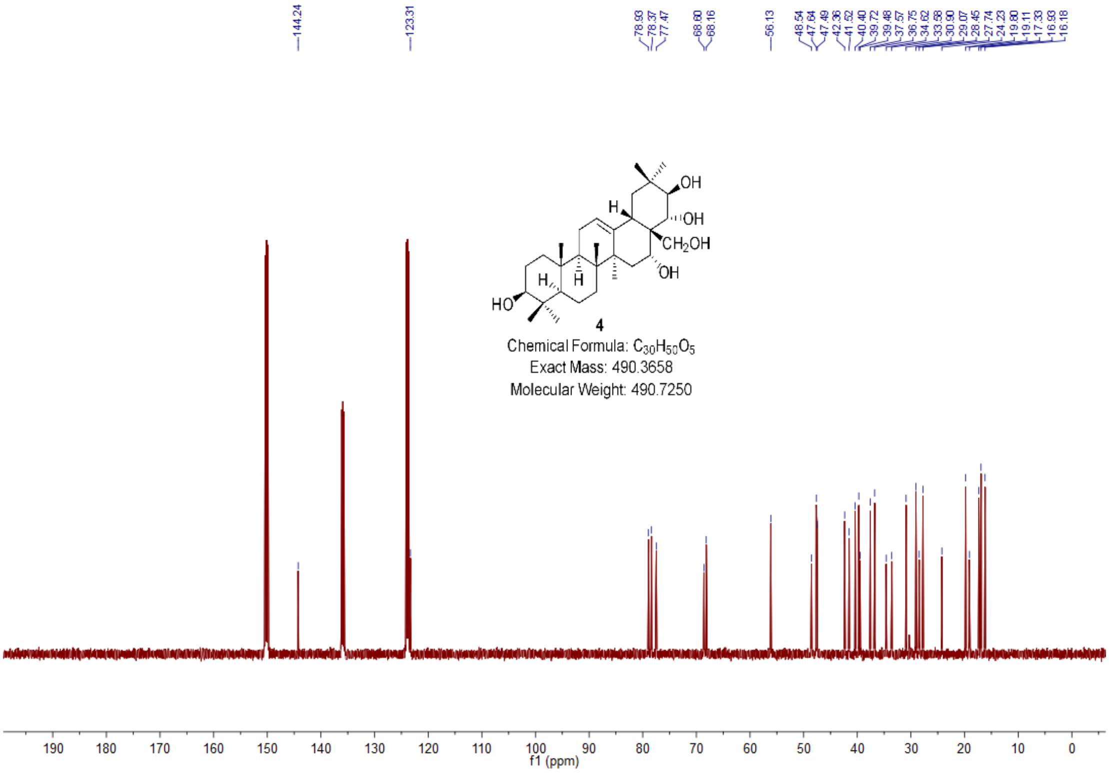

Supplementary Figure 156. ^{13}C-NMR spectrum of compound 4 (126 MHz, pyridine-d5, 25 ºC)

Supplementary Tables

Supplementary Table 1. Summary of the unsuccessful attempts at directed C-H oxidation on PTs.

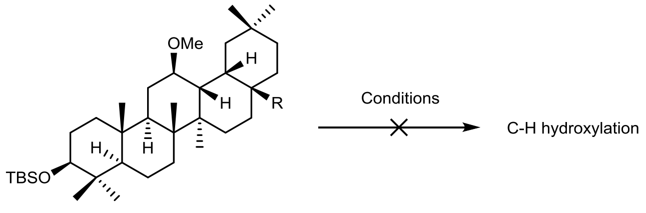

Entry	Substrate	R	Conditions
1	**S1**	CH_3OH	$Pb(OAc)_4$, I_2, $CaCO_3$, hv, benzene, 0 °C to rt
2	**S1**	CH_3OH	1. DMAP, **S12**, DCM, rt<br>2. TFA, TEMPO, toluene, 60 °C
3	**S2**	CHOOH	Fe(S,S-PDP), AcOH, 30% H_2O_2, CH_3CN
4	**S2**	CHOOH	1. $SOCl_2$, 80 °C<br>2. 4-$CF_3(C_6F_4)NH_2$, pyridine, DCM

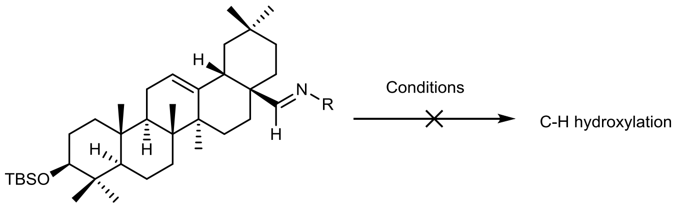

Entry	R	Conditions
5	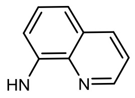	$Pd(OAc)_2$, $PhI(OAc)_2$, AcOH, Ac_2O, 80 °C
6	OH	$Pd(OAc)_2$, $PhI(OAc)_2$ AcOH, Ac_2O, 100 °C
7	OMe	$Pd(OAc)_2$, $PhI(OAc)_2$ AcOH, Ac_2O, 100 °C
8	OBn	$Pd(OAc)_2$, $PhI(OAc)_2$ AcOH, Ac_2O, 100 °C

Supplementary Table 2. Comparison of the ^{13}C NMR data of synthetic 1 with those of the natural Saikosaponin E

Position	^{13}C		
	Reported[9]	Synthetic **1**	Δδ
1	38.60	38.56	-0.04
2	26.60	26.57	-0.03
3	88.60	88.62	0.02
4	39.70	39.74	0.04
5	55.30	55.30	0.00
6	17.90	17.87	-0.03
7	31.80	31.85	0.05
8	42.20	42.19	-0.01
9	52.90	52.88	-0.02
10	36.30	36.34	0.04
11	132.00	132.03	0.03
12	131.20	131.23	0.03
13	84.00	83.95	-0.05
14	45.60	45.63	0.03
15	36.10	36.12	0.02
16	64.00	64.01	0.01
17	47.00	47.00	0.00
18	52.10	52.13	0.03
19	37.80	37.76	-0.04
20	31.60	31.61	0.01
21	34.70	34.69	-0.01
22	25.70	25.74	0.04
23	27.80	27.80	0.00
24	16.30	16.32	0.02
25	18.20	18.20	0.00
26	20.00	19.98	-0.02
27	20.90	20.89	-0.01
28	73.00	73.02	0.02
29	33.70	33.66	-0.04
30	23.80	23.80	0.00
C-1'	106.80	106.82	0.02
C-2'	71.70	71.72	0.02
C-3'	85.20	85.15	-0.05
C-4'	71.50	71.53	0.03
C-5'	71.00	70.96	-0.04
C-6'	17.30	17.28	-0.02
C-1"	106.80	106.72	-0.08
C-2"	75.90	75.83	-0.07

C-3"	78.40	78.40	0.00
C-4"	72.20	72.17	-0.03
C-5"	78.70	78.72	0.02
C-6"	62.70	62.67	-0.03

Supplementary Table 3. Comparison of the ^{13}C NMR data of synthetic 2 with those of the natural Albizai saponin.

Position	^{13}C		
	Reported[10]	Synthetic **2**	Δδ
1	39.1	39.1	0.0
2	26.7	27.7	1.0
3	89.9	89.8	0.1
4	39.5	39.8	0.3
5	56.7	56.3	-0.4
6	18.9	19.1	0.2
7	33.7	33.9	0.2
8	40.4	40.4	0.0
9	47.6	47.9	0.3
10	37.4	37.5	0.1
11	24.1	24.2	0.1
12	122.6	123.0	0.4
13	145.2	145.5	0.3
14	42.5	42.6	0.1
15	36.4	36.7	0.3
16	74.9	74.9	0.0
17	*	49.4	
18	42.0	41.9	-0.1
19	47.6	47.6	0.0
20	31.0	31.6	0.6
21	36.4	36.7	0.3
22	*	34.0	
23	28.4	28.6	0.2
24	17.1	17.9	0.8
25	15.8	16.0	0.2
26	17.1	17.5	0.4
27	*	27.0	
28	*	180.4	
29	33.4	33.4	0.0
30	*	24.3	

C-1'	104.6	105.6	1.0
C-2'	58.5	58.4	-0.1
C-3'	76.5	77.2	0.7
C-4'	73.2	73.3	0.1
C-5'	76.0	76.6	0.6
C-6'	69.9	69.6	-0.3
-CO-NH-	24.2	25.3	1.1
Me(CO-NH-)	171.0	170.6	-0.5
C-1"	104.9	105.8	0.9
C-2"	72.3	72.8	0.5
C-3"	74.2	75.3	1.1
C-4"	68.6	66.9	-1.7
C-5"	65.8	*	

Supplementary Table 4. Comparison of ^{13}C NMR data of the synthetic 3 with those of the natural Kochianoside I.

Position	^{13}C		
	Reported[11]	Synthetic **3**	Δδ
1	38.7	38.6	-0.1
2	26.6	26.6	0.0
3	89.1	89.0	-0.1
4	39.6	39.5	-0.1
5	55.8	55.7	-0.1
6	18.5	18.5	0.0
7	33.1	33.0	-0.1
8	40.0	39.9	-0.1
9	48.0	47.9	-0.1
10	37.0	36.9	-0.1
11	23.8	23.8	0.0
12	122.9	123.0	+0.1
13	144.2	144.2	0.0
14	42.6	42.5	-0.1
15	28.0	28.0	0.0
16	17.0	17.0	0.0
17	53.1	53.1	0.0
18	43.5	43.5	0.0
19	46.1	46.0	-0.1
20	31.5	31.5	0.0
21	43.3	43.3	0.0
22	71.5	71.5	0.0
23	28.3	28.2	-0.1
24	17.0	16.9	-0.1
25	15.4	15.4	0.0
26	17.4	17.4	0.0
27	26.8	26.8	0.0
28	179.4	179.4	0.0
29	33.4	33.4	0.0
30	25.2	25.1	-0.1
C-1’	107.2	107.2	0.0
C-2’	75.6	75.5	-0.1
C-3’	78.2	78.2	0.0
C-4’	73.4	73.5	0.1
C-5’	77.8	77.8	0.0
C-6’	172.7	*	*

Supplementary Table 5. Comparison of the ^{1}H NMR data of synthetic 4 with those of the natural Barringtogenol C

Position	^{1}H		
	Reported[12]	Synthetic **4**	Δδ
1			
2			
3	3.47	3.46 dd (5.1,10.76)	-0.01
4			
5			
6			
7			
8			
9			
10			
11			
12	5.45 t (3.5)	5.43 t (3.22)	-0.02
13			
14			
15			
16	5.05	5.03	-0.02
17			
18	2.81 dd (4.0, 14.0)	2.81 dd (4.21, 13.79)	0
19	3.05 t (14.0)	3.04 t (13.43)	-0.01
20			
21	4.78 d (9.5)	4.78 d (9.58)	0
22	4.63 d (9.5)	4.63 d (9.54)	0
23	1.24	1.22	-0.02
24	1.06	1.05	-0.01
25	0.98	0.96	-0.02
26	0.97	0.95	-0.02
27	1.86	1.85	-0.01
28	4.04 dd (4.0,10.0)	4.03 d (10.42)	-0.01
	3.74 d (10.0)	3.73 d (10.41)	-0.01
29	1.34	1.32	-0.02
30	1.39	1.38	-0.01

Supplementary Table 6. Comparison of the ^{13}C NMR data of synthetic 4 with those of the natural Barringtogenol C

Position	^{13}C		
	Reported[13]	Synthetic **4**	Δδ
1	39.4	39.19	0.02
2	28.1	27.95	0.08
3	78.1	77.82	-0.05
4	39.2	38.94	-0.03
5	55. 9	55.59	-0.08
6	18.8	18.56	-0.01
7	33.3	33.05	-0.02
8	40.2	39.88	-0.09
9	47.3	47.11	0.04
10	37.3	37.04	-0.03
11	23.9	23.68	0.01
12	123	122.77	0
13	143.9	143.75	0.08
14	42.1	41.86	-0.01
15	34.3	34.12	0.05
16	67.9	67.64	-0.03
17	47.2	46.96	-0.01
18	41.3	41.04	-0.03
19	48.3	48.04	-0.03
20	36.4	36.21	0.04
21	78.7	78.45	-0.02
22	77.5	77.09	-0.18
23	28.7	28.51	0.04
24	16.5	16.35	0.08
25	15.8	15.61	0.04
26	17	16.79	0.02
27	27.4	27.19	0.02
28	68.5	68.2	-0.07
29	30.5	30.36	0.09
30	19.4	19.24	0.07

Supplementary Methods

General information

All reactions were carried out under argon with anhydrous solvents in flame-dried glassware, unless otherwise noted. Tetrahydrofuran (THF) was distilled immediately before use from sodium under an argon atmosphere. Methylene chloride (CH_2Cl_2), *N*,*N*-dimethylformamide (DMF), triethylamine (Et_3N), methanol (MeOH), ethanol (EtOH), acetone ($(CH_3)_2CO$), acetonitrile (MeCN), toluene, and pyridine were treated in standard processes. Reagents were purchased at the highest commercial quality and used without further purification, unless otherwise stated. Solvents for chromatography were used as supplied by Adamas-beta®. Reactions were monitored by thin layer chromatography (TLC) carried out on Millipore Sigma glass TLC plates (silica gel 60 coated with F254, 250 μm) using UV light for visualization and aqueous ammonium cerium nitrate/ammonium molybdate or basic aqueous potassium permanganate as developing agent. SiliaFlash® P60 silica gel (particle size: 40–63 μm, pore size: 60 Å) was used for flash column chromatography. NMR spectra were recorded on a Bruker Avance III 400 MHz or an Agilent DD2 500 MHz NMR spectrometer. The spectra were calibrated by using residual undeuterated solvents (for ^{1}H NMR) and deuterated solvents (for ^{13}C NMR) as internal references: undeuterated chloroform (δH = 7.26 ppm) and $CDCl_3$ (δC = 77.16 ppm). The following abbreviations are used to designate multiplicities: s = singlet, d = doublet, t = triplet, q = quartet, m = multiplet, br = broad. IR spectra were recorded on a Thermo Scientific Nicolet 380 FT-IR spectrometer. Melting points (m.p.) are uncorrected and were recorded on an SGW X-4 apparatus. High-resolution mass spectra (HRMS) were recorded on a Bruker Apex III 7.0 Tesla FT-ICR, an IonSpec 4.7 Tesla FT-ICR, or a Waters Micromass GCT Premier mass spectrometer.

Preparation of the C-H activation substrates (S1, S2, and 5-15)

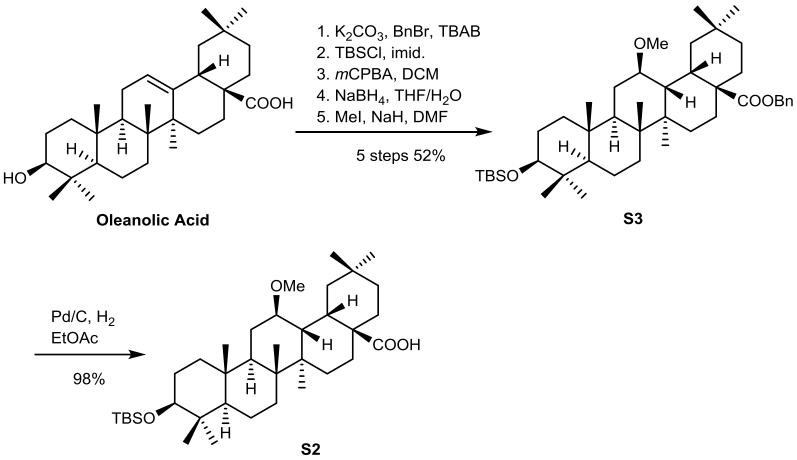

Ester S3 | Compound **S3** (76.05 g, 52%) was prepared as a white solid from oleanolic acid (100 g, 219 mmol), employing a literature procedure.[14]

Acid S2 | To a stirred solution of **S3** (2.0 g, 2.88 mmol) in EtOAc (10 mL), was added 10% Pd/C (300 mg). The mixture was allowed to stir at 25 °C for 2 hours under an atmosphere of H_2 (1 atm).

Then the mixture was filtered through a pad of celite and washed with EtOAc (3 × 10 mL). The filtrate was concentrated under vacuum. The residue was purified by flash column chromatography (EtOAc/petroleum ether, 1:5) to give **S2** (1.70 g, 98%) as a white foam. R_f = 0.5 (silica, PE/EtOAc = 3:1); $[\alpha]_D^{25}$ = -1.4 (*c* 1.0, $CHCl_3$); IR (film) ν_{max} = 2949, 1693, 1462, 1258, 1097, 835 cm^{-1}; ^1H NMR (400 MHz, $CDCl_3$) δ 3.36 (s, 3H), 3.33–3.22 (m, 1H), 3.16 (dd, *J* = 11.2, 4.5 Hz, 1H), 2.77–2.68 (m, 1H), 0.95 (s, 3H), 0.92 (s, 3H), 0.91 (s, 3H), 0.88 (s, 15H), 0.86 (s, 3H), 0.82 (s, 3H), 0.72 (s, 3H), 0.64 (d, *J* = 9.5 Hz, 1H), 0.03 (s, 6H); ^{13}C NMR (126 MHz, $CDCl_3$) δ 182.95, 79.49, 77.54, 56.03, 55.51, 49.04, 47.28, 41.61, 41.31, 40.47, 39.58, 38.83, 37.20, 36.59, 34.56, 33.65, 33.40, 32.70, 31.30, 30.76, 28.86, 28.56, 27.85, 26.13, 26.07, 23.61, 23.21, 18.58, 18.27, 18.06, 16.69, 16.05, 15.98, -3.60, -4.76; ESI-HRMS (*m/z*) calcd for $C_{37}H_{66}O_4SiNa$ $[M + Na]^+$ 625.4623, found 625.4627.

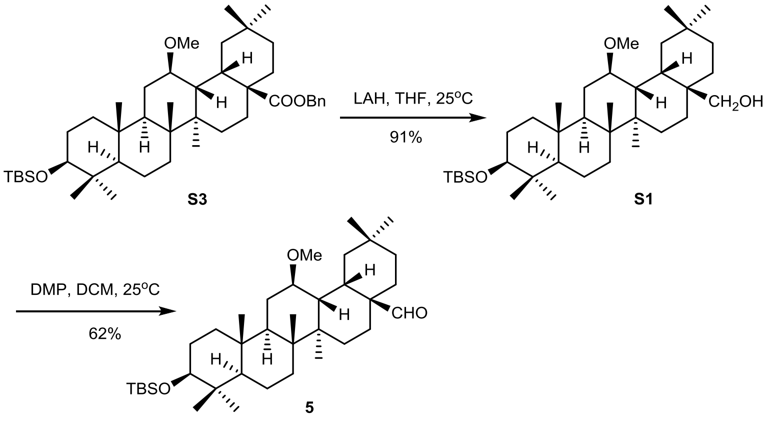

Alcohol S1 | To a stirred solution of **S3** (3.0 g, 4.33 mmol, 1.0 equiv.) in THF (10 mL), was added $LiAlH_4$ (493 mg, 13.0 mmol, 3.0 equiv.) at 0 ℃. The mixture was stirred at 25 °C for 2 hours; the excess reagents werequenched with water and 15% NaOH (20 mL and 20 mL). The resulting mixture was extracted with EtOAc (3 × 20 mL). The combined organic phases were washed with brine (10 mL), dried over anhydrous Na_2SO_4, filtered, and concentrated under vacuum. The residue was purified by flash column chromatography (EtOAc/petroleum ether, 1:10) to give **S1** (2.70 g, 91%) as a colorless oil. R_f = 0.3 (silica, PE/EtOAc = 10:1); $[\alpha]_D^{28}$ = 4.5 (*c* 1.0, $CHCl_3$); IR (film) ν_{max} = 3445, 2948, 2856, 1647, 1098, 758 cm^{-1}; ^1H NMR (400 MHz, $CDCl_3$) δ 3.51 (s, 2H), 3.32 (s, 3H), 3.24 (td, *J* = 10.2, 5.5 Hz, 1H), 3.16 (dd, *J* = 11.3, 4.6 Hz, 1H), 2.03 (dd, *J* = 10.9, 4.5 Hz, 1H), 1.98–1.90 (m, 1H), 1.86 (dd, *J* = 10.9, 5.2 Hz, 1H), 1.00 (s, 3H), 0.95 (s, 3H), 0.90 (s, 3H), 0.88 (s, 12H), 0.84 (s, 6H), 0.73 (s, 3H), 0.64 (d, *J* = 9.2 Hz, 1H), 0.03 (s, 6H); ^{13}C NMR (101 MHz, $CDCl_3$) δ 79.48, 68.16, 56.34, 55.51, 48.94, 41.93, 40.96, 39.59, 39.44, 38.87, 37.19, 37.09, 36.80, 34.34, 33.60, 32.78, 31.25, 30.84, 30.79, 28.56, 27.86, 26.66, 26.46, 26.05, 23.91, 23.15, 18.62, 18.25, 18.17, 16.48, 16.13, 16.02, -3.61, -4.76; ESI-HRMS (*m/z*) calcd for $C_{37}H_{72}NO_3Si$ $[M + NH_4]^+$ 606.5276, found 606.5277.

Aldehyde 5 | To a stirred solution of alcohol **S1** (2.24 g, 3.8 mmol, 1.0 equiv.) in CH_2Cl_2 (38 mL), were added $NaHCO_3$ (958 mg, 11.4 mmol, 3.0 equiv.) and Dess Martin periodinane (4.03 g, 9.5 mmol, 2.5 equiv.). After stirring at 25 °C for 2 hours, the excess reagents were quenched with saturated aq. Na_2SO_3 (50 mL), and the resulting mixture was extracted with EtOAc (3 × 40 mL). The combined organic phases were washed with brine (20 mL), dried over anhydrous Na_2SO_4, filtered, and concentrated under vacuum. The residue was purified by flash column

chromatography (EtOAc/petroleum ether, 1:30) to give **5** (1.38 g, 62%) as a white foam. R_f = 0.5 (silica, PE/EtOAc = 50:1); $[\alpha]_D^{28}$ = -29.0 (*c* 0.9, $CHCl_3$); IR (film) ν_{max} = 3447, 2949, 2855, 1724, 1098, 757 cm^{-1}; ^1H NMR (400 MHz, $CDCl_3$) δ 9.35 (d, *J* = 1.0 Hz, 1H), 3.34 (s, 3H), 3.29–3.21 (m, 1H), 3.15 (dd, *J* = 11.3, 4.7 Hz, 1H), 2.56–2.47 (m, 1H), 2.03–1.97 (m, 1H), 0.95 (s, 3H), 0.94 (s, 3H), 0.89 (s, 3H), 0.88 (s, 12H), 0.84 (s, 3H), 0.82 (s, 3H), 0.72 (s, 3H), 0.63 (d, *J* = 9.3 Hz, 1H), 0.02 (s, 6H); ^{13}C NMR (101 MHz, $CDCl_3$) δ 207.35, 79.48, 76.99, 56.24, 55.50, 49.84, 48.86, 42.70, 41.81, 40.57, 39.57, 38.86, 37.18, 36.04, 33.83, 33.67, 32.81, 30.78, 30.62, 28.57, 28.03, 27.85, 27.72, 26.18, 26.05, 23.40, 21.56, 18.57, 18.25, 17.82, 16.36, 16.09, 16.01, -3.62, -4.76; ESI-HRMS (*m/z*) calcd for $C_{37}H_{70}NO_3Si$ $[M + NH_4]^+$ 604.5119, found 604.5121.

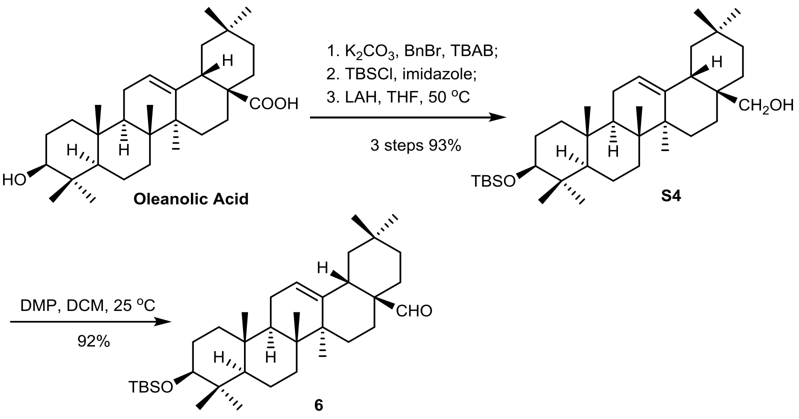

Alcohol S4 | Compound **S4** (22.70 g, 93%) was prepared as a white solid from oleanolic acid (20 g, 43.8 mmol), employing a literature procedure.[15]

Aldehyde 6 | To a stirred solution of **S4** (219 mg, 0.39 mmol, 1.0 equiv.) in CH_2Cl_2 (4.0 mL), were added $NaHCO_3$ (198 mg, 2.36 mmol, 6.0 equiv.) and Dess Martin periodinane (667 mg, 1.57 mmol, 4.0 equiv.). After stirring at 25 °C for 2 hours, the excess reagents were quenched with saturated aq. Na_2SO_3 (5.0 mL), and the resulting mixture was extracted with EtOAc (3 × 5.0 mL). The combined organic phases were washed with brine (4.0 mL), dried over anhydrous Na_2SO_4, filtered, and concentrated under vacuum. The residue was purified by flash column chromatography (EtOAc/petroleum ether, 1:30) to give **6** (200 mg, 92%) as a white foam. R_f = 0.7 (silica, PE/EtOAc = 10:1); $[\alpha]_D^{25}$ = 52.9 (*c* 1.0, $CHCl_3$); IR (film) ν_{max} = 2952, 2926, 1726, 1458, 1254, 769 cm^{-1}; ^1H NMR (500 MHz, $CDCl_3$) δ 9.40 (s, 1H), 5.34 (t, *J* = 3.3 Hz, 1H), 3.18 (dd, *J* = 11.1, 4.5 Hz, 1H), 2.62 (dd, *J* = 13.6, 4.0 Hz, 1H), 1.97 (td, *J* = 13.7, 4.0 Hz, 1H), 1.13 (s, 3H), 0.92 (s, 3H), 0.91 (s, 3H), 0.90 (s, 6H), 0.88 (s, 9H), 0.74 (s, 3H), 0.73 (s, 3H), 0.69 (d, *J* = 11.7 Hz, 1H), 0.03 (s, 6H); ^{13}C NMR (126 MHz, $CDCl_3$) δ 207.69, 143.12, 123.50, 79.61, 55.44, 49.25, 47.75, 45.76, 41.85, 40.59, 39.74, 39.48, 38.65, 37.05, 33.33, 33.24, 32.98, 30.80, 28.71, 27.92, 27.77, 26.89, 26.08, 25.72, 23.61, 23.59, 22.29, 18.65, 18.28, 17.18, 16.27, 15.53, -3.58, -4.74; ESI-HRMS (*m/z*) calcd for $C_{36}H_{62}O_2SiNa$ $[M + Na]^+$ 577.4411, found 577.4413.

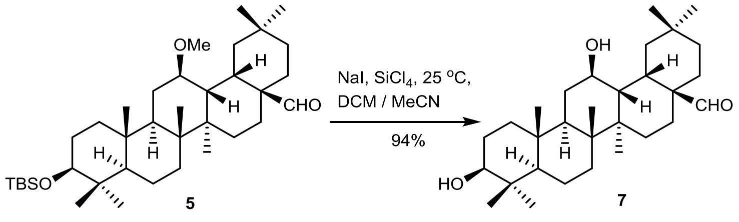

Aldehyde 7 | To a stirred solution of **5** (3.8 g, 6.47 mmol, 1.0 equiv.) in CH_2Cl_2/MeCN (32 mL/32 mL), were added NaI (1.46 g, 9.71 mmol, 1.5 equiv.) and $SiCl_4$ (1.11 mL, 9.71 mmol, 1.5 equiv.).[8] The mixture was stirred at 35 ℃ for 5 hours, the excess reagents were quenched with saturated aq. Na_2SO_3 (30 mL), and the resulting mixture was extracted with EtOAc (3 × 20 mL). The combined organic phases were washed with brine (20 mL), dried over anhydrous Na_2SO_4, filtered, and concentrated under vacuum. The residue was purified by flash column chromatography (EtOAc/petroleum ether, 1:2) to give **7** (2.80 g, 94%) as a white foam. R_f = 0.5 (silica, PE/EtOAc = 1:1); $[\alpha]_D^{28}$ = -32.0 (*c* 1.0, $CHCl_3$); IR (film) ν_{max} = 3403, 2945, 2865, 1716, 1460, 1386, 755 cm^{-1}; ^1H NMR (400 MHz, $CDCl_3$) δ 9.37 (s, 1H), 3.80–3.72 (m, 1H), 3.18 (dd, *J* = 11.4, 4.7 Hz, 1H), 2.62–2.25 (m, 1H), 0.96 (s, 6H), 0.93 (s, 3H), 0.89 (s, 3H), 0.86 (s, 3H), 0.81 (s, 3H), 0.75 (s, 3H), 0.65 (d, *J* = 9.5 Hz, 1H); ^{13}C NMR (126 MHz, $CDCl_3$) δ 207.36, 78.98, 68.19, 55.33, 49.87, 49.15, 43.89, 41.76, 40.79, 38.97, 38.77, 37.10, 35.80, 33.91, 33.56, 32.74, 31.92, 30.59, 30.56, 28.20, 28.15, 27.96, 27.41, 23.32, 21.94, 18.37, 17.69, 16.42, 16.05, 15.51; ESI-HRMS (*m/z*) calcd for $C_{30}H_{50}O_3Na$ $[M + Na]^+$ 481.3652, found 481.3654.

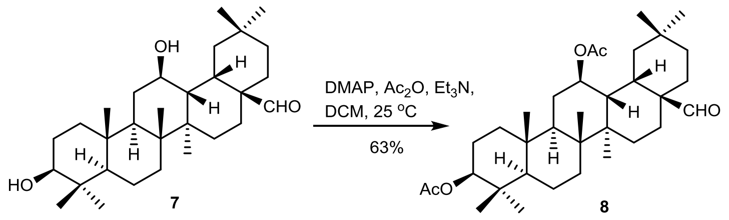

Aldehyde 8 | To a stirred solution of **7** (500 mg, 1.09 mmol, 1.0 equiv.) in CH_2Cl_2 (6.0 mL), were added DMAP (14 mg, 0.11 mmol, 0.1 equiv.), Et_3N (0.61 mL, 4.36 mmol, 4.0 equiv.), and Ac_2O (0.41 mL, 4.36 mmol, 4.0 equiv.). The mixture was stirred at 25 °C for 3 hours, the excess reagents were quenched with saturated aq. $NaHCO_3$ (10 mL), and the resulting mixture was extracted with CH_2Cl_2 (3 × 5 mL). The combined organic phases were washed with brine (10 mL), dried over anhydrous Na_2SO_4, filtered, and concentrated under vacuum. The residue was purified by flash column chromatography (EtOAc/petroleum ether, 1:5) to give **8** (375 mg, 63%) as a white powder. R_f = 0.3 (silica, PE/EtOAc = 6:1); $[\alpha]_D^{28}$ = -32.8 (*c* 0.8, $CHCl_3$); IR (film) ν_{max} = 2949, 2928, 2861, 1730, 1245, 755 cm^{-1}; ^1H NMR (400 MHz, $CDCl_3$) δ 9.36 (d, *J* = 0.8 Hz, 1H), 5.02–4.93 (m, 1H), 4.46 (dd, *J* = 11.2, 4.8 Hz, 1H), 2.32–2.24 (m, 1H), 2.03 (s, 3H), 2.03 (s, 3H), 1.00 (s, 3H), 0.90 (s, 3H), 0.88 (s, 3H), 0.84 (s, 3H), 0.82 (s, 3H), 0.82 (s, 3H), 0.80 (s, 3H); ^{13}C NMR (101 MHz, $CDCl_3$) δ 206.80, 171.06, 170.83, 80.70, 71.70, 55.35, 49.79, 48.67, 41.69, 40.83, 38.39, 37.92, 37.09, 35.79, 33.71, 33.34, 32.50, 30.67, 30.50, 28.10, 28.08, 27.83, 27.40, 23.68, 23.15, 21.69, 21.43, 21.33, 18.20, 17.54, 16.63, 16.33, 16.04; ESI-HRMS (*m/z*) calcd for $C_{34}H_{54}O_5Na$ $[M + Na]^+$ 565.3863, found 565.3870.

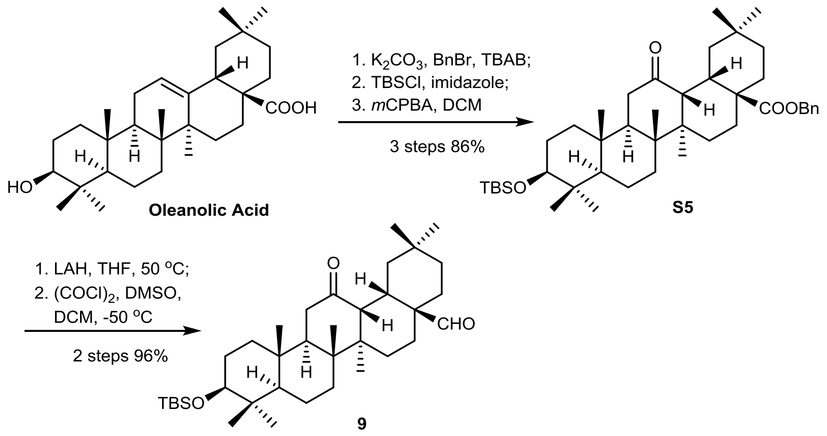

Ketone ester S5 | Compound **S5** (127.4 g, 86%) was prepared as a white solid from oleanolic acid (100 g, 219 mmol), employing a literature procedure.[14]

Aldehyde 9 | To a stirred solution of **S5** (4.80 g, 7.1 mmol, 1.0 equiv.) in THF (58 mL), was added $LiAlH_4$ (4.38 g, 115 mmol, 16.0 equiv.). The mixture was stirred at 50 °C for 2 hours, the excess reagents were quenched with saturated aq. seignette salt solution (50 mL), and the resulting mixture was extracted with EtOAc (3 × 30 mL). The combined organic phases were washed with brine (20 mL), dried over anhydrous Na_2SO_4, filtered, and concentrated under vacuum. To a stirred solution of $(COCl)_2$ (10 mL, 115 mmol, 16.0 equiv.) in CH_2Cl_2 (200 mL) was added DMSO (16.5 mL, 231 mmol, 32.0 equiv.) at -50 °C. After stirring at the same temperature for 30 minutes, the crude product made from **S5** was added into the mixture. After stirring at -50 °C for 2 hours, Et_3N (48 mL, 345 mmol, 48.0 equiv.) was added, and the resulting mixture was stirred at room temperature for additional 30 minutes. Then the mixture was treated with saturated aq. NH_4Cl (30 mL) and extracted with CH_2Cl_2 (3 × 30 mL). The combined organic phases were washed with brine (20 mL), dried over anhydrous Na_2SO_4, filtered, and concentrated under vacuum. The residue was purified by flash column chromatography (EtOAc/petroleum ether, 1:15) to give **9** (3.90 g, 96%) as a white powder. R_f = 0.3 (silica, PE/EtOAc = 20:1); $[\alpha]_D^{25}$ = -26.0 (*c* 1.0, $CHCl_3$); IR (film) ν_{max} = 2952, 2928, 2854, 1694, 1255, 770 cm^{-1}; ^1H NMR (400 MHz, $CDCl_3$) δ 9.33 (s, 1H), 3.15 (dd, *J* = 11.1, 4.4 Hz, 1H), 2.62–2.54 (m, 1H), 2.42 (d, *J* = 4.4 Hz, 1H), 2.29–2.20 (m, 1H), 2.17–2.07 (m, 1H), 0.96 (s, 3H), 0.95 (s, 3H), 0.93 (s, 3H), 0.92 (s, 3H), 0.89 (s, 3H), 0.87 (s, 9H), 0.84 (s, 3H), 0.74 (s, 3H), 0.02 (s, 6H); ^{13}C NMR (101 MHz, $CDCl_3$) δ 211.85, 206.68, 79.23, 55.26, 52.23, 49.92, 49.68, 42.10, 41.53, 39.52, 38.60, 38.06, 36.94, 35.91, 33.88, 33.51, 32.02, 30.82, 30.74, 28.51, 28.01, 27.59, 26.70, 26.03, 23.08, 21.37, 20.50, 18.59, 18.24, 16.15, 16.00, 15.37, -3.61, -4.79; ESI-HRMS (*m/z*) calcd for $C_{36}H_{66}NO_3Si$ $[M + NH_4]^+$ 588.4806, found 588.4806.

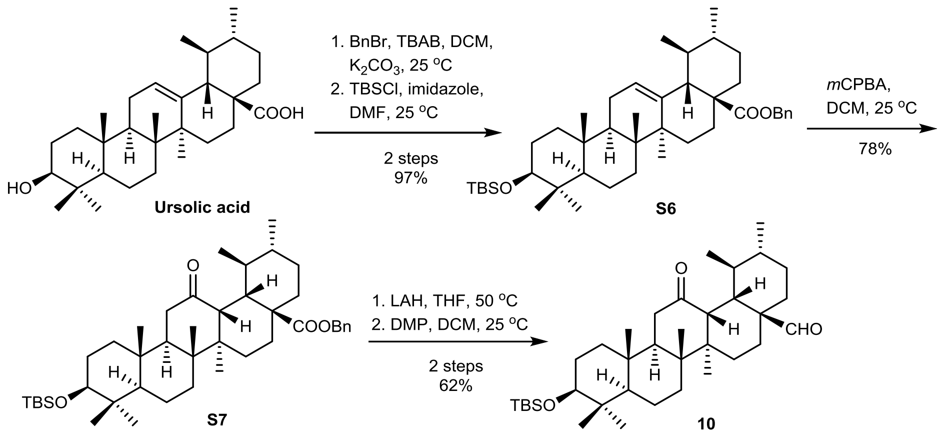

Benzyl ester S6 | To a stirred solution of ursolic acid (10 g, 22 mmol, 1.0 equiv.) in CH_2Cl_2 (44 mL), were added aq. K_2CO_3 (6.1 g, 43.8 mmol/20 mL, 2.0 equiv.), TBAB (5.7 g, 18 mmol, 0.8 equiv.), and benzyl bromide (4.0 ml, 33 mmol, 1.5 equiv.). The mixture was stirred at 25 °C for 3 hours, the excess reagents were quenched with saturated aq. $NaHCO_3$ (30 mL), and the resulting mixture was extracted with CH_2Cl_2 (3 × 30 mL). The combined organic phases were washed with brine (20 mL), dried over anhydrous Na_2SO_4, filtered, and concentrated under vacuum. The residue was dissolved in DMF (55 mL), to which imidazole (4.5 g, 66 mmol) and TBSCl (8.3 g, 55 mmol) were added. The mixture was stirred at 25 °C for 6 hours, the excess reagents were quenched with saturated aq. $NaHCO_3$ (30 mL), and the resulting mixture was extracted with CH_2Cl_2 (3 × 30 mL). The combined organic phases were washed with brine (20 mL), dried over anhydrous Na_2SO_4, filtered, and concentrated under vacuum. The residue was purified by flash column chromatography (EtOAc/petroleum ether, 1:30) to give **S6** (14 g, 97%) as a colorless oil. R_f = 0.5 (silica, PE/EtOAc = 10:1); $[\alpha]_D^{28}$ = -103.0 (*c* 1.0, $CHCl_3$); IR (film) ν_{max} = 3446, 2950, 2927, 1108, 835, 773, 695 cm^{-1}; ^1H NMR (400 MHz, $CDCl_3$) δ 7.38–7.28 (m, 1H), 5.24 (t, *J* = 3.3 Hz, 1H), 5.10 (d, *J* = 12.5 Hz, 1H), 4.98 (d, *J* = 12.5 Hz, 1H), 3.18 (dd, *J* = 11.2, 4.6 Hz, 1H), 2.26 (d, *J* = 11.2 Hz, 1H), 1.07 (s, 3H), 0.94 (d, *J* = 6.1 Hz, 3H), 0.89 (s, 15H), 0.85 (d, *J* = 6.4 Hz, 3H), 0.74 (s, 3H), 0.64 (s, 3H), 0.03 (s, 6H); ^{13}C NMR (101 MHz, $CDCl_3$) δ 177.45, 138.21, 136.52, 128.54, 128.29, 128.07, 125.96, 79.64, 66.12, 55.45, 53.03, 48.27, 47.75, 42.17, 39.68, 39.45, 39.24, 38.98, 38.78, 36.98, 36.78, 33.26, 30.82, 28.71, 28.09, 27.80, 26.07, 24.40, 23.70, 23.44, 21.33, 18.64, 18.27, 17.15, 17.14, 16.29, 15.62, -3.61, -4.76; ESI-HRMS (*m/z*) calcd for $C_{43}H_{69}O_3Si$ $[M + H]^+$ 661.5010, found 661.5007.

Ketoester S7 | To a stirred solution of **S6** (10 g, 15.1 mmol, 1.0 equiv.) in CH_2Cl_2 (100 mL), was added 3-chloroperbenzoic acid (11.8 g, 68.1 mmol, 4.5 equiv.). The mixture was allowed to stir at 25 °C for 8 hours, the excess reagents were quenched with saturated aq. $NaHCO_3$ (100 mL), and the resulting mixture was extracted with EtOAc (3 × 150 mL). The combined organic phases were washed with brine (250 mL), dried over anhydrous Na_2SO_4, and filtered. The volatiles were removed under vacuum, and the residue was purified by flash column chromatography (EtOAc/petroleum ether, 1:15) to give **S7** (8.0 g, 78%) as a colorless oil. R_f = 0.3 (silica, PE/EtOAc = 15:1); $[\alpha]_D^{28}$ = 14.6 (*c* 0.6, $CHCl_3$); IR (film) ν_{max} = 3445, 2951, 2929, 1721, 1687, 835, 756 cm^{-1}; ^1H NMR (400 MHz, $CDCl_3$) δ 7.43–7.27 (m, 1H), 5.19 (d, *J* = 12.3 Hz, 1H), 5.10 (d, *J* = 12.3 Hz, 1H), 3.16 (dd, *J* = 11.1, 4.7 Hz, 1H), 2.81 (dd, *J* = 11.4, 8.5 Hz, 1H), 2.62–2.52 (m, 1H), 2.41–2.34 (m, 1H), 1.31 (s, 3H), 0.91 (s, 3H), 0.90 (s, 6H), 0.88 (s, 9H), 0.76 (s, 3H), 0.69 (d, *J* = 6.0 Hz, 3H), 0.66 (d, *J* = 6.3 Hz, 3H), 0.03 (d, *J* = 1.7 Hz, 6H); ^{13}C NMR (101 MHz, $CDCl_3$) δ

216.51, 178.26, 136.16, 128.61, 128.33, 128.30, 79.23, 66.70, 59.34, 55.46, 50.95, 46.99, 43.81, 43.17, 40.39, 39.90, 39.55, 38.55, 37.24, 36.52, 34.28, 32.07, 31.88, 29.34, 28.50, 27.67, 27.55, 26.02, 25.95, 24.71, 20.20, 20.16, 18.22, 18.11, 17.92, 16.18, 16.06, -3.65, -4.81; ESI-HRMS (*m/z*) calcd for $C_{43}H_{69}O_4Si$ $[M + H]^+$ 677.4960, found 677.4963.

Ketoaldehyde 10 | To a stirred solution of **S7** (3.38 g, 4.99 mmol, 1.0 equiv.) in THF (30 mL), was added $LiAlH_4$ (1.33 g, 34.9 mmol, 7.0 equiv.). The mixture was allowed to stir at 50 ℃ for 3 hours, the excess reagents were quenched with saturated aq. $NaHCO_3$ (100 mL) and the resulting mixture was extracted with EtOAc (3 × 150 mL). The combined organic phases were washed with brine (100 mL), dried over anhydrous Na_2SO_4, and filtered. The volatiles were removed under vacuum, and the residue was dissolved in CH_2Cl_2 (100 mL), to which were added $NaHCO_3$ (958 mg, 34.9 mmol, 7.0 equiv.) and Dess Martin periodinane (4.03 g, 9.5 mmol, 2.0 equiv.). After stirring at 25 °C for 2 hours, the excess reagents were quenched with saturated aq. Na_2SO_3 (50 mL) and the resulting mixture was extracted with EtOAc (3 × 50 mL). The combined organic phases were washed with brine (20 mL), dried over anhydrous Na_2SO_4, filtered, and concentrated under vacuum. The residue was purified by flash column chromatography (EtOAc/petroleum ether, 1:30) to give **10** (1.38 g, 62%) as a white foam. R_f = 0.2 (silica, PE/EtOAc = 50:1); $[\alpha]_D^{28}$ = 53.8 (*c* 0.9, $CHCl_3$); IR (film) ν_{max} = 3447, 2928, 2856, 1717, 1105, 757 cm^{-1}; ^1H NMR (500 MHz, $CDCl_3$) δ 9.50 (s, 1H), 3.19 (dd, *J* = 11.2, 4.2 Hz, 1H), 2.45 (dd, *J* = 10.0, 5.0 Hz, 1H), 2.42–2.33 (m, 1H), 2.29–2.18 (m, 2H), 1.12 (s, 3H), 0.94 (s, 3H), 0.93 (s, 3H), 0.91 (s, 3H), 0.88 (s, 12H), 0.82 (d, *J* = 6.2 Hz, 3H), 0.75 (s, 3H), 0.03 (s, 6H); ^{13}C NMR (126 MHz, $CDCl_3$) δ 216.39, 206.20, 79.35, 56.22, 55.60, 50.08, 49.58, 43.50, 41.72, 39.85, 39.50, 39.26, 39.01, 38.87, 37.42, 36.56, 34.95, 30.02, 29.00, 28.75, 27.69, 26.33, 26.20, 26.04, 24.39, 20.99, 19.29, 19.00, 18.37, 18.25, 16.35, 16.34, -3.63, -4.77; ESI-HRMS (*m/z*) calcd for $C_{36}H_{62}O_3SiNa$ $[M + Na]^+$ 593.4360, found 593.4365.

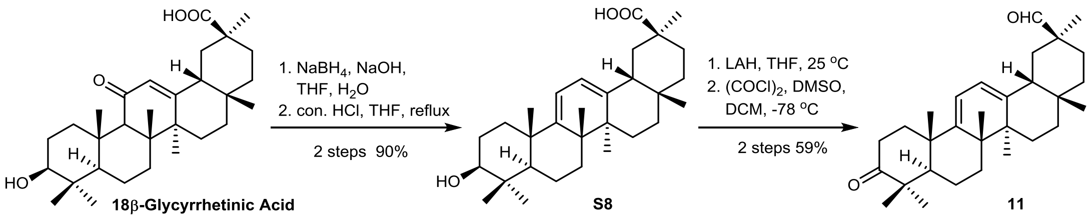

Carboxylic acid S8 | Compound **S8** (1.74 g, 90%) was prepared as a white solid from 18β-glycyrrhetinic acid (2.0 g, 2.12 mmol), employing a literature procedure.[16]

Aldehyde 11 | To a stirred solution of **S8** (1.5 g, 3.3 mmol, 1.0 equiv.) in THF (33 mL), was added $LiAlH_4$ (626 mg, 16.5 mmol, 5.0 equiv.). The mixture was stirred at 25 °C for 2 hours, the excess reagents were quenched with saturated aq. seignette salt solution (50 mL) and the resulting mixture was extracted with EtOAc (3 × 30 mL). The combined organic phases were washed with brine (20 mL), dried over anhydrous $NaSO_4$, filtered, and concentrated under vacuum. To a stirred solution of $(COCl)_2$ (2.0 ml, 34.0 mmol, 10.0 equiv.) in CH_2Cl_2 (44 mL), was added DMSO (3.2 mL, 68.1 mmol, 20.0 equiv.) at -50 ℃. The mixture was stirred at the same temperature for 30 minutes, to which the crude product made from **S8** was added. The resulting mixture was stirred at -50 ℃ for 2 hours before Et_3N (19 mL, 204.2 mmol, 60.0 equiv.) was added, and the stirring continued at 25 °C for 30 minutes. Then the excess reagents were quenched with saturated aq. NH_4Cl (30 mL) and the resulting mixture was extracted with CH_2Cl_2 (3 × 30 mL). The combined

organic phases were washed with brine (20 mL), dried over anhydrous Na_2SO_4, filtered, and concentrated under vacuum. The residue was purified by flash column chromatography (EtOAc/petroleum ether, 1:5) to give **11** (850 mg, 59%) as a white powder. R_f = 0.3 (silica, PE/EtOAc = 10:1); $[\alpha]_D^{28}$ = 212.8 (*c* 1.0, $CHCl_3$); IR (film) ν_{max} = 2964, 2952, 2926, 2867, 1463, 757, 657 cm^{-1}; ^1H NMR (400 MHz, $CDCl_3$) δ 9.46 (s, 1H), 5.65 (d, *J* = 5.8 Hz, 1H), 5.62 (d, *J* = 5.8 Hz, 1H), 2.64–2.54 (m, 1H), 2.52–2.44 (m, 1H), 2.26–2.16 (m, 1H), 1.26 (s, 3H), 1.14 (s, 3H), 1.11 (s, 3H), 1.07 (s, 3H), 1.02 (s, 3H), 0.93 (s, 3H), 0.82 (s, 3H); ^{13}C NMR (101 MHz, $CDCl_3$) δ 217.84, 206.76, 152.95, 146.31, 121.50, 117.58, 51.89, 47.47, 47.24, 46.12, 43.08, 40.60, 40.32, 38.38, 37.91, 37.69, 34.64, 31.84, 31.37, 28.71, 28.39, 27.05, 26.97, 25.73, 25.36, 24.25, 21.43, 20.75, 20.32, 19.68; ESI-HRMS (*m/z*) calcd for $C_{30}H_{45}O_2$ $[M + H]^+$ 437.3414, found 437.3410.

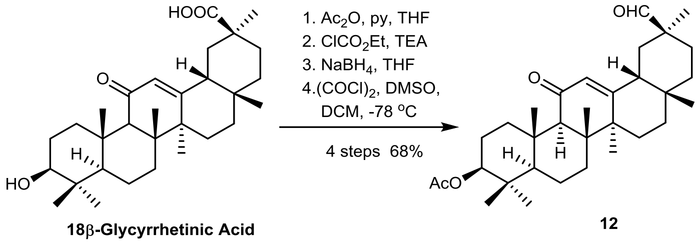

18β-Glycyrrhetinic Acid

12

Aldehyde 12 | Compound **12** (1.8 g, 68%) was prepared as a white solid from 18β-glycyrrhetinic acid (2.5 g, 5.3 mmol), employing a literature procedure.[17]

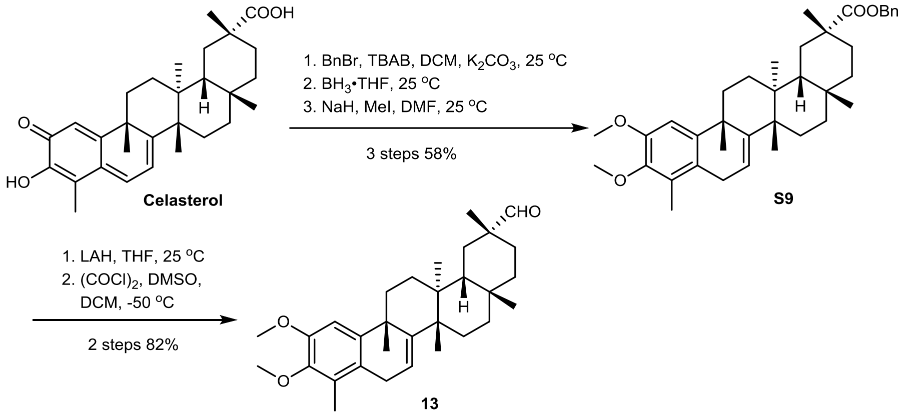

Benzyl ester S9 | To a stirred solution of celasterol (500 mg, 1.11 mmol, 1.0 equiv.) in CH_2Cl_2 (11 mL), were added aq. K_2CO_3 (460 mg, 3.33 mmol/1.5 mL, 3.0 equiv.), TBAB (537 mg, 1.67 mmol, 1.5 equiv.), and BnBr (0.26 mL, 2.22 mmol, 2.0 equiv.). The mixture was stirred at 25 °C for 3 hours, the excess reagents were quenched with saturated aq. $NaHCO_3$ (30 mL) and the resulting mixture was extracted with CH_2Cl_2 (3 × 30 mL). The combined organic phases were washed with brine (20 mL), dried over anhydrous Na_2SO_4, filtered, and concentrated under vacuum. The residue was dissolved in THF (20 mL), to which borane-tetrahydrofuran complex (4.9 ml, 4.84 mmol, 4.3 equiv.) was added at 0 °C. The mixture was stirred at 25 °C for 3 hours, the excess reagents were quenched with saturated aq. Na_2SO_3 (30 mL) and the resulting mixture was extracted with EtOAc (3 × 30 mL). The combined organic phases were washed with brine (20 mL), dried over anhydrous Na_2SO_4, filtered, and concentrated under vacuum. The residue was dissolved in DMF (20 mL), to which 60% NaH (4.9 mL, 4.84 mmol, 4.3 equiv.) and iodomethane (0.3 mL, 4.84 mmol, 4.3 equiv.) were added at 0 °C. The resulting mixture was stirred at 25 °C for 4 hours,

the excess reagents were quenched with saturated aq. Na_2SO_3 (30 mL), and the resulting mixture was extracted with EtOAc (3 × 30 mL). The combined organic phases were washed with brine (20 mL), dried over anhydrous Na_2SO_4, filtered, and concentrated under vacuum. The residue was purified by flash column chromatography (EtOAc/petroleum ether, 1:10) to give **S9** (370 mg, 58%) as an orange oil. R_f = 0.3 (silica, PE/EtOAc = 15:1); $[\alpha]_D^{25}$ = 10.7 (*c* 1.0, $CHCl_3$); IR (film) ν_{max} = 2957, 2869, 1727, 1487, 1458, 756 cm^{-1}; ^1H NMR (400 MHz, $CDCl_3$) δ 7.36–7.27 (m, 5H), 6.75 (s, 1H), 5.77 (dd, *J* = 6.0, 1.8 Hz, 1H), 4.99 (d, *J* = 12.6 Hz, 1H), 4.91 (d, *J* = 12.7 Hz, 1H), 3.87 (s, 3H), 3.77 (s, 3H), 3.32–3.23 (m, 1H), 3.05–2.98 (m, 1H), 2.52–2.45 (m, 1H), 2.17 (s, 3H), 1.33 (s, 3H), 1.22 (s, 3H), 1.20 (s, 3H), 1.08 (s, 3H), 0.66 (s, 3H); ^{13}C NMR (101 MHz, $CDCl_3$) δ 178.43, 151.04, 149.31, 144.98, 144.78, 136.19, 128.56, 128.06, 128.01, 127.98, 125.72, 117.85, 106.46, 66.30, 60.48, 56.04, 44.48, 43.88, 40.68, 37.73, 37.29, 36.97, 34.88, 34.52, 34.27, 33.06, 31.72, 30.90, 30.70, 30.37, 30.05, 29.02, 28.01, 22.92, 18.63, 11.95 ppm; ESI-HRMS (*m/z*) calcd for $C_{38}H_{54}NO_4$ $[M + NH_4]^+$ 588.4047, found 588.4044.

Aldehyde 13 | To a stirred solution of **S9** (370 mg, 0.65 mmol, 1.0 equiv.) in THF (10 mL), was added $LiAlH_4$ (123 mg, 3.24 mmol, 5.0 equiv.). The mixture was stirred at 25 °C for 5 hours, the excess reagents were quenched with saturated aq. seignette salt solution (50 mL) and the resulting mixture was extracted with EtOAc (3 × 30 mL). The combined organic phases were washed with brine (20 mL), dried over anhydrous $NaSO_4$, filtered, and concentrated under vacuum. To a stirred solution of $(COCl)_2$ (0.6 mL, 7.77 mmol, 12.0 equiv.) in CH_2Cl_2 (13 mL), was added DMSO (1.0 mL, 15.5 mmol, 24.0 equiv.) at -50 ℃. The mixture was stirred at the same temperature for 30 minutes, to which the crude product made from **S9** was added. After stirring at -50 ℃ for 2 hours, Et_3N (6.0 ml, 46.7 mmol, 72.0 equiv.) was added and the stirring continued at 25 °C for 30 minutes. Then the excess reagents were quenched with saturated aq. NH_4Cl (30 mL) and the resulting mixture was extracted with CH_2Cl_2 (3 × 30 mL). The combined organic phases were washed with brine (20 mL), dried over anhydrous Na_2SO_4, filtered, and concentrated under vacuum. The residue was purified by flash column chromatography (EtOAc/petroleum ether, 1:10) to give **13** (246 mg, 82%) as an orange oil. R_f = 0.4 (silica, PE/EtOAc = 10:1); $[\alpha]_D^{25}$ = -9.2 (*c* 1.0, $CHCl_3$); IR (film) ν_{max} = 2956, 2868, 1738, 1487, 1091, 755 cm^{-1}; ^1H NMR (400 MHz, $CDCl_3$) δ 9.32 (d, *J* = 1.2 Hz, 1H), 6.75 (s, 1H), 5.76 (dd, *J* = 6.1, 1.8 Hz, 1H), 3.85 (s, 3H), 3.76 (s, 3H), 3.32–3.23 (m, 1H), 3.04–2.96 (m, 1H), 2.17 (s, 3H), 1.34 (s, 2H), 1.23 (s, 3H), 1.09 (s, 2H), 0.94 (s, 3H), 0.60 (s, 3H); ^{13}C NMR (101 MHz, $CDCl_3$) δ 205.45, 151.01, 149.01, 144.96, 144.69, 127.94, 125.71, 118.12, 106.28, 60.45, 55.99, 44.64, 43.95, 43.81, 37.87, 37.25, 36.92, 34.56, 34.39, 31.58, 30.94, 30.62, 29.41, 28.85, 27.96, 27.70, 26.48, 22.91, 20.30, 11.94; ESI-HRMS (*m/z*) calcd for $C_{31}H_{45}O_3$ $[M + H]^+$ 465.3363, found 465.3359.

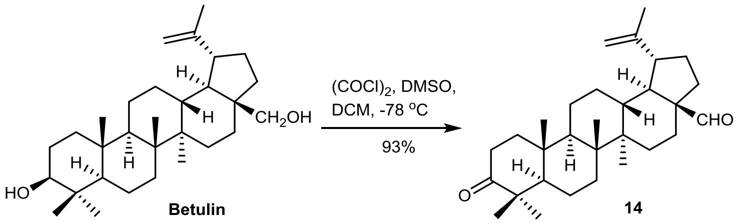

Ketoaldehyde 14 | Compound **14** (185 mg, 93%) was prepared as a white solid from betulin (200 mg, 0.45 mmol), using a literature procedure.[18]

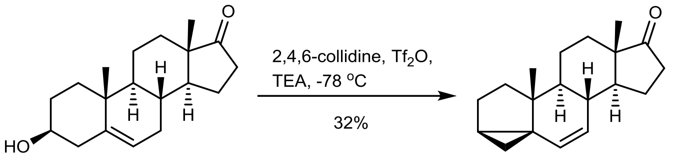

Ketone 15 | Compound **15** (320 mg, 32%) was prepared as a white solid from dehydro-*epi*-andrsterone (1.0 g, 3.47 mmol), using a literature procedure.[19]

Initial tests on C-H oxidations

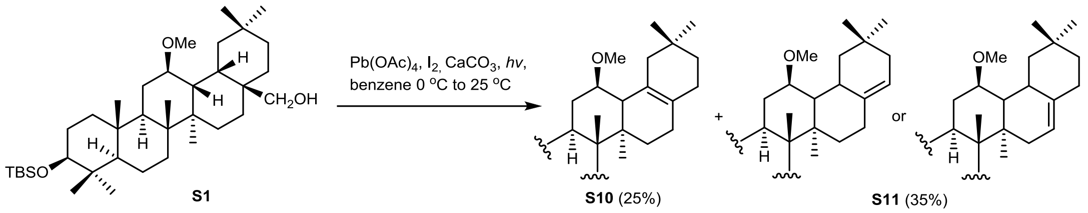

Compound S10 & S11 | To a stirred solution of **S1** (150mg, 0.25 mmol, 1.0 equiv.) in benzene (12 mL), were added $CaCO_3$ (90 mg, 0.90 mmol, 3.6 equiv.), $Pb(OAc)_4$ (159 mg, 0.36 mmol, 1.4 equiv.), and I_2 (66 mg, 0.52 mmol, 2.1 equiv.) at 0 ℃. The mixture was irradiated with an infrared lamp (125 w) for 20 min, and then the excess reagents were quenched with saturated aq. Na_2SO_3 (10 mL). The resulting mixture was extracted with EtOAc (3 × 10 mL). The combined organic phases were washed with brine (20 mL), dried over anhydrous Na_2SO_4, filtered, and concentrated under vacuum. The residue was purified by flash column chromatography (EtOAc/petroleum ether, 1:30) to give **S10** (36 mg, 25%) and **S11** (50 mg, 35%) as white foams.

S10: R_f = 0.3 (silica, PE/EtOAc = 50:1); $[\alpha]_D^{25}$ = -41.5 (*c* 1.0, $CHCl_3$); IR (film) ν_{max} = 2948, 1462, 1257, 1097, 835, 773 cm^{-1}; ^1H NMR (400 MHz, $CDCl_3$) δ 3.28 (s, 3H), 3.17 (dd, *J* = 11.4, 4.7 Hz, 1H), 3.16–3.08 (m, 1H), 2.25–2.10 (m, 2H), 2.05 (ddd, *J* = 7.9, 5.3, 2.2 Hz, 1H), 0.98 (s, 3H), 0.89 (s, 4H), 0.87 (s, 15H), 0.85 (s, 3H), 0.79 (s, 3H), 0.74 (s, 3H), 0.68 (d, *J* = 9.4 Hz, 1H), 0.03 (s, 6H); ^{13}C NMR (126 MHz, $CDCl_3$) δ 129.98, 127.42, 79.56, 79.25, 55.92, 55.00, 48.78, 46.16, 43.17, 41.93, 40.44, 39.61, 38.97, 37.31, 35.18, 34.60, 30.03, 29.93, 29.51, 29.15, 28.70, 28.56, 27.94, 27.45, 26.93, 26.08, 18.56, 18.28, 16.40, 16.31, 16.29, 16.08, -3.60, -4.74 ppm; ESI-HRMS (*m/z*) calcd for $C_{36}H_{64}O_2SiNa$ $[M + Na]^+$ 579.4568, found 579.4569.

S11 (The position of the double bond was not determined): R_f = 0.2 (silica, PE/EtOAc = 50:1); $[\alpha]_D^{25}$ = -33.7 (*c* 1.0, $CHCl_3$); IR (film) ν_{max} = 2949, 2854, 1463, 1257, 773 cm^{-1}; ^1H NMR (400 MHz, $CDCl_3$) δ 5.25 (d, *J* = 4.9 Hz, 1H), 3.33 (s, 3H), 3.29–3.21 (m, 1H), 3.17 (dd, *J* = 11.4, 4.6 Hz, 1H), 2.52–2.40 (m, 1H), 0.98 (s, 3H), 0.96 (s, 3H), 0.91 (s, 3H), 0.90 (s, 3H), 0.89 (s, 3H), 0.89 (s, 9H), 0.87 (s, 3H), 0.73 (s, 3H), 0.66 (d, *J* = 10.3 Hz, 1H), 0.03 (s, 6H); ^{13}C NMR (126 MHz, $CDCl_3$) δ 139.52, 116.39, 79.54, 77.48, 55.65, 55.40, 48.68, 41.43, 41.33, 40.99, 40.60, 40.41, 39.60, 38.96, 37.27, 33.94, 33.71, 33.25, 32.68, 32.46, 31.78, 28.62, 27.91, 26.08, 25.88, 24.19, 18.47, 18.28, 17.31, 16.92, 16.46, 16.14, -3.60, -4.74; ESI-HRMS (*m/z*) calcd for $C_{36}H_{64}O_2SiNa$ $[M + Na]^+$ 579.4568, found 579.4567.

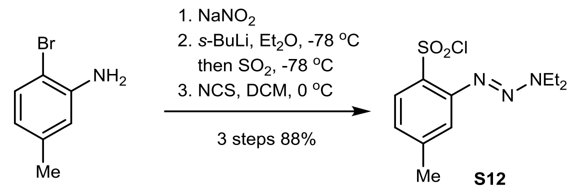

Sulfuryl chloride S12 | Compound **S12** (820 mg, 88%) was prepared as a yellow oil from 2-bromo-5-methylbenzenamine (600 mg, 3.22 mmol), using a literature procedure.[2]

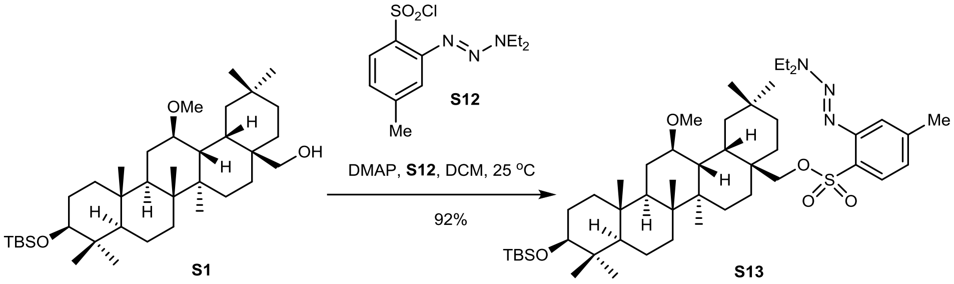

Compound S13 | To a stirred solution of **S1** (200 mg, 0.34 mmol, 1.0 equiv.) and **S12** (246 mg, 0.85 mmol, 2.5 equiv.) in CH_2Cl_2 (2.5 mL), was added DMAP (577 mg, 1.36 mmol, 4.0 equiv.). The mixture was allowed to stir at 25 °C for 3 hours, the excess reagents were quenched with saturated aq. $NaHCO_3$ (10 mL) and the resulting mixture was extracted with EtOAc (3 × 5 mL). The combined organic phases were washed with brine (10 mL), dried over anhydrous Na_2SO_4, filtered, and concentrated under vacuum. The residue was purified by flash column chromatography (EtOAc/petroleum ether, 1:10) to give **S13** (262 mg, 92%) as a yellow oil. R_f = 0.3 (silica, PE/EtOAc = 10:1); $[\alpha]_D^{25}$ = -11.1 (*c* 1.0, $CHCl_3$); IR (film) ν_{max} = 2927, 2855, 1593, 1463, 1176, 835 cm^{-1}; ^1H NMR (400 MHz, $CDCl_3$) δ 7.83 (d, *J* = 8.1 Hz, 1H), 7.31 (s, 1H), 6.99 (d, *J* = 8.0 Hz, 1H), 4.08 (d, *J* = 9.2 Hz, 1H), 3.81 (q, *J* = 7.2 Hz, 4H), 3.53 (d, *J* = 9.2 Hz, 1H), 3.24 (s, 3H), 3.18–3.08 (m, 2H), 2.39 (s, 3H), 2.01–1.91 (m, 1H), 1.80–1.71 (m, 1H), 0.88 (s, 12H), 0.87 (s, 6H), 0.79 (s, 3H), 0.77 (s, 3H), 0.72 (s, 3H), 0.64 (s, 3H), 0.02 (s, 6H); ^{13}C NMR (126 MHz, $CDCl_3$) δ 149.53, 145.18, 131.16, 125.71, 125.31, 118.56, 79.47, 77.05, 76.18, 56.14, 55.51, 49.27, 48.91, 42.31, 41.70, 40.75, 39.79, 39.58, 38.85, 37.15, 36.38, 36.35, 33.92, 33.32, 32.66, 31.86, 31.69, 30.64, 28.55, 27.84, 26.41, 26.31, 26.06, 24.14, 22.07, 21.83, 18.56, 18.26, 18.19, 16.09, 16.05, 16.01, 14.67, 11.59, -3.62, -4.75; ESI-HRMS (*m/z*) calcd for $C_{48}H_{83}N_3O_5SSiNa$ $[M + Na]^+$ 864.5715, found 864.5732.

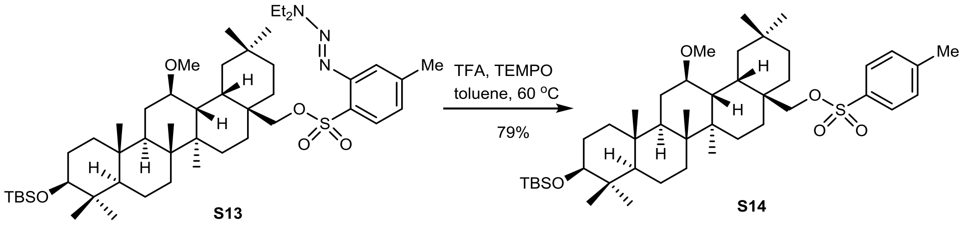

Compound S14 | To a stirred solution of **S13** (100 mg, 0.12 mmol, 1.0 equiv.) and TEMPO (19 mg, 0.12 mmol, 1.0 equiv.) in toluene (4.0 mL), was added TFA (41 mg, 0.36 mmol, 3.0 equiv.). The mixture was allowed to stir at 60 ℃ for 8 hours, the excess reagents were quenched with saturated 1N aq. HCl (10 mL), and the resulting mixture was extracted with EtOAc (3 × 5 mL). The combined organic phases were washed with brine (10 mL), dried over anhydrous Na_2SO_4,

filtered, and concentrated under vacuum. The residue was purified by flash column chromatography (EtOAc/petroleum ether, 1:10) to give **S14** (70 mg, 79%) as a yellow oil. R_f = 0.4 (silica, PE/EtOAc = 9:1); $[\alpha]_D^{25}$ = -4.4 (*c* 1.0, $CHCl_3$); IR (film) ν_{max} = 2927, 2855, 1463, 1362, 1177, 836 cm^{-1}; ^1H NMR (400 MHz, $CDCl_3$) δ 7.78 (d, *J* = 8.2 Hz, 2H), 7.33 (d, *J* = 8.1 Hz, 2H), 4.06 (d, *J* = 9.1 Hz, 1H), 3.61 (d, *J* = 9.1 Hz, 1H), 3.28 (s, 3H), 3.19–3.05 (m, 2H), 2.44 (s, 3H), 2.02–1.95 (m, 1H), 1.91–1.82 (m, 1H), 0.89 (s, 3H), 0.88 (s, 15H), 0.80 (s, 3H), 0.79 (s, 3H), 0.72 (s, 3H), 0.64 (s, 3H), 0.02 (s, 6H); ^{13}C NMR (126 MHz, $CDCl_3$) δ 144.65, 132.87, 129.95, 128.33, 79.38, 76.99, 76.08, 56.27, 55.42, 48.81, 41.68, 40.70, 39.65, 39.55, 38.80, 37.11, 36.41, 36.37, 33.87, 33.34, 32.60, 31.51, 31.31, 30.63, 28.51, 27.80, 26.33, 26.28, 26.04, 23.97, 22.38, 21.78, 18.50, 18.24, 18.15, 16.08, 16.00, -3.63, -4.78; ESI-HRMS (*m/z*) calcd for $C_{44}H_{74}O_5SSiNa$ $[M + Na]^+$ 765.4918, found 765.4921.

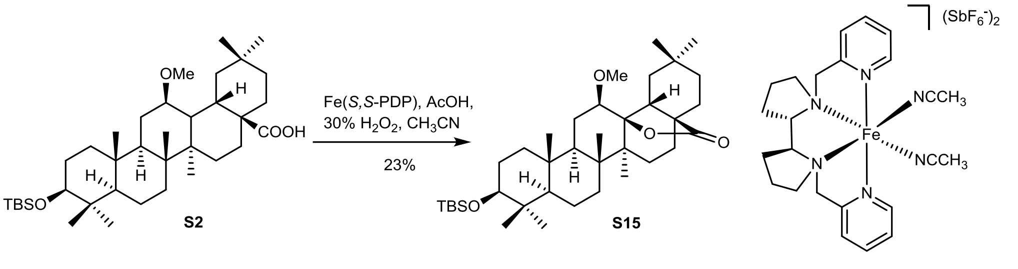

Lactone S15 | To a stirred solution of **S2** (60 mg, 0.10 mmol, 1.0 equiv.) in CH_3CN (0.3 mL), were added Fe(S,S-PDP) (19 mg, 0.02 mmol, 0.2 equiv.) and HOAc (3.0 μL, 0.05 mmol, 0.5 equiv.). The mixture was allowed to stir at 25 °C, and a solution of H_2O_2 (30 wt%, 46 μL, 0.40 mmol, 4.0 equiv.) in CH_3CN (1.0 mL) was added dropwise via syringe over ca. 45-75 seconds. The mixture was allowed to stir at 25 °C for 1 hour, the excess reagents were quenched with saturated aq. $NaHCO_3$ (10 mL), and the resulting mixture was extracted with EtOAc (3 × 15 mL). The combined organic phases were washed with brine (25 mL), dried over anhydrous Na_2SO_4, filtered, and concentrated under vacuum. The residue was purified by flash column chromatography (EtOAc/petroleum ether, 1:9) to give **S15** (14 mg, 23%) as a white foam. R_f = 0.3 (silica, PE/EtOAc = 15:1); $[\alpha]_D^{25}$ = 13.8 (*c* 1.0, $CHCl_3$); IR (film) ν_{max} = 2931, 2857, 1762, 1463, 1105, 835 cm^{-1}; ^1H NMR (400 MHz, $CDCl_3$) δ 3.40 (s, 3H), 3.28 (dd, *J* = 11.7, 5.5 Hz, 1H), 3.16 (dd, *J* = 11.3, 4.4 Hz, 1H), 2.73–2.66 (m, 1H), 2.10–2.00 (m, 1H), 1.15 (s, 3H), 1.05 (s, 3H), 0.99 (s, 3H), 0.91 (s, 3H), 0.88 (s, 15H), 0.73 (s, 3H), 0.64 (d, *J* = 8.9 Hz, 1H), 0.03 (s, 6H); ^{13}C NMR (101 MHz, $CDCl_3$) δ 179.63, 92.54, 79.41, 76.32, 58.31, 55.26, 49.19, 43.84, 42.70, 42.56, 42.11, 39.58, 39.15, 37.82, 36.99, 34.43, 33.62, 33.46, 31.85, 28.49, 27.79, 27.51, 27.34, 26.03, 24.05, 23.66, 20.64, 19.54, 18.63, 18.23, 17.98, 16.43, 15.92, -3.62, -4.78; ESI-HRMS (*m/z*) calcd for $C_{37}H_{64}O_4SiNa$ $[M + Na]^+$ 623.4466, found 623.4472.

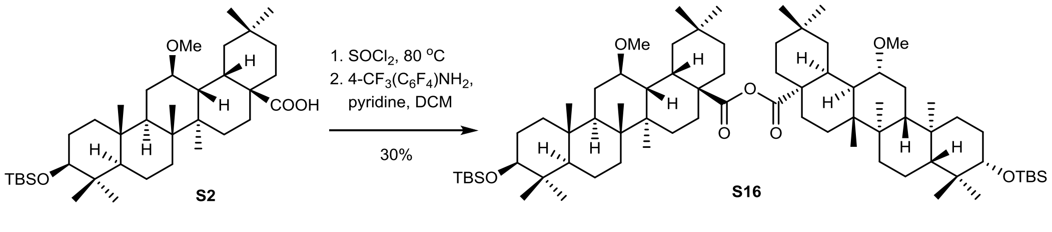

Compound S16 | Acid **S2** (100 mg, 0.17 mmol, 1.0 equiv.) was dissolved in $SOCl_2$ (5.0 mL) and the mixture was allowed to stir at 50 ℃ for 2 hours. After removal of $SOCl_2$ under vacuum, the residue was dissolved in CH_2Cl_2 (2.0 mL), to which 4-$CF_3(C_6F_4)NH_2$ (20 μL, 0.18 mmol, 1.1 equiv.) and pyridine (54 μL, 0.66 mmol, 3.9 equiv.) were added. The resulting mixture was stirred at 25 °C for 8 hours, and the excess reagents were quenched with saturated aq. $NaHCO_3$ (10 mL), and the resulting mixture was extracted with EtOAc (3 × 5 mL). The combined organic phases were washed with brine (10 mL), dried over anhydrous Na_2SO_4, filtered, and concentrated under vacuum. The residue was purified by flash column chromatography (EtOAc/petroleum ether, 1:15) to give **S16** (59 mg, 30%) as a white foam. R_f = 0.3 (silica, PE/EtOAc = 10:1); $[\alpha]_D^{25}$ = 5.6 (*c* 1.0, $CHCl_3$); IR (film) ν_{max} = 2949, 1799, 1731, 1463, 1257, 835 cm^{-1}; ^1H NMR (500 MHz, $CDCl_3$) δ 3.28 (s, 3H), 3.25–3.19 (m, 1H), 3.15 (dd, *J* = 11.4, 4.5 Hz, 1H), 2.80–2.74 (m, 1H), 0.94 (s, 3H), 0.92 (s, 3H), 0.90 (s, 6H), 0.88 (s, 12H), 0.81 (s, 3H), 0.73 (s, 3H), 0.63 (d, *J* = 10.2 Hz, 1H), 0.02 (s, 6H); ^{13}C NMR (126 MHz, $CDCl_3$) δ 174.07, 79.50, 76.64, 55.77, 55.53, 49.08, 48.98, 41.73, 41.59, 40.53, 39.57, 38.85, 37.17, 36.49, 34.53, 33.62, 32.72, 32.43, 31.30, 30.71, 28.84, 28.58, 27.88, 26.16, 26.06, 23.50, 23.27, 18.59, 18.26, 18.07, 16.50, 16.04, 16.02, -3.60, -4.76.

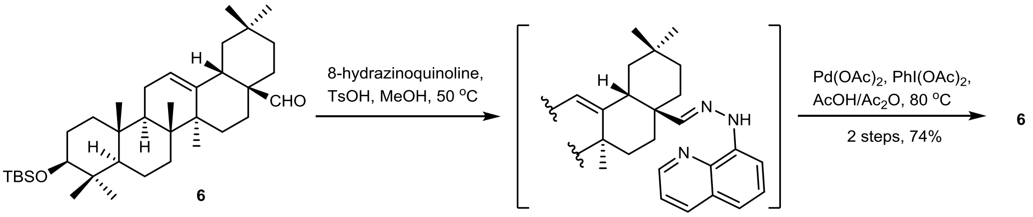

Compound 6 | To a stirred solution of aldehyde **6** (400 mg, 0.70 mmol, 1.0 equiv.) in MeOH (6.0 mL), were added 8-hydrazinoline (120 mg, 0.77 mmol, 1.1 equiv.) and *p*-toluenesulfonic acid monohydrate (6.0 mg, 0.03 mmol, 0.04 equiv.). The mixture was allowed to stir at 50 ℃ for 8 hours, the excess reagents were quenched with saturated aq. $NaHCO_3$ (20 mL) and the resulting mixture was extracted with EtOAc (3 × 15 mL). The combined organic phases were washed with brine (25 mL), dried over anhydrous Na_2SO_4, filtered, and concentrated under vacuum. The residue was dissolved in toluene (2.0 mL), to which $Pd(OAc)_2$ (186 mg, 0.83 mmol, 1.2 equiv.), $PhI(OAc)_2$ (445 mg, 1.38 mmol, 2.0 equiv.), AcOH (3.5 mL), and Ac_2O (3.5 mL) were added. The mixture was stirred at 80 ℃ for 8 hours, the excess reagents were quenched with saturated aq. $NaHCO_3$ (5.0 mL), and the resulting mixture was extracted with EtOAc (3 × 5 mL). The combined organic phases were washed with brine (5.0 mL), dried over anhydrous Na_2SO_4, filtered, and concentrated under vacuum. The residue was purified by flash column chromatography (EtOAc/petroleum ether, 1:40) to give **6** (300 mg, 76%) as a white foam. The hydrazone was hydrolyzed under the oxidation conditions.

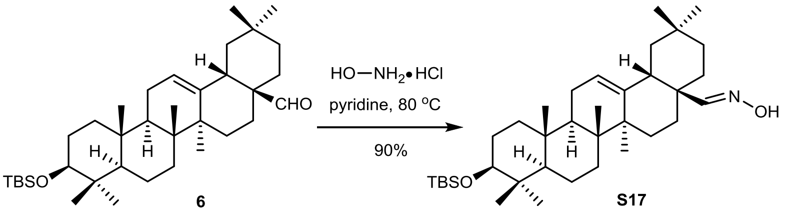

Compound S17 | To a stirred solution of aldehyde **6** (100 mg, 0.18 mmol, 1.0 equiv.) in pyridine

(4.0 mL), was added hydroxylamine hydrochloride (20 mg, 0.27 mmol, 1.5 equiv.). The mixture was allowed to stir at 80 ℃ for 2 hours before it was concentrated under vacuum. The residue was purified by flash column chromatography (EtOAc/petroleum ether, 1:15) to give **S17** (92 mg, 90%) as a white foam. R_f = 0.4 (silica, PE/EtOAc = 10:1); $[\alpha]_D^{25}$ = 85.0 (*c* 1.0, $CHCl_3$); IR (film) ν_{max} = 3295, 2927, 2855, 1462, 1250, 908, 735 cm^{-1}; ^1H NMR (400 MHz, $CDCl_3$) δ 5.25 (s, 1H), 3.22 (dd, *J* = 11.1, 4.4 Hz, 1H), 2.54–2.46 (m, 1H), 2.20–2.09 (m, 1H), 1.17 (s, 3H), 0.95 (s, 3H), 0.94 (s, 9H), 0.92 (s, 9H), 0.86 (s, 3H), 0.78 (s, 3H), 0.07 (s, 6H); ^{13}C NMR (101 MHz, $CDCl_3$) δ 159.15, 143.79, 122.95, 79.61, 77.48, 77.16, 76.84, 55.40, 47.82, 46.07, 42.90, 41.72, 39.90, 39.47, 38.61, 37.03, 34.08, 33.29, 32.99, 32.87, 30.87, 28.67, 27.77, 26.91, 26.18, 26.08, 24.98, 23.84, 23.67, 18.63, 18.27, 17.28, 16.19, 15.61, -3.59, -4.75; ESI-HRMS (*m/z*) calcd for $C_{36}H_{64}NO_2Si$ $[M + H]^+$ 570.4701, found 570.4702.

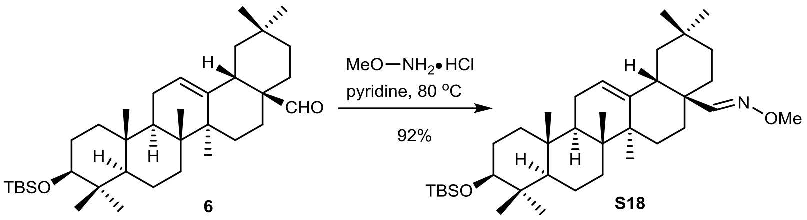

Compound S18 | To a stirred solution of aldehyde **6** (50 mg, 0.09 mmol, 1.0 equiv.) in pyridine (3.0 mL), was added methoxyammonium chloride (12 mg, 0.14 mmol, 1.5 equiv.). The mixture was allowed to stir at 80 ℃ for 2 hours before it was concentrated under vacuum. The residue was purified by flash column chromatography (EtOAc/petroleum ether, 1:60) to give **S18** (48 mg, 92%) as a white foam. R_f = 0.6 (silica, PE/EtOAc = 50:1); $[\alpha]_D^{25}$ = 81.6 (*c* 1.0, $CHCl_3$); IR (film) ν_{max} = 2856, 1462, 1360, 1254, 835, 741 cm^{-1}; ^1H NMR (400 MHz, $CDCl_3$) δ 5.29 (s, 1H), 3.83 (s, 3H), 3.23 (dd, *J* = 11.0, 4.4 Hz, 1H), 2.43–2.35 (m, 1H), 2.17–2.08 (m, 1H), 1.19 (s, 3H), 0.97 (s, 3H), 0.95 (s, 9H), 0.94 (s, 9H), 0.91 (s, 3H), 0.80 (s, 3H), 0.09 (s, 6H); ^{13}C NMR (101 MHz, $CDCl_3$) δ 158.69, 143.92, 122.97, 79.59, 61.26, 55.41, 47.80, 45.99, 44.06, 41.70, 40.05, 39.47, 39.44, 38.62, 37.02, 34.00, 33.36, 32.86, 32.83, 30.84, 28.69, 27.77, 26.67, 26.27, 26.07, 24.57, 23.79, 23.67, 18.65, 18.26, 17.24, 16.27, 15.53, -3.58, -4.75; ESI-HRMS (*m/z*) calcd for $C_{37}H_{66}NO_2Si$ $[M + H]^+$ 584.4857, found 584.4860.

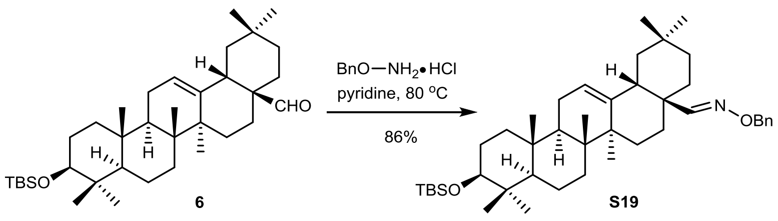

Compound S19 | To a stirred solution of aldehyde **6** (50 mg, 0.09 mmol, 1.0 equiv.) in pyridine (3.0 mL), was added benzyloxyammonium chloride (22 mg, 0.14 mmol, 1.5 equiv.). The mixture was allowed to stir at 80 ℃ for 2 hours before it was concentrated under vacuum. The residue was purified by flash column chromatography (EtOAc/petroleum ether, 1:60) to give **S19** (51 mg, 86%) as a white foam. R_f = 0.4 (silica, PE/EtOAc = 50:1); $[\alpha]_D^{25}$ = 51.0 (*c* 1.0, $CHCl_3$); IR (film) ν_{max} = 3283, 2853, 1650, 1075, 834, 772 cm^{-1}; ^1H NMR (400 MHz, $CDCl_3$) δ 7.39–7.27 (m, 5H), 5.23–5.19 (m, 1H), 5.01 (s, 2H), 3.19 (dd, *J* = 11.0, 4.2 Hz, 1H), 2.41–2.34 (m, 1H), 1.12 (s, 3H), 0.90

(s, 21H), 0.76 (s, 3H), 0.69 (s, 3H), 0.04 (s, 6H); ^{13}C NMR (101 MHz, $CDCl_3$) δ 158.96, 143.94, 138.11, 128.83, 128.44, 127.88, 122.98, 79.64, 77.52, 77.20, 76.89, 75.75, 55.44, 47.85, 46.03, 43.98, 41.73, 39.96, 39.78, 39.50, 38.65, 37.05, 34.09, 33.39, 33.22, 32.86, 30.88, 28.74, 27.81, 26.75, 26.25, 26.12, 24.61, 23.86, 23.69, 18.65, 18.31, 17.17, 16.32, 15.56, -3.54, -4.71; ESI-HRMS (*m/z*) calcd for $C_{43}H_{70}NO_2Si$ [M + H]$^+$ 660.5170, found 660.5175.

Schönecker-Baran oxidation for the site-selective hydroxylation

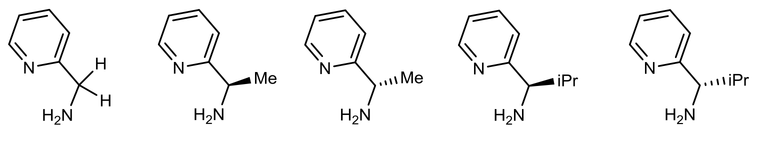

Amines D1-D3 | Compounds **D3(*R*)** (380 mg, 19%) and **D3(*S*)** (358 mg, 18%) were prepared as yellow oils from 2-methyl-1-(pyridin-2-yl)propan-1-one (2.03 g, 13.4 mmol), using a literature procedure.[20] Compounds **D1**, **D2(*R*)**, and **D2(*S*)** were purchased from Shanghai Haohong Scientific Co., Ltd.

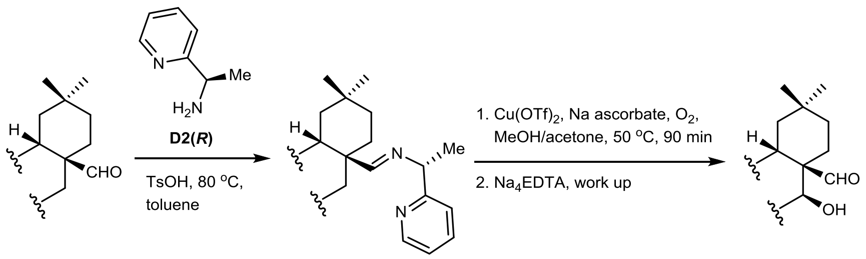

Standard procedure for imine formation.[19,21] To a solution of the aldehyde or ketone substrate and *p*-toluenesulfonic acid monohydrate (0.10 equiv.) in toluene (0.10 M) in a flame-dried flask, was added amine **D2(*S*)** (3.0 equiv.). The mixture was heated to 80 ℃ until imine formation was complete as monitored by ^{1}H NMR (normally *ca.* 2 h for aldehyde and 10 h for ketone). The mixture was cooled to 25 °C and diluted with EtOAc (30 mL). The organic layer was washed sequentially with saturated aqueous NH_4Cl (2 × 20 mL), saturated $NaHCO_3$ (1 × 20 mL), and brine (1 × 20 mL), and was then dried over anhydrous Na_2SO_4, filtered, and concentrated under vacuum.

Standard procedure for Schönecker-Baran oxidation.[19,21] The imine substrate (1.0 equiv), copper(II) triflate (1.3 equiv.), and sodium L-ascorbate (2.0 equiv.) were added to a round-bottom flask. Acetone (0.05 M) and methanol (0.05 M) were added at 25 °C. The mixture was stirred for 5 min (and the reaction mixture may turn brown). O_2 from a balloon was bubbled through the mixture for 5 min (resulting in a blue/green solution), and then the mixture was heated to 50 ℃ under an O_2 atmosphere for 1.5 h. The mixture was cooled to 25 °C, EtOAc (3 mL) and saturated aqueous Na_4EDTA (6.0 mL, pH ~10) were added and the stirring continued for 0.5 h. The layers were then separated. The aqueous layer was extracted with EtOAc (3 × 10 mL). The combined organic phase was washed sequentially with saturated $NaHCO_3$ (1 × 20 mL) and brine (1 × 20

mL), and was then dried over anhydrous Na_2SO_4, filtered, and concentrated under vacuum. The crude product was purified by flash column chromatography.

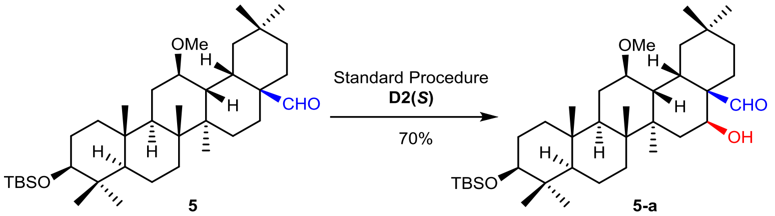

5 **5-a**

Compound 5-a | This compound was prepared from substrate **5** (106 mg, 0.17 mmol, 1.0 equiv.) following the standard procedure in the presence of **D2(*S*)** (104 mg, 0.85 mmol, 3.0 equiv.). The residue was purified by flash column chromatography (EtOAc/petroleum ether, 1:5) to afford product **5-a** (76 mg, 70%) as a white foam. R_f = 0.5 (silica, PE/EtOAc = 5:1); $[\alpha]_D^{28}$ = -62.9 (*c* 0.5, $CHCl_3$); IR (film) ν_{max} = 3421, 2946, 2857, 1709, 1462, 772 cm^{-1}; ^1H NMR (500 MHz, $CDCl_3$) δ 9.40 (d, *J* = 2.2 Hz, 1H), 3.99–3.93 (m, 1H), 3.34 (s, 3H), 3.28–3.20 (m, 1H), 3.16 (dd, *J* = 11.2, 4.4 Hz, 1H), 2.67–2.61 (m, 1H), 2.05–1.99 (m, 1H), 1.95–1.90 (m, 1H), 1.82–1.75 (m, 1H), 1.02 (s, 3H), 0.95 (s, 3H), 0.93 (s, 3H), 0.89 (s, 6H), 0.88 (s, 9H), 0.83 (s, 3H), 0.73 (s, 3H), 0.03 (s, 6H); ^{13}C NMR (126 MHz, $CDCl_3$) δ 209.63, 79.44, 76.86, 66.10, 56.42, 55.50, 53.65, 48.63, 43.55, 43.21, 42.00, 39.60, 38.88, 37.73, 37.19, 35.98, 33.67, 33.28, 32.92, 32.79, 30.44, 28.59, 27.84, 26.17, 26.06, 23.36, 21.85, 18.87, 18.55, 18.26, 16.45, 16.08, 16.02, -3.61, -4.75; ESI-HRMS (*m/z*) calcd for $C_{37}H_{70}NO_4Si$ $[M + NH_4]^+$ 620.5069, found 620.5076.

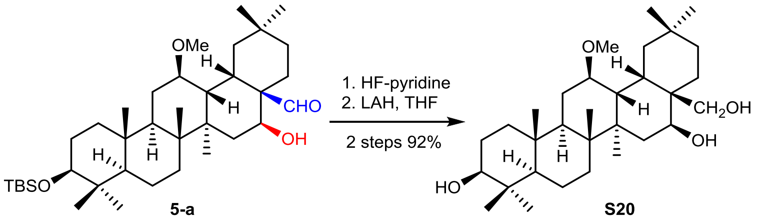

5-a **S20**

Compound S20 | To a stirred solution of **5-a** (120 mg, 0.20 mmol, 1.0 equiv.) in THF (3.0 mL), was added hydrogen fluoride-pyridine (70% HF, 0.5 mL) at 25 °C. After stirring at 25 °C for 8 h, the excess reagents were quenched with saturated aq. $NaHCO_3$ (20 mL), and the resulting mixture was extracted with EtOAc (3 × 10 mL). The combined organic phases were washed with brine (10 mL), dried over anhydrous Na_2SO_4, filtered, and concentrated under vacuum. The residue was dissolved in THF (2.0 mL) and was added LAH (38 mg, 0.99 mmol) at 0 °C. The mixture was stirred at 25 °C for 2 hours, the excess reagents were quenched with water and 15% NaOH (5.0 mL and 5.0 mL), and the resulting mixture was then extracted with EtOAc (3 × 10 mL). The combined organic phases were washed with brine (20 mL), dried over anhydrous Na_2SO_4, filtered, and concentrated under vacuum. The residue was purified by flash column chromatography (EtOAc/petroleum ether, 1:1) to give **S20** (90 mg, 92%) as a white solid. R_f = 0.5 (silica, PE/EtOAc = 1:1); $[\alpha]_D^{28}$ = -43.2 (*c* 1.0, $CHCl_3$); mp 256.1–258.4 °C; IR (film) ν_{max} = 3385, 2947, 1465, 1388, 1029, 756 cm^{-1}; ^1H NMR (400 MHz, $CDCl_3$) δ 4.36 (d, *J* = 10.9 Hz, 1H), 4.15 (dd, *J* = 11.3, 4.2 Hz, 1H), 3.36–3.31 (m, 1H), 3.30 (s, 3H), 3.23–3.14 (m, 2H), 1.06 (s, 3H), 1.02 (s, 3H), 0.98 (s, 3H), 0.92 (s, 3H), 0.87 (s, 3H), 0.86 (s, 3H), 0.77 (s, 3H); ^{13}C NMR (126 MHz, $CDCl_3$) δ 78.98, 77.19, 69.75, 69.65, 56.36, 55.48, 48.75, 43.47, 41.99, 40.65, 39.81, 39.04, 38.89, 37.32,

37.21, 36.92, 34.31, 33.66, 33.25, 32.78, 30.68, 28.16, 27.42, 26.40, 26.18, 24.42, 19.35, 18.44, 16.53, 16.14, 15.50; ESI-HRMS (*m/z*) calcd for $C_{31}H_{53}O_3$ $[M + H]^+$ 473.3989, found 473.3989.

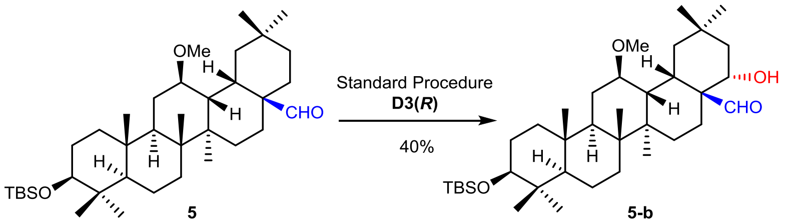

Compound 5-b | This compound was prepared from substrate **5** (100 mg, 0.17 mmol, 1.0 equiv.) following the standard procedure in the presence of **D3(*R*)** (77 mg, 0.51 mmol, 3.0 equiv.). The residue was purified by flash column chromatography (EtOAc/petroleum ether, 1:5) to afford **5b** (50 mg, 49%) as a white foam. R_f = 0.3 (silica, PE/EtOAc = 5:1); $[\alpha]_D^{28}$ = -42.7 (*c* 0.7, $CHCl_3$); IR (film) ν_{max} = 3446, 2950, 2855, 1722, 1098, 757 cm^{-1}; ^1H NMR (500 MHz, $CDCl_3$) δ 9.55 (s, 1H), 3.83 (dd, *J* = 12.0, 4.5 Hz, 1H), 3.34 (s, 3H), 3.29–3.22 (m, 1H), 3.16 (dd, *J* = 11.3, 4.3 Hz, 1H), 2.58–2.51 (m, 1H), 2.05–2.00 (m, 1H), 1.95–1.90 (m, 1H), 0.99 (s, 3H), 0.97 (s, 3H), 0.95 (s, 3H), 0.88 (s, 12H), 0.85 (s, 3H), 0.82 (s, 3H), 0.72 (s, 3H), 0.03 (s, 6H); ^{13}C NMR (126 MHz, $CDCl_3$) δ 208.23, 79.47, 77.19, 67.00, 56.35, 55.48, 48.84, 42.67, 42.22, 42.00, 41.15, 39.59, 38.86, 37.19, 35.62, 33.72, 32.74, 31.54, 31.37, 28.58, 27.85, 27.23, 26.22, 26.06, 24.48, 18.56, 18.26, 18.04, 16.39, 16.05, 16.02, 15.00, -3.61, -4.75; ESI-HRMS (*m/z*) calcd for $C_{37}H_{70}NO_4Si$ $[M + NH_4]^+$ 620.5069, found 620.5072.

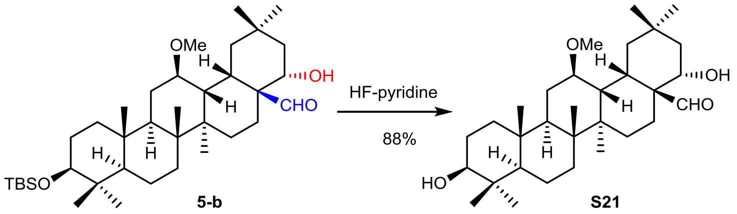

Compound S21 | To a stirred solution of **5-b** (70 mg, 0.12 mmol, 1.0 equiv.) in THF (2.0 mL), was added hydrogen fluoride-pyridine (70% HF, 0.5 mL) at 25 °C. After stirring at 25 °C for 8 h, the excess reagents were quenched with saturated aq. $NaHCO_3$ (20 mL), and the resulting mixture was extracted with EtOAc (3 × 10 mL). The combined organic phases were washed with brine (10 mL), dried over anhydrous Na_2SO_4, filtered, and concentrated under vacuum. The residue was purified by flash column chromatography (EtOAc/petroleum ether, 1:1) to give **S21** (50 mg, 88%) as a white solid. R_f = 0.2 (silica, PE/EtOAc = 1:1); $[\alpha]_D^{28}$ = -52.9 (*c* 1.1, $CHCl_3$); mp 101.8–104.3 ℃; IR (film) ν_{max} = 3447, 2948, 2872, 1719, 1088, 755 cm^{-1} ; ^1H NMR (400 MHz, $CDCl_3$) δ 9.53 (d, *J* = 1.3 Hz, 1H), 3.82 (dd, *J* = 12.0, 4.7 Hz, 1H), 3.33 (s, 3H), 3.30–3.22 (m, 1H), 3.18 (dd, *J* = 11.4, 4.8 Hz, 1H), 2.57–2.49 (m, 1H), 2.04–1.98 (m, 1H), 1.95–1.88 (m, 1H), 0.97 (s, 3H), 0.96 (s, 3H), 0.96 (s, 3H), 0.94 (s, 3H), 0.84 (s, 3H), 0.81 (s, 3H), 0.75 (s, 3H); ^{13}C NMR (101 MHz, $CDCl_3$) δ 208.21, 78.95, 77.08, 66.94, 56.37, 55.47, 55.32, 48.72, 42.62, 42.19, 41.92, 41.10, 38.97, 38.79, 37.23, 35.56, 33.68, 32.58, 31.44, 31.32, 28.12, 27.34, 27.16, 26.16, 24.44, 18.31, 18.03, 16.34, 15.96, 15.47, 14.92; ESI-HRMS (*m/z*) calcd for $C_{31}H_{56}NO_4$ $[M + NH_4]^+$ 506.4204, found 506.4211.

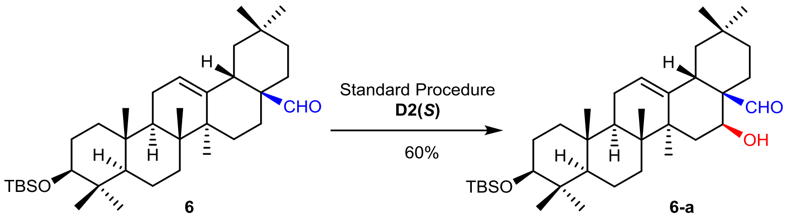

Compound 6-a | This compound was prepared from substrate **6** (2.0 g, 3.60 mmol, 1.0 equiv.) following the standard procedure in the presence of **D2(*S*)** (1.3 g, 10.8 mmol, 3.0 equiv.). The residue was purified by flash column chromatography (EtOAc/petroleum ether, 1:5) to afford product **6-a** (1.26 g, 60%) as a white foam. R_f = 0.3 (silica, PE/EtOAc = 5:1); $[\alpha]_D^{25}$ = 38.6 (*c* 1.0, $CHCl_3$); IR (film) ν_{max} = 3439, 2925, 2854, 1713, 1257, 835 cm^{-1}; ^1H NMR (400 MHz, $CDCl_3$) δ 9.46 (d, *J* = 2.7 Hz, 1H), 5.39 (t, *J* = 3.5 Hz, 1H), 4.17 (ddd, *J* = 11.8, 4.6, 2.6 Hz, 1H), 3.17 (dd, *J* = 11.1, 4.5 Hz, 1H), 2.75–2.68 (m, 1H), 1.19 (s, 3H), 0.95 (s, 3H), 0.94 (s, 3H), 0.90 (s, 6H), 0.88 (s, 9H), 0.77 (s, 3H), 0.74 (s, 3H), 0.72–0.65 (m, 1H), 0.03 (s, 6H); ^{13}C NMR (101 MHz, $CDCl_3$) δ 210.09, 141.88, 124.24, 79.51, 65.86, 55.38, 52.65, 46.85, 45.38, 43.97, 43.38, 39.85, 39.47, 38.61, 36.94, 36.83, 33.24, 32.86, 32.55, 30.56, 28.69, 27.72, 26.72, 26.06, 23.68, 23.63, 21.83, 18.58, 18.26, 17.24, 16.25, 15.51, -3.60, -4.76; ESI-HRMS (*m/z*) calcd for $C_{36}H_{62}O_3SiNa$ $[M + Na]^+$ 593.4360, found 593.4363.

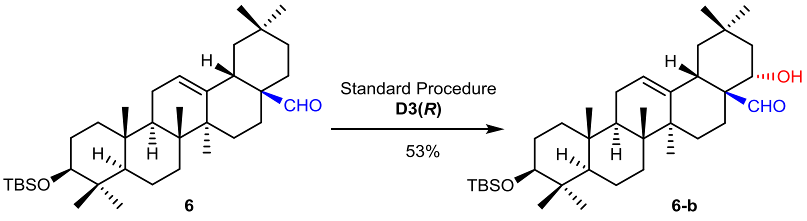

Compound 6-b | This compound was prepared from substrate **6** (20 mg, 0.04 mmol, 1.0 equiv.) following the standard procedure in the presence of **D3(*R*)** (17 mg, 0.11 mmol, 3.0 equiv.). The residue was purified by flash column chromatography (EtOAc/petroleum ether, 1:5) to afford product **6-b** (11 mg, 53%) as a white foam. R_f = 0.4 (silica, PE/EtOAc = 5:1); $[\alpha]_D^{25}$ = 27.0 (*c* 0.5, $CHCl_3$); IR (film) ν_{max} = 3265, 2926, 2854, 1718, 1259, 805 cm^{-1}; ^1H NMR (500 MHz, $CDCl_3$) δ 9.72 (d, *J* = 0.6 Hz, 1H), 5.38 (t, *J* = 3.6 Hz, 1H), 3.80 (dd, *J* = 6.4, 6.0 Hz, 1H), 3.18 (dd, *J* = 11.2, 4.5 Hz, 1H), 2.83–2.58 (m, 1H), 1.17 (s, 3H), 0.97 (s, 3H), 0.96 (s, 3H), 0.91 (s, 3H), 0.90 (s, 3H), 0.88 (s, 9H), 0.76 (s, 3H), 0.74 (s, 3H), 0.73–0.66 (m, 1H), 0.03 (s, 6H); ^{13}C NMR (126 MHz, $CDCl_3$) δ 210.94, 141.77, 124.62, 79.57, 68.04, 55.40, 54.06, 47.67, 45.15, 42.41, 41.77, 41.57, 39.96, 39.49, 38.66, 37.01, 33.25, 32.82, 31.43, 28.69, 27.75, 26.28, 26.07, 25.96, 24.88, 23.69, 18.61, 18.28, 17.12, 16.38, 16.26, 15.53, -3.59, -4.74; ESI-HRMS (*m/z*) calcd for $C_{36}H_{62}O_3SiNa$ $[M + Na]^+$ 593.4360, found 593.4361.

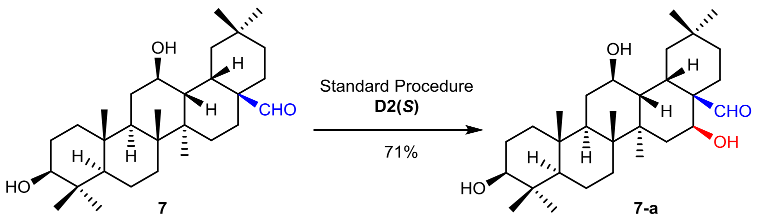

Compound 7-a | This compound was prepared from substrate **7** (26 mg, 0.06 mmol, 1.0 equiv.) following the standard procedure in the presence of **D2(*S*)** (34 mg, 0.28 mmol, 3.0 equiv.). The residue was purified by flash column chromatography (EtOAc/petroleum ether, 1:1) to afford product **7-a** (19 mg, 71%) as a white foam. R_f = 0.4 (silica, PE/EtOAc = 1:3); $[\alpha]_D^{28}$ = -74.8 (*c* 0.6, $CHCl_3$); IR (film) ν_{max} = 3404, 2945, 2865, 1706, 1030, 756 cm^{-1}; ^1H NMR (400 MHz, $CDCl_3$) δ 9.44 (d, *J* = 3.0 Hz, 1H), 4.03–3.97 (m, 1H), 3.83–3.74 (m, 1H), 3.19 (dd, *J* = 11.4, 4.7 Hz, 1H), 2.68–2.60 (m, 1H), 1.03 (s, 3H), 0.97 (s, 3H), 0.95 (s, 3H), 0.94 (s, 3H), 0.92 (s, 3H), 0.83 (s, 3H), 0.76 (s, 3H); ^{13}C NMR (101 MHz, $CDCl_3$) δ 209.35, 78.93, 67.96, 65.99, 55.32, 53.66, 48.92, 44.79, 43.37, 41.98, 38.99, 38.78, 37.79, 37.09, 35.75, 33.61, 33.32, 32.93, 32.70, 31.83, 30.42, 28.16, 27.39, 23.31, 21.93, 18.79, 18.35, 16.50, 16.04, 15.50; ESI-HRMS (*m/z*) calcd for $C_{30}H_{50}O_4Na$ $[M + Na]^+$ 497.3601, found 497.3603.

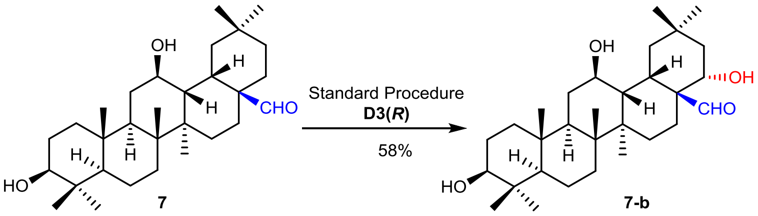

Compound 7-b | This compound was prepared from substrate **7** (26 mg, 0.06 mmol, 1.0 equiv.) following the standard procedure in the presence of **D3(*R*)** (26 mg, 0.17 mmol, 3.0 equiv.). The residue was purified by flash column chromatography (EtOAc/petroleum ether, 1:1) to afford product **7-b** (12 mg, 58%) as a white foam. R_f = 0.5 (silica, PE/EtOAc = 1:3); $[\alpha]_D^{28}$ = -43.9 (*c* 0.6, $CHCl_3$); IR (film) ν_{max} = 3445, 2945, 2920, 2870, 1715, 756 cm^{-1}; ^1H NMR (500 MHz, $CDCl_3$) δ 9.62 (s, 1H), 3.85 (dd, *J* = 12.0, 4.6 Hz, 1H), 3.81–3.75 (m, 1H), 3.19 (dd, *J* = 11.5, 4.6 Hz, 1H), 2.60–2.48 (m, 1H), 2.00–1.95 (m, 1H), 1.89–1.82 (m, 1H), 0.99 (s, 3H), 0.99 (s, 3H), 0.97 (s, 3H), 0.97 (s, 3H), 0.88 (s, 3H), 0.82 (s, 3H), 0.76 (s, 3H); ^{13}C NMR (126 MHz, $CDCl_3$) δ 208.55, 78.98, 68.37, 67.83, 55.41, 55.33, 49.14, 43.99, 42.58, 41.96, 41.34, 39.00, 38.77, 37.11, 35.37, 33.64, 32.67, 32.00, 31.58, 31.41, 28.17, 27.43, 27.27, 24.48, 18.37, 17.95, 16.46, 16.02, 15.50, 15.20; ESI-HRMS (*m/z*) calcd for $C_{30}H_{54}NO_4$ $[M + NH_4]^+$ 492.4047, found 492.4047.

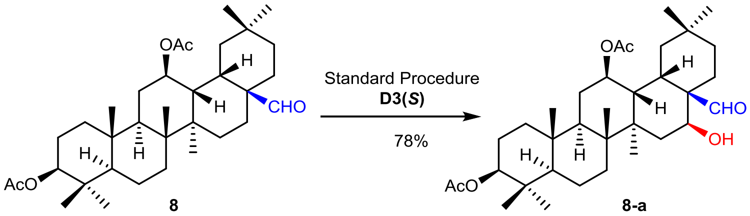

Compound 8-a | This compound was prepared from substrate **8** (25 mg, 0.05 mmol, 1.0 equiv.) following the standard procedure in the presence of **D3(*S*)** (21 mg, 0.14 mmol, 3.0 equiv.). The residue was purified by flash column chromatography (EtOAc/petroleum ether, 1:3) to afford product **8-a** (20 mg, 78%) as a white foam. R_f = 0.2 (silica, PE/EtOAc = 5:1); $[\alpha]_D^{28}$ = -35.6 (*c* 0.8, $CHCl_3$); IR (film) ν_{max} = 3446, 2948, 2859, 1731, 1246, 756 cm^{-1}; ^1H NMR (500 MHz, $CDCl_3$) δ 9.45 (d, *J* = 2.9 Hz, 1H), 5.12–4.87 (m, 1H), 4.46 (dd, *J* = 11.6, 4.5 Hz, 1H), 4.02–3.96 (m, 1H), 2.40–2.33 (m, 1H), 2.03 (s, 6H), 1.06 (s, 3H), 0.93 (s, 3H), 0.92 (s, 3H), 0.85 (s, 3H), 0.84 (s, 3H), 0.83 (s, 3H), 0.83 (s, 3H); ^{13}C NMR (126 MHz, $CDCl_3$) δ 208.82, 171.05, 170.57, 80.65, 71.37,

65.76, 55.35, 53.56, 48.39, 43.42, 41.91, 41.80, 38.39, 37.93, 37.69, 37.09, 35.70, 33.37, 33.15, 33.04, 32.48, 30.32, 28.12, 27.32, 23.66, 23.04, 21.93, 21.42, 21.26, 18.63, 18.19, 16.63, 16.42, 16.03; ESI-HRMS (*m/z*) calcd for $C_{34}H_{58}NO_6$ [M + NH_4]$^+$ 576.4259, found 576.4257.

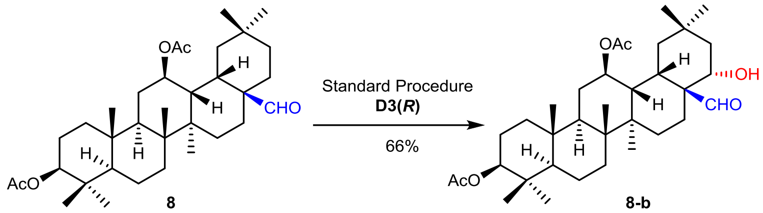

8 **8-b**

Compound 8-b | This compound was prepared from substrate **8** (25mg, 0.05 mmol, 1.0 equiv.) following the standard procedure in the presence of **D3(*R*)** (21 mg, 0.14 mmol, 3.0 equiv.). The residue was purified by flash column chromatography (EtOAc/petroleum ether, 1:3) to afford product **8-b** (17 mg, 66%) as a white foam. R_f = 0.5 (silica, PE/EtOAc = 3:1); $[\alpha]_D^{28}$ = -13.8 (*c* 1.1, $CHCl_3$); IR (film) ν_{max} = 3446, 2950, 2925, 1730, 1246, 755 cm^{-1}; ^{1}H NMR (400 MHz, $CDCl_3$) δ 9.61 (s, 1H), 5.03–4.95 (m, 1H), 4.47 (dd, *J* = 11.3, 4.5 Hz, 1H), 3.82 (dd, *J* = 12.0, 4.8 Hz, 1H), 2.31–2.23 (m, 1H), 2.03 (s, 3H), 2.03 (s, 3H), 1.02 (s, 3H), 0.95 (s, 3H), 0.89 (s, 3H), 0.87 (s, 3H), 0.84 (s, 3H), 0.82 (s, 6H); ^{13}C NMR (101 MHz, $CDCl_3$) δ 208.11, 171.10, 170.75, 80.69, 71.79, 67.88, 55.33, 48.64, 42.49, 41.87, 41.39, 40.90, 38.37, 37.92, 37.08, 35.34, 33.41, 32.40, 31.62, 31.28, 28.10, 27.38, 27.16, 24.13, 23.67, 21.43, 21.27, 18.18, 17.79, 16.63, 16.35, 16.00, 15.00; ESI-HRMS (*m/z*) calcd for $C_{34}H_{58}NO_6$ [M + NH_4]$^+$ 576.4259, found 576.4255.

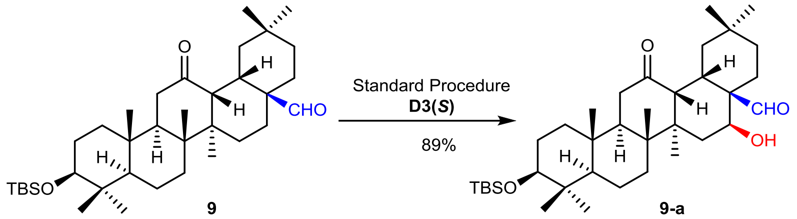

9 **9-a**

Compound 9-a | This compound was prepared from substrate **9** (25 mg, 0.04 mmol, 1.0 equiv.) following the standard procedure in the presence of **D3(*S*)** (20 mg, 0.13mmol, 3.0 equiv.). The residue was purified by flash column chromatography (EtOAc/petroleum ether, 1:5) to afford product **9-a** (23 mg, 89%) as a white foam. R_f = 0.3 (silica, PE/EtOAc = 5:1); $[\alpha]_D^{25}$ = -49.9 (*c* 1.0, $CHCl_3$); IR (film) ν_{max} = 3540, 2926, 2855, 1705, 1097, 862 cm^{-1}; ^{1}H NMR (400 MHz, $CDCl_3$) δ 9.40 (d, *J* = 2.9 Hz, 1H), 4.05 (ddd, *J* = 11.4, 4.9, 3.0 Hz, 1H), 3.15 (dd, *J* = 11.2, 4.5 Hz, 1H), 2.72–2.64 (m, 1H), 2.35 (d, *J* = 4.5 Hz, 1H), 2.30–2.22 (m, 1H), 2.18–2.06 (m, 1H), 1.98–1.91 (m, 2H), 1.92–1.82 (m, 1H), 1.00 (s, 9H), 0.93 (s, 3H), 0.90 (s, 3H), 0.88 (s, 9H), 0.85 (s, 3H), 0.75 (s, 3H), 0.03 (s, 6H); ^{13}C NMR (126 MHz, $CDCl_3$) δ 210.89, 208.61, 79.20, 65.59, 55.26, 53.65, 52.73, 49.48, 44.57, 41.77, 39.55, 38.50, 38.09, 36.96, 36.60, 35.81, 33.52, 33.29, 32.98, 32.02, 30.55, 28.53, 27.58, 26.04, 23.13, 22.05, 21.43, 18.58, 18.25, 16.25, 16.00, 15.38, -3.60, -4.77; ESI-HRMS (*m/z*) calcd for $C_{36}H_{62}O_4SiNa$ [M + Na]$^+$ 609.4310, found 609.4309.

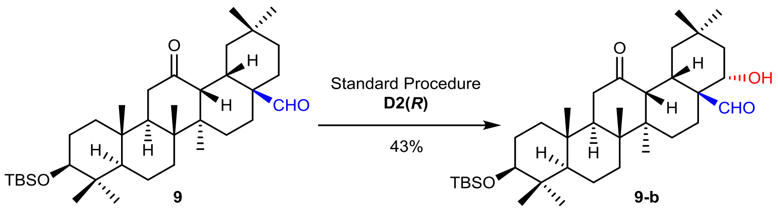

Compound 9-b | This compound was prepared from substrate **9** (25 mg, 0.04 mmol, 1.0 equiv.) following the standard procedure in the presence of **D2(*R*)** (27 mg, 0.22 mmol, 3.0 equiv.). The residue was purified by flash column chromatography (EtOAc/petroleum ether, 1:5) to afford product **9-b** (11 mg, 43%) as a white foam. R_f = 0.3 (silica, PE/EtOAc = 5:1); $[\alpha]_D^{28}$ = -25.9 (*c* 0.5, $CHCl_3$); IR (film) ν_{max} = 3430, 2926, 2854, 1699, 1259, 835 cm^{-1}; ^{1}H NMR (500 MHz, $CDCl_3$) δ 9.57 (d, *J* = 1.4 Hz, 1H), 3.90 (dd, *J* = 12.0, 4.8 Hz, 1H), 3.16 (dd, *J* = 11.3, 4.3 Hz, 1H), 2.59–2.53 (m, 1H), 2.34 (d, *J* = 4.3 Hz, 1H), 2.29–2.23 (m, 1H), 2.17–2.08 (m, 1H), 2.04–1.98 (m, 1H), 1.97–1.91 (m, 1H), 1.79–1.71 (m, 1H), 1.02 (s, 3H), 0.96 (s, 6H), 0.95 (s, 3H), 0.90 (s, 3H), 0.88 (s, 9H), 0.84 (s, 3H), 0.74 (s, 3H), 0.03 (s, 6H); ^{13}C NMR (126 MHz, $CDCl_3$) δ 211.56, 207.78, 79.24, 68.07, 55.50, 55.29, 52.18, 49.77, 42.93, 42.79, 41.72, 39.55, 38.58, 38.09, 36.97, 35.40, 33.52, 31.99, 31.59, 31.50, 28.53, 27.60, 26.11, 26.05, 24.33, 20.74, 18.59, 18.26, 16.20, 16.01, 15.38, 14.93, -3.60, -4.77; ESI-HRMS (*m/z*) calcd for $C_{36}H_{62}O_4SiNa$ $[M + Na]^+$ 609.4310, found 609.4310.

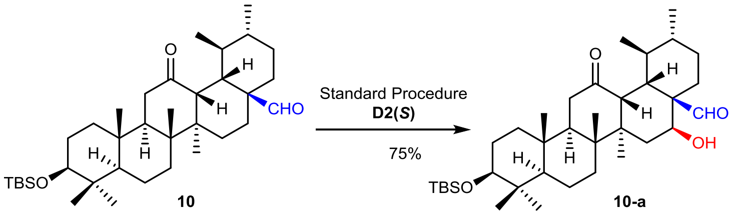

Compound 10-a | This compound was prepared from substrate **10** (30 mg, 0.05 mmol, 1.0 equiv.) following the standard procedure in the presence of **D2(*S*)** (20 mg, 0.16 mmol, 3.0 equiv.). The residue was purified by flash column chromatography (EtOAc/petroleum ether, 1:5) to afford product **10-a** (23 mg, 75%) as a white foam. R_f = 0.4 (silica, PE/EtOAc = 5:1); $[\alpha]_D^{28}$ = 59.3 (*c* 0.7, $CHCl_3$); IR (film) ν_{max} = 3533, 2932, 2856, 1704, 1102, 758 cm^{-1}; ^{1}H NMR (400 MHz, $CDCl_3$) δ 9.54 (d, *J* = 3.2 Hz, 1H), 3.89–3.83 (m, 1H), 3.21 (dd, *J* = 11.4, 4.3 Hz, 1H), 2.51–2.43 (m, 2H), 2.33–2.23 (m, 1H), 2.20–2.01 (m, 3H), 1.85–1.78 (m, 1H), 1.31 (s, 3H), 0.96 (d, *J* = 5.9 Hz, 3H), 0.96 (s, 3H), 0.93 (s, 3H), 0.88 (s, 9H), 0.87 (d, *J* = 6.5 Hz, 3H), 0.77 (s, 3H), 0.76 (s, 3H), 0.03 (d, *J* = 1.8 Hz, 6H); ^{13}C NMR (101 MHz, $CDCl_3$) δ 215.88, 208.82, 79.33, 66.65, 55.59, 53.45, 53.38, 48.17, 46.57, 42.32, 40.94, 39.76, 39.43, 39.18, 38.82, 38.77, 37.46, 36.74, 35.16, 28.87, 28.70, 27.70, 27.64, 26.02, 25.92, 21.32, 20.00, 18.56, 18.24, 18.13, 16.52, 16.30, -3.64, -4.77; ESI-HRMS (*m/z*) calcd for $C_{36}H_{62}O_4SiNa$ $[M + Na]^+$ 609.4310, found 609.4312.

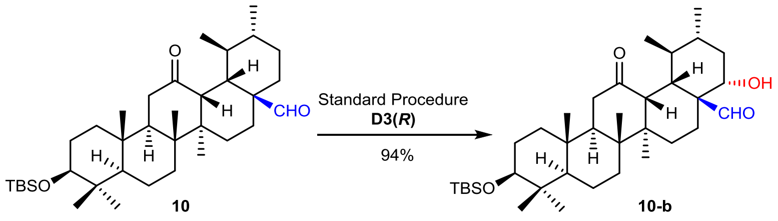

Compound 10-b | This compound was prepared from substrate **10** (30 mg, 0.05 mmol, 1.0 equiv.) following the standard procedure in the presence of **D3(*R*)** (40 mg, 0.26 mmol, 3.0 equiv.); during work up the mixture was additionally treated with 6N aq. HCl (0.1 mL) for 1 h. The residue was purified by flash column chromatography (EtOAc/petroleum ether, 1:5) to afford product **10-b** (29 mg, 94%) as a white foam. R_f = 0.4 (silica, PE/EtOAc = 5:1); $[\alpha]_D^{28}$ = 43.4 (*c* 0.3, $CHCl_3$); IR (film) ν_{max} = 3447, 2927, 2854, 1705, 1069, 835 cm^{-1}; ^1H NMR (400 MHz, $CDCl_3$) δ 9.68 (d, *J* = 1.7 Hz, 1H), 3.73 (dd, *J* = 11.3, 4.3 Hz, 1H), 3.22 (dd, *J* = 11.4, 4.4 Hz, 1H), 2.50–2.40 (m, 2H), 2.33–2.24 (m, 1H), 2.20–2.12 (m, 1H), 1.99–1.92 (m, 1H), 1.28 (s, 3H), 1.00 (d, *J* = 6.3 Hz, 3H), 0.96 (s, 3H), 0.93 (s, 3H), 0.89 (s, 9H), 0.88 (d, *J* = 5.4 Hz, 3H), 0.76 (s, 3H), 0.72 (s, 3H), 0.04 (d, *J* = 1.2 Hz, 6H); ^{13}C NMR (101 MHz, $CDCl_3$) δ 216.01, 208.11, 79.39, 68.88, 55.62, 54.76, 54.15, 48.22, 44.45, 42.34, 39.92, 39.44, 39.22, 38.87, 38.75, 37.48, 37.17, 36.71, 35.12, 28.89, 27.66, 27.32, 26.74, 26.04, 21.07, 19.85, 18.58, 18.26, 17.90, 16.54, 16.48, 16.29, -3.63, -4.76; ESI-HRMS (*m/z*) calcd for $C_{36}H_{62}O_4SiNa$ $[M + Na]^+$ 609.4310, found 609.4314.

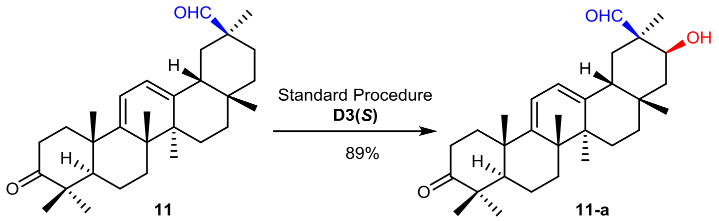

Compound 11-a | This compound was prepared from substrate **11** (25 mg, 0.06 mmol, 1.0 equiv.) following the standard procedure in the presence of **D3(*S*)** (43 mg, 0.29 mmol, 3.0 equiv.). The residue was purified by flash column chromatography (EtOAc/petroleum ether, 1:3) to afford product **11-a** (23 mg, 89%) as a white solid. R_f = 0.3 (silica, PE/EtOAc = 3:1); $[\alpha]_D^{28}$ = 326.9 (*c* 0.7, $CHCl_3$); mp 243.8–246.5 ℃; IR (film) ν_{max} = 3481, 2968, 2925, 1705, 1456, 756 cm^{-1}; ^1H NMR (400 MHz, $CDCl_3$) δ 9.66 (d, *J* = 2.2 Hz, 1H), 5.65 (d, *J* = 5.8 Hz, 1H), 5.60 (d, *J* = 5.8 Hz, 1H), 3.66–3.59 (m, 1H), 2.64–2.54 (m, 1H), 2.53–2.45 (m, 1H), 2.25–2.17 (m, 1H), 2.05–1.98 (m, 1H), 1.26 (s, 3H), 1.19 (s, 3H), 1.14 (s, 3H), 1.11 (s, 3H), 1.07 (s, 3H), 1.00 (s, 3H), 0.90 (s, 3H); ^{13}C NMR (101 MHz, $CDCl_3$) δ 217.70, 208.44, 153.50, 144.76, 122.14, 117.41, 72.83, 51.86, 51.72, 47.48, 46.71, 46.61, 43.08, 42.05, 40.60, 38.43, 37.91, 34.84, 34.62, 31.34, 28.38, 28.02, 26.99, 25.71, 25.38, 21.44, 20.84, 20.75, 20.26, 19.65; ESI-HRMS (*m/z*) calcd for $C_{30}H_{44}O_3Na$ $[M + Na]^+$ 475.3183, found 475.3188.

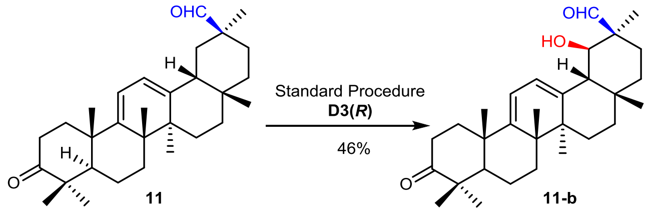

Compound 11-b | This compound was prepared from substrate **11** (25 mg, 0.06 mmol, 1.0 equiv.) following the standard procedure in the presence of **D3(*R*)** (43 mg, 0.29 mmol, 3.0 equiv.). The residue was purified by flash column chromatography (EtOAc/petroleum ether, 1:3) to afford product **11-b** (12 mg, 46%) as a white solid. R_f = 0.6 (silica, PE/EtOAc = 2:1); $[\alpha]_D^{28}$ = 295.7 (*c* 0.3, $CHCl_3$); mp 204.1–208.8 ℃; IR (film) ν_{max} = 3544, 2967, 2932, 2865, 1706, 757 cm^{-1}; ^1H

NMR (400 MHz, $CDCl_3$) δ 9.90 (s, 1H), 5.72 (d, J = 5.7 Hz, 1H), 5.69 (d, J = 5.7 Hz, 1H), 3.62 (d, J = 11.0 Hz, 1H), 2.65–2.47 (m, 2H), 2.26–2.18 (m, 1H), 1.29 (s, 3H), 1.23 (s, 3H), 1.21 (s, 3H), 1.12 (s, 3H), 1.08 (s, 3H), 1.02 (s, 3H), 0.90 (s, 3H); ^{13}C NMR (101 MHz, $CDCl_3$) δ 217.52, 207.64, 155.13, 140.88, 123.80, 116.93, 74.04, 54.65, 51.76, 50.56, 47.46, 43.42, 40.43, 38.52, 37.90, 36.81, 34.64, 34.57, 31.26, 29.11, 28.51, 28.18, 27.08, 25.74, 25.49, 22.65, 21.44, 21.42, 19.61, 19.25; ESI-HRMS (*m/z*) calcd for $C_{30}H_{44}O_3Na$ $[M + Na]^+$ 475.3183, found 475.3187.

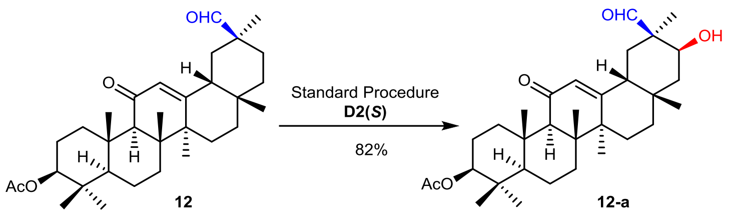

Compound 12-a | This compound was prepared from substrate **12** (26 mg, 0.05 mmol, 1.0 equiv.) following the standard procedure in the presence of **D2(*S*)** (20 mg, 0.16 mmol, 3.0 equiv.). The residue was purified by flash column chromatography (EtOAc/petroleum ether, 1:4) to afford product **12-a** (22 mg, 82%) as a white solid. R_f = 0.2 (silica, PE/EtOAc = 5:1); $[\alpha]_D^{28}$ = 51.8 (*c* 0.45, $CHCl_3$); mp 244.6–246.5 °C; IR (film) ν_{max} = 3480, 2950, 2923, 1726, 1658, 1248 cm^{-1}; ^1H NMR (400 MHz, $CDCl_3$) δ 9.62 (d, J = 2.2 Hz, 1H), 5.63 (s, 1H), 4.51 (dd, J = 11.6, 4.8 Hz, 1H), 3.68–3.62 (m, 1H), 2.81–2.74 (m, 1H), 2.34 (s, 1H), 2.05 (s, 3H), 1.36 (s, 3H), 1.22 (s, 3H), 1.15 (s, 3H), 1.11 (s, 3H), 0.87 (s, 6H), 0.86 (s, 3H); ^{13}C NMR (101 MHz, $CDCl_3$) δ 207.63, 199.86, 171.19, 167.24, 129.14, 80.69, 72.64, 61.91, 55.13, 51.54, 48.24, 46.23, 45.53, 43.35, 40.23, 38.93, 38.18, 37.08, 35.08, 32.78, 28.42, 28.17, 27.27, 26.49, 23.66, 21.46, 20.81, 18.82, 17.48, 16.81, 16.53; ESI-HRMS (*m/z*) calcd for $C_{32}H_{49}O_5$ $[M + H]^+$ 513.3575, found 513.3572.

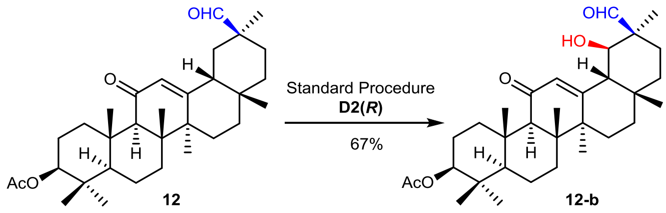

Compound 12-b | This compound was prepared from substrate **12** (26 mg, 0.05 mmol, 1.0 equiv.) following the standard procedure in the presence of **D2(*R*)** (20 mg, 0.16 mmol, 3.0 equiv.). The residue was purified by flash column chromatography (EtOAc/petroleum ether, 1:4) to afford product **12-b** (18 mg, 67%) as a white solid. R_f = 0.2 (silica, PE/EtOAc = 6:1); $[\alpha]_D^{28}$ = 231.3 (*c* 0.25, $CHCl_3$); mp 250.9–254.7 ℃; IR (film) ν_{max} = 3501, 2970, 2930, 1721, 1660, 1247 cm^{-1}; ^1H NMR (400 MHz, $CDCl_3$) δ 9.68 (d, J = 1.0 Hz, 1H), 5.67 (s, 1H), 4.52 (dd, J = 11.6, 4.8 Hz, 1H), 3.71–3.67 (m, 1H), 2.78–2.71 (m, 1H), 2.38 (s, 1H), 2.12–2.07 (m, 1H), 2.05 (s, 3H), 1.38 (s, 3H), 1.26 (s, 3H), 1.17 (s, 3H), 1.16 (s, 3H), 0.88 (s, 6H), 0.85 (s, 3H); ^{13}C NMR (101 MHz, $CDCl_3$) δ 207.63, 199.23, 171.14, 162.42, 131.18, 80.66, 74.75, 61.82, 55.96, 55.05, 51.04, 45.49, 43.42, 38.92, 38.19, 37.01, 36.90, 35.14, 32.85, 29.41, 28.58, 28.19, 27.46, 26.58, 23.69, 22.12, 22.06, 21.46, 18.78, 17.53, 16.87, 16.57; ESI-HRMS (*m/z*) calcd for $C_{32}H_{49}O_5$ $[M + H]^+$ 513.3575, found 513.3579.

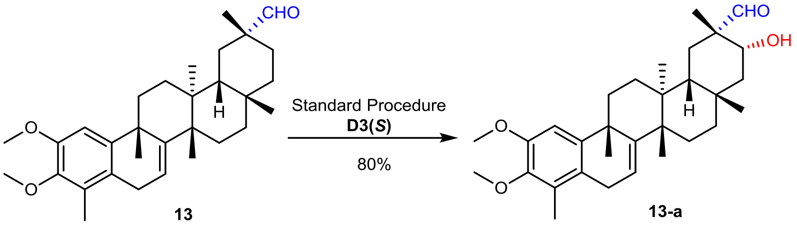

Compound 13-a | This compound was prepared from substrate **13** (35 mg, 0.08 mmol, 1.0 equiv.) following the standard procedure in the presence of **D3(*S*)** (34 mg, 0.23 mmol, 3.0 equiv.). The residue purified by flash column chromatography (EtOAc/petroleum ether, 1:5) to afford product **13-a** (29 mg, 80%) as an orange oil. R_f = 0.3 (silica, PE/EtOAc = 5:1); $[\alpha]_D^{25}$ = -5.3 (*c* 1.0, $CHCl_3$); IR (film) ν_{max} = 3486, 2925, 2869, 1713, 1262, 737 cm^{-1}; ^1H NMR (400 MHz, $CDCl_3$) δ 9.38 (d, *J* = 2.7 Hz, 1H), 6.74 (s, 1H), 5.79 (dd, *J* = 6.1, 1.8 Hz, 1H), 3.84 (s, 3H), 3.76 (s, 3H), 3.33–3.24 (m, 1H), 3.05–2.97 (m, 1H), 2.17 (s, 3H), 1.34 (s, 3H), 1.24 (s, 3H), 1.22 (s, 3H), 1.14 (s, 3H); ^{13}C NMR (101 MHz, $CDCl_3$) δ 207.92, 151.06, 148.64, 145.04, 144.50, 128.03, 125.68, 118.46, 106.29, 73.71, 60.48, 56.03, 49.60, 44.57, 44.23, 43.75, 37.71, 37.25, 37.03, 34.49, 34.28, 33.61, 32.53, 31.71, 30.97, 28.82, 27.99, 23.98, 22.81, 20.96, 11.96; ESI-HRMS (*m/z*) calcd for $C_{31}H_{44}NaO_4$ $[M + Na]^+$ 503.3132, found 503.3124.

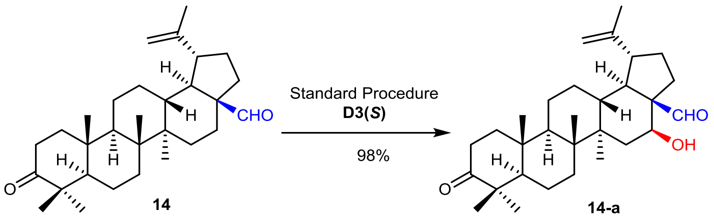

Compound 14-a | This compound was prepared from substrate **14** (25 mg, 0.06 mmol, 1.0 equiv.) following the standard procedure in the presence of **D3(*S*)** (43 mg, 0.29 mmol, 3.0 equiv.); during work up the mixture was additionally treated with 6N aq. HCl (0.1 mL) for 1h. The residue was purified by flash column chromatography (EtOAc/petroleum ether, 1:5) to afford product **14-a** (25 mg, 98%) as a white foam. R_f = 0.3 (silica, PE/EtOAc = 5:1); $[\alpha]_D^{28}$ = 11.5 (*c* 0.45, $CHCl_3$); IR (film) ν_{max} = 3446, 2929, 2868, 1704, 1696, 752 cm^{-1}; ^1H NMR (400 MHz, $CDCl_3$) δ 10.00 (d, *J* = 1.5 Hz, 1H), 4.72 (d, *J* = 39.7 Hz, 2H), 3.70 (ddd, *J* = 11.5, 4.8, 1.7 Hz, 1H), 2.89–2.76 (m, 1H), 2.54–2.35 (m, 2H), 1.71 (s, 3H), 1.07 (s, 3H), 1.02 (s, 6H), 0.98 (s, 3H), 0.92 (s, 3H); ^{13}C NMR (101 MHz, $CDCl_3$) δ 218.08, 209.11, 148.54, 111.00, 76.05, 63.69, 55.04, 49.37, 48.61, 48.13, 47.48, 44.81, 40.99, 39.74, 39.42, 38.98, 36.97, 34.23, 33.69, 32.46, 30.08, 26.75, 25.27, 21.26, 21.17, 19.71, 19.08, 16.08, 15.88, 15.56; ESI-HRMS (*m/z*) calcd for $C_{30}H_{50}NO_3$ $[M + NH_4]^+$ 472.3785, found 472.3786.

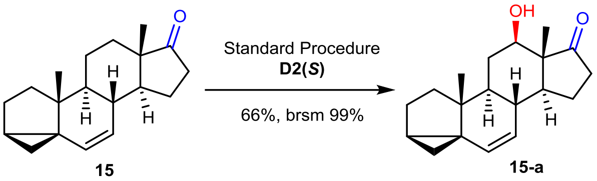

Compound 15-a | This compound was prepared from substrate **15** (23 mg, 0.09 mmol, 1.0 equiv.) following the standard procedure in the presence of **D2(*S*)** (52 mg, 0.43 mmol, 3.0 equiv.). The

residue was purified by flash column chromatography (EtOAc/petroleum ether, 1:10) to afford product **15-a** (16 mg, 66%) and recover substrate **15** (8.0 mg, brsm 99%) as a white powder. R_f = 0.4 (silica, PE/EtOAc = 5:1); $[\alpha]_D^{25}$ = -5.6 (*c* 1.0, $CHCl_3$); IR (film) ν_{max} = 3555, 2963, 1718, 1455, 1056, 812 cm^{-1}; ^1H NMR (400 MHz, $CDCl_3$) δ 5.54 (d, *J* = 9.8 Hz, 1H), 5.28 (dd, *J* = 9.7, 2.4 Hz, 1H), 3.88–3.77 (m, 1H), 2.96 (s, 1H), 2.56–2.43 (m, 1H), 2.28–2.20 (m, 1H), 1.00 (s, 3H), 0.93 (s, 3H), 0.84 (t, *J* = 4.8 Hz, 1H), 0.50 (dd, *J* = 8.0, 5.3 Hz, 1H); ^{13}C NMR (126 MHz, $CDCl_3$) δ 222.56, 133.50, 123.99, 73.06, 52.52, 48.08, 44.43, 42.81, 36.83, 35.98, 35.06, 31.64, 29.25, 25.95, 25.12, 21.85, 17.95, 14.89, 8.43; ESI-HRMS (*m/z*) calcd for $C_{19}H_{26}O_2Na$ $[M + Na]^+$ 309.1825, found 309.1829.

Synthesis of Saikosaponin E (1)

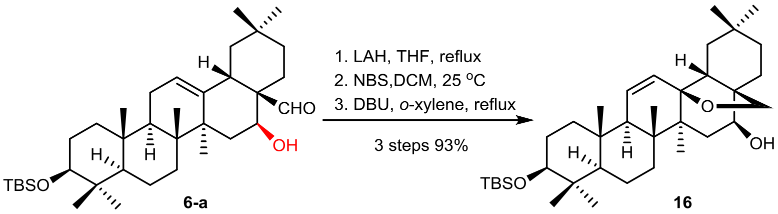

Compound 16 | To a stirred solution of **6-a** (470 mg, 0.82 mmol, 1.0 equiv.) in THF (12 mL), was added $LiAlH_4$ (130 mg, 3.42 mmol, 4.0 equiv.) at 0 ℃. The mixture was stirred at 25 °C for 2 hours, the excess reagents were quenched with water and 15% NaOH (10 mL and 10 mL), and the resulting mixture was extracted with EtOAc (3 × 10 mL). The combined organic phases were washed with brine (20 mL), dried over anhydrous Na_2SO_4, filtered, and concentrated under vacuum. The residue was dissolved in CH_2Cl_2 (10 mL), to which was added NBS (600 mg, 3.60 mmol, 4.4 equiv.). The mixture was stirred at 25 °C for 6 hours, the excess reagents were quenched with aq. Na_2SO_3 (10 mL), and the resulting mixture was extracted with CH_2Cl_2 (3 × 10 mL). The combined organic phases were washed with brine (20 mL), dried over anhydrous Na_2SO_4, filtered, and concentrated under vacuum. The residue was dissolved in *o*-xylene (7.0 mL), to which was added DBU (5.0 mL, 33.5 mmol, 41.0 equiv.). The mixture was stirred at 150 ℃ for 7 hours, the excess reagents were quenched with aq. $NaHCO_3$ (10 mL), and the resulting mixture was extracted with CH_2Cl_2 (3 × 10 mL). The combined organic phases were washed with brine (20 mL), dried over anhydrous Na_2SO_4, filtered, and concentrated under vacuum. The residue was purified by flash column chromatography (EtOAc/petroleum ether, 1:8) to give **16** (437 mg, 93%) as a white powder. R_f = 0.2 (silica, PE/EtOAc = 10:1); $[\alpha]_D^{25}$ = 39.4 (*c* 1.0, $CHCl_3$); IR (film) ν_{max} = 3413, 2924, 2853, 1462, 1258, 800 cm^{-1}; ^1H NMR (500 MHz, $CDCl_3$) δ 5.88 (d, *J* = 10.4 Hz, 1H), 5.41 (dd, *J* = 10.3, 3.0 Hz, 1H), 4.21 (dd, *J* = 9.8, 5.9 Hz, 1H), 3.90 (d, *J* = 7.5 Hz, 1H), 3.17 (dd, *J* = 11.4, 4.6 Hz, 1H), 3.12 (d, *J* = 7.5 Hz, 1H), 2.04–1.98 (m, 1H), 1.08 (s, 3H), 0.99 (s, 3H), 0.98 (s, 3H), 0.91 (s, 3H), 0.89 (s, 15H), 0.74 (s, 3H), 0.03 (d, *J* = 2.5 Hz, 6H); ^{13}C NMR (126 MHz, $CDCl_3$) δ 132.96, 129.71, 83.89, 79.53, 72.36, 65.35, 55.06, 52.74, 51.67, 46.39, 45.69, 41.92, 39.66, 38.41, 37.52, 36.38, 35.58, 34.40, 33.73, 31.65, 31.64, 28.35, 27.65, 26.08, 25.15, 23.85, 20.84, 19.63, 18.28, 18.06, 18.03, 15.58, -3.57, -4.75; ESI-HRMS (*m/z*) calcd for $C_{36}H_{62}O_3SiNa$ $[M + Na]^+$ 593.4360, found 593.4360.

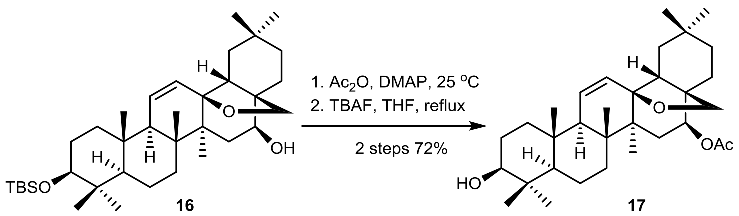

Compound 17 | To a stirred solution of **16** (177 mg, 0.31 mmol, 1.0 equiv.) in CH_2Cl_2 (3.0 mL), were added Ac_2O (63 mg, 0.62 mmol, 2.0 equiv.) and DMAP (114 mg, 0.93 mmol, 3.0 equiv.). The mixture was stirred at 25 °C for 4 hours, the excess reagents were quenched with $NaHCO_3$ (10 mL) and the resulting mixture was extracted with CH_2Cl_2 (3 × 3.0 mL). The combined organic phases were washed with brine (10 mL), dried over anhydrous Na_2SO_4, filtered, and concentrated under vacuum. The residue was dissolved in THF (3 mL), to which was added TBAF (1M in THF, 6.3 mL, 20.0 equiv.) at 25 °C. After stirring at 70 ℃ for 10h, the excess reagents were quenched with saturated aq. $NaHCO_3$ (10 mL), and the resulting mixture was extracted with EtOAc (3 × 3.0 mL). The combined organic phases were washed with brine (10 mL), dried over anhydrous Na_2SO_4, filtered, and concentrated under vacuum. The residue was purified by flash column chromatography (EtOAc/petroleum ether, 1:3) to give **17** (112 mg, 72%) as a white foam. R_f = 0.3 (silica, PE/EtOAc = 5:1); $[\alpha]_D^{25}$ = 100.1 (*c* 1.0, $CHCl_3$); IR (film) ν_{max} = 3445, 2927, 2865, 1737, 1259, 738 cm^{-1}; ^1H NMR (500 MHz, $CDCl_3$) δ 5.87 (d, *J* = 10.3 Hz, 1H), 5.38–5.43 (m, 2H), 3.97 (d, *J* = 7.4 Hz, 1H), 3.16–3.22 (m, 2H), 2.05 (s, 3H), 1.08 (s, 3H), 1.03 (s, 3H), 0.96 (s, 6H), 0.88 (s, 6H), 0.76 (s, 3H), 0.69 (dd, *J* = 11.5, 2.0 Hz, 1H); ^{13}C NMR (126 MHz, $CDCl_3$) δ 170.79, 132.86, 129.66, 83.93, 78.93, 73.23, 68.84, 54.85, 52.56, 51.70, 45.35, 45.28, 41.89, 39.04, 38.35, 37.44, 36.42, 34.31, 33.46, 32.01, 31.52, 31.50, 27.89, 27.16, 25.46, 23.78, 21.35, 20.59, 19.58, 17.96, 17.73, 15.06; ESI-HRMS (*m/z*) calcd for $C_{32}H_{50}O_4Na$ $[M + Na]^+$ 521.3601, found 521.3604.

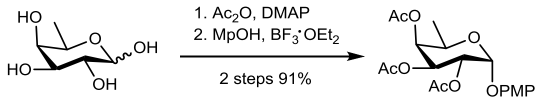

Compound S22 | To a stirred solution of D-fucose (500 mg, 3.05 mmol, 1.0 equiv.) in pyridine (10 mL), were added Ac_2O (2.42 mL, 25.6 mmol, 8.4 equiv.) and DMAP (53 mg, 0.43 mmol, 0.1 equiv.). The mixture was stirred at 25 °C for 2 hours, the excess reagents were quenched with $NaHCO_3$ (10 mL), and the resulting mixture was extracted with EtOAc (3 × 10 mL). The combined organic phases were washed with brine (20 mL), dried over anhydrous Na_2SO_4, filtered, and concentrated under vacuum. The residue was dissolved in CH_2Cl_2 (6 mL), to which were added 4-methoxyphenol (757 mg, 6.09 mmol, 2.0 equiv.) and boron trifluoride etherate (1.15 mL, 9.14 mmol, 3.0 equiv.) at 0 ℃. After stirring at 25 °C for 3 h, the excess reagents were quenched with saturated aq. $NaHCO_3$ (20 mL), and the resulting mixture was extracted with EtOAc (3 × 10 mL). The combined organic phases were washed with brine (10 mL), dried over anhydrous Na_2SO_4, filtered, and concentrated under vacuum. The residue was purified by flash column chromatography (EtOAc/petroleum ether, 1:5) to give **S22** (1.1 g, 91%) as a white foam. R_f = 0.2 (silica, PE/EtOAc = 5:1); $[\alpha]_D^{25}$ = 169.3 (*c* 1.0, $CHCl_3$); IR (film) ν_{max} = 2924, 2852, 1746, 1507, 1069, 830 cm^{-1}; ^1H NMR (500 MHz, $CDCl_3$) δ 7.00–6.94 (m, 1H), 6.85–6.79 (m, 1H), 5.62 (d, *J* =

3.6 Hz, 1H), 5.56 (dd, J = 10.9, 3.4 Hz, 1H), 5.38–5.34 (m, 1H), 5.25 (dd, J = 10.9, 3.7 Hz, 1H), 4.34–4.27 (m, 1H), 3.77 (s, 3H), 2.18 (s, 3H), 2.07 (s, 3H), 2.02 (s, 3H), 1.13 (d, J = 6.5 Hz, 3H); ^{13}C NMR (126 MHz, $CDCl_3$) δ 170.70, 170.59, 170.23, 155.34, 150.77, 117.90, 114.81, 95.81, 71.18, 68.11, 68.08, 65.34, 55.79, 20.91, 20.86, 20.78, 16.01; ESI-HRMS (*m/z*) calcd for $C_{19}H_{24}O_9Na$ [M + Na]$^+$ 419.1313, found 419.1318.

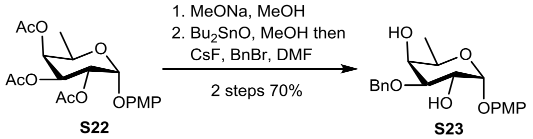

Compound S23 | To a stirred solution **S22** (1.1 g, 2.78 mmol, 1.0 equiv.) in CH_2Cl_2/MeOH (2.0 mL/7.0 mL), was added MeONa (150 mg, 2.78 mmol, 1.0 equiv.). The mixture was stirred at 25 °C for 8 hours, the excess reagents were quenched with acid resin until pH to 7. The mixture was filtered through a pad of celite and washed with MeOH for three times. The filtrate was concentrated under vacuum. The residue was dissolved in MeOH (10 mL), to which was added dibutyltin oxide (829 mg, 3.33 mmol, 1.2 equiv.) at 25 °C. After stirring at 50 ℃ for 4 h, the mixture was concentrated under vacuum. The residue was dissolved in DMF (10 mL), to which were added cesium fluoride (633 mg, 4.16 mmol, 1.5 equiv.) and benzyl bromide (0.5 mL, 4.16 mmol, 1.5 equiv.) at 25 °C. After stirring at 60 ℃ for 8 h, the excess reagents were quenched with saturated aq. $NaHCO_3$ (20 mL), and the resulting mixture was extracted with EtOAc (3 × 10 mL). The combined organic phases were washed with brine (10 mL), dried over anhydrous Na_2SO_4, filtered, and concentrated under vacuum. The residue was purified by flash column chromatography (EtOAc/petroleum ether, 1:2) to give **S23** (700 mg, 70%) as a white foam. R_f = 0.6 (silica, PE/EtOAc = 1:1); $[\alpha]_D^{25}$ = 194.5 (*c* 0.4, $CHCl_3$); IR (film) ν_{max} = 3519, 2923, 2853, 1507, 1034, 801 cm^{-1}; ^{1}H NMR (400 MHz, $CDCl_3$) δ 7.52–7.30 (m, 1H), 7.07–6.96 (m, 1H), 6.87–6.79 (m, 1H), 5.45 (d, J = 3.9 Hz, 1H), 4.87–4.70 (m, 1H), 4.18–4.00 (m, 2H), 3.91 (d, J = 2.4 Hz, 1H), 3.83 (dd, J = 9.7, 3.2 Hz, 1H), 3.77 (s, 3H), 1.29 (d, J = 6.6 Hz, 3H); ^{13}C NMR (126 MHz, $CDCl_3$) δ 155.33, 150.89, 137.97, 128.80, 128.26, 128.01, 118.26, 114.80, 98.70, 78.91, 72.36, 69.57, 68.44, 66.58, 55.80, 16.34; ESI-HRMS (*m/z*) calcd for $C_{20}H_{24}O_6Na$ [M + Na]$^+$ 383.1465, found 383.1469.

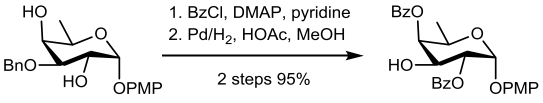

Compound S24 | To a stirred solution of **S23** (823 mg, 2.28 mmol, 1.0 equiv.) in CH_2Cl_2 (10 mL), were added pyridine (0.8 mL, 9.13 mmol, 4.0 equiv.), DMAP (112 mg, 0.91 mmol, 0.4 equiv.), and benzoyl chloride (1.1 mL, 9.13 mmol, 4.0 equiv.). The mixture was stirred at 25 °C for 5 hours, the excess reagents were quenched with $NaHCO_3$ (10 mL), and the resulting mixture was extracted with EtOAc (3 × 10 mL). The combined organic phases were washed with brine (20 mL), dried over anhydrous Na_2SO_4, filtered, and concentrated under vacuum. The residue was dissolved in MeOH (15 mL), to which were added acetic acid (0.2 mL) and 10% palladium on activated carbon (250 mg) at 25 °C. After stirring under H_2 (1 atm) atmosphere for 8 h, the mixture was filtered through a pad of celite and washed with EtOAc (3 × 10 mL). The filtrate was concentrated

under vacuum. The residue was purified by flash column chromatography (EtOAc/petroleum ether, 1:3) to give **S24** (1.04 g, 95%) as a white foam. R_f = 0.6 (silica, PE/EtOAc = 2:1); $[\alpha]_D^{25}$ = 161.2 (*c* 0.5, $CHCl_3$); IR (film) ν_{max} = 3485, 2960, 2853, 1720, 1265, 711 cm^{-1}; ^1H NMR (400 MHz, $CDCl_3$) δ 8.19–8.15 (m, 2H), 8.11–8.06 (m, 2H), 7.66–7.55 (m, 2H), 7.47 (dt, *J* = 21.4, 7.7 Hz, 4H), 7.06–6.99 (m, 2H), 6.87–6.77 (m, 2H), 5.74 (d, *J* = 3.7 Hz, 1H), 5.64 (d, *J* = 2.6 Hz, 1H), 5.49 (dd, *J* = 10.4, 3.7 Hz, 1H), 4.70 (dd, *J* = 10.4, 3.5 Hz, 1H), 4.45 (q, *J* = 6.3 Hz, 1H), 3.76 (s, 3H), 1.25 (d, *J* = 6.5 Hz, 3H); ^{13}C NMR (101 MHz, $CDCl_3$) δ 166.96, 166.87, 155.41, 151.14, 133.64, 133.60, 130.14, 130.01, 129.55, 129.48, 128.74, 128.62, 118.30, 114.83, 96.80, 74.31, 71.99, 67.71, 66.15, 55.80, 16.46; ESI-HRMS (*m/z*) calcd for $C_{27}H_{26}O_8Na$ [M + Na]$^+$ 501.1520, found 501.1523.

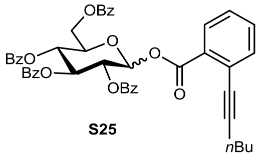

S25

Compound S25 | This compound was prepared by a literature procedure.[22]

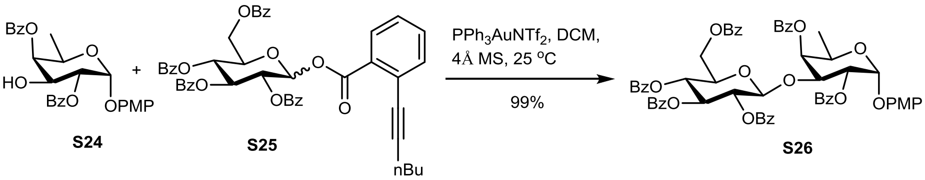

Disaccharide S26 | To a stirred mixture of **S24** (700 mg, 1.46 mmol, 1.0 equiv.), **S25** (2.24 g, 2.93 mmol, 2.0 equiv.), and 5Å molecular sieves (2.0 g) in CH_2Cl_2 (20 mL), was added PPh_3AuNTf_2 (108 mg, 0.146 mmol, 0.1 equiv.). The mixture was stirred at 25 °C for 3 hours, the excess reagents were quenched with Et_3N (5.0 mL). The mixture was filtered through a pad of celite and washed with EtOAc (3 × 10 mL). The filtrate was concentrated under vacuum. The residue was purified by flash column chromatography (EtOAc/petroleum ether/toluene, 1:3:6) to give **S26** (1.54 g, 99%) as a white foam. R_f = 0.3 (silica, PE/EtOAc = 10:1); $[\alpha]_D^{25}$ = 92.1 (*c* 1.0, $CHCl_3$); IR (film) ν_{max} = 2960, 1732, 1451, 1265, 1096, 708 cm^{-1}; ^1H NMR (500 MHz, $CDCl_3$) δ 8.12–8.06 (m, 2H), 8.06–8.01 (m, 2H), 7.91–7.84 (m, 4H), 7.72–7.69 (m, 2H), 7.56–7.46 (m, 4H), 7.44–7.39 (m, 4H), 7.38–7.28 (m, 8H), 7.22–7.18 (m, 2H), 7.02–6.97 (m, 2H), 6.91–6.86 (m, 2H), 6.79–6.74 (m, 2H), 5.85–5.76 (m, 2H), 5.68 (d, *J* = 3.7 Hz, 1H), 5.60 (t, *J* = 9.7 Hz, 1H), 5.54 (dd, *J* = 10.5, 3.7 Hz, 1H), 5.39 (dd, *J* = 9.8, 7.8 Hz, 1H), 5.22 (d, *J* = 7.8 Hz, 1H), 4.71 (dd, *J* = 10.5, 3.5 Hz, 1H), 4.63 (d, *J* = 4.4 Hz, 2H), 4.32–4.22 (m, 2H), 3.74 (s, 3H), 1.16 (d, *J* = 6.5 Hz, 3H); ^{13}C NMR (126 MHz, $CDCl_3$) δ 166.25, 166.19, 165.85, 165.64, 165.23, 164.63, 155.45, 150.98, 133.52, 133.38, 133.26, 133.18, 133.05, 132.74, 130.15, 130.01, 129.96, 129.85, 129.78, 129.72, 129.47, 129.32, 128.98, 128.95, 128.84, 128.58, 128.50, 128.48, 128.32, 128.08, 118.52, 114.75, 101.75, 96.63, 74.03, 73.69, 72.97, 72.35, 72.09, 70.76, 69.98, 66.25, 63.33, 55.77, 16.46; ESI-HRMS (*m/z*) calcd for $C_{61}H_{56}NO_{17}$ [M + NH_4]$^+$ 1074.3543, found 1074.3544.

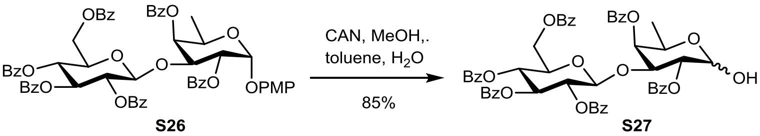

Disaccharide S27 | To a stirred solution of **S26** (1.41 g, 1.33 mmol, 1.0 equiv.) in MeOH/toluene/H_2O (10 mL each), was added ceric ammonium nitrate (CAN) (3.65 g, 6.66 mmol, 5.0 equiv.). The mixture was stirred at 25 °C for 7 hours, the excess reagents were quenched with $NaHCO_3$ (10 mL), and the resulting mixture was extracted with EtOAc (3 × 10 mL). The combined organic phases were washed with brine (20 mL), dried over anhydrous Na_2SO_4, filtered, and concentrated under vacuum. The residue was purified by flash column chromatography (EtOAc/petroleum ether/toluene, 1:1:2) to give **S27** (914 mg, 85%) as an orange foam. The α/β anomers were difficult to separate.

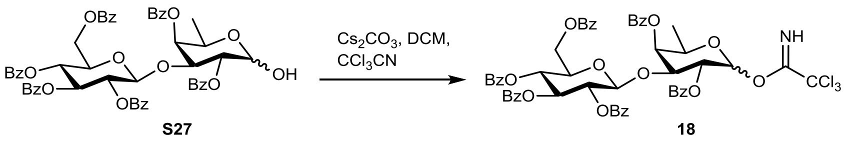

Disaccharide donor 18 | To a stirred solution of **S27** (114 mg, 0.12 mmol, 1.0 equiv.) in CH_2Cl_2 (2.0 mL), were added Cs_2CO_3 (4.0 mg, 0.01 mmol, 0.1 equiv.) and trichloroacetonitrile (173 mg, 1.2 mmol, 10.0 equiv.). The mixture was stirred at 25 °C for 2 hours before it was filtered and concentrated under vacuum. The residue was used for the next step without further purification.

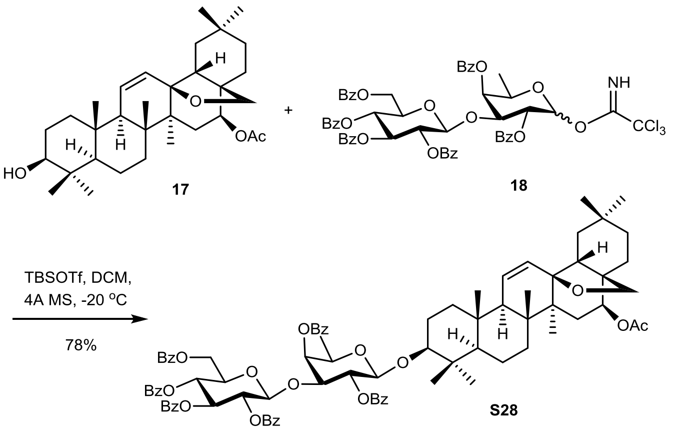

PT 3-*O*-disaccharide S28 | To a stirred solution of **17** (20 mg, 0.04 mmol, 1.0 equiv.), donor **18** (65 mg, 0.06 mmol, 1.5 equiv.), and 4Å molecular sieves (120 mg) in CH_2Cl_2 (1.0 mL), was added TBSOTf (1.06 mg, 0.004 mmol, 0.1 equiv.) at -20 ℃. The mixture was stirred at the same temperature for 2 hours, the excess reagents were quenched with Et_3N (5.0 mL). The mixture was filtered through a pad of celite and washed with CH_2Cl_2 (3 × 2 mL). The filtrate was concentrated under vacuum. The residue was purified by flash column chromatography (EtOAc/petroleum ether, 1:3) to give **S28** (45 mg, 78%) as a white foam. R_f = 0.5 (silica, PE/EtOAc = 2:1); $[\alpha]_D^{25}$ = 67.0 (*c* 1.0, $CHCl_3$); IR (film) ν_{max} = 2924, 2855, 1732, 1263, 1069, 708 cm^{-1}; ^1H NMR (500 MHz, $CDCl_3$) δ 8.10–8.05 (m, 2H), 7.99–7.96 (m, 2H), 7.84–7.81 (m, 2H), 7.75–7.72 (m, 2H), 7.67–7.63 (m, 2H), 7.57–7.51 (m, 1H), 7.49–7.24 (m, 15H), 7.19–7.15 (m, 2H), 7.10–7.05 (m, 2H), 5.86–5.82 (m, 1H), 5.72–5.66 (m, 1H), 5.62 (d, *J* = 3.4 Hz, 1H), 5.58–5.48 (m, 2H), 5.42–5.35 (m, 2H), 5.31 (dd, *J* = 9.8, 7.8 Hz, 1H), 4.99 (d, *J* = 7.8 Hz, 1H), 4.59–4.50 (m, 3H), 4.18 (dd, *J* = 10.1, 3.6 Hz, 1H), 4.10–4.02 (m, 1H), 3.95 (d, *J* = 7.4 Hz, 1H), 3.74 (q, *J* = 6.4 Hz, 1H), 3.16 (d, *J* = 7.3 Hz, 1H), 2.98 (dd, *J* = 11.6, 4.5 Hz, 1H), 2.04 (s, 3H), 1.22 (d, *J* = 6.4 Hz, 3H), 1.02 (s, 3H), 0.98 (s, 3H), 0.97 (s, 3H), 0.88 (s, 3H), 0.80 (s, 3H), 0.54 (s, 3H), 0.51 (s, 3H); ^{13}C NMR (126 MHz, $CDCl_3$) δ

170.82, 166.49, 166.19, 165.81, 165.13, 164.73, 164.53, 133.46, 133.22, 133.16, 132.96, 132.87, 132.66, 130.29, 129.93, 129.91, 129.87, 129.81, 129.78, 129.75, 129.63, 129.61, 129.54, 129.01, 128.92, 128.78, 128.46, 128.36, 128.30, 128.28, 128.05, 103.50, 101.48, 89.78, 83.91, 77.98, 73.21, 72.98, 72.84, 72.21, 71.98, 71.91, 69.86, 69.74, 68.86, 63.05, 55.17, 52.53, 51.68, 45.32, 45.26, 41.87, 39.01, 38.38, 37.46, 36.10, 34.32, 33.48, 31.98, 31.55, 31.45, 27.48, 25.82, 25.46, 23.78, 21.36, 20.55, 19.53, 17.82, 17.48, 16.88, 15.55; ESI-HRMS (*m/z*) calcd for $C_{86}H_{94}O_{19}Na$ $[M + Na]^+$ 1453.6282, found 1453.6275.

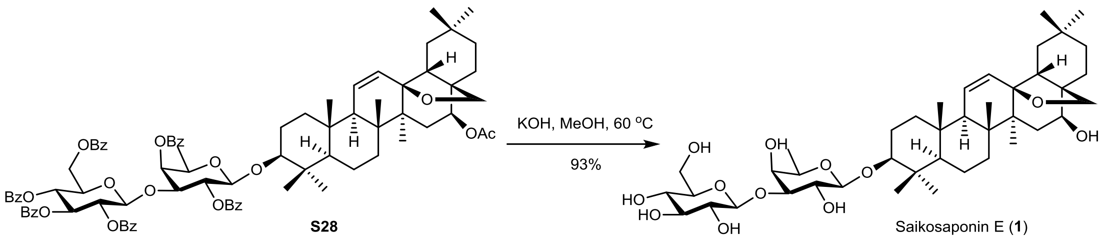

Saikosaponin E (1) | To a stirred solution of **S28** (18 mg, 0.01 mmol, 1.0 equiv.) in MeOH (2.5 mL), was added KOH (70 mg, 1.25 mmol, 125 equiv.) at 25 °C. The mixture was stirred at 50 ℃ for 8 hours, the excess reagents were quenched with acetic acid (0.07 mL, 125 equiv.). The mixture was filtered through a pad of celite and washed with MeOH (3 × 2.0 mL). The filtrate was concentrated under vacuum. The residue was purified by reversed-phase silica gel column chromatography (ODS RP-18) (MeOH/H_2O, 4:1 to 9:1) to give **1** (9.0 mg, 93%) as a white powder. R_f = 0.4 (ODS RP-18, MeOH/H_2O = 9:1); $[\alpha]_D^{25}$ = 48.2 (*c* 0.5, MeOH); IR (film) ν_{max} = 2961, 2929, 1733, 1263, 1073, 802 cm^{-1}; ^1H NMR (500 MHz, pyridine-d5) δ 5.98 (d, *J* = 10.4 Hz, 1H), 5.68 (dd, *J* = 10.3, 2.7 Hz, 1H), 5.41 (d, *J* = 7.8 Hz, 1H), 4.77 (d, *J* = 7.7 Hz, 1H), 4.59–4.51 (m, 3H), 4.42 (d, *J* = 6.9 Hz, 1H), 4.37 (dd, *J* = 11.8, 5.4 Hz, 1H), 4.32–4.15 (m, 4H), 4.06 (t, *J* = 8.4 Hz, 1H), 4.04–3.98 (m, 1H), 3.80 (q, *J* = 6.3 Hz, 1H), 3.31–3.38 (m, 2H), 2.55–2.49 (m, 1H), 2.29–2.19 (m, 1H), 2.11–2.04 (m, 1H), 1.49 (d, *J* = 6.3 Hz, 3H), 1.37 (s, 3H), 1.32 (s, 3H), 1.17 (s, 3H), 0.97 (s, 6H), 0.91 (s, 6H); ^{13}C NMR (126 MHz, pyridine-d5) δ 132.03, 131.23, 106.82, 106.72, 88.62, 85.15, 83.95, 78.72, 78.40, 75.83, 73.02, 72.17, 71.72, 71.53, 70.96, 64.01, 62.67, 55.30, 52.88, 52.13, 47.00, 45.63, 42.19, 39.74, 38.56, 37.76, 36.34, 36.12, 34.69, 33.66, 31.85, 31.61, 27.80, 26.57, 25.74, 23.80, 20.89, 19.98, 18.20, 17.87, 17.28, 16.32.

Synthesis of Albizia saponin (2)

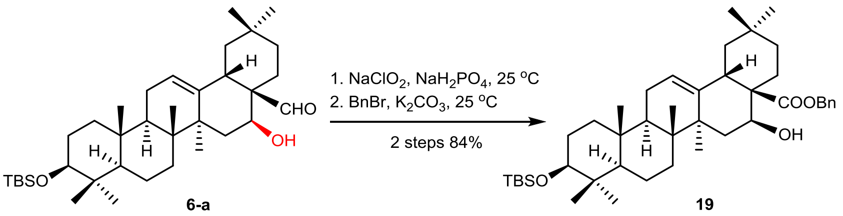

Compound 19 | To a solution of aldehyde **6-a** (1.24 g, 2.17 mmol, 1.0 equiv.) and DMSO (4 mL) in *t*-BuOH (20 ml), were added a solution of $NaClO_2$ (80% purity, 1.72 g, 15.2 mmol, 7.0 equiv.) and $NaH_2PO_4•2H_2O$ (2.37 g, 15.2 mmol, 7.0 equiv.) in water (10 mL). After stirring at 30 ℃ for 4 h, the reaction mixture was diluted with an aqueous 10% NaOH solution (20 mL) and the aqueous phase was extracted with hexanes (50 ml). The aqueous phase was acidified with aqueous 1M HCl

solution (to pH = 1) and extracted with CH_2Cl_2 (3 × 80 mL). The combined CH_2Cl_2 layers were dried (Na_2SO_4) and concentrated under reduced pressure. A mixture of the residue and K_2CO_3 (900 mg, 6.51 mmol, 3.0 equiv.) in DMF (10 mL) was stirred at 25 °C for 20 min before BnBr (0.3 mL, 2.6 mmol, 1.2 equiv.) was added. After stirring at 25 °C for 4 h, the excess reagents were quenched with $NaHCO_3$ (aq.), and then the resulting mixture was extracted with CH_2Cl_2 (3 × 30 mL). The combined organic layers were washed with saturated aqueous NaCl solution (100 mL), dried (Na_2SO_4), and concentrated under reduced pressure. Flash column chromatography (EtOAc/petroleum ether, 1:20) afforded **19** (1.24 g, 84%) as a white solid. R_f = 0.4 (silica, PE/EtOAc = 10:1); $[\alpha]_D^{25}$ = 23.1 (*c* 1.2, $CHCl_3$); ^{1}H NMR (500 MHz, $CDCl_3$) δ 7.41–7.29 (m, 5H), 5.29 (t, *J* = 3.7 Hz, 1H), 5.15–5.03 (m, 2H), 4.22–4.09 (m, 1H), 3.42 (d, *J* = 11.6 Hz, 1H), 3.17 (dd, *J* = 11.5, 4.5 Hz, 1H), 3.07–3.03 (m, 1H), 2.28–2.24 (m, 1H), 1.89–1.20 (m, 17H), 1.18 (s, 3H), 1.17–1.10 (m, 1H), 0.95 (s, 3H), 0.92 (s, 3H), 0.90 (s, 3H), 0.88 (s, 9H), 0.86 (s, 3H), 0.74 (s, 3H), 0.68 (dd, *J* = 11.6, 1.8 Hz, 1H), 0.58 (s, 3H), 0.03 (s, 6H); ^{13}C NMR (126 MHz, $CDCl_3$) δ 177.95, 142.23, 135.53, 128.53, 128.22, 128.09, 123.16, 79.37, 66.33, 64.75, 55.24, 50.54, 46.75, 45.36, 43.93, 43.33, 39.42, 39.31, 38.42, 37.38, 36.78, 33.19, 33.03, 32.67, 30.54, 28.52, 27.58, 26.81, 26.62, 25.91, 23.94, 23.42, 18.44, 18.11, 16.83, 16.08, 15.29, -3.76, -4.91; ESI-HRMS (*m/z*) calcd for $C_{43}H_{68}O_4SiNa$ $[M + Na]^+$ 699.4779, found 699.4777.

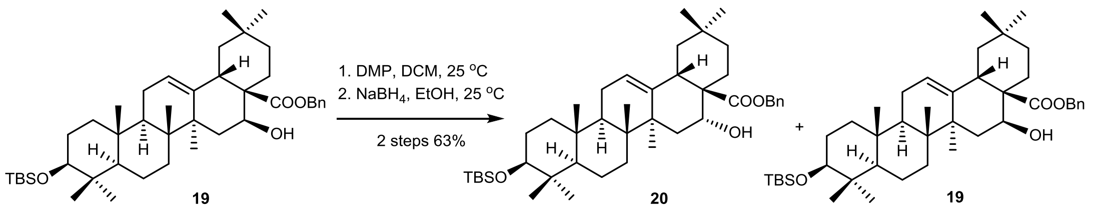

dr = 5:1

Compound 20 | To a solution of **19** (300mg, 0.44 mmol, 1.0 equiv.) in CH_2Cl_2 (8 mL), was added Dess-Martin periodinane (374 mg, 0.4 mmol, 1.0 equiv.) at 0 ℃. After stirring at 25 °C for 3 h, the excess reagents were quenched with saturated aqueous $Na_2S_2O_3$ solution, and then the resulting mixture was extracted with CH_2Cl_2 (3 × 30 mL). The combined organic layers were washed with saturated aqueous NaCl solution (100 ml), dried (Na_2SO_4), and concentrated under reduced pressure. The residue was dissolved in EtOH (10 mL), to which was added $NaBH_4$ (83 mg, 2.2 mmol, 5.0 equiv.). After stirring at 25 °C overnight, the excess reagents were quenched with aqueous 1M HCl solution, and then the resulting mixture was extracted with CH_2Cl_2 (3 × 30 mL). The combined organic layers were washed with saturated aqueous NaCl solution (100 mL), dried (Na_2SO_4), and concentrated under reduced pressure. The residue was purified by flash column chromatography (EtOAc/petroleum ether, 1:50) to give **20** (216 mg, 74% for 2 steps) as a white solid and recovered **19** (14%). **Compound 20**: R_f = 0.5 (silica, PE/EtOAc = 10:1); $[\alpha]_D^{25}$ = 24.7 (*c* 1.0, $CHCl_3$); ^{1}H NMR (500 MHz, $CDCl_3$) δ 7.39–7.27 (m, 5H), 5.38 (t, *J* = 3.7 Hz, 1H), 5.15–4.92 (m, 2H), 4.55 (d, *J* = 3.9 Hz, 1H), 3.19 (dd, *J* = 11.1, 4.5 Hz, 1H), 3.11–3.07 (m, 1H), 2.19–2.13 (m, 1H), 1.34 (s, 3H), 0.97 (s, 3H), 0.91 (s, 3H), 0.90 (s, 3H), 0.89 (s, 9H), 0.89 (s, 3H), 0.74 (s, 3H), 0.72–0.68 (m, 1H), 0.60 (s, 3H), 0.04 (s, 6H); ^{13}C NMR (126 MHz, $CDCl_3$) δ 176.52, 142.59, 136.05, 128.44, 128.05, 128.03, 123.07, 79.42, 74.99, 66.32, 55.36, 48.81, 46.75, 46.35, 41.32, 40.71, 39.52, 39.32, 38.52, 36.89, 35.51, 35.44, 33.01, 32.79, 30.59, 30.39, 28.51, 27.63, 27.02, 25.93, 24.71, 23.32, 18.47, 18.12, 16.98, 16.13, 15.49, -3.74, -4.90; ESI-HRMS (*m/z*) calcd for $C_{43}H_{68}O_4SiNa$ $[M + Na]^+$ 699.4779, found 699.4782.

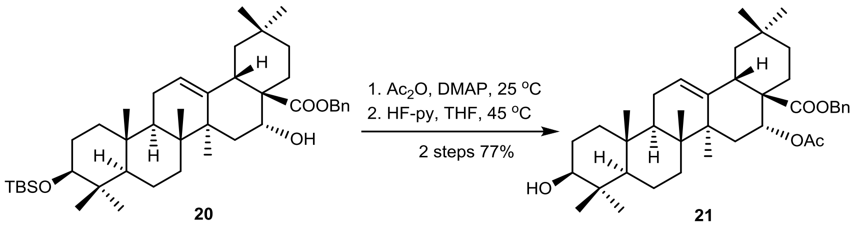

Compound 21 | To a solution of **20** (280 mg, 0.41 mmol, 1.0 equiv.) and DMAP (25 mg, 0.21 mmol, 0.5 equiv.) in pyridine (4 mL), was added Ac_2O (117 μL, 1.23 mmol, 3.0 equiv.). After stirring at 25 °C for 3 hours, the mixture was poured into water, the excess reagents were quenched with Na_2CO_3. The mixture was extracted with CH_2Cl_2 (3 × 20 mL). The organic layer was washed with saturated aqueous NaCl solution (50 mL), dried (Na_2SO_4), and concentrated under reduced pressure. The residue was used in the next step without further purification. To a solution of the residue in THF (4 mL), was added hydrogen fluoride-pyridine (65% wt.%, 0.4 mL, 2.9 mmol, 7.0 equiv.) at 0 ℃. The mixture was heated to 45 ℃ and stirred for 12 h, the excess reagents were slowly quenched with saturated aqueous $NaHCO_3$ solution. The mixture was extracted with CH_2Cl_2 (3 × 20 mL), and the combined organic layers were washed with saturated aqueous NaCl solution (50 ml), dried (Na_2SO_4), and concentrated under reduced pressure. Flash column chromatography (EtOAc/petroleum ether, 1:6) afforded **21** (190 mg, 77% for 2 steps) as a white solid: R_f = 0.2 (silica, PE/EtOAc = 5:1); $[\alpha]_D^{25}$ = -8.2 (*c* 1.0, $CHCl_3$); ^{1}H NMR (500 MHz, $CDCl_3$) δ 7.37–7.28 (m, 5H), 5.68–5.66 (m, 1H), 5.39 (t, *J* = 3.7 Hz, 1H), 5.11–5.02 (m, 2H), 3.20 (dd, *J* = 11.3, 4.3 Hz, 1H), 3.12–3.09 (m, 1H), 2.08 (s, 3H), 1.22 (s, 3H), 0.97 (s, 3H), 0.97 (s, 3H), 0.92 (s, 3H), 0.88 (s, 3H), 0.76 (s, 3H), 0.69 (dd, *J* = 11.6, 1.9 Hz, 1H), 0.53 (s, 3H); ^{13}C NMR (126 MHz, $CDCl_3$) δ 175.17, 169.94, 141.74, 135.77, 128.48, 128.26, 128.16, 123.71, 78.92, 76.44, 66.60, 55.24, 47.54, 46.65, 46.15, 41.05, 40.46, 39.40, 38.72, 38.47, 36.98, 35.04, 33.15, 32.92, 32.08, 30.95, 30.40, 28.07, 27.16, 26.42, 24.16, 23.27, 22.01, 18.21, 16.81, 15.57, 15.40; ESI-HRMS (*m/z*) calcd for $C_{39}H_{60}NO_5$ $[M + NH_4]^+$ 622.4466, found 622.4465.

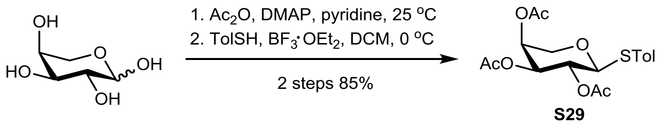

Compound S29[23] | To a stirred solution of L-arabinose (12 g, 80 mmol, 1.0 equiv.) in pyridine (50 mL), were added Ac_2O (46 mL, 480 mmol, 6.0 equiv.) and DMAP (100 mg, 0.82 mmol, 0.01 equiv.). The mixture was stirred at 25 °C for 2 hours, the excess reagents were quenched with $NaHCO_3$ (200 mL) and the resulting mixture was extracted with EtOAc (3 × 100 mL). The combined organic phases were washed with brine (150 mL), dried over anhydrous Na_2SO_4, filtered, and concentrated under vacuum. The residue and *p*-thiocresol (14.6 g, 117.8 mmol, 1.5 equiv.) were dissolved in CH_2Cl_2 (100 mL), to which was added boron trifluoride diethyl etherate (14.9 ml, 117.8 mmol, 1.5 equiv.) at 0 ℃. The solution was stirred at 0 ℃ for 8 h, and then was diluted with CH_2Cl_2 (100 mL). The mixture was washed with H_2O (100 mL), saturated aqueous $NaHCO_3$ solution (200 mL), and saturated aqueous NaCl solution (100 mL). The organic phase was dried (Na_2SO_4) and concentrated under reduced pressure. Purification of the resulting residue by flash column chromatography (EtOAc/petroleum ether, 1:10) gave **S29** (26.14 g, 85%) as a

colorless oil: R_f = 0.5 (silica, PE/EtOAc = 3:1); ^{1}H NMR (500 MHz, $CDCl_3$) δ 7.40 (d, *J* = 8.1 Hz, 2H), 7.12 (d, *J* = 8.0 Hz, 2H), 5.28–5.25 (m, 1H), 5.23 (d, *J* = 8.2 Hz, 1H), 5.09 (dd, *J* = 8.5, 3.4 Hz, 1H), 4.74 (d, *J* = 8.0 Hz, 1H), 4.17–4.13 (m, 1H), 3.67–3.64 (m, 1H), 2.33 (s, 3H), 2.10 (s, 6H), 2.05 (s, 3H).

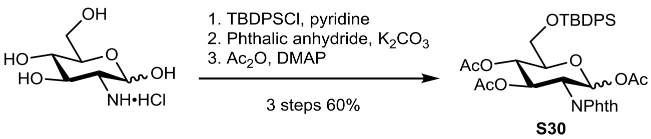

S30

Compound S30 | D-Glucosamine hydrochloride (5.0 g, 23.2 mmol, 1.0 equiv.) and TBDPSCl (8.3 g, 30.2 mmol, 1.3 equiv.) were dissolved in pyridine (50 mL). The mixture was stirred at 25 °C for 60 h, and was then diluted with CH_2Cl_2 (30 mL) and washed with H_2O (50 mL) and saturated aqueous NaCl solution(50 mL). The organic layer was concentrated under reduced pressure. The resulting residue was purified by flash column chromatography (petroleum ether/EtOAc then CH_2Cl_2/MeOH, 10:1) to give a light yellow oil. To a solution of the residue and phthalic anhydride (3.16 g, 21.3 mmol, 1.0 equiv.) in CH_2Cl_2 (100 mL), was added K_2CO_3 (8.04 g, 58.2 mmol, 2.5 equiv.). After stirring at 25 °C overnight, the mixture was filtered. The filtrates were concentrated under reduced pressure. The resulting residue was dissolved in pyridine (50 mL), to which was added Ac_2O (7.4 mL, 77.6 mmol, 3.3 equiv.). After stirring at 25 °C overnight, the mixture was poured into ice water, the excess reagents were quenched with sodium bicarbonate. The mixture was extracted with CH_2Cl_2 (3 × 50 mL). The organic layer was washed with saturated aqueous NaCl solution (100 mL), dried (Na_2SO_4), and concentrated under reduced pressure. Flash column chromatography (EtOAc/petroleum ether, 1:6) afforded **S30** (9.3 g, 71%, α/β = 3:2) as a white foamy solid.

The β anomer: R_f = 0.3 (silica, PE/EtOAc = 5:1); $[\alpha]_D^{25}$ = 57.7 (*c* 1.2, $CHCl_3$); ^{1}H NMR (500 MHz, $CDCl_3$) δ 7.87 (dd, *J* = 5.5, 3.0 Hz, 2H), 7.75 (dd, *J* = 5.5, 3.0 Hz, 2H), 7.70–7.65 (m, 4H), 7.46–7.33 (m, 6H), 6.50 (d, *J* = 8.8 Hz, 1H), 5.87 (dd, *J* = 10.6, 9.1 Hz, 1H), 5.34–5.25 (m, 1H), 4.46 (dd, *J* = 10.6, 8.9 Hz, 1H), 3.85 (ddd, *J* = 10.1, 4.4, 2.1 Hz, 1H), 3.82–3.74 (m, 1H), 2.02 (s, 3H), 1.90 (s, 3H), 1.86 (s, 3H), 1.07 (s, 9H); ^{13}C NMR (126 MHz, $CDCl_3$) δ 170.45, 169.50, 168.87, 135.97, 135.91, 134.63, 133.34, 129.94, 129.90, 127.89, 127.83, 123.98, 89.96, 75.27, 71.11, 68.85, 62.53, 53.86, 26.90, 21.04, 20.82, 20.71, 19.46; ESI-HRMS (m/z) calcd for $C_{36}H_{39}NO_{12}SiNa$ $[M + Na]^+$ 696.2235, found 696.2238.

The α anomer: R_f = 0.3 (silica, PE/EtOAc = 5:1); $[\alpha]_D^{25}$ = 84.8 (*c* 1.1, $CHCl_3$); ^{1}H NMR (500 MHz, $CDCl_3$) δ 7.85 (dd, *J* = 5.3, 3.0 Hz, 2H), 7.76–7.72 (m, 2H), 7.67 (dd, *J* = 7.8, 1.5 Hz, 2H), 7.64 (dd, *J* = 7.9, 1.3 Hz, 2H), 7.45–7.36 (m, 6H), 6.55 (dd, *J* = 11.6, 9.2 Hz, 1H), 6.33 (d, *J* = 3.4 Hz, 1H), 5.38–5.27 (m, 1H), 4.71 (dd, *J* = 11.6, 3.4 Hz, 1H), 4.15 (dt, *J* = 10.3, 2.7 Hz, 1H), 3.77–3.76 (m, 2H), 2.04 (s, 3H), 1.94 (s, 3H), 1.88 (s, 3H), 1.06 (s, 9H); ^{13}C NMR (126 MHz, $CDCl_3$) δ 169.82, 169.65, 169.48, 135.68, 135.65, 134.37, 132.91, 129.75, 129.71, 127.70, 123.69, 90.69, 72.73, 69.53, 67.40, 61.99, 53.01, 26.72, 21.02, 20.77, 20.65, 19.22; ESI-HRMS (m/z) calcd for $C_{36}H_{39}NO_{12}SiNa$ $[M + Na]^+$ 696.2235, found 696.2238.

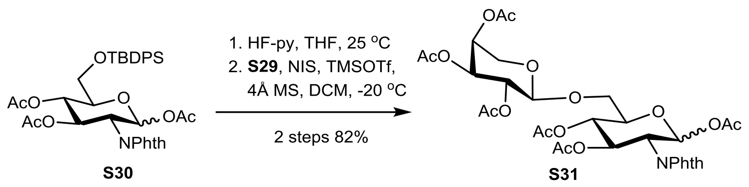

Disaccharide S31 | To a solution of **S30** (2.0 g, 3.0 mmol, 1.0 equiv.) in THF (30 mL), was added hydrogen fluoride-pyridine (65% wt.%, 3 mL, 21.7 mmol, 7.2 equiv.) at 25 °C. After stirring for 4 h, the excess reagents were slowly quenched with saturated aqueous $NaHCO_3$ solution. The mixture was extracted with CH_2Cl_2 (3 × 30 mL), and the combined organic layers were washed with saturated aqueous NaCl solution (100 mL), dried (Na_2SO_4), and concentrated under reduced pressure. Flash column chromatography (petroleum ether/EtOAc, 5:1 to 3:2) afforded a white foamy solid. A mixture of the solid (862 mg, 1.98 mmol, 1.0 equiv.), **S29** (1.14 g, 2.97 mmol, 1.5 equiv.), and 4Å MS in dry CH_2Cl_2 (10 mL) was stirred at 25 °C for 30 min, and then cooled to -20 ℃. NIS (668 mg, 2.98 mmol, 1.5 equiv.) was added, followed by Me_3SiOTf (0.32 mL, 2.57 mmol, 1.3 equiv.). The mixture was stirred for another 40 min before addition of Et_3N (1 mL) and then filtered through a pad of celite. The filtrate was concentrated under reduced pressure. Purification of the resulting residue by flash column chromatography (EtOAc/petroleum ether, 1:3) gave **S31** (1.24 g, 90%, α/β= 6:1) as a light yellow syrup: R_f = 0.6 (silica, PE/EtOAc = 1:1); ^1H NMR (500 MHz, $CDCl_3$) δ 7.90–7.78 (m, 14H), 7.76–7.71 (m, 14H), 6.54 (dd, *J* = 11.6, 9.1 Hz, 6H), 6.46 (d, *J* = 8.9 Hz, 1H), 6.25 (d, *J* = 3.3 Hz, 6H), 5.86 (dd, *J* = 10.6, 9.1 Hz, 1H), 5.21–5.01 (m, 21H), 4.98 (dd, *J* = 5.3, 1.5 Hz, 6H), 4.96 (dd, *J* = 5.3, 1.4 Hz, 1H), 4.66 (dd, *J* = 11.6, 3.4 Hz, 6H), 4.48–4.17 (m, 28H), 3.86–3.78 (m, 7H), 3.70–3.63 (m, 7H), 2.15 (s, 21H), 2.11 (s, 3H), 2.10 (s, 18H), 2.08 (s, 18H), 2.07 (s, 18H), 2.06 (s, 18H), 2.04 (s, 3H), 1.97 (s, 3H), 1.85 (s, 18H), 1.84 (s, 3H), 1.71 (s, 3H); ^{13}C NMR (126 MHz, $CDCl_3$) δ 170.84, 170.82, 170.55, 170.49, 170.23, 169.89, 169.78, 169.77, 169.69, 169.64, 169.60, 168.73, 134.62, 131.33, 123.89, 106.25, 106.07, 90.64, 89.83, 81.32, 80.62, 77.43, 77.17, 77.11, 74.00, 71.63, 70.75, 70.02, 69.30, 67.26, 66.22, 65.65, 63.42, 53.69, 52.99, 21.18, 21.00, 20.97, 20.96, 20.93, 20.88, 20.61; ESI-HRMS (m/z) calcd for $C_{31}H_{35}NO_{17}Na$ $[M + Na]^+$ 716.1797, found 716.1802.

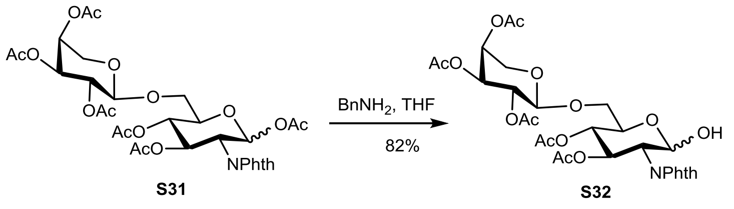

Disaccharide S32 | To a solution of **S31** (8.7 g, 12.5 mmol, 1.0 equiv.) in THF (40 mL), was added $BnNH_2$ (4.1 ml, 37.5 mmol, 3.0 equiv.). The mixture was stirred at 25 °C for 4 h, and was then diluted with CH_2Cl_2 (50 mL), and washed with H_2O (50 mL) and saturated aqueous NaCl solution (50 mL). The organic layer was concentrated under reduced pressure. Purification of the resulting residue by flash column chromatography (EtOAc/petroleum ether, 1:1) gave **S32** (6.7 g, 82%) as a yellow syrup. The α/β anomers were difficult to separate.

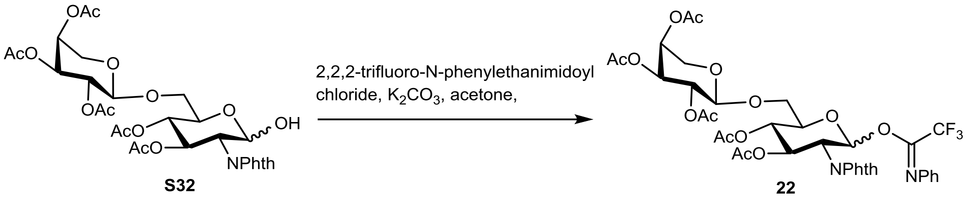

Disaccharide donor 22 | To a stirred solution of **S32** (140 mg, 0.21 mmol, 1.0 equiv.) in acetone (2.0 mL), were added 2,2,2-trifluoro-*N*-phenylethanimidoyl chloride (89 mg, 0.42 mmol, 2.0 equiv.) and K_2CO_3 (89 mg, 0.64 mmol, 3.0 equiv.). The mixture was stirred at 25 °C for 2 h before it was filtered and concentrated under vacuum. The residue was used for next step without further purification.

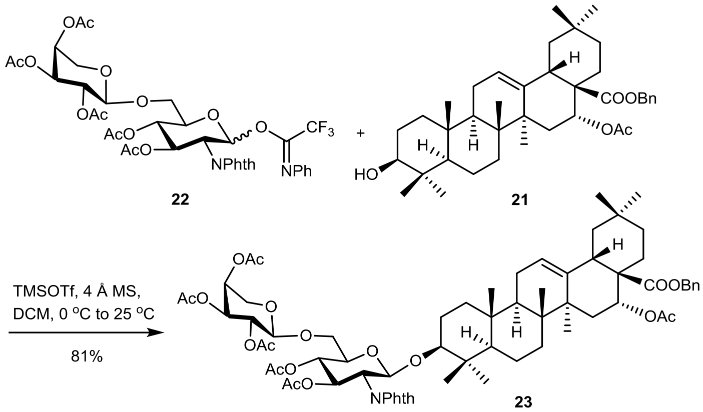

PT 3-*O*-disaccharide 23 | A mixture of the **21** (68 mg, 0.18 mmol, 1.0 equiv.), donor **22** (140 mg, 0.17 mmol, 1.0 equiv.), and 4Å MS in dry CH_2Cl_2 (2 mL) was stirred at 25 °C for 20 min, and then cooled to 0 ˚C. TMSOTf (8.0 mg, 0.04 mmol, 0.2 equiv.) was added. The mixture was warmed to 25 °C and stirred for another 1.5 hour before addition of Et_3N (1 mL). The mixture was filtered through a pad of Celite, the filtrate was concentrated under reduced pressure. Purification of the resulting residue by flash column chromatography (EtOAc/petroleum ether, 1:2) gave **23** (110 mg, 81%) as a white solid: R_f = 0.4 (silica, PE/EtOAc = 1:1); $[\alpha]_D^{25}$ = 12.8 (*c* 1.0, $CHCl_3$); ^1H NMR (500 MHz, $CDCl_3$) δ 7.83 (m, 2H), 7.72 (m, 2H), 7.35–7.27 (m, 5H), 5.79 (dd, *J* = 10.8, 9.0 Hz, 1H), 5.62 (m, 1H), 5.38–5.33 (m, 2H), 5.26 (td, *J* = 3.6, 1.9 Hz, 1H), 5.17 (dd, *J* = 9.1, 6.6 Hz, 1H), 5.11–4.95 (m, 4H), 4.55 (d, *J* = 6.7 Hz, 1H), 4.29 (dd, *J* = 10.8, 8.4 Hz, 1H), 4.06–4.02 (m, 1H), 3.89–3.82 (m, 2H), 3.71 (dd, *J* = 11.8, 7.3 Hz, 1H), 3.63–3.60 (m, 1H), 3.14–2.99 (m, 2H), 2.14 (s, 3H), 2.09 (s, 3H), 2.06 (s, 3H), 2.05 (s, 3H), 2.02 (s, 3H), 1.84 (s, 3H), 1.15 (s, 3H), 0.95 (s, 3H), 0.90 (s, 3H), 0.82 (s, 3H), 0.56 (s, 3H), 0.46 (s, 3H), 0.39 (s, 3H); ^{13}C NMR (126 MHz, $CDCl_3$) δ 175.13, 170.24, 170.16, 169.99, 169.88, 169.60, 169.47, 169.18, 141.55, 135.73, 134.27, 128.44, 128.23, 128.13, 123.79, 123.46, 100.45, 99.67, 90.25, 76.43, 73.37, 70.70, 70.05, 69.71, 68.96, 67.52, 67.46, 66.58, 63.23, 62.83, 55.21, 54.89, 47.51, 46.60, 46.13, 40.96, 40.43, 39.35, 38.34, 38.24, 36.62, 35.02, 33.12, 32.81, 31.98, 30.93, 30.37, 27.45, 26.30, 26.27, 25.50, 24.15, 23.21, 21.97, 20.92, 20.88, 20.67, 20.64, 20.46, 17.91, 16.74, 16.33, 15.28; ESI-HRMS (m/z) calcd for $C_{68}H_{87}NO_{20}Na$ $[M + Na]^+$ 1260.5714, found 1260.5717.

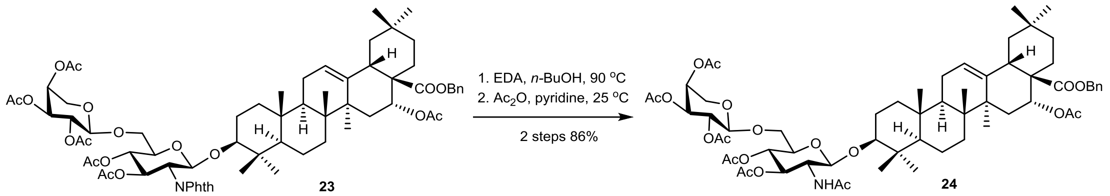

Compound 24 | A solution of **23** (80 mg, 0.064 mmol, 1.0 equiv.) in BuOH (2.5 mL) and $NH_2CH_2CH_2NH_2$ (2.5 mL) was stirred at 90 ℃ overnight, and was then concentrated to give a residue. The residue was co-evaporated with EtOH twice and then dissolved in pyridine-Ac_2O (2 mL, 1:1). The resulting mixture was stirred at 25 °C for 4 h and then concentrated to give a residue, which was subjected to silica gel column chromatography (acetone/EtOAc/petroleum ether, 1:2:5) to provide **24** (64 mg, 86%) as a white amorphous solid: R_f = 0.5 (silica, PE/EtOAc = 6:1); $[\alpha]_D^{25}$ = -14.9 (*c* 1.0, $CHCl_3$); ^{1}H NMR (500 MHz, $CDCl_3$) δ 7.40–7.27 (m, 5H), 5.66 (d, *J* = 3.4 Hz, 1H), 5.53 (d, *J* = 8.9 Hz, 1H), 5.39 (dd, *J* = 3.0, 2.4 Hz, 1H), 5.28–5.19 (m, 2H), 5.14 (dd, *J* = 9.1, 6.6 Hz, 1H), 5.11–4.94 (m, 4H), 4.93–4.86 (m, 1H), 4.64 (d, *J* = 8.3 Hz, 1H), 4.52 (d, *J* = 6.7 Hz, 1H), 3.09 (dd, *J* = 6.6, 4.9 Hz, 2H), 2.13 (s, 3H), 2.09–2.06 (m, 6H), 2.03 (s, 3H), 2.01 (s, 3H), 2.01 (s, 3H), 1.91 (s, 3H), 1.25 (s, 3H), 1.19 (s, 3H), 0.96 (s, 3H), 0.91 (s, 3H), 0.89 (s, 3H), 0.87 (s, 3H), 0.75 (s, 3H), 0.66 (d, *J* = 11.2 Hz, 1H), 0.51 (s, 3H); ^{13}C NMR (126 MHz, $CDCl_3$) δ 175.14, 171.01, 170.21, 169.97, 169.88, 169.86, 169.48, 169.16, 141.54, 135.75, 128.46, 128.43, 128.25, 128.16, 123.86, 123.67, 102.97, 100.41, 90.09, 77.25, 77.20, 77.16, 76.99, 76.74, 76.46, 73.37, 72.31, 70.04, 69.23, 68.99, 67.58, 67.41, 66.60, 63.16, 62.76, 55.58, 55.28, 53.79, 47.54, 46.70, 46.19, 41.01, 40.46, 39.42, 38.92, 38.47, 36.67, 35.03, 33.13, 32.96, 32.01, 30.94, 30.38, 29.67, 29.64, 29.26, 27.92, 27.87, 26.35, 26.29, 25.76, 24.16, 23.34, 23.23, 21.97, 20.90, 20.88, 20.80, 20.76, 20.69, 20.65, 20.61, 18.05, 16.79, 16.43, 15.33; ESI-HRMS (m/z) calcd for $C_{62}H_{91}N_2O_{19}$ $[M + NH_4]^+$ 1167.6211, found 1167.6210.

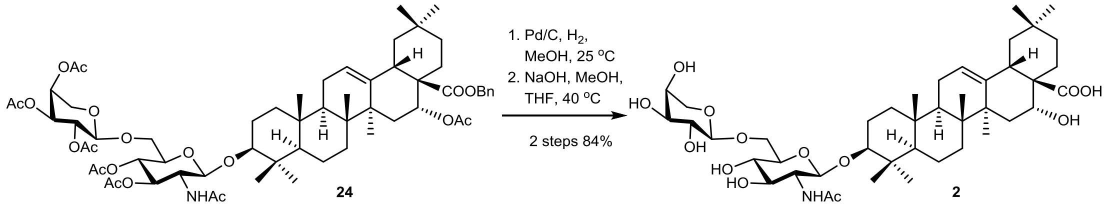

Albizia saponin 2 | To a solution of **24** (64 mg, 0.056 mmol, 1.0 equiv.) in MeOH (2 mL), was added Pd/C (14 mg). The mixture was stirred at 25 °C under a hydrogen atmosphere until the reaction was complete as determined by TLC. The catalyst was removed by filtration through Celite. The filtrate was concentrated under reduced pressure. The residue was dissolved in methanol/THF (3 mL, v/v 1:1), to which was added NaOH (45 mg, 1.13 mmol, 20.0 equiv.). The mixture was stirred at 40 ℃ for 12 h, the excess NaOH was neutralized with HCl (1 M), and the mixture was concentrated. The residue was purified by reversed-phase silica gel column chromatography (ODS RP-18) (MeOH/H_2O, 3:1) to afford **2** (38 mg, 84%) as a white amorphous solid: R_f = 0.5 (ODS RP-18, MeOH/H_2O = 2:1); $[\alpha]_D^{25}$ = 6.1 (*c* 1.0, MeOH); ^{1}H NMR (500 MHz, pyridine-d5) δ 8.92 (d, *J* = 9.0 Hz, 1H), 5.65–5.60 (m, 1H), 5.28 (d, *J* = 3.4 Hz, 1H), 4.99 (d, *J* = 8.3 Hz, 1H), 4.95 (d, *J* = 6.6 Hz, 1H), 4.88 (dd, *J* = 11.0, 1.9 Hz, 1H), 4.57 (d, *J* = 10.5 Hz, 1H), 4.48 (dd, *J* = 8.4, 6.6 Hz, 1H), 4.42–4.28 (m, 3H), 4.25 (s, 1H), 4.20 (dd, *J* = 8.5, 3.2 Hz, 1H), 4.14–4.06 (m, 2H), 3.77 (dd, *J* = 11.7, 1.5 Hz, 1H), 3.20 (dd, *J* = 11.7, 4.5 Hz, 1H), 2.95–2.81 (m, 1H), 2.15 (s, 3H), 1.85 (s, 3H), 1.21 (s, 3H), 1.13 (s, 3H), 1.10 (s, 3H), 1.03 (s, 3H), 1.00 (s, 3H),

0.83 (s, 3H); ^{13}C NMR (126 MHz, pyridine-d5) δ 180.45, 170.56, 145.52, 123.00, 105.81, 105.56, 89.81, 77.18, 76.57, 75.27, 74.86, 73.27, 72.79, 70.41, 69.57, 66.88, 58.34, 56.31, 49.39, 47.87, 47.64, 42.61, 41.94, 40.39, 39.76, 39.13, 37.50, 36.72, 36.66, 34.01, 33.89, 33.38, 31.58, 28.62, 27.75, 27.04, 25.26, 24.32, 24.20, 19.07, 17.96, 17.51, 16.00; ESI-HRMS (m/z) calcd for $C_{43}H_{70}NO_{13}$ $[M + H]^+$ 808.4842, found 808.4841.

Synthesis of Kochianoside (3)

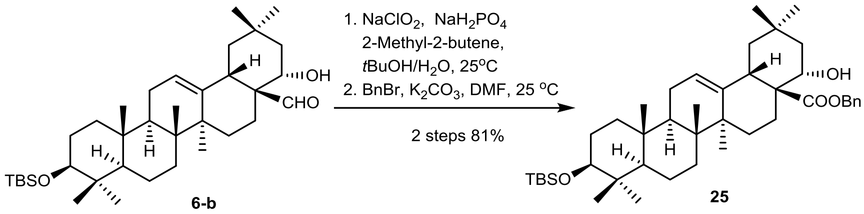

Compound 25 | To a solution of aldehyde **6-b** (220 mg, 0.38 mmol, 1.0 equiv.) and DMSO (2.5 mL) in *t*-BuOH (10 mL), was added a solution of $NaClO_2$ (80% purity, 305 mg, 2.7 mmol, 7.1 equiv.) and NaH_2PO_4 (342 mg, 2.7 mmol, 7.1 equiv.) in water (10 mL). After stirring at 30 ℃ for 4 h, the mixture was diluted with an aqueous 10% NaOH solution (20 mL), and the aqueous phase was extracted with hexanes (50 mL). The aqueous phase was acidified with aqueous 1M HCl solution (to pH = 1) and extracted with CH_2Cl_2 (3 × 80 mL). The combined CH_2Cl_2 layers were dried (Na_2SO_4) and concentrated under reduced pressure. The residue (100 mg, 0.2 mmol, 1.0 equiv.) and K_2CO_3 (85 mg, 0.6 mmol, 3.0 equiv.) in DMF (2 mL) were stirred at 25 °C for 10 min before BnBr (26 μL, 0.22 mmol, 1.1 equiv.) was added. After stirring for 2 h, the excess reagents werequenched with aqueous 1M HCl solution, and the resulting mixture was extracted with CH_2Cl_2 (3 × 30 mL). The combined organic layers were washed with saturated aqueous NaCl solution (100 mL), dried (Na_2SO_4), and concentrated under reduced pressure. Flash column chromatography (EtOAc/petroleum ether, 1:10) afforded **25** (116 mg, 81%, over 2 steps) as a white solid: R_f = 0.4 (silica, PE/EtOAc = 6:1); $[\alpha]_D^{25}$ = 22.6 (*c* 1.0, $CHCl_3$); ^1H NMR (500 MHz, $CDCl_3$) δ 7.40–7.29 (m, 5H), 5.28 (t, *J* = 3.5 Hz, 1H), 5.17–5.07 (m, 2H), 3.95–3.67 (m, 1H), 3.17 (dd, *J* = 11.3, 4.4 Hz, 1H), 2.91–2.87 (m, 1H), 2.34 (d, *J* = 2.6 Hz, 1H), 2.02–1.98 (m, 1H), 1.82 (dd, *J* = 9.2, 3.5 Hz, 2H), 1.74–1.67 (m, 2H), 1.14 (s, 3H), 0.96 (s, 3H), 0.94 (s, 3H), 0.89 (s, 3H), 0.88 (s, 9H), 0.86 (s, 3H), 0.74 (s, 3H), 0.68 (d, *J* = 10.6 Hz, 1H), 0.59 (s, 3H), 0.03 (s, 6H); ^{13}C NMR (126 MHz, $CDCl_3$) δ 176.71, 142.73, 135.95, 128.77, 128.42, 128.30, 123.37, 79.66, 70.98, 66.82, 55.48, 53.11, 47.78, 45.66, 42.38, 42.03, 41.69, 39.65, 39.54, 38.69, 37.09, 33.26, 32.94, 31.22, 28.75, 27.82, 27.43, 26.24, 26.14, 25.06, 23.65, 18.70, 18.34, 17.03, 16.91, 16.32, 15.54, -3.52, -4.68; ESI-HRMS (m/z) calcd for $C_{43}H_{68}O_4SiNa$ $[M + Na]^+$ 699.4779, found 699.4784.

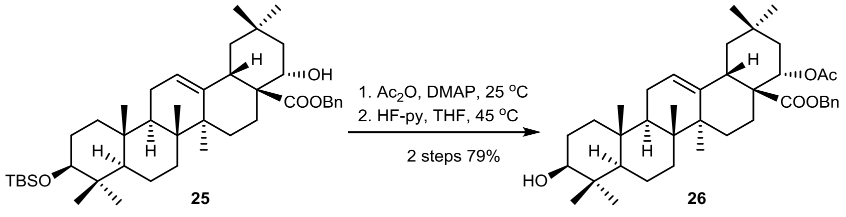

Compound 26 | To a solution of **25** (122 mg, 0.18 mmol, 1.0 equiv.) and DMAP (22 mg, 0.18

mmol, 1.0 equiv.) in pyridine (2 mL), was added Ac_2O (43 μL, 0.45 mmol, 2.5 equiv.). After stirring at 25 °C for 4 h, the mixture was poured into water, the excess reagents were quenched with Na_2CO_3. The mixture was extracted with CH_2Cl_2 (3 × 20 mL), and the organic layer was washed with saturated aqueous NaCl solution (30 mL), dried (Na_2SO_4), and concentrated under reduced pressure. The residue in THF (3 mL) was added hydrogen fluoride-pyridine (65% wt.%, 0.2 ml, 1.2 mmol, 6.0 equiv.) at 0 °C. The mixture was heated to 40 ℃ and stirred for 12 h, the excess reagents were slowly quenched with saturated aqueous $NaHCO_3$ solution, and then the resulting mixture was extracted with CH_2Cl_2 (3 × 20 mL). The combined organic layers were washed with saturated aqueous NaCl solution (50 ml), dried (Na_2SO_4), and concentrated under reduced pressure. Flash column chromatography (EtOAc/petroleum ether, 1:6) afforded **26** (96 mg, 79% over 2 steps) as a white solid: R_f = 0.2 (silica, PE/EtOAc = 6:1); $[\alpha]_D^{25}$ = 27.6 (*c* 1.0, $CHCl_3$); ^1H NMR (500 MHz, $CDCl_3$) δ 7.39–7.28 (m, 5H), 5.28 (t, *J* = 3.5 Hz, 1H), 5.23 (dd, *J* = 11.0, 6.3 Hz, 1H), 5.09 (d, *J* = 12.4 Hz, 1H), 4.92 (d, *J* = 12.4 Hz, 1H), 3.26–3.14 (m, 1H), 3.01–2.98 (m, 1H), 2.06–1.99 (m, 1H), 1.86 (s, 3H), 1.83–1.70 (m, 5H), 1.66–1.21 (m, 14H), 1.14 (s, 3H), 1.04 (s, 3H), 0.98 (s, 3H), 0.95 (s, 3H), 0.87 (s, 3H), 0.77 (s, 3H), 0.75–0.63 (m, 1H), 0.57 (s, 3H); ^{13}C NMR (126 MHz, $CDCl_3$) δ 174.40, 170.12, 141.95, 136.06, 128.36, 128.35, 127.96, 123.61, 78.96, 72.88, 66.65, 55.16, 51.00, 47.48, 44.90, 42.56, 41.75, 39.44, 39.06, 38.73, 38.42, 36.94, 32.88, 32.58, 31.43, 28.08, 27.16, 26.81, 26.32, 24.67, 23.38, 20.93, 18.27, 16.85, 16.82, 15.55, 15.22; ESI-HRMS (m/z) calcd for $C_{39}H_{56}O_5Na$ $[M + Na]^+$ 627.4020, found 627.4020.

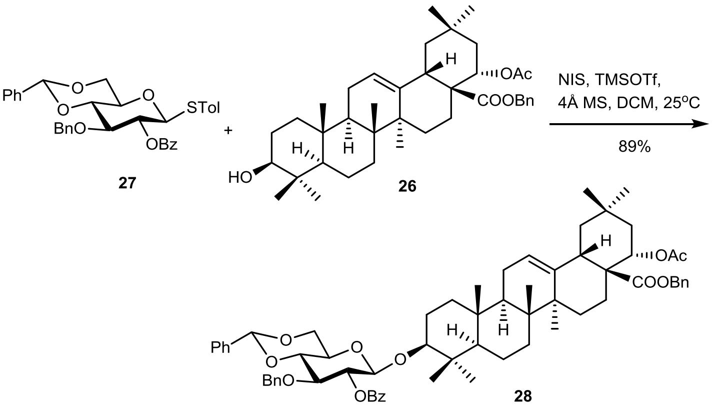

PT 3-*O*-glycoside 28 | A mixture of **26** (82 mg, 0.14 mmol, 1.0 equiv.), **27**[24] (93 mg, 0.16 mmol, 1.1 equiv.), and 4Å MS in dry CH_2Cl_2 (2 mL) was stirred at 25 °C for 30 min, and then cooled to -20 ℃. NIS (40 mg, 0.18 mmol, 1.3 equiv.) was added, followed by Me_3SiOTf (30 μl, 0.16 mmol, 1.1 equiv.). The resulting mixture was stirred for another 40 min before addition of Et_3N (1 mL), and was then filtered through a pad of Celite. The filtrates was concentrated under reduced pressure. Purification of the resulting residue by flash column chromatography (EtOAc/petroleum ether, 1:8) gave **28** (127 mg, 89%) as a white solid: R_f = 0.4 (silica, PE/EtOAc = 5:1); $[\alpha]_D^{25}$ = 34.1 (*c* 1.0, $CHCl_3$); ^1H NMR (500 MHz, $CDCl_3$) δ 8.02–7.97 (m, 2H), 7.58 (t, *J* = 7.4 Hz, 1H), 7.52 (dd, *J* = 7.7, 1.5 Hz, 2H), 7.47–7.36 (m, 5H), 7.35–7.30 (m, 5H), 7.14 (t, *J* = 6.6 Hz, 3H), 7.10–7.04 (m, 2H), 5.61 (s, 1H), 5.39–5.31 (m, 1H), 5.27 (t, *J* = 3.4 Hz, 1H), 5.24–5.19 (m, 1H), 5.09 (d, *J* = 12.3 Hz, 1H), 4.90 (d, *J* = 12.3 Hz, 1H), 4.82 (d, *J* = 12.2 Hz, 1H), 4.71 (d, *J* = 12.2 Hz, 1H), 4.65 (d, *J* = 7.9 Hz, 1H), 4.35 (dd, *J* = 10.5, 4.9 Hz, 1H), 3.88–3.84 (m, 3H), 3.50–3.45 (m, 1H), 3.07 (dd, *J* = 11.5, 4.7 Hz, 1H), 3.00–2.97 (m, 1H), 2.01 (d, *J* = 8.9 Hz, 1H), 1.86 (s, 3H), 1.83–1.53 (m, 9H), 1.46–1.12 (m, 10H), 1.10 (s, 3H), 1.04 (s, 3H), 0.94 (s, 3H), 0.90–0.87 (m, 1H),

0.82 (s, 3H), 0.66 (s, 3H), 0.61 (s, 3H), 0.51 (s, 3H); ^{13}C NMR (126 MHz, $CDCl_3$) δ 174.56, 170.31, 165.21, 142.12, 138.13, 137.49, 136.24, 133.15, 130.21, 130.04, 129.21, 128.58, 128.57, 128.48, 128.45, 128.33, 128.26, 128.19, 127.69, 126.22, 123.84, 104.06, 101.45, 90.38, 81.93, 78.12, 74.07, 73.96, 73.09, 69.04, 66.86, 66.40, 55.62, 51.18, 47.65, 45.11, 42.75, 41.90, 39.63, 39.26, 38.97, 38.61, 36.80, 33.11, 32.71, 31.64, 27.89, 26.98, 26.48, 26.09, 24.88, 23.59, 21.15, 18.23, 17.05, 16.97, 16.37, 15.34; ESI-HRMS (m/z) calcd for $C_{66}H_{80}O_{11}Na$ $[M + Na]^+$ 1071.5593, found 1071.5591.

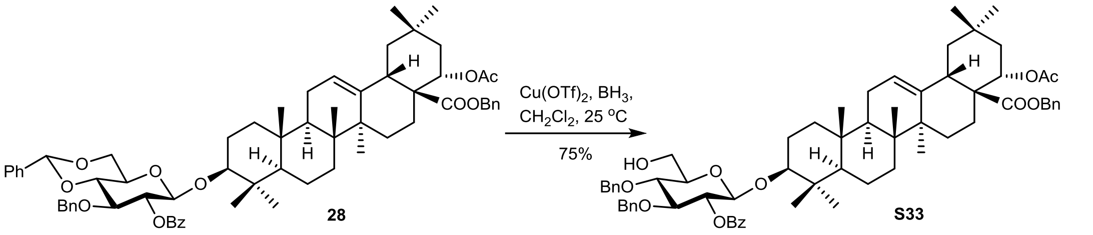

Compound S33 | To a solution of **28** (49 mg, 0.047 mmol, 1.0 equiv.) in CH_2Cl_2 (2 mL), was added a solution of borane/tetrahydrofuran complex in THF (1M, 0.24 mL, 0.24 mmol, 5.0 equiv.) at 25 °C. The mixture was stirred for 10 min, to which was added freshly dried copper(II) trifluoromethanesulfonate (2 mg, 0.005 mmol, 0.1 equiv.). After stirring for 3 h, the mixture was cooled down to 0 ℃, and the excess reagents were quenched by sequential additions of trimethylamine and methanol. The resulting mixture was concentrated at reduced pressure followed by co-evaporation with methanol. The residue was purified by flash column chromatography (EtOAc/petroleum ether, 2:3) on silica gel to give **S33** (36 mg, 75%): R_f = 0.5 (silica, PE/EtOAc = 1:1); $[\alpha]_D^{25}$ = 38.9 (*c* 1.0, $CHCl_3$); ^1H NMR (500 MHz, $CDCl_3$) δ 8.00 (d, *J* = 7.8 Hz, 2H), 7.56 (t, *J* = 7.4 Hz, 1H), 7.42 (t, *J* = 7.7 Hz, 2H), 7.38–7.27 (m, 10H), 7.17–7.10 (m, 5H), 5.31–5.26 (m, 2H), 5.22 (dd, *J* = 11.3, 5.8 Hz, 1H), 5.09 (d, *J* = 12.4 Hz, 1H), 4.91–4.86 (m, 2H), 4.75 (d, *J* = 11.2 Hz, 1H), 4.67 (dd, *J* = 11.0, 5.2 Hz, 2H), 4.60 (d, *J* = 7.9 Hz, 1H), 3.91–3.80 (m, 2H), 3.74–3.68 (m, 2H), 3.51–3.39 (m, 1H), 3.06 (dd, *J* = 10.7, 4.8 Hz, 1H), 2.98 (d, *J* = 10.7 Hz, 1H), 2.05–1.90 (m, 2H), 1.86 (s, 3H), 1.82–1.64 (m, 7H), 1.59–1.56 (m, 1H), 1.49–1.12 (m, 10H), 1.10 (s, 3H), 1.04 (s, 3H), 0.94 (s, 3H), 0.81 (s, 3H), 0.67 (s, 3H), 0.60 (s, 3H), 0.51 (s, 3H); ^{13}C NMR (126 MHz, $CDCl_3$) δ 174.33, 170.09, 165.06, 141.89, 137.75, 136.03, 132.95, 129.99, 129.73, 128.51, 128.36, 128.35, 128.27, 128.24, 128.12, 128.02, 127.99, 127.96, 127.64, 123.62, 103.32, 90.12, 82.62, 77.84, 75.17, 75.11, 75.03, 74.03, 72.87, 66.63, 62.10, 55.41, 50.97, 47.43, 44.88, 42.54, 41.68, 39.42, 39.05, 38.75, 38.41, 36.59, 32.89, 32.49, 31.43, 27.71, 26.77, 26.26, 26.10, 24.66, 23.36, 20.93, 18.02, 16.83, 16.75, 16.18, 15.12; ESI-HRMS (m/z) calcd for $C_{66}H_{82}O_{11}Na$ $[M + Na]^+$ 1073.5749, found 1073.5751.

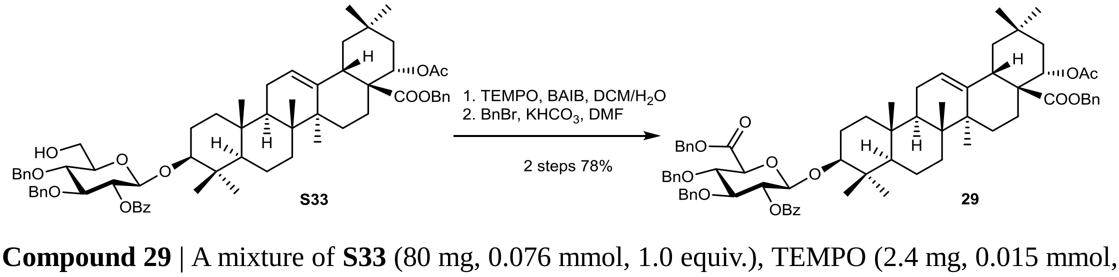

Compound 29 | A mixture of **S33** (80 mg, 0.076 mmol, 1.0 equiv.), TEMPO (2.4 mg, 0.015 mmol, 0.2 equiv.), and BAIB (61 mg, 0.19 mmol, 2.5 equiv.) in CH_2Cl_2 (1 mL), was added H_2O (1 mL).

After stirring at 25 °C for 1.5 h, the excess reagents were quenched with saturated aqueous $Na_2S_2O_3$ solution. The mixture was extracted with CH_2Cl_2 (3 × 20 mL), and combined organic layers were washed with saturated aqueous NaCl solution (100 mL), dried (Na_2SO_4), and concentrated under reduced pressure. The residue was dissolved in DMF (2 mL), to which were added BnBr (11 μL, 0.09 mmol, 1.2 equiv.) and $KHCO_3$ (19 mg, 0.19 mmol, 2.5 equiv.). After stirring at 25 °C for 2 h, the excess reagents were quenched with aqueous 1 M HCl solution, and then the resulting mixture was extracted with CH_2Cl_2 (3 × 20 mL). The combined organic layers were washed with saturated aqueous NaCl solution (30 mL), dried (Na_2SO_4), and concentrated under reduced pressure. Flash column chromatography (EtOAc/petroleum ether, 1:6) afforded **29** (68 mg, 78% for 2 steps) as a white solid: R_f = 0.2 (silica, PE/EtOAc = 5:1); $[\alpha]_D^{25}$ = 29.2 (*c* 1.1, $CHCl_3$); ^1H NMR (500 MHz, $CDCl_3$) δ 7.98 (d, *J* = 8.1 Hz, 2H), 7.55 (t, *J* = 7.4 Hz, 1H), 7.41 (t, *J* = 7.7 Hz, 2H), 7.35–7.28 (m, 12H), 7.20–7.07 (m, 7H), 5.33 (t, *J* = 8.6 Hz, 1H), 5.27 (s, 1H), 5.25–5.20 (m, 2H), 5.17 (d, *J* = 12.2 Hz, 1H), 5.09 (d, *J* = 12.4 Hz, 1H), 4.90 (d, *J* = 12.4 Hz, 1H), 4.73 (dd, *J* = 10.8, 8.1 Hz, 2H), 4.64 (d, *J* = 11.2 Hz, 1H), 4.60 (d, *J* = 7.8 Hz, 1H), 4.54 (d, *J* = 10.7 Hz, 1H), 4.02–3.96 (m, 2H), 3.81 (dd, *J* = 10.7, 4.4 Hz, 1H), 3.05 (dd, *J* = 11.5, 4.4 Hz, 1H), 2.99 (d, *J* = 10.3 Hz, 1H), 2.00 (d, *J* = 9.3 Hz, 1H), 1.85 (s, 3H), 1.83–1.61 (m, 7H), 1.56–1.51 (m, 1H), 1.47–1.12 (m, 10H), 1.09 (s, 3H), 1.04 (s, 3H), 0.94 (s, 3H), 0.91–0.83 (m, 2H), 0.81 (s, 3H), 0.66 (s, 3H), 0.61–0.59 (m, 1H), 0.58 (s, 3H), 0.51 (s, 3H); ^{13}C NMR (126 MHz, $CDCl_3$) δ 174.34, 170.09, 168.02, 164.93, 141.91, 137.72, 137.60, 136.03, 135.17, 132.97, 129.74, 128.55, 128.41, 128.35, 128.33, 128.27, 128.22, 128.01, 127.96, 127.92, 127.77, 127.65, 123.64, 103.61, 90.11, 81.97, 79.33, 74.98, 74.60, 73.58, 72.88, 67.17, 66.64, 55.36, 50.97, 47.41, 44.89, 42.54, 41.68, 39.42, 38.74, 36.56, 32.89, 32.49, 31.43, 27.67, 26.77, 26.26, 25.71, 24.67, 23.38, 20.93, 18.01, 16.83, 16.75, 16.12, 15.12; ESI-HRMS (m/z) calcd for $C_{73}H_{86}O_{12}Na$ $[M + Na]^+$ 1177.6011, found 1177.6016.

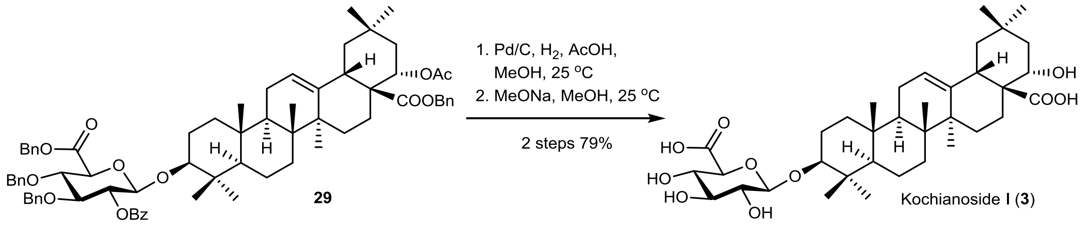

Kochianoside I (3) | To a solution of **29** (60 mg, 0.051 mmol, 1.0 equiv.) in MeOH (2 mL), were added Pd/C (15 mg) and AcOH (20 μL). The mixture was stirred at 25 °C under a hydrogen atmosphere until the reaction was complete as determined by TLC. The catalyst was removed by filtration through Celite. The filtrate was concentrated under reduced pressure. The residue was dissolved in MeOH (2 mL), to which NaOMe (10 mg, 0.19 mmol, 3.6 equiv.) was added. The mixture was stirred at 25 °C overnight, and the excess reagents were quenched with Amberlite IR-120 (H^+). Amberlite was removed through filtration and the filtrate was concentrated to give **3** (26 mg, 79%) as a white solid: R_f = 0.5 (ODS RP-18, MeOH/H_2O = 2:1); $[\alpha]_D^{25}$ = 19.8 (*c* 0.7, MeOH); ^1H NMR (500 MHz, pyridine-d_5) δ 5.49 (s, 1H), 5.06 (d, *J* = 7.0 Hz, 1H), 4.66–4.54 (m, 2H), 3.45–3.35 (m, 2H), 2.76–2.73 (m, 1H), 2.52–2.49 (m, 1H), 2.28–2.26 (m, 2H), 1.39 (s, 3H), 1.35 (s, 3H), 1.15 (s, 3H), 1.05 (s, 3H), 1.04 (s, 3H), 1.01 (s, 3H), 0.82 (s, 3H); ^{13}C NMR (126 MHz, pyridine-d_5) δ 179.40, 144.16, 123.05, 107.21, 89.00, 78.15, 77.76, 75.53, 73.49, 71.53, 55.70, 53.08, 47.92, 45.94, 43.49, 43.29, 42.50, 39.92, 39.50, 38.58, 36.89, 33.39, 33.00, 31.52,

28.18, 27.92, 26.75, 26.58, 25.13, 23.74, 18.44, 17.33, 16.94, 16.87, 15.36; ESI-HRMS (m/z) calcd for $C_{36}H_{56}O_{10}Na$ [M + Na]$^+$ 671.3766, found 671.3760.

Synthesis of Barringtogenol C (4)

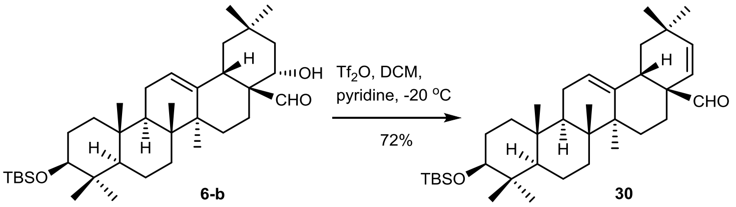

Compound 30 | To a solution of aldehyde **6-b** (3.0 g, 5.2 mmol, 1.0 equiv.) in CH_2Cl_2 (15 mL), were added pyridine (4.1 mL, 51.7 mmol, 10.0 equiv.) and Tf_2O (3.5 mL, 25.8 mmol, 5.0 equiv.) at -20 ℃. The mixture was stirred at the same temperature for 3 hours, the excess reagents were quenched with aq. $NaHCO_3$ (20 mL), and the resulting mixture was extracted with CH_2Cl_2 (3 × 10 mL). The combined organic phases were washed with brine (20 mL), dried over anhydrous Na_2SO_4, filtered, and concentrated under vacuum. The residue was purified by flash column chromatography (EtOAc/petroleum ether, 1:30) to give **30** (2.1 g, 72%) as a white powder: R_f = 0.4 (silica, PE/EtOAc = 15:1); $[\alpha]_D^{25}$ = -35.0 (*c* 1.0, $CHCl_3$); IR (film): ν_{max} = 2951, 2855, 1726, 1256, 1103, 771 cm^{-1}; ^1H NMR (500 MHz, $CDCl_3$) δ 9.34 (s, 1H), 5.61 (dd, *J* = 9.9, 1.1 Hz, 1H), 5.46 (t, *J* = 3.5 Hz, 1H), 5.12 (d, *J* = 9.9 Hz, 1H), 3.18 (dd, *J* = 11.3, 4.6 Hz, 1H), 2.84–2.78 (m, 1H), 1.12 (s, 3H), 1.04 (s, 3H), 1.00 (s, 3H), 0.92 (s, 3H), 0.90 (s, 3H), 0.88 (s, 9H), 0.77 (s, 3H), 0.74 (s, 3H), 0.03 (s, 6H); ^{13}C NMR (126 MHz, $CDCl_3$) δ 205.26, 142.51, 140.69, 124.46, 124.15, 79.59, 55.52, 53.10, 47.81, 45.41, 42.31, 39.53, 39.48, 39.27, 38.76, 38.62, 37.08, 37.03, 33.43, 33.40, 31.07, 28.71, 27.86, 27.80, 26.74, 26.08, 26.01, 24.38, 23.63, 18.65, 18.27, 17.28, 17.15, 16.30, 16.25, 15.71, 15.46, -3.58, -4.74; ESI-HRMS (m/z) calcd for $C_{36}H_{60}O_2SiNa$ [M + Na]$^+$ 575.4255, found 575.4255.

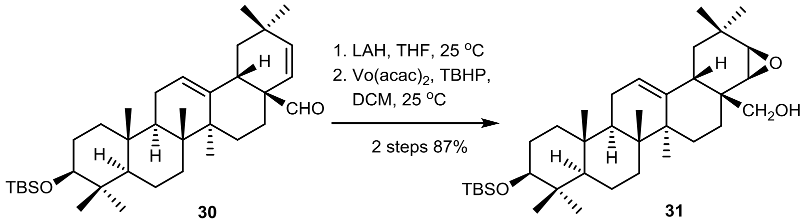

Compound 31 | To a solution of aldehyde **30** (3.0 g, 5.3 mmol, 1.0 equiv.) in THF (25 mL), was added $LiAlH_4$ (608 mg, 16.0 mmol, 3.0 equiv.). The mixture was stirred at 25 °C for 1 hour, the excess reagents were quenched with saturated aq. seignette salt solution (50 mL), and the resulting mixture was extracted with EtOAc (3 × 30 mL). The combined organic phases were washed with brine (20 mL), dried over anhydrous Na_2SO_4, filtered, and concentrated under vacuum. The residue was dissolved in CH_2Cl_2 (25 mL), to which $Vo(acac)_2$ (142 mg, 0.53 mmol, 0.1 equiv.) and TBHP (4.9 mL, 5.5 M in nonane, 26.7 mmol, 5.0 equiv.) were added. The mixture was stirred at the same temperature for 12 hours, the excess reagents were quenched with aq. Na_2SO_3 (20 mL), and the resulting mixture was extracted with CH_2Cl_2 (3 × 10 mL). The combined organic phases were washed with brine (20 mL), dried over anhydrous Na_2SO_4, filtered, and concentrated under

vacuum. The residue was purified by flash column chromatography (EtOAc/petroleum ether, 1:15) to give **31** (2.7 g, 87% over two steps) as a colorless oil: R_f = 0.4 (silica, PE/EtOAc = 5:1); $[\alpha]_D^{25}$ = 23.2 (*c* 0.5, $CHCl_3$); IR (film): ν_{max} = 3422, 2958, 2854, 1461, 1258, 1100, 802 cm^{-1}; ^1H NMR (500 MHz, $CDCl_3$) δ 5.24 (s, 1H), 3.65 (d, *J* = 10.9 Hz, 1H), 3.58 (d, *J* = 10.9 Hz, 1H), 3.18 (dd, *J* = 11.0, 4.3 Hz, 1H), 3.00 (d, *J* = 3.5 Hz, 1H), 2.84 (d, *J* = 3.2 Hz, 1H), 1.10 (s, 3H), 1.06 (s, 3H), 1.03 (s, 3H), 0.94 (s, 3H), 0.93 (s, 3H), 0.91 (s, 3H), 0.89 (s, 9H), 0.75 (s, 3H), 0.03 (s, 6H); ^{13}C NMR (126 MHz, $CDCl_3$) δ 142.52, 124.00, 79.61, 65.86, 61.09, 60.80, 55.46, 47.75, 45.09, 42.39, 39.89, 39.51, 38.78, 38.08, 38.03, 36.99, 33.02, 30.48, 29.64, 28.70, 27.80, 26.08, 25.32, 25.29, 23.91, 23.71, 21.01, 18.69, 18.28, 17.00, 16.28, 15.73, -3.58, -4.73; ESI-HRMS (m/z) calcd for $C_{36}H_{62}O_3SiNa$ $[M + Na]^+$ 593.4360, found 593.4364.

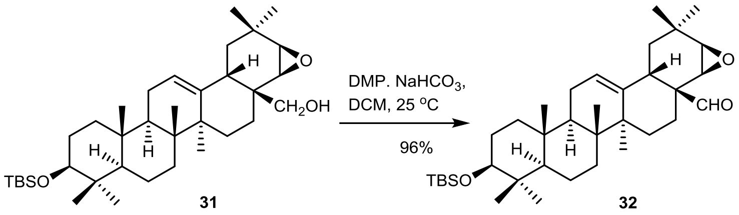

Compound 32 | To a stirred solution of alcohol **31** (4.2 g, 7.4 mmol, 1.0 equiv.) in CH_2Cl_2 (36 mL), were added $NaHCO_3$ (2.5 g, 29.4 mmol, 4.0 equiv.) and Dess Martin periodinane (11 g, 25.7 mmol, 3.5 equiv.). The mixture was stirred at 25 °C for 5 hours, the excess reagents were quenched with saturated aq. Na_2SO_3 (30 mL), and the resulting mixture was extracted with CH_2Cl_2 (3 × 30 mL). The combined organic phases were washed with brine (20 mL), dried over anhydrous Na_2SO_4, filtered, and concentrated under vacuum. The residue was purified by flash column chromatography (EtOAc/petroleum ether, 1:15) to give **32** (4.0 g, 96%) as a white powder: R_f = 0.4 (silica, PE/EtOAc = 10:1); $[\alpha]_D^{25}$ = 6.6 (*c* 1.0, $CHCl_3$); IR (film): ν_{max} = 2953, 2855, 1725, 1460, 1256, 1101, 772 cm^{-1}; ^1H NMR (500 MHz, $CDCl_3$) δ 9.63 (s, 1H), 5.41 (t, *J* = 3.5 Hz, 1H), 3.17 (dd, *J* = 11.1, 4.5 Hz, 1H), 2.99 (d, *J* = 3.6 Hz, 1H), 2.80 (d, *J* = 2.8 Hz, 1H), 2.69–2.63 (m, 1H), 1.09 (s, 6H), 1.08 (s, 3H), 0.90 (s, 3H), 0.89 (s, 3H), 0.88 (s, 9H), 0.74 (s, 3H), 0.74 (s, 3H), 0.03 (s, 6H); ^{13}C NMR (126 MHz, $CDCl_3$) δ 206.84, 141.47, 125.04, 79.56, 59.28, 58.76, 55.45, 50.00, 47.68, 44.46, 42.47, 39.74, 39.47, 38.69, 37.02, 36.63, 33.14, 30.24, 29.48, 28.70, 27.75, 26.49, 26.06, 25.04, 24.08, 23.67, 21.83, 18.60, 18.27, 17.19, 16.27, 15.59, -3.59, -4.75; ESI-HRMS (m/z) calcd for $C_{36}H_{60}O_3SiNa$ $[M + Na]^+$ 591.4204, found 591.4196.

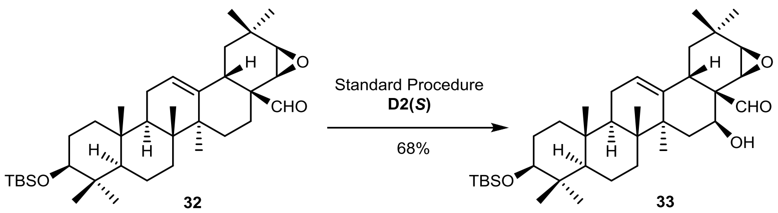

Compound 33 | This compound was prepared from substrate **32** (100 mg, 0.17 mmol, 1.0 equiv.) following the standard procedure in the presence of **D2(*S*)** (61 mg, 0.50 mmol, 3.0 equiv.). The residue was purified by flash column chromatography (EtOAc/petroleum ether, 1:6) to afford product **33** (70 mg, 68%) as a white foam: R_f = 0.4 (silica, PE/EtOAc = 3:1); $[\alpha]_D^{25}$ = -16.8 (*c* 0.2, $CHCl_3$); IR (film): ν_{max} = 2925, 2854, 1261, 1101, 1022, 801 cm^{-1}; ^1H NMR (400 MHz, $CDCl_3$) δ

9.66 (d, *J* = 2.8 Hz, 1H), 5.47 (t, *J* = 3.5 Hz, 1H), 4.18–4.08 (m, 1H), 3.73 (d, *J* = 3.6 Hz, 1H), 3.17 (dd, *J* = 11.0, 4.6 Hz, 1H), 2.90–2.75 (m, 3H), 1.14 (s, 3H), 1.12 (s, 3H), 1.10 (s, 3H), 0.91 (s, 3H), 0.90 (s, 3H), 0.88 (s, 9H), 0.77 (s, 3H), 0.74 (s, 3H), 0.03 (s, 6H); ^{13}C NMR (126 MHz, $CDCl_3$) δ 210.35, 140.19, 125.82, 79.51, 68.98, 58.99, 56.84, 55.43, 52.89, 46.87, 44.20, 43.83, 39.78, 39.49, 38.94, 38.68, 37.31, 36.95, 33.11, 30.14, 29.36, 28.71, 27.74, 26.06, 26.01, 24.22, 23.72, 18.56, 18.27, 17.29, 16.28, 15.60, -3.59, -4.74; ESI-HRMS (m/z) calcd for $C_{36}H_{60}O_4SiNa$ $[M + Na]^+$ 607.4153, found 607.4157.

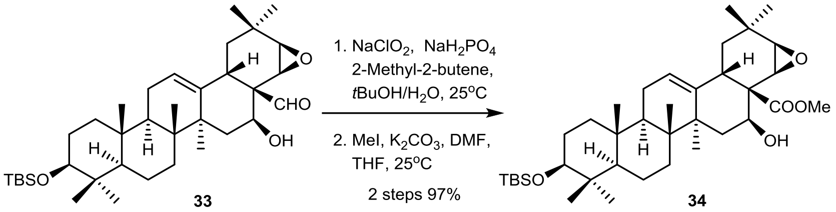

Compound 34 | To a solution of aldehyde **33** (500 mg, 0.85 mmol, 1.0 equiv.) and 2-methyl-2-butene (1 mL, 8.5 mmol, 10.0 equiv.) in *t*-BuOH (8.0 ml), was added a solution of $NaClO_2$ (80% purity, 387 mg, 4.3 mmol, 5.0 equiv.) and NaH_2PO_4•$2H_2O$ (718 mg, 6.0 mmol, 7.0 equiv.) in water (2.0 mL). After stirring at 25 °C for 3 h, the reaction mixture was diluted with an aqueous 10% NaOH solution (20 mL) and the aqueous phase was extracted with hexanes (50 ml). The aqueous phase was acidified with aqueous 1M HCl solution (to pH = 1) and extracted with CH_2Cl_2 (3 × 80 mL). The combined CH_2Cl_2 layers were dried (Na_2SO_4) and concentrated under reduced pressure. The residue was dissolved in DMF (50 mL) and THF (4.0 mL), to which K_2CO_3 (591 mg, 4.3 mmol, 5.0 equiv.) and MeI (0.22 mL, 3.4 mmol, 4.0 equiv.) were added. After stirring at 25 °C for 7 h, the excess reagents were quenched with MeOH (10 mL) and the resulting mixture was concentrated under reduced pressure. The residue was purified by flash column chromatography (petroleum ether/EtOAc, 5:1) afforded **34** (510 mg, 97% over 2 steps) as a white solid: R_f = 0.5 (silica, PE/EtOAc = 2:1); $[\alpha]_D^{25}$ = 18.9 (*c* 0.5, $CHCl_3$); IR (film): ν_{max} = 3507, 2926, 2853, 1700, 1462, 1260, 1074, 798 cm^{-1}; ^{1}H NMR (500 MHz, $CDCl_3$) δ 5.32 (s, 1H), 4.20–4.05 (m, 1H), 3.79 (d, *J* = 11.2 Hz, 1H), 3.75 (d, *J* = 3.5 Hz, 1H), 3.71 (s, 3H), 3.16 (dd, *J* = 11.1, 4.2 Hz, 1H), 2.90–2.83 (m, 2H), 1.12 (s, 3H), 1.08 (s, 3H), 1.06 (s, 3H), 0.89 (s, 3H), 0.89 (s, 3H), 0.87 (s, 9H), 0.73 (s, 6H), 0.01 (s, 6H); ^{13}C NMR (126 MHz, $CDCl_3$) δ 176.10, 141.00, 124.14, 79.44, 67.91, 61.11, 57.94, 55.37, 52.45, 51.44, 46.86, 44.46, 43.83, 39.42, 39.38, 38.87, 38.55, 38.09, 36.93, 32.89, 30.12, 29.33, 28.64, 27.69, 26.22, 26.03, 24.30, 23.57, 18.55, 18.22, 16.87, 16.22, 15.52, -3.63, -4.78; ESI-HRMS (m/z) calcd for $C_{37}H_{62}O_5SiNa$ $[M + Na]^+$ 637.4259, found 637.4260.

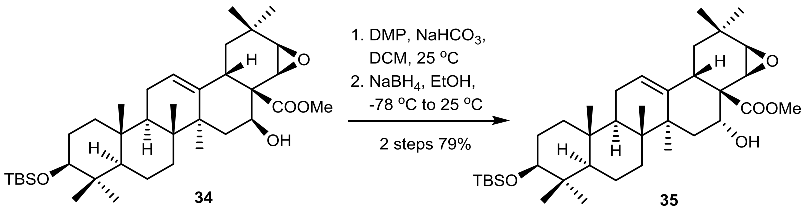

Compound 35 | To a stirred solution of ester **34** (446 mg, 0.73 mmol, 1.0 equiv.) in CH_2Cl_2 (14 mL) were added $NaHCO_3$ (183 mg, 2.2 mmol, 3.0 equiv.) and Dess Martin periodinane (617 mg,

1.5 mmol, 2.0 equiv.). The mixture was stirred at 25 °C for 3 hours, the excess reagents were quenched with saturated aq. Na_2SO_3 (100 mL), and the resulting mixture was extracted with CH_2Cl_2 (3 × 10 mL). The combined organic phases were washed with brine (20 mL), dried over anhydrous Na_2SO_4, filtered, and concentrated under vacuum. The residue was dissolved in EtOH (11 mL), to which $NaBH_4$ (129 mg, 3.4 mmol, 4.7 equiv.) was added at -78 ℃. After stirring at -78 ℃ for 2 h and then at 25 °C for 8 h, the excess reagents were quenched with saturated aq. NH_4Cl (10 mL), and the resulting mixture was extracted with CH_2Cl_2 (3 × 10 mL). The combined organic phases were washed with brine (10 mL), dried over anhydrous Na_2SO_4, filtered, and concentrated under vacuum. The residue was purified by flash column chromatography (EtOAc/petroleum ether, 1:5) to give **35** (352 mg, 79% over two steps) as a colorless oil: R_f = 0.4 (silica, PE/EtOAc = 3:1); $[\alpha]_D^{25}$ = 12.6 (*c* 0.5, $CHCl_3$); IR (film): ν_{max} = 2926, 2853, 1743, 1261, 1101, 809 cm^{-1}; ^1H NMR (500 MHz, $CDCl_3$) δ 5.37 (t, *J* = 3.4 Hz, 1H), 4.72–4.68 (m, 1H), 3.66 (s, 3H), 3.39 (d, *J* = 3.7 Hz, 1H), 3.17 (dd, *J* = 11.1, 4.5 Hz, 1H), 2.92 (d, *J* = 3.5 Hz, 1H), 2.84–2.78 (m, 1H), 2.40–2.32 (m, 1H), 1.32 (s, 3H), 1.07 (s, 3H), 1.04 (s, 3H), 0.90 (s, 3H), 0.89 (s, 3H), 0.87 (s, 9H), 0.73 (s, 3H), 0.72 (s, 3H), 0.02 (s, 6H); ^{13}C NMR (126 MHz, $CDCl_3$) δ 173.97, 141.88, 123.71, 79.51, 72.04, 61.69, 60.27, 55.41, 52.38, 50.75, 46.74, 44.97, 41.69, 39.56, 39.43, 38.58, 37.02, 36.41, 34.55, 33.15, 29.91, 29.09, 28.64, 27.73, 26.94, 26.05, 24.45, 23.50, 18.60, 18.24, 16.91, 16.25, 15.56, -3.61, -4.78; ESI-HRMS (m/z) calcd for $C_{37}H_{62}O_5SiNa$ $[M + Na]^+$ 637.4259, found 637.4260.

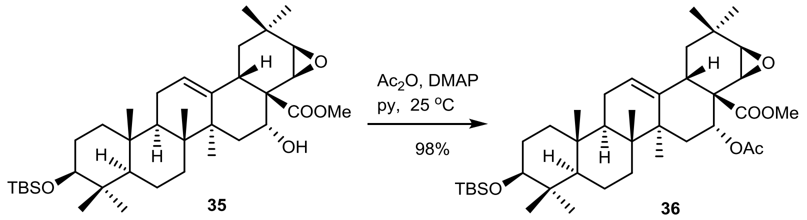

35 36

Compound 36 | To a stirred solution of 16-ol **35** (324 mg, 0.53 mmol, 1.0 equiv.) in pyridine (6.0 mL) were added DMAP (970 mg, 7.9 mmol, 15.0 equiv.) and Ac_2O (0.5 mL, 5.3 mmol, 10.0 equiv.). The mixture was stirred at 25 °C for 6 hours, the excess reagents were quenched with saturated aq. $NaHCO_3$ (10 mL), and the resulting mixture was extracted with CH_2Cl_2 (3 × 10 mL). The combined organic phases were washed with brine (10 mL), dried over anhydrous Na_2SO_4, filtered, and concentrated under vacuum. The residue was purified by flash column chromatography (EtOAc/petroleum ether, 1:15) to give **36** (340 mg, 98%) as a colorless oil: R_f = 0.4 (silica, PE/EtOAc = 5:1); $[\alpha]_D^{25}$ = 1.4 (*c* 0.5, $CHCl_3$); IR (film): ν_{max} = 2962, 2856, 1747, 1261, 1099, 800 cm^{-1}; ^1H NMR (500 MHz, $CDCl_3$) δ 5.80–5.77 (m, 1H), 5.43 (t, *J* = 3.4 Hz, 1H), 3.70 (s, 3H), 3.26 (d, *J* = 3.5 Hz, 1H), 3.17 (dd, *J* = 11.1, 4.6 Hz, 1H), 2.91–2.83 (m, 2H), 2.23–2.16 (m, 1H), 2.09 (s, 3H), 1.20 (s, 3H), 1.09 (s, 3H), 1.07 (s, 3H), 0.91 (s, 3H), 0.88 (s, 3H), 0.88 (s, 9H), 0.73 (s, 6H), 0.02 (s, 6H); ^{13}C NMR (126 MHz, $CDCl_3$) δ 172.45, 169.99, 140.74, 124.67, 79.53, 73.24, 61.11, 59.03, 55.49, 52.69, 50.00, 46.83, 44.76, 41.52, 39.66, 39.45, 38.63, 37.06, 36.40, 33.21, 31.48, 30.12, 29.06, 28.66, 27.74, 26.44, 26.05, 24.32, 23.53, 21.75, 18.57, 18.26, 16.98, 16.24, 15.58, -3.60, -4.75; ESI-HRMS (m/z) calcd for $C_{39}H_{64}O_6SiNa$ $[M + Na]^+$ 679.4364, found 679.4366.

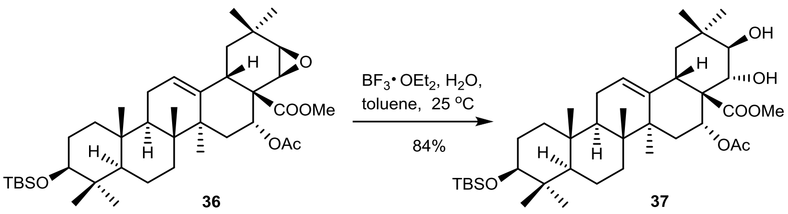

Compound 37 | To a stirred solution of epoxide **36** (45 mg, 0.068 mmol, 1.0 equiv.) in toluene (1.5 mL), were added boron trifluoride etherate (13 μL, 0.10 mmol, 1.5 equiv.) and H_2O (50 μL). The mixture was stirred at 25 °C for 6 hours, the excess reagents were quenched with saturated aq. Na_2SO_3 (3.0 mL), and the resulting mixture was extracted with CH_2Cl_2 (3 × 3.0 mL). The combined organic phases were washed with brine (5.0 mL), dried over anhydrous Na_2SO_4, filtered, and concentrated under vacuum. The residue was purified by flash column chromatography (EtOAc/petroleum ether, 1:1) to give **37** (39 mg, 84%) as a colorless oil: R_f = 0.4 (silica, PE/EtOAc = 1:2); $[\alpha]_D^{25}$ = -3.1 (*c* 0.2, $CHCl_3$); IR (film): ν_{max} = 3446, 2925, 2854, 1743, 1457, 1259, 801 cm^{-1}; ^1H NMR (500 MHz, $CDCl_3$) δ 5.83 (br s, 1H), 5.43 (br s, 1H), 3.68 (s, 3H), 3.64 (d, *J* = 9.1 Hz, 1H), 3.53 (d, *J* = 9.5 Hz, 1H), 3.18 (dd, *J* = 11.0, 4.4 Hz, 1H), 3.14–3.08 (m, 1H), 2.83 (br s, 1H), 2.43 (br s, 1H), 2.32–2.24 (m, 1H), 2.09 (s, 3H), 1.89 (dd, *J* = 8.7, 3.2 Hz, 2H), 1.85–1.80 (m, 1H), 1.24 (s, 3H), 1.04 (s, 3H), 1.00 (s, 3H), 0.91 (s, 3H), 0.89 (s, 3H), 0.88 (s, 9H), 0.73 (s, 3H), 0.69 (s, 3H), 0.03 (s, 6H); ^{13}C NMR (126 MHz, $CDCl_3$) δ 174.59, 169.95, 140.39, 124.69, 79.51, 77.44, 75.61, 70.34, 55.48, 54.95, 52.79, 46.79, 46.58, 41.36, 40.41, 39.70, 39.46, 38.63, 37.05, 35.23, 33.05, 31.83, 29.86, 28.66, 27.75, 26.67, 26.06, 23.52, 22.28, 18.72, 18.55, 18.27, 16.88, 16.25, 15.57, -3.59, -4.74; ESI-HRMS (m/z) calcd for $C_{39}H_{66}O_7SiNa$ $[M + Na]^+$ 697.4470, found 697.4470.

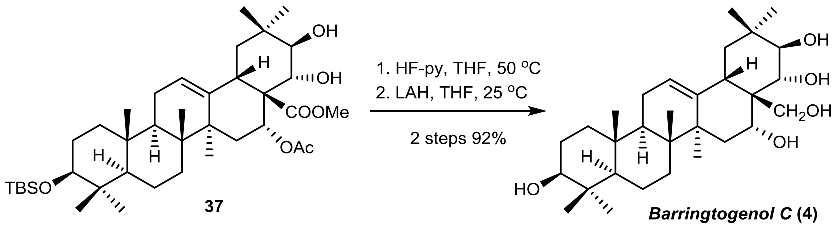

Barringtogenol C 4 | To a stirred solution of **37** (187 mg, 0.28 mmol, 1.0 equiv.) in THF (3.1 mL), was added hydrogen fluoride-pyridine (65% wt.%, 1.4 mL) at 0 ℃. The mixture was heated to 50 ℃ and stirred for 20 h, and the excess reagents wereslowly quenched with a saturated aqueous $NaHCO_3$ solution. The mixture was extracted with CH_2Cl_2 (3 × 5.0 mL), and the combined organic layers were washed with brine (5.0 mL), dried over anhydrous Na_2SO_4, filtered, and concentrated under vacuum. The residue was dissolved in THF (10 mL), to which was added $LiAlH_4$ (64 mg, 1.7 mmol, 6.0 equiv.). The mixture was stirred at 25 °C for 16 hours, the excess reagents were quenched with saturated aq. seignette salt solution (10 mL), and the resulting mixture was extracted with EtOAc (3 × 10 mL). The combined organic phases were washed with brine (10 mL), dried over anhydrous Na_2SO_4, filtered, and concentrated under vacuum. The residue was purified by column chromatography (DCM/MeOH, 15:1) to give **4** (125 mg, 92% over two steps) as a white solid: R_f = 0.5 (silica, DCM/MeOH = 9:1); $[\alpha]_D^{25}$ = 17.3 (*c* 0.2, $CHCl_3$); IR (film): ν_{max} = 3347, 2922, 2852, 1735, 1463, 1261, 801 cm^{-1}; ^1H NMR (400 MHz, pyridine-d5) δ 5.84 (s, 1H), 5.44 (s, 1H), 5.02 (s, 1H), 4.79 (d, *J* = 9.4 Hz, 1H), 4.64 (d, *J* = 9.4 Hz, 1H), 4.02 (d,

J = 10.3 Hz, 1H), 3.73 (d, J = 10.3 Hz, 1H), 3.47 (dd, J = 10.0, 5.3 Hz, 1H), 3.07–3.00 (m, 1H), 2.84–2.78 (m, 1H), 2.14–2.08 (m, 1H), 1.85 (s, 3H), 1.39 (s, 3H), 1.33 (s, 3H), 1.23 (s, 3H), 1.06 (s, 3H), 0.96 (s, 6H), 0.92–0.87 (m, 1H); ^{13}C NMR (126 MHz, pyridine-d5) δ 144.24, 123.31, 78.93, 78.37, 77.47, 68.60, 68.16, 56.13, 48.54, 47.64, 47.49, 42.36, 41.52, 40.40, 39.72, 39.48, 37.57, 36.75, 34.62, 33.58, 30.90, 29.07, 28.45, 27.74, 24.23, 19.80, 19.11, 17.33, 16.93, 16.18; ESI-HRMS (m/z) calcd for $C_{30}H_{50}O_5Na$ [M + Na]$^+$ 513.3550, found 513.3558.

Supplementary References

1. Dearmas, P., Concepcion, J. I., Francisco, C. G., Hernandez, R., Salazar, J. A. & Suarez, E. Intramolecular hydrogen abstraction - Hypervalent organoiodine compounds, convenient reagents for alkoxyl radical generation. *J. Chem. Soc. Perkin. 1*, 405-411 (1989).
2. Voica, A.-F., Mendoza, A., Gutekunst, W. R., Fraga, J. O. & Baran, P. S. Guided desaturation of unactivated aliphatics. *Nat. Chem.* **4**, 629-635 (2012).
3. Chen, M. S. & White, M. C. A predictably selective aliphatic C-H oxidation reaction for complex molecule synthesis. *Science* **318**, 783-787 (2007).
4. Liu, T., Myers, M. C. & Yu, J.-Q. Copper-catalyzed bromination of C(sp^3)-H bonds distal to functional groups. *Angew. Chem. Int. Ed.* **56**, 306-309 (2017).
5. Zhu, R.-Y., Saint-Denis, T. G., Shao, Y., He, J., Sieber, J. D., Senanayake, C. H. & Yu, J.-Q. Ligand-enabled Pd(II)-catalyzed bromination and iodination of C(sp^3)-H bonds. *J. Am. Chem. Soc.* **139**, 5724-5727 (2017).
6. Xu, Y., Yan, G.-B., Ren, Z & Dong, G.-B. Diverse sp^3 C-H functionalization through alcohol β-sulfonyloxylation. *Nat. Chem.* **7**, 829-834 (2015).
7. Huang, Z.-X, Wang, C.-P & Dong, G.-B. A hydrazone-based *exo*-directing-group strategy for C-H oxidation of aliphatic amines. *Angew. Chem. Int. Ed.* **55**, 5299-5303 (2016).
8. Desai, L. V., Hull, K. L. & Sanford, M. S. Palladium-catalyzed oxygenation of unactivated sp^3 C-H bonds. *J. Am. Chem. Soc.* **126**, 9542-9543 (2004).
9. Yu, J.-Q., Deng, A.-J., Wu, L.-Q., Zhang, Z.-H., Liu, Y., Wang, W.-J. & Qin, H.-L. Osteoclast-inhibiting saikosaponin derivatives from *Bupleurum Chinense*. *Fitoterapia* **85**, 101-108 (2013).
10. Carpani, G., Orsini, F., Sisti, M. & Verotta, L. Saponins from *Albizzia-Anthelmintica*. *Phytochemistry* **28**, 863-866 (1989).
11. Yoshikawa, M., Dai, Y., Shimada, H., Morikawa, T., Matsumura, N., Yoshizumi, S., Matsuda, H., Matsuda, H. & Kubo, M. Studies on Kochiae Fructus .Ⅱ. On the saponin constituents from the fruit of Chinese *Kochia scoparia* (Chenopodiaceae): Chemical structures of kochianosides I, II, III, and IV. *Chem Pharm Bull* **45**, 1052-1055 (1997).
12. Tuntiwachwuttikul P., Pancharoen, O., Mahabusarakam, W., Wiriyachitra, P., Taylor, W. C., Bubb, W. A. & Towers, G. H. N. A triterpenoid saponin from *Maesa Ramentacea*. *Phytochemistry* **44**, 491-495 (1997).
13. Qiu, S.-X., Gong, Y. & Cheung, H. T. A. A triterpene from *Marsdenia globifera*. *Phytochemistry* **34**, 1385-1387 (1993).

14. Li, H., Zou, H., Gao, L.-X., Liu, T., Yang, F., Li, J.-Y., Li, J., Qiu, W.-W. & Tang, J. Synthesis and biological evalution of oleanolic acid derivatives as novel inhibitors of protein tyrosine phosphatase 1B. *Heterocycles* **85**, 1117-1139 (2012).
15. Serbian, I. & Csuk, R. An improved scalable synthesis of α- and β-Amyrin. *Molecules* **23**, 1552 (2018).
16. You, R., Long, W.-Y., Lai, Z.-H., Sha, L., Wu, K., Yu, X., Lai, Y.-S., Ji, H., Huang, Z.-J. & Zhang, Y. -H. Discovery of a potential anti-inflammatory agent: 3-Oxo-29-noroleana -1,9(11),12-trien-2,20-dicarbonitrile. *J. Med. Chem.* **56**, 1984-1995 (2013).
17. Li, B., Cai, S., Yang, Y.-A., Chen, S.-C., Chen, R., Shi, J.-B., Liu, X.-H. & Tang, W.-J. Novel unsaturated glycyrrhetic acids derivatives: Design, synthesis and anti-inflammatory activity. *Eur. J. Med. Chem.* **139**, 337-348 (2017).
18. Flekhter, O. B., Ashavina, O. Y., Boreko, E. I., Karachurina, L. T., Pavlova, N. I., Kabal'Nova, N. N., Savinova, O. V., Galin, F. Z., Nikolaeva, S. N., Zarudii, F. S., Baltina, L. A. & Tolstikov, G. A. Synthesis of 3-*O*-acetylbetulinic and betulonic aldehydes according to Swern and the pharmacological Aactivity of related oximes. *Pharm. Chem. J.* **36**, 303-306 (2002).
19. See, Y. Y., Herrmann, A. T., Aihara, Y. & Baran, P. S. Scalable C-H oxidation with copper: Synthesis of polyoxypregnanes. *J. Am. Chem. Soc.* **137**, 13776-13779 (2015).
20. Chelucci, G., Baldino, S., Solinas, R. & Baratta W. Asymmetric synthesis of 1-substituted-1-(pyridin-2-yl)methylamines by diastereoselective reduction of enantiopure N-*p*-toluenesulfinyl ketimines. *Tetrahedron Lett.* **46**, 5555-5558 (2005).
21. Schonecker, B., Zheldakova, T., Liu, Y., Kotteritzsch, M., Gunther, W. & Gorls, H. Biomimetic hydroxylation of nonactivated CH_2 groups with copper complexes and molecular oxygen. *Angew. Chem. Int. Ed.* **42**, 3240-3244 (2003).
22. Li, Y., Yang, X., Liu, Y., Zhu, C., Yang, Y. & Yu, B. Gold(I)-catalyzed glycosylation with glycosyl ortho-alkynylbenzoates as donors: general scope and application in the synthesis of a cyclic triterpene saponin. *Chem. Eur. J.* **16**, 1871-1882 (2010).
23. Mukhopadhyay, B., Kartha, K. P. R., Russell, D. A & Field, R. A. Streamlined synthesis of per-*O*-acetylated sugars, glycosyl iodides, or thioglycosides from unprotected reducing sugars. *J. Org. Chem.* **69**, 7758-7760 (2004).
24. Weishaupt, M. W., Matthies, S., Hurevich, M., Pereira, C. L., Hahm, H. S. & Seeberger P. H. Automated glycan assembly of a *S. pneumoniae serotype* 3 CPS antigen. *Beilstein J. Org. Chem.* **12**, 1440-1446 (2016).
